# TRBC1-CAR T cell therapy in peripheral T cell lymphoma: a phase 1/2 trial

**DOI:** 10.1038/s41591-024-03326-7

**Published:** 2024-11-11

**Authors:** Kate Cwynarski, Gloria Iacoboni, Eleni Tholouli, Tobias Menne, David A. Irvine, Nivetha Balasubramaniam, Leigh Wood, Justin Shang, Eric Xue, Yiyun Zhang, Silvia Basilico, Margarida Neves, Meera Raymond, Ian Scott, Mohamed El-Kholy, Ram Jha, Heather Dainton-Smith, Rehan Hussain, William Day, Mathieu Ferrari, Simon Thomas, Koki Lilova, Wolfram Brugger, Teresa Marafioti, Pierre Lao-Sirieix, Paul Maciocia, Martin Pule

**Affiliations:** 1 Department of Haematology, University College London, London, UK; 2 University College London Hospital, London, UK; 3 Department of Haematology, Vall d’Hebron University Hospital, Barcelona, Spain; 4 Department of Haematology, Manchester Royal Infirmary, Manchester, UK; 5 The Newcastle upon Tyne Hospitals NHS Foundation Trust, Newcastle upon Tyne, UK; 6 Queen Elizabeth University Hospital, Glasgow, UK; 7 Autolus Therapeutics, London, UK

**Keywords:** Phase I trials, Haematological diseases

## Abstract

Relapsed/refractory peripheral T cell lymphomas (PTCLs) are aggressive tumors with a poor prognosis. Unlike B cell lymphomas, treatment of PTCL has not benefited from advances in immunotherapy. This is largely due to a lack of suitable target antigens that discriminate malignant from normal T cells, thus avoiding severe immunosuppression consequent to depletion of the entire T cell compartment. We recently described a targeting strategy based on the mutually exclusive expression of T cell antigen receptor beta-chain constant domain (TRBC) 1 and 2. Selective targeting of the T cell antigen receptor beta-chain expressed by the (clonal) malignancy spares normal T cells expressing the other chain. The LibraT1 study is an ongoing, multicenter, international, single-arm phase 1/2 study of TRBC1-directed autologous chimeric antigen receptor (CAR) T cells (AUTO4) in relapsed/refractory TRBC1-positive PTCL. Primary objectives were assessment of safety and tolerability of AUTO4 infusion. Key secondary endpoints included efficacy, CAR T cell expansion and persistence. Here we describe the findings from dose escalation in LibraT1 in the first ten patients, in a non-prespecified interim analysis. AUTO4 resulted in low frequency of severe immunotoxicity, with one of ten patients developing grade 3 cytokine release syndrome. Complete metabolic response was observed in four of ten evaluable patients, with remissions being durable beyond 1 year in two patients. While an absence of circulating CAR T cells was observed, CAR T cells were readily detected in lymph node biopsy samples from sites of original disease suggesting homing to tumor sites. These results support the continuing exploration of TRBC1 targeting in PTCL. ClinicalTrials.gov registration: NCT03590574.

## Abstract (web summary)

In the dose-escalation part of the ongoing phase 1/2 LIBRA4 trial, patients with relapsed/refractory peripheral T cell lymphoma received T cell antigen receptor beta-chain constant domain 1 (TRBC1)-targeted chimeric antigen receptor T cell therapy, showing limited toxicity and encouraging preliminary clinical responses.

## Main

PTCLs are a heterogeneous group of aggressive disorders representing 10–15% of non-Hodgkin lymphoma (NHL) and approximately 3% of all hematological malignancies^1,2^. First-line treatment for the most common histological subtypes typically consists of CHOP-like chemotherapy (cyclophosphamide, doxorubicin, vincristine and prednisolone) with or without etoposide, and brentuximab vedotin for the CD30^+^ anaplastic large cell lymphoma (ALCL) subtype^3^. Consolidation with high-dose chemotherapy and autologous hematopoietic stem cell transplant is a consideration for medically fit patients in remission. Despite this, the majority of patients with PTCLs have refractory or relapsed disease following initial treatment^2^. The median progression-free survival (PFS) and overall survival (OS) for patients with relapsed/refractory (r/r) PTCL is less than 6 months^2^.

A lack of suitable surface targets that distinguish malignant from normal T cells has hampered the development of targeted immunotherapies in PTCL. In contrast, B cell lymphomas can be readily targeted using pan-B cell antigens because concomitant depletion of the healthy B cell compartment is tolerable. CAR T cell therapies targeting CD19 result in durable responses in patients with refractory B-NHL^4^ and other B cell malignancies. An analogous approach for PTCL, where CAR T cells target pan-T cell antigens, could theoretically result in unacceptable profound and prolonged cellular immunosuppression.

The α/β T cell antigen receptor (TCR) complex is expressed by most healthy T cells and by nearly all cases of PTCL^5^. Often forgotten, the TCR β-chain constant region is encoded by two genes: *TRBC1* and *TRBC2*. During TCR gene rearrangement, productive TCRα requires pairing with either TRBC1 or TRBC2. Thus, physiological T cells are a mixture of TRBC1/TRBC2 T cells, at a TRBC1:TRBC2 ratio of approximately 2:3. T cell lymphomas, however, being clonal, exclusively express either TRBC1 or TRBC2. We previously proposed a strategy for immunotherapy of PTCL exploiting this aspect of TCR gene rearrangement^6^. In patients with a TRBC1 PTCL, CAR T cells directed against TRBC1 should target the lymphoma, but spare healthy TRBC2 T cells. A converse approach would be used for TRBC2 lymphomas.

AUTO4 is a TRBC1-targeting CAR T cell therapeutic. AUTO4 is manufactured from autologous T cells that are transduced with a bi-cistronic γ-retroviral vector encoding a TRBC1-specific CAR, coexpressed with the sort-suicide gene *RQR8* (ref. ^7^). The TRBC1-CAR is derived from a humanized form of the Jovi-1 antibody, which has a high selectivity for TRBC1 (ref. ^6^), and has a 41BB-ζ endodomain^8^. While AUTO4 can concomitantly target nonmalignant TRBC1^+^ T cells, T cells expressing TRBC2 may be spared, preserving functional cellular immunity^6^. T cell fratricide during manufacture is avoided by depletion of TRBC1^+^ T cells before transduction.

LibraT1 is an ongoing phase 1/2 multicenter clinical trial of AUTO4 CAR T cells in TRBC1^+^ r/r PTCL-not otherwise specified (PTCL-NOS), angioimmunoblastic T cell lymphoma (AITL) and ALCL (NCT03590574). In LibraT1, diagnostic material from patients with r/r disease is first screened by either immunohistochemistry (IHC) for TRBC1 or next-generation sequencing (NGS) for TRBC1/2 subtyping. Eligible patients with TRBC1 clonal disease then undergo leukapheresis to facilitate AUTO4 manufacture. Study participants receive lymphodepletion with fludarabine (Flu) and cyclophosphamide (Cy) before AUTO4 infusion, which is administered as a single intravenous infusion. LibraT1 was designed as a dose-escalation study. Here we show the ad hoc interim report data from the first ten treated patients.

## Results

### Patient characteristics

**Fig. 1:**
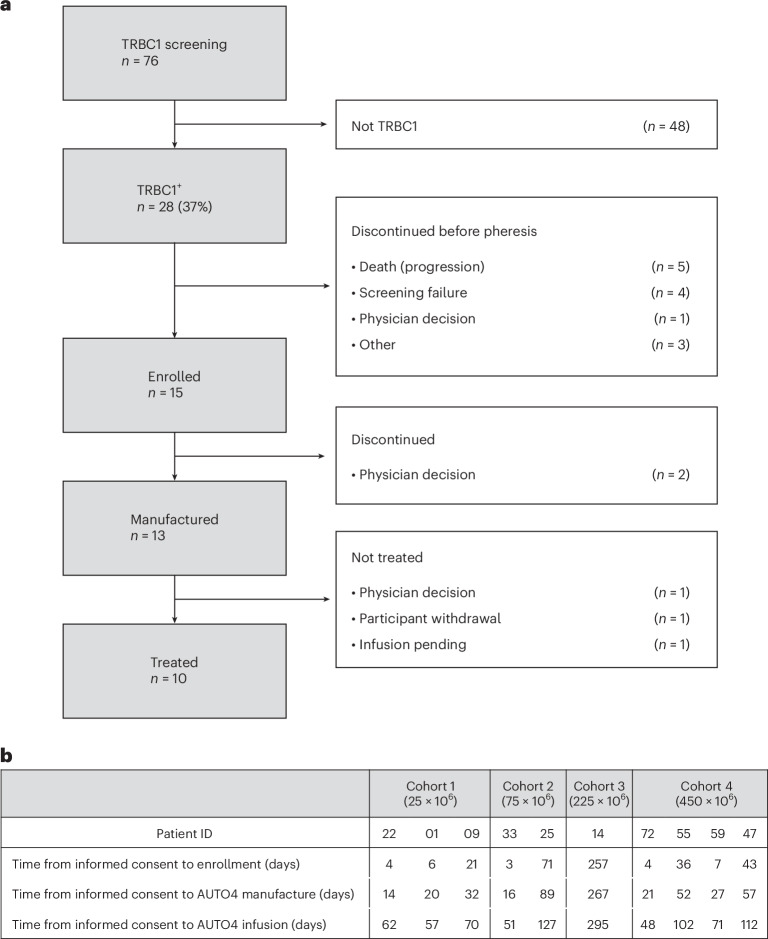
CONSORT diagram. **a**, CONSORT diagram for AUTO4/LibraT1 study (NCT03590574). **b**, Timeline from informed consent to enrollment (leukapheresate), AUTO4 CAR T cell manufacturing and AUTO4 CAR T cell infusion for treated patients.

**Extended Data Fig. 1:**
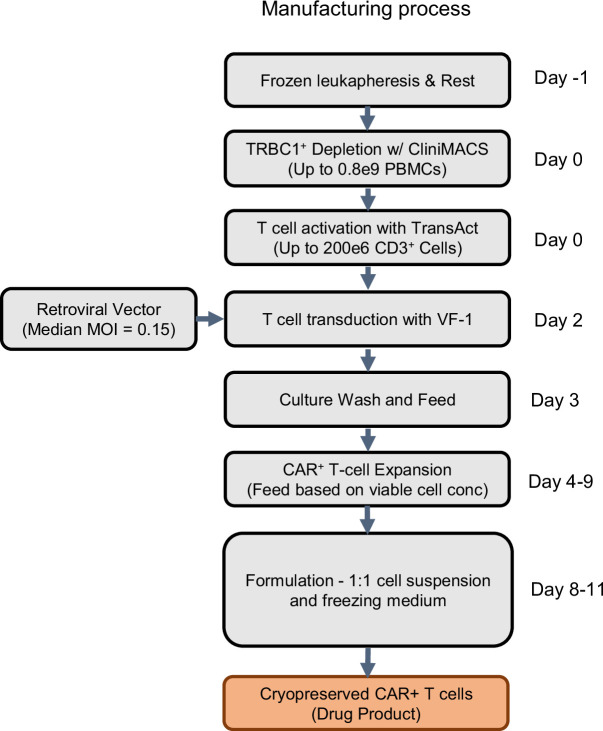
CAR T manufacturing. Diagram of manufacturing process for AUTO4 CAR-T cells.

As of 28 April 2023, diagnostic material from 76 patients with r/r PTCL (PTCL-NOS, AITL or ALCL) was screened by NGS or IHC. Twenty-eight (37%) patients were found to be TRBC1^+^. Subsequent eligibility required fluorodeoxyglucose (FDG)-avid measurable disease on positron emission tomography with computed tomography (PET-CT) according to the Lugano classification and an Eastern Cooperative Oncology Group Performance status of 0 or 1 and lack of central nervous system (CNS) disease (see Methods and study plan in Supplementary Information). Thirteen patients discontinued before apheresis (5 due to progressive disease; 4 due to screen failure; 2 were outside the allowable screening window for treatment; one due to patient/physician choice; and one due to septic shock/multi-organ failure). Fifteen patients were apheresed and enrolled (first patient enrolled 24 September 2018 and last patient enrolled 16 March 2022). AUTO4 was successfully manufactured for 13 patients (manufacture failed in 2 patients—one due to transduction failure and another due to target dose not reached). Ten patients were dosed with AUTO4. Patients were dosed in one of four cohorts: 25 × 10^6^, 75 × 10^6^, 225 × 10^6^ or 450 × 10^6^ CAR T cells (see CONSORT diagram in Fig. 1, manufacturing diagram in Extended Data Fig. 1 and study objectives in Extended Data Table 1). The study allowed rapid single patient dose escalation in the 75 × 10^6^ and 225 × 10^6^ cohorts if no toxicity was observed.

**Extended Data Fig. 2:**
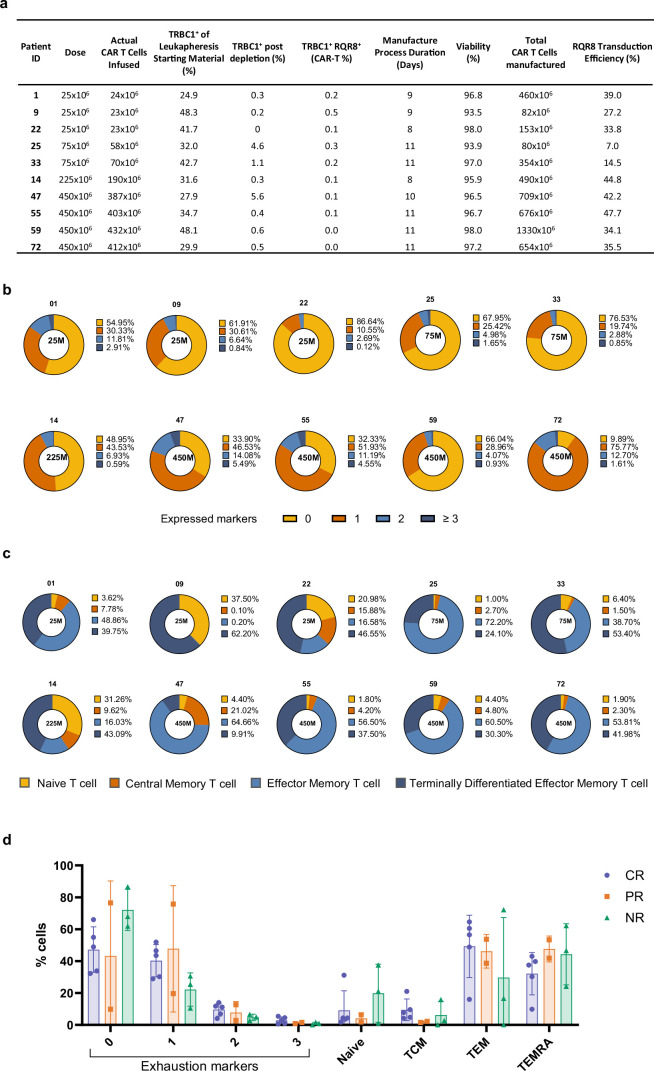
Drug product characterization. **a**) CAR T cell product characteristics. **b**) Flow cytometric analysis of cryopreserved CAR-T cell drug products for patients treated using a panel to determine product exhaustion. Markers included were PD1, TM3, LAG3 and TIGIT. ‘0’ denotes cells not expressing any of the markers; ‘1’ includes cells expressing a single marker; ‘2’ includes cells expressing any two markers in combination; ‘3’ includes any three in combination or all four markers. Gating strategy in Supplementary Information. **c**) Flow cytometric analysis of cryopreserved CAR-T cell drug products for patients treated. Immunophenotyping panel included CD45RA and CCR7. CCR7^+^ CD45RA^+^ were considered naïve cells; CCR7^+^ CD45RA^-^ T cells were considered central memory (TCM); CCR7^-^ CD45RA^-^ were considered Effector memory cells (TEM) and CCR7^-^ CD45RA^+^ cells were considered Terminally differentiated effector memory T cell (TEMRA). Gating strategy in Supplementary Information. **d**) Exhaustion marker expression and CAR T cell differentiation grouped by clinical response. CR = complete response, PR = partial response, NR = non-responder. CR patients 01, 14, 47, 55 and 59. PR patients 33 and 72, NR patients 09, 22 and 25. Mean ± SD. Source data

Infused patient demographics and disease features are summarized in Table 1. Median patient age was 55 years (range 34 to 63 years) and 80% had stage III/IV disease. Five patients (50%) had relapsed disease, five patients had primary refractory disease (50%) and five patients had disease (50%) refractory to the last line of therapy. Bridging therapy was administered to eight of ten patients, and all patients except for one ALCL patient who achieved CR upon brentuximab bridging (patient 14, cohort 3) had FDG-avid measurable PET-positive disease before Flu/Cy lymphodepletion and AUTO4 infusion. Of the infused patients, five were diagnosed with PTCL-NOS, four with AITL and one with anaplastic lymphoma kinase-negative ALCL. Patients received a median of two prior lines of therapy (range 1–5), including autologous stem cell transplantation (ASCT) in three patients (30%). Bridging therapy was administered in 80% of patients (Extended Data Table 2). AUTO4 drug product characteristics are summarized in Extended Data Fig. 2a–c. No correlation was determined between exhaustion marker expression or CAR T cell differentiation and clinical outcome (Extended Data Fig. 2d).

**Table 1:** Patient characteristics

Patient ID	Cohort dose	Histologic subtype	Age (years)	Sex	No. of prior lines	Prior ASCT	Bridging
22	25 × 10^6^	PTCL-NOS	34	Female	5	N	Y
01	25 × 10^6^	AITL	57	Male	2	N	Y
09	25 × 10^6^	AITL	61	Female	2	Y	N
33	75 × 10^6^	PTCL-NOS	35	Female	1	N	Y
25	75 × 10^6^	PTCL-NOS	53	Male	4	N	Y
14	225 × 10^6^	ALCL	47	Male	3	Y	Y
72	450 × 10^6^	PTCL-NOS	44	Male	2	Y	Y
55	450 × 10^6^	AITL	63	Male	3	N	Y
59	450 × 10^6^	PTCL-NOS	58	Male	3	N	N
47	450 × 10^6^	AITL	61	Male	2	N	Y

### Safety of AUTO4 administration

**Extended Data Fig. 3:**
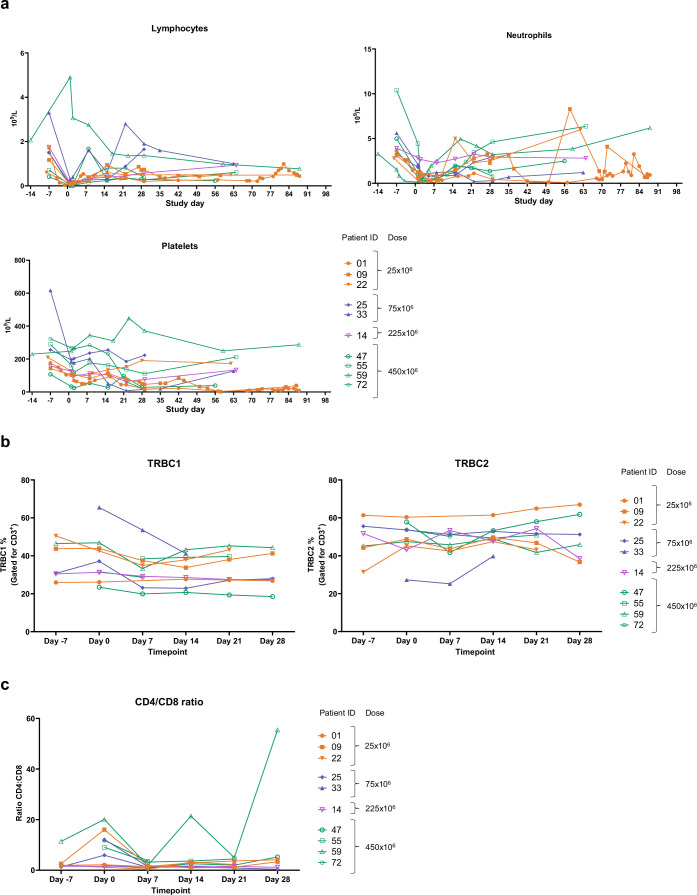
Peripheral Blood Counts. **a**) Peripheral blood counts for first 3 months are shown: Lymphocytes, Neutrophils and Platelets. **b**) TRBC1 and TRBC2 % in peripheral blood determined by flow cytometry. **c**) CD4:CD8 ratio in peripheral blood determined by flow cytometry. Source data

**Extended Data Fig. 4:**
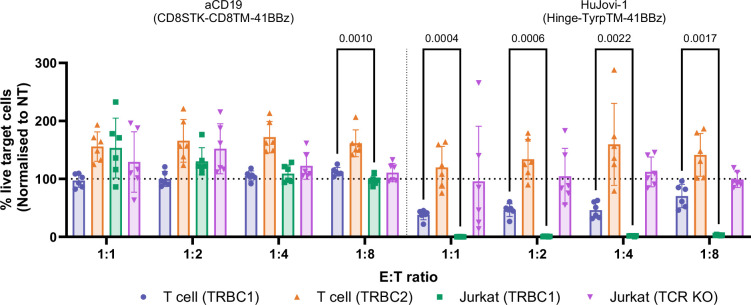
In vitro cytotoxicity. *In vitro* cytotoxicity assay on Jurkat TRBC1 and TCR KO cell lines, and TRBC1^+^ and TRBC2^+^ healthy human T cells, with HuJovi1-hinge-TyrpTM-41BBz CAR (AUTO4) and control anti-CD19 CAR (CD8stk-CD8TM-41BBz). E:T ratios 1:1, 1:2, 1:4 and 1:8, 72h, n=6 (individual healthy PBMC donors). Mean ± SD. Two-way ANOVA with Tukey’s post-test for T cell (TRBC1) vs Jurkat (TRBC1). Exact P value in graph. Source data

Treatment with Flu/Cy and AUTO4 was generally well tolerated (Table 2 and Extended Data Table 3). Any-grade cytokine release syndrome (CRS) was observed in four of ten patients (all dosed at 450 × 10^6^) at a median onset of 1 day (range, 1–5), lasting a median duration of 2 days (range, 2–10). In three of four patients, CRS was grade 1–2. One patient experienced grade 3 CRS on day 8, which resolved within 3 days. Tocilizumab was given to two patients; no steroids were administered in any patient to treat CRS. Importantly, no immune cell-associated neurotoxicity syndrome (ICANS) of any grade or dose-limiting toxicity (DLT) were observed in any patient (Table 2). The most common additional treatment-related adverse events irrespective of causality were transient neutropenia (grade 3 in 40%), thrombocytopenia (≥ grade 3 in 20%), anemia (≥ grade 3 in 40%) and lymphopenia, consistent with effects expected from lymphodepletion chemotherapy (Extended Data Table 3). The lymphopenia was observed after Flu/Cy lymphodepletion and AUTO4 infusion generally recovered to baseline within 3 weeks (Extended Data Fig. 3a). Unexpectedly, no alteration of the peripheral blood (PB) TRBC1:TRBC2 ratio was seen, suggesting lack of CAR T cell-mediated depletion of normal TRBC1^+^ T cells (Extended Data Fig. 3b,c). Notably, AUTO4 CAR T cells can deplete healthy TRBC1^+^ T cells in vitro, albeit less than T cell lymphoma cell lines (Extended Data Fig. 4).

**Table 2:** Summary of adverse events

	25 × 10^6^ (*n* = 3)	75 × 10^6^ (*n* = 2)	225 × 10^6^ (*n* = 1)	450 × 10^6^ (*n* = 4)	Total (*n* = 10)
DLT	0	0	0	0	0
Any-grade neutropenia	3 (100%)	2 (100%)	0	3 (75%)	8 (80%)
Any-grade infections	3 (100%)	1 (50%)	1 (50%)	1 (25%)	6 (60%)
Any-grade CRS	0	0	0	4 (100%)	4 (40%)
Grade 3 CRS	0	0	0	1 (25%)	1 (10%)
Any-grade ICANS	0	0	0	0	0

**Extended Data Fig. 5:**
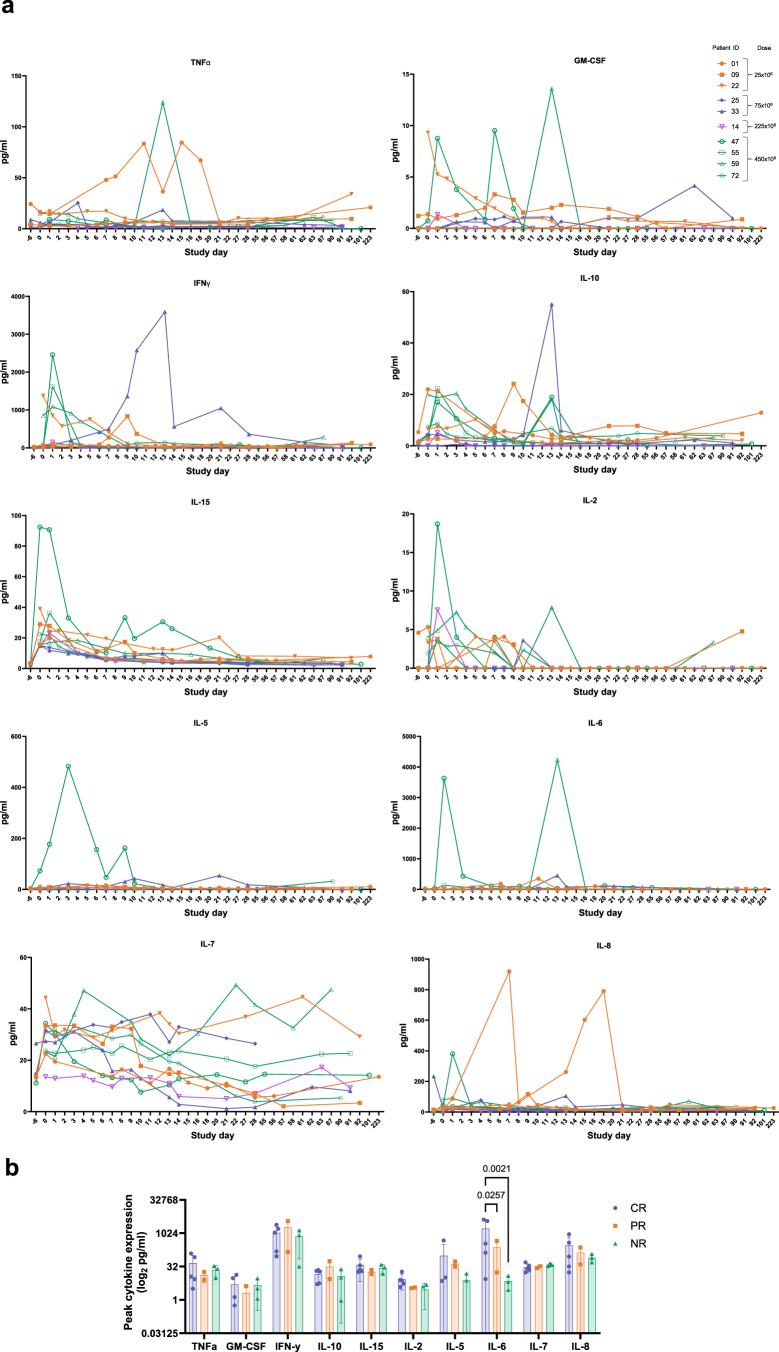
Serum cytokines. **a**) Cytokine levels measured in peripheral blood for TNF, GM-CSF, IFNγ, IL-2, IL-5, IL-6, IL-7, IL-8, IL-10 and IL-15. **b**) Peak serum cytokine concentrations grouped by clinical response. Mean ± SD. Two-way ANOVA with Tukey’s post-test. Exact P value in graph. CR = complete response, PR = partial response, NR = non-responder. CR patients 01, 14, 47, 55 and 59. PR patients 33 and 72, NR patients 09, 22 and 25. Source data

Serum cytokine levels were low across the study (Extended Data Fig. 5a). This is consistent with the low severity of CRS seen. Notably, a significant correlation was found between peak interleukin (IL)-6 serum concentrations and clinical response (Extended Data Fig. 5b).

Two patients experienced a rise in Epstein–Barr virus (EBV) genomic DNA copy number. One patient (dosed at 25 × 10^6^) developed grade 2 EBV reactivation on study day 27 in the context of immune-mediated thrombocytopenia. Rituximab was administered (once per week) on day 78 and the event resolved on day 93. Another patient (dosed at 75 × 10^6^) developed grade 1 EBV infection on day 29 after AUTO4 infusion, which was still DNA positive at time of data cutoff. No treatment was administered in this patient.

### Disease response following AUTO4 infusion

**Fig. 2:**
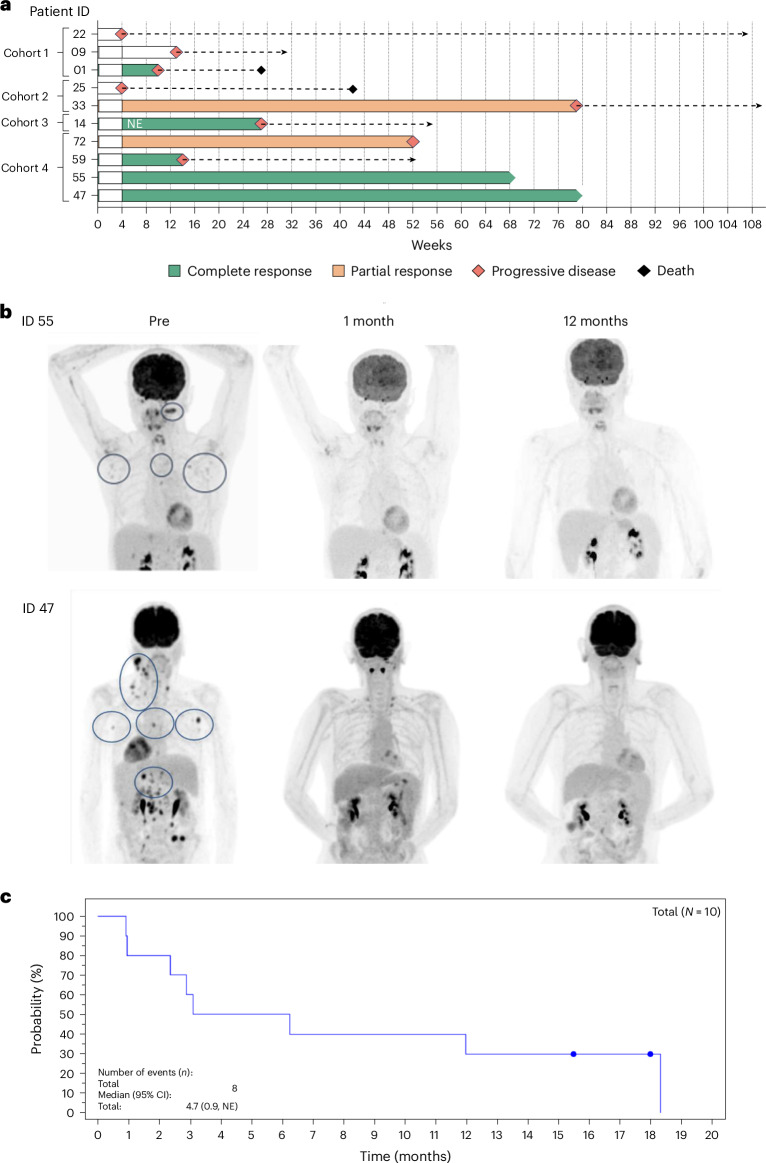
Clinical trial cohort. **a**, Swimmer plot showing the outcome in patients who received AUTO4. Note, one patient (14) who received 225 × 10^6^ AUTO4 CAR T cells was in CMR following bridging at time of infusion and is marked NE. Cohort 1, 25 × 10^6^; cohort 2, 75 × 10^6^; cohort 3, 225 × 10^6^; cohort 4, 450 × 10^6^. **b**, ^18^FDG PET-CT imaging before AUTO4, at month 1 and at 12 months from two participants (47 and 55) in long-term CMR following AUTO4. **c**, PFS based on overall response (Lugano classification). Median with 95% CI calculated from PROC LIFETEST output. Time relative to first AUTO4 treatment. 1 month = 30.4375 days. Source data

**Extended Data Fig. 6:**
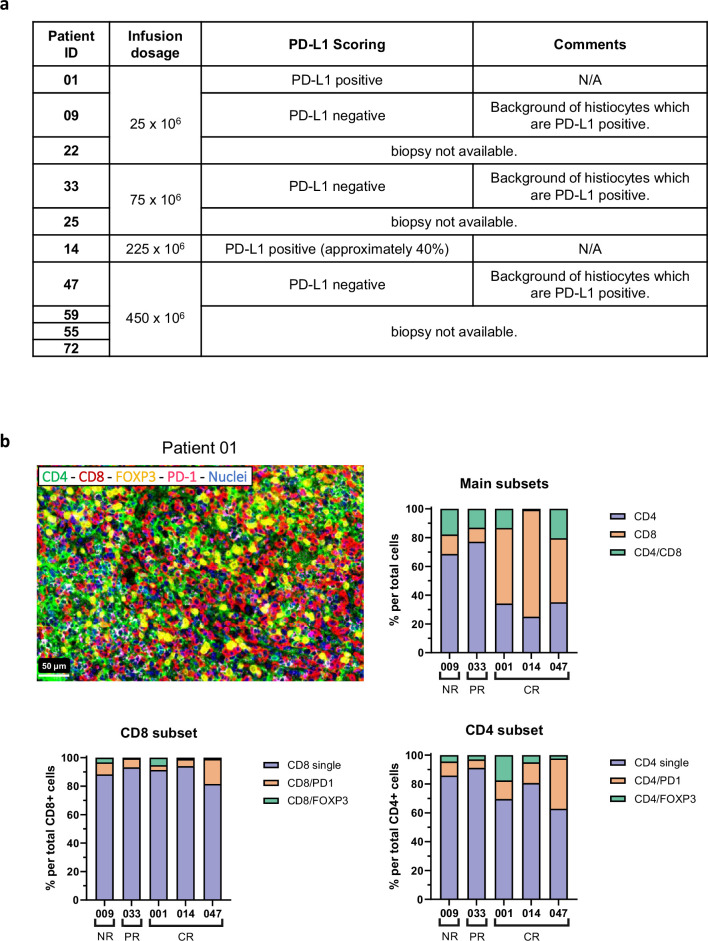
Screening biopsies. **a**) Tumour PD-L1 expression score on patients screening biopsies obtained via IHC. One tissue section stained per patient. **b**) Representative multiplex immunofluorescent staining of screening biopsy targeting CD4 (green), CD8 (red) FOXP3 (yellow) PD-1 (magenta) and nuclei (blue). Top right, quantification of main T cell subsets (CD4^+^, CD8^+^ and dual CD4^+^/CD8^+^) in patients, grouped by clinical response. Bottom left, quantification of CD8 subsets (CD8^+^, CD8^+^/PD1^+^, CD8^+^/FOXP3^+^) in patients, grouped by clinical response. Bottom right, quantification of CD4 subsets (CD4^+^, CD4^+^/PD1^+^, CD4^+^/FOXP3^+^) in patients, grouped by clinical response. CR = complete response, PR = partial response, NR = non-responder. One tissue section stained per patient. Source data

Median follow-up was 13.8 months at data cutoff (28 April 2023). One patient who received 225 × 10^6^ CAR T cells achieved complete metabolic response (CMR) by PET-CT after bridging therapy, so response at month 1 was evaluable in only nine of ten patients. Individual patient outcomes are illustrated in Fig. 1a. The best overall response rate (complete response (CR) + partial response (PR)) at any time after AUTO4 infusion by PET-CT among all response-evaluable patients was 66.6% (six of nine patients). CMR was observed in four of six responding patients and two patients achieved PR. At the highest dose level (450 × 10^6^ CAR T cells), all four patients achieved an objective response with three patients achieving CMR and one patient achieving a PR by PET-CT. Among patients in CMR at month 1, one patient was dosed at the 25 × 10^6^ dose level (relapsed at month 2 and did not receive further treatments until death due to underlying disease at study day 190), and three patients were dosed at the highest dose level tested (450 × 10^6^). Two of these three patients are in ongoing remission at 15 and 18 months, respectively, having received no further anti-lymphoma therapy (Fig. 2b). Interestingly, both patients had AITL. Of note, one of these two patients had disease that was refractory to all three prior lines of therapy (Extended Data Table 2). The third patient at the highest dose level tested relapsed at month 3 after sustaining a CMR at day 28. Two infused patients (nos. 72 and 33) who had achieved a PR did not require any further therapy with follow-up ≥ 12 months, but then showed disease progression at 12 and 18 months, respectively. Three patients had no response. No definite correlation was found between clinical response and pretreatment healthy T cell infiltrates or tumor programmed death-ligand 1 (PD-L1) expression (Extended Data Fig. 6). The median duration of remission in all responding patients across all cohorts was 5.3 months (95% confidence interval (CI) 1.4, NE). Among all infused patients, median PFS was 4.7 months (95% CI 0.9, non-evaluable (NE)) and median OS was not reached, with 90% (95% CI 47.3, 98.5) and 78.8% (95% CI 38.1, 94.3) projected to at months 9 and 18, respectively (PFS plot in Fig. 2c). Among all infused patients, two patients died due to the underlying disease at study day 190 and 294 and eight of ten are alive at the last follow-up.

### AUTO4 expansion and persistence

**Extended Data Fig. 7:**
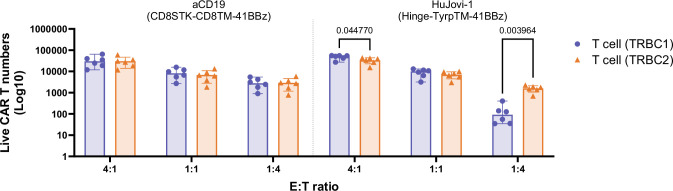
In vitro reverse killing. Reverse killing assay for healthy human T cell (TRBC1^+^ or TRBC2^+^) with HuJovi1-hinge-TyrpTM-41BBz CAR (AUTO4) and control anti-CD19 CAR (CD8stk-CD8TM-41BBz). E:T ratios 4:1, 1:1 and 1:4, 72h, n=6 (individual healthy PBMC donors). Mean ± SD. Two tailed multiple paired t test (Holm-Sidak method). Median (95% CI). Exact P value in graph. Source data

AUTO4 CAR T cell expansion and persistence were assessed in all infused patients in PB by flow cytometry and digital droplet polymerase chain reaction (ddPCR), and in lymph nodes by ddPCR and immunofluorescence (IF). At the 25 × 10^6^, 75 × 10^6^ and 225 × 10^6^ dose levels, no expansion was detected in PB. At the highest dose level of 450 × 10^6^, one patient had detectable transgene in PB with 1,476 vector copy number (VCN) per μg DNA in PB 10 min after infusion. The value dropped to 156 VCN per μg DNA by day 7 and 76 VCN per μg DNA by day 13. Day 123 was the latest time point in which CAR T cells were detectable by ddPCR (70 VCN per μg DNA; limit of quantification of 20 copies per μg DNA). No CAR T cells were detected by flow cytometry. Notably, some evidence for reverse targeting of AUTO4 CAR T cells by normal TRBC1^+^ T cells could be detected in vitro (Extended Data Fig. 7).

**Fig. 3:**
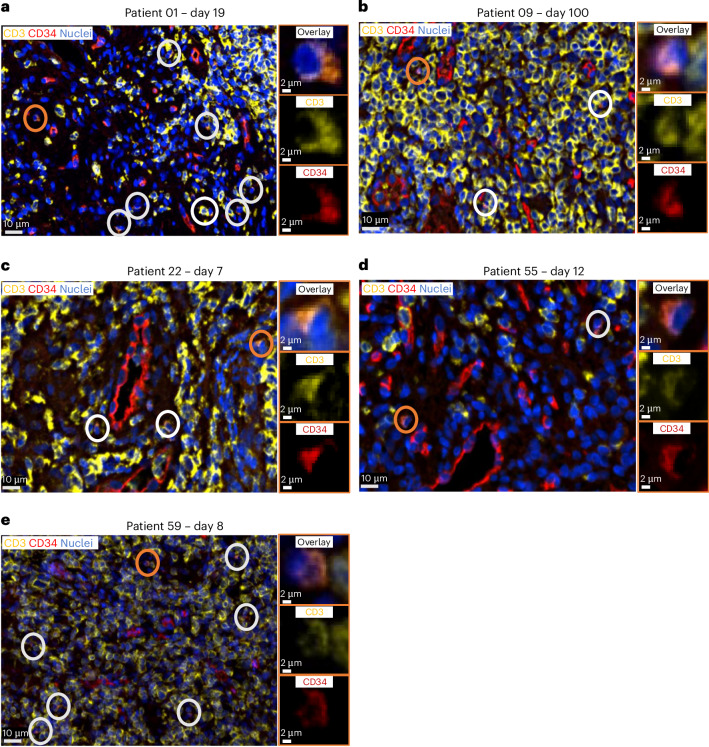
Lymph node biopsy. **a**–**e**, Lymph node biopsy samples after AUTO4 infusion for patient ID 01 (day 19) (**a**), 09 (day 100) (**b**), 22 (day 7) (**c**), 55 (day 12) (**d**) and 59 (day 8) (**e**). The main images show formalin-fixed, paraffin-embedded (FFPE) tissue sections of a T cell lymphoma stained by double IF with anti-CD34 (Q/BenD10), which stains RQR8 (red), and CD3 (yellow), which detects T cells. DAPI (blue) is used for nuclear counterstaining. CAR T cells (orange) are identified by coexpression of both Q/BenD10 and anti-CD3. The magnifications of the orange circled cells show overlay (top), CD3 staining (center) and CD34 staining (bottom). One tissue section stained per patient.

The LibraT1 study included on-treatment biopsy samples of sites of disease after AUTO4 administration when feasible. In contrast to findings in PB, in all five of five patients with posttreatment lymph nodes accessible to biopsy and suitable for testing (median 11 days after CAR T, range 7–279 days), both IF and ddPCR confirmed infiltration of AUTO4 CAR T cells at these tumor sites. Lymph node ddPCR AUTO4 copy number ranged from 111 to 171,700 VCN per μg DNA. IF biopsy imaging is shown in Fig. 3. PCR quantification of integrant copy number and IF counting is shown in Table 3.

**Table 3:** CAR T detection in lymph nodes by PCR and IF

Infusion dosage	Patient ID	Time point (after infusion)	VCN/µg DNA	CAR per total CD3^+^ cells (IF) (%)
25 × 10^6^	09	Day 100	520.15	2.71
	22	Day 7	4,141	1.20
	01	Day 19	19,695	4.41
75 × 10^6^	33	Biopsy not done		
	25	Day 28	Tissue exhausted	
225 × 10^6^	14	Biopsy not done		
450 × 10^6^	47	Biopsy not done		
	59	Day 8	2,525	4.06
	55	Day 12	10,302	1.39
	72	Day 62	Low-quality sample	

## Discussion

PTCL is an area of unmet clinical need. CHOP or CHOEP (cyclophosphamide, doxorubicin, vincristine, etoposide and prednisone) followed by consideration of consolidating ASCT for chemosensitive disease is commonly used as initial treatment. However, more than half of patients have either refractory or relapsing disease following initial treatment^9^. There is no standard of care for patients with r/r PTCL, and overall outcome remains poor^10^. In a retrospective analysis of 153 patients with r/r PTCL, median PFS was 3.1 months and OS was only 5.5 months^11^. The largest prospective study in PTCL, the international T cell project, analyzed the clinical outcomes of 633 patients with r/r PTCL and reported a median OS of 5 months for refractory disease and 11 months for relapsed disease^2^.

Few studies to date have reported on use of CAR T in PTCL. Anti-CD7 CAR T have been tested in T cell acute lymphoblastic leukemia, with high response rates. CD7 is a pan-T cell antigen also expressed on natural killer cells, but expression is frequently lost in PTCL with only approximately 25% of tumors expressing CD7 (ref. ^5^). Early data with CAR T cells targeting CD5, another pan-T antigen that is expressed on 20–90% of cases of PTCL^5,12^, have been reported in PTCL. Two of ten patients achieved a transient CR, with short CAR T persistence noted^13^; this study continues with a revised manufacturing process. A CAR T cell study targeting CD4 was stopped^14^. These pan-T cell targets are limited by expression on healthy T cells resulting in CAR T cell fratricide and immunosuppression caused by depletion of normal T cells. In addition, T cell lymphomas frequently have aberrant downregulation of one or more of these markers^5^. Other targets such as CD30, CD37 or CD70, which have none or limited expression on normal T cells can be used; however, these are only expressed on a small proportion of PTCL cases^15^.

The CD3–TCRαβ complex is a pan-T cell target, expressed on almost all cases of PTCL of αβ T cell origin^5^. We recently proposed a targeting strategy that exploits a gene duplication at the TCR β-chain constant locus and could avoid fratricide and prevent profound immunosuppression. In the human genome, two loci encode the TCR β-chain constant regions TRBC1 and TRBC2. During TCR gene rearrangement, either TRBC1 or TRBC2 are selected, such that healthy T cells are a mixture of TRBC1/TRBC2 TCR, while a PTCL, being clonal, expresses either one or the other. While only a two-amino-acid inversion is the targetable difference between TRBC1 and TRBC2, we previously demonstrated that the antibody Jovi-1 was highly selective for TRBC1 (ref. ^6^). In LibraT1, this present study, we have explored the preliminary safety and efficacy CAR T cell product specifically targeting TRBC1^+^ tumors in r/r PTCL using AUTO4, an autologous CAR T cell therapy based on Jovi-1.

AUTO4 was safe with minimal toxicity observed. Any-grade CRS was observed in four of ten patients (all at 450 × 10^6^). One patient (450 × 10^6^ cohort) developed grade 3 CRS, which resolved within 3 days. Importantly, no ICANS of any grade or DLT was seen. This lack of immunotoxicity correlated with low levels of serum cytokines. Two cases of EBV reactivation were observed. However, given the late timing of these reactivations and lack of CAR T cell persistence and lymphopenia at the time, these are unlikely to have been caused by AUTO4 CAR T cells.

Notably, AUTO4 CAR T cells were not readily detected in PB but were detected at high levels in all post-treatment biopsy samples, including a patient treated with the lowest dose of 25 × 10^6^ cells. In addition, surprisingly, selective depletion of normal TRBC1^+^ T cells did not occur. The mechanism for the absence of CAR T expansion and TRBC1 depletion is not fully understood. Normal T cell depletion has been observed in CAR T cell studies targeting CD7, suggesting a lack of intrinsic resistance to CAR T cell-based killing^16^, although, similarly to previous literature reports^17^, differential sensitivity of malignant and healthy T cells to TCR targeting was also detected for the AUTO4 CAR T cells. Further, normal TRBC1^+^ T cells can be engaged by AUTO4 CAR T cells potentially leading to ‘reverse killing’^18^, with relative paucity of normal T cells within diseased lymph nodes permitting nodal engraftment. Alternatively, the AUTO4 manufacturing process may have contributed to poor engraftment. Notably, AUTO4 CAR T cells predominantly displayed an effector memory or terminally differentiated immunophenotype, potentially reflecting the 10-day expansion protocol used in the study.

In diffuse large B cell lymphoma, a high area under the curve for CAR T cell detection over the first 28 days correlates with positive outcome in third-line^19^, but not second-line studies^4^. There is insufficient data to extrapolate to AUTO4 from these data and given the high response rates at a higher dose level, the clinical benefit of circulating AUTO4 cells is unclear and is best determined by ongoing clinical exploration. Circulating engraftment might be improved by administration of more intensive lymphodepletion and improving manufacture by, for example, shortening the process, and/or adding dasatinib to the AUTO4 cells during the manufacture expansion phase^20^. Admittedly, if continuing high response rates in the absence of TRBC1 depletion are observed, it could be argued that the complex paradigm of TRBC1/TRBC2 targeting may not be needed.

The preliminary efficacy and the ongoing responses reported in this phase 1 study are encouraging, with most responses observed at the highest AUTO4 dose: three of four patients who received the 450 × 10^6^ CAR T cells (highest dose) achieved CMR at month 1 and two of them remain in CMR beyond 18 months, suggesting a potential for AUTO4 CAR T to induce long-lasting remissions in a proportion of patients. Two patients with partial response are alive beyond 12 months without receiving any new anticancer medication. Furthermore, a possible impact on survival was seen, with eight of ten patients alive at last follow-up and a median duration of OS of 13.8 months, compared to a historical average of <6 months in this patient cohort^2^. Interpretation of efficacy from LibraT1 must, however, be interpreted with caution given this is a small, single-arm study.

Overall, our preliminary data support further development of AUTO4 CAR T cell therapy. The study is ongoing, with additional patients due to be treated to define the recommended phase 2 dose using an improved manufacturing process.

## Methods

### TRBC1 screening

TRBC1 status could be determined either by IHC or using the commercially available CE-marked in vitro diagnostic LymphoTrack Dx TRB Assay (Invivoscribe).

Assessment of TRBC1 expression on malignant cells by IHC was performed on serial sections (5-µm thickness) of fresh frozen lymph node tissue biopsy samples collected for screening. Single staining was performed for the following antibody markers: TRBC1 (JOVI-1 murine IgG22 monoclonal antibody clone, GeneTex), TCR VẞF1 (clone 8A3, GeneTex) and Ki-67 (M1B-1, Leica Biosystems). TCR Vẞ F1 staining enables identification of T cells, while Ki-67 allows for the distinction between healthy and proliferating malignant T cells. Staining was performed using the OptiViewDAB IHC detection kit (Ventana) on the BenchMark ULTRA DISCOVERY automated platform (Ventana). The tumor was considered TRBC1 clonal if ≥40% of viable tumors cells exhibit membrane staining at any intensity (≥1+) as reviewed by an expert hematopathologist.

The LymphoTrack Dx TRB Assay was performed at the Laboratory of Personalized Molecular Medicine, Invivoscribe (Hallbergmoos) according to manufacturer’s instructions to enable prospective patient selection. Briefly, DNA was extracted from 6 to 15 curls (5-μm thickness) from FFPE blocks and PCR amplified using 24 Illumina indexed master mixes with proprietary primer sets within Vβ and Jβ regions, before NGS using a MiSeq instrument. The sequencing data were analyzed using the LymphoTrack Dx Software-MiSeq package (Invivoscribe). The Merged Read Summary Report was used to identify the top merged read sequences and their frequencies to assess clonality. Evidence of clonality was determined if the top merged read represented ≥2.5% (if ≥20,000 total reads) or ≥5% (if ≥10,000 or <20,000 reads) of the total reads, and if the top merged read was more than two times the percentage of the fifth most frequent merged sequence for a detected D-J rearrangement, or more than two times the percentage of the third most frequent merged sequence for a detected J rearrangement. Clonal incomplete V-J sequences, as opposed to D-J sequences, were not considered acceptable to determine eligibility. Usage of J1 or J2 in rearranged sequences was used to infer TRBC1 or TRBC2 association, respectively.

### Vector and vector manufacture

AUTO4 is an autologous CAR T cell product coexpressing a humanized second-generation CAR targeting TRBC1 and the RQR8 safety switch, achieved by transduction of TRBC2-positive cells with a single bi-cistronic γ-retroviral vector (Supplementary Fig. 1b). The TRBC1-CAR was constructed from a single-chain variable fragment derived from a humanized form of the JOVI-1 antibody^6^ fused to the CD8α stalk fused to the endodomains of 41BB and CD3ζ^8^. RQR8 is a fusion of two copies of a rituximab binding mimotope separated by a fragment of human CD34 (ref. ^7^), which allows selective depletion of transgenic T cells with the therapeutic monoclonal antibody rituximab in the event of unmanageable toxicity. In addition, RQR8 allows convenient tracking and selection of CAR T cells by staining with the anti-CD34 QBEnd10 monoclonal antibody. γ-retroviral vector was produced under Good Manufacturing Practice conditions by three-plasmid co-transfection of HEK293T cells and subsequent harvest and purification of the culture supernatant^21^. The viral vector was pseudotyped with the RD114 envelope.

### CAR T cell manufacture

CAR T cell production was performed on the Miltenyi CliniMACS Prodigy with autologous leukapheresate used as starting material. First, pheresate was incubated with biotinylated anti-TRBC1 (using JOVI-1 monoclonal antibody) for 10 min at 4 °C followed by a wash step and secondary labeling with an anti-biotin CliniMACS. TRBC1 depletion was then performed with the MACS column on the CliniMACS Prodigy. The TRBC1^+^ depleted cells were washed and resuspended in TexMACS with 3% HABS, cytokine stimulated and activated with TransAct per the manufacturer’s instructions. On day 2, cells were transduced with γ-retroviral vector facilitated with VectoFusin and using spinoculation at 400*g* for 2 h at 32 °C on the Prodigy. On day 3, the cells were washed and expanded up to day 10. Cells were then cryopreserved in one or more CryoMACS bag(s) and stored in a vapor-phase liquid nitrogen environment before administration.

### Flow cytometry leukapheresis and drug product characterization

Frozen drug product and leukapheresis characterization experiments included fluorescence minus multiple controls (FMX), peripheral blood mononuclear cells (PBMCs) and single-stained UltraComp eBeads Compensation Beads (Thermo Fisher Scientific) to determine gating thresholds and calculate compensation. Samples were rested overnight in TexMACS10 medium (Miltenyi Biotec) after thawing. Samples were stained with a Fixable viability dye (BD Horizon) and then blocked (Miltenyi Biotec). Phenotypic characterization was performed using antibodies for memory and exhaustion markers diluted in Brilliant Stain Buffer Plus (BD Horizon). Staining for CCR7 was carried out before surface staining at 37 °C for 15 min (BioLegend). Intracellular staining was done using the Transcription Factor Buffer Set (eBioscience) according to the manufacturer’s instructions. Transduced CAR cells were identified using CAR anti-idiotype antibody and secondary donkey anti-rabbit conjugated to PE (BioLegend). Samples were analyzed using FCS Express software (De Novo Software). The gating strategy and antibody panel are in the Supplementary Information.

### Study design

LibraT1 (NCT03590574) is an ongoing, multicenter, single-arm study of AUTO4 in r/r TRBC1-positive PTCL (see study protocol in the Supplementary Information). The study consists of two phases: phase 1 dose escalation and phase 2 dose expansion. Each patient goes through the following five steps:

1. Screening stage consisting of TRBC1 screening and eligibility inclusion/exclusion criteria assessment. Inclusion criteria are: male or female, aged ≥18 years; willing and able to give written, informed consent to be screened for TRBC1-positive T-NHL and to enter the main study; confirmed diagnosis of selected T-NHL including PTCL-NOS, or AITL or ALCL; confirmed TRBC1-positive tumor; relapsed or refractory disease and have had ≥1 prior lines of therapy; PET-positive measurable disease per Lugano classification; Eastern Cooperative Oncology Group Performance Status 0 or 1; adequate bone marrow function without the requirement for ongoing blood products and meets the following criteria: absolute neutrophil count ≥ 1.0 × 10^9^ per liter, absolute lymphocyte count ≥ 0.5 × 10^9^ per liter (at entry and before leukapheresis), hemoglobin ≥80 g l^−1^, platelets ≥ 75 × 10^9^ per liter; adequate renal, hepatic, pulmonary and cardiac function defined as: creatinine clearance (as estimated by Cockcroft Gault) ≥60 ml per min, serum alanine aminotransferase/aspartate aminotransferase ≤2.5 times the upper limit of normal, total bilirubin ≤25 μmol l^−1^ (1.5 mg dl^−1^), except in patients with Gilbert’s syndrome; left ventricular ejection fraction ≥ 50% by echocardiogram or multigated acquisition cardiac scan, unless the institutional lower limit of normal is lower; baseline oxygen saturation ≥92% on room air and ≤ grade 1 dyspnea; for females of childbearing potential (defined as <2 years after last menstruation or not surgically sterile), a negative serum or urine pregnancy test must be documented at screening, before preconditioning and confirmed before receiving the first dose of study treatment. For females who are not postmenopausal (<24 months of amenorrhea) or who are not surgically sterile (absence of ovaries and/or uterus), a highly effective method of contraception together with a barrier method must be used from the start of the preconditioning stage and for at least 12 months after the last dose of AUTO4 (study treatment). They must agree not to donate eggs (ova, oocytes) for the purposes of assisted reproduction during the study and for 12 months after receiving the last dose of study drug; for males, it must be agreed that two acceptable methods of contraception are used from the start of the preconditioning stage and for at least 12 months after the last dose of AUTO4 (one by the patient (usually a barrier method), and one by the patient’s partner). Also, that sperm will not be donated during the treatment period and for at least 12 months after the last dose of study treatment; no contraindications for leukapheresis or the preconditioning regimen.
  Exclusion criteria are: general exclusion criteria: patients with T cell leukemia; females who are pregnant or lactating; prior treatment with investigational gene therapy or approved gene therapy or genetically engineered cell therapy product or allogeneic stem cell transplant; known history or presence of clinically relevant CNS pathology such as epilepsy, paresis, aphasia, stroke within prior 3 months, severe brain injuries, dementia, Parkinson’s disease, cerebellar disease, organic brain syndrome, uncontrolled mental illness or psychosis. Patients with a known history or prior diagnosis of optic neuritis or other immunologic or inflammatory disease affecting the CNS; current or history of CNS involvement by malignancy; clinically significant, uncontrolled heart disease (New York Heart Association Class III or IV heart failure, uncontrolled angina, severe uncontrolled ventricular arrhythmias, sick-sinus syndrome or electrocardiographic evidence of acute ischemia or grade 3 conduction system abnormalities unless the patient has a pacemaker) or a recent (within 12 months) cardiac event; uncontrolled cardiac arrhythmia (patients with rate-controlled atrial fibrillation are not excluded); evidence of pericardial effusion; patients with evidence of uncontrolled hypertension or with a history of hypertension crisis or hypertensive encephalopathy; patients with a history (within 3 months) or evidence of deep vein thrombosis or pulmonary embolism requiring ongoing therapeutic anticoagulation at the time of preconditioning; patients with active gastrointestinal bleeding; patients with any major surgical intervention in the last 3 months; active infectious bacterial, viral or fungal disease (hepatitis B virus, hepatitis C virus, human immunodeficiency virus, human T cell lymphotropic virus or syphilis) requiring treatment; active autoimmune disease requiring immunosuppression; history of other neoplasms unless disease free for at least 2 years (adequately treated carcinoma in situ, curatively treated non-melanoma skin cancer, breast or prostate cancer on hormonal therapy are allowed); prior treatment with programmed cell death protein 1 (PD-1), PD-L1 or cytotoxic T lymphocyte-associated protein 4 targeted therapy (CTLA-4) or tumor necrosis factor (TNF) receptor superfamily agonists including CD134 (OX40), CD27, CD137 (41BB) and CD357 (glucocorticoid-induced TNF receptor family-related protein) within 6 weeks before AUTO4 infusion; the following medications are excluded: Steroids: Therapeutic doses of corticosteroids within 72 h of leukapheresis or preconditioning chemotherapy administration. However, physiological replacement, topical and inhaled steroids are permitted; cytotoxic chemotherapies within 2 weeks before leukapheresis or AUTO4 infusion; antibody therapy use within 2 weeks before AUTO4 infusion, or five half-lives of the respective antibody, whichever is longer; live vaccine within 4 weeks before enrollment; research participants receiving any other investigational agents, or concurrent biological, chemotherapy or radiation therapy; use of rituximab (or rituximab biosimilar) within the last 6 months before AUTO4 infusion; patients, who in the opinion of the investigator, may not be able to understand or comply with the safety monitoring requirements of the study. Exclusion criteria for preconditioning chemotherapy and AUTO4 infusion: Severe intercurrent infection at the time of preconditioning chemotherapy or the scheduled AUTO4 infusion; requirement for supplementary oxygen or active pulmonary infiltrates or significant deterioration of organ function at the time of preconditioning chemotherapy or scheduled AUTO4 infusion; significant clinical deterioration of organ functions from screening, as determined by the investigator.
2. Leukapheresis stage followed by AUTO4 manufacture;
3. Preconditioning stage consisting of lymphodepleting treatment with Flu (30 mg m^2^ on days −6 to −3) and Cy (500 mg m^2^ on days −6 and −5) (Flu/Cy) before AUTO4 infusion;
4. Treatment stage in which AUTO4 is administered intravenously as a single infusion on day 0;
5. Follow-up stage starting after AUTO4 administration up to 24 months after the infusion of the last patient with AUTO4 or at their disease progression or withdrawal of consent.

In phase 1 dose escalation, four dose levels were explored: cohort 1 (*n* = 3 patients) received 25 × 10^6^ AUTO4 T cells; cohort 2 (*n* = 2 patients) received 75 × 10^6^ AUTO4 T cells; cohort 3 (*n* = 1 patient) received 225 × 10^6^ AUTO4 T cells; and cohort 4 (*n* = 4 patients) received 450 × 10^6^ AUTO4 T cells.

Patients were assigned sequentially to dose groups, with a rolling six design. For cohorts 2 and 3, if no CAR T cell expansion was detected in any of the patients treated (with at least one patient treated at that dose), together with no grade ≥ 1 CRS/neurotoxicity or ≥grade 2 AUTO4-related adverse events in the DLT period (first 28 days after AUTO4 infusion), accelerated escalation to the next level was allowed. If CAR T cell expansion was detected in the potential single patient cohorts (cohorts 2 and 3), the cohort must be expanded to a minimum of three patients treated. For cohort 4, the standard rolling six design was applied, with a minimum of three patients treated.

Primary endpoints in phase 1 are incidence of ≥grade 3 toxicity occurring within 60 days of AUTO4 infusion and the frequency of DLTs within 28 days of AUTO4 infusion. Overall response (CR + PR) rate by Lugano PET-CT criteria is a secondary endpoint (Extended Data Table 1).

### Toxicity assessment

1. Adverse events over the first 28 days after CAR infusion were graded according to Common Terminology Criteria for Adverse Events (version 5.0).
2. CRS and neurotoxicity were graded by the American Society for Transplantation and Cellular Therapy (ASTCT)/CTCAE v5.0 and American Society for Blood and Marrow Transplantation (ASBMT).
3. ICANS were graded according to grading criteria by ref. ^22^.
4. Hemophagocytic lymphohistiocytosis was graded as per ref. ^23^.

### Response assessment and translational analysis

Disease response assessments were performed at protocol-defined time points (pre-lymphodepleting chemotherapy (LD), months 1, 3, 6, 9, 12, 15, 18 and 24) by ^18^FDG PET-CT according to the Response Criteria for NHL-Lugano classification^24^. All participants had disease status evaluation within 4 weeks of initiation of LD. For those patients who received a bridging chemotherapy regimen, baseline PET-CT scans were done after bridging therapy and before LD and AUTO4 infusion.

### Whole-blood flow cytometry

The surface assay follows a lyse wash protocol, for each sample a fluorescence-minus-one control was generated to determine CAR positivity. The compensation was set up using the Lyric software reference settings maintained daily by using the performance quality-control wizard. When creating reference settings, single-stained compensation beads (BD Horizon, BD Biosciences) were used. A volume of 100 µl of whole blood was used for staining. The sample was blocked (Miltenyi Biosciences) followed by staining with a cocktail of antibodies to identify the immune cell subsets, and the presence of transformed CARs.

To assess viability, a fixable viability dye (BD Horizon) was added to Lysis solution (BD Biosciences). The Live/Lysis solution was added to the blood and incubated. Cells were washed and resuspended in BD Stain buffer (BD Biosciences).

The sample was then transferred to a TruCount Tube (BD Biosciences) for acquisition and analysis on the BD FACSLyric (BD Biosciences).

The ratio of TRBC1/TRBC2 was determined using a surface assay following a two-step staining protocol using fluorescence-minus-one controls and secondary control for TRBC2. Frozen PBMCs were thawed, blocked using Human FcR Block (Miltenyi Biotec) and stained for viability using Fixable Viability Stain 700 (BD Biosciences). Cells were washed and resuspended in a surface stain master mix containing anti-CD45 PerCP-Cy 5.5 (332784Biol, BD), anti-CD3 BV510 (300448, BioLegend), anti-CD4 BV605 (344646, BioLegend), anti-CD8 PE-Cy7 (335822, BD), anti-CD19 BV786 (563325, BD), anti-TCR beta 1 AF488 (Santa Cruz Biotechnology), CD34 PE (FAB7227P, R&D systems) and anti-TRBC2 biotin (Autolus). Cells were washed and resuspended in a secondary only master mix containing Streptavidin antibody BV421 (405225, BioLegend). Cells were washed, resuspended and results acquired in BD FACSLyric (BD Biosciences). Data were analyzed using FCS Express (De Novo Software). The gating strategy is available in the Supplementary Information.

### CAR T cell persistence by ddPCR

DNA was extracted from whole-blood samples using the QIAamp DNA Mini Kit (Qiagen) according to the manufacturer’s instructions. Extracted DNA was diluted to a desired final concentration and used as a template in a ddPCR reaction. Each reaction contains HindIII-HF DNA digest along with primers and probes to detect both the target Psi packaging gene and the reference gene *RPP30* to determine vector copy numbers. Following reaction setup, in each well, the ddPCR reaction was partitioned into nanoliter-sized water-in-oil droplets using the QX200 Droplet Generator (Bio-Rad) and subsequently amplified by PCR. Positive and negative droplets were then quantified on the QX200 Droplet Reader Generator (Bio-Rad). Using the QuantaSoft software generator (Bio-Rad), Poisson statistics are applied to the data to determine the target copy number variation of Psi, and normalization against the two-copy *RPP30* values yields the VCN per cell.

### Lymph node FFPE ddPCR

DNA was extracted from fresh frozen FFPE lymph node tissue biopsy curls (3 × 10 µm) using the QIAamp DNA FFPE Tissue Extraction KIT (Qiagen), per the manufacturer’s instructions. Extracted DNA was diluted to the desired final concentration and used as a template for ddPCR reaction. For the ddPCR assay, please refer to ‘CAR T cell persistence by ddPCR’.

### Lymph node FFPE IHC and multiplexed IF

A four-color panel was developed to assess CAR T cell persistence in FFPE lymph node biopsy samples (*n* = 5) collected after AUTO4 product infusion. The panel includes the following antibodies: CD34 (QBend10, Leica Biosystems) for CAR T cell detection; CD3 (NCL-L-CD3565, Leica Biosystems) to detect T cells; Ki-67 (MIB-1, Leica Biosystems) to aid the distinction between healthy and proliferative malignant T cells; and spectral DAPI (Akoya Biosciences) for nuclei identification. An Opal 6-Plex Manual Detection Kit (Akoya Biosciences) was used to add fluorescent labels to primary antibodies. Staining was performed on the Leica Bond RX platform with the Bond Polymer refine detection kit (Leica Biosystems). Multispectral imaging was performed using the PhenoImager HT and exported from the inForm software (Akoya Biosciences). Multispectral whole-slide images were generated. Additionally, regions of interest were acquired at a ×20 magnification. Image cell segmentation was performed on QuPath on the DAPI channel with the StarDist package for automatic cell detection. CAR persistence was determined as the percentage of CAR T cells (CD3^+^CD34^+^) per total T cells (CD3^+^).

A five-color panel was developed to assess T cell infiltration in FFPE lymph node biopsy samples (*n* = 5) collected before AUTO4 product infusion. The panel includes the following antibodies: CD4 (CD4-368-L-CE, Leica Biosystems) for CD4^+^ T cell detection; CD8 (CD8-4B11-L-CE, Leica Biosystems) for CD8^+^ T cell detection; PD-1 (NAT105, Abcam) for detection of exhausted CD4^+^ and CD8^+^ T cells; FOXP3 (236A/E7, Abcam) for detection of regulatory T cells; and spectral DAPI (Akoya Biosciences) for nuclei identification. An Opal 6-Plex Manual Detection Kit (Akoya Biosciences) was used to add fluorescent labels to primary antibodies. Staining was performed on the Leica Bond RX platform with the Bond Polymer refine detection kit (Leica Biosystems). Multispectral imaging was performed using the PhenoImager HT and exported from the inForm software (Akoya biosciences). Multispectral whole-slide images were generated. Image cell segmentation was performed on QuPath on the DAPI channel with the StarDist package for automatic cell detection. The representative image overlay from patient 01 was acquired on QuPath. Cell counts were extracted from image cell segmentation and distribution percentages calculated for the following marker combinations: CD4^+^ single; CD4^+^PD-1^+^; CD4^+^FOXP3^+^; CD8^+^ single; CD8^+^PD-1^+^; CD8^+^FOXP3^+^; and CD4^+^CD8^+^. Distribution plots were generated with GraphPad Prism (v9.0).

PD-L1 assessment was performed by IHC in FFPE lymph node biopsy samples (*n* = 5) collected before AUTO4 product infusion. PD-L1 (SP263, Roche Diagnostics) staining was performed on the Leica Bond RX platform with the Bond Polymer refine detection kit (Leica Biosystems). PD-L1 scoring was performed by a senior hematopathologist.

### Serum cytokines

Two Meso Scale Discovery multiplex sandwich immunoassay panels (10-V Plex and Cytokine Human Panel 1), performed at Q2 Solutions (Edinburgh) according to manufacturer’s instructions, were used to determine serum cytokines (interferon-y, granulocyte–macrophage colony-stimulating factor, IL-2, IL-5, IL-6, IL-8, IL-10, IL-15, IL-7 and TNF) concentrations in serum samples.

### In vitro CAR T cytotoxicity

Leukocyte cones of healthy donors were purchased from National Health Service Blood and Transplant (NHSBT, United Kingdom), with consent for nonclinical use. Work was performed under approval of the Human Tissue Authority (HTA license 12642). Whole blood was extracted from each cone and diluted to 50 ml with sterile PBS. PBMCs were isolated by Ficoll gradient centrifugation using SepMate 50 (85450, StemCell) and Ficoll Paque Plus (GE17-1440-02, Merk) layering 25 ml of whole-blood mixture to each SepMate 50. The cells were centrifuged at 1,200*g* for 20 min. The buffy coat was extracted and washed twice with sterile PBS. PBMCs were resuspended at 2 × 10^7^ per ml in cell separation buffer (20144, StemCell) and incubated with 3 μg per 2 × 10^5^ cells of biotinylated Jovi-1 (ANC-101-030, Ancell) for 10 min. Samples were centrifuged at 400*g* for 5 min and then washed with separation buffer before following EasySep Release Human Biotin Positive Selection Kit (17653, StemCell) protocol. The unbound (TRBC2^+^) fraction was harvested from the first incubation on the magnetic rack. The bound (TRBC1^+^) fraction was collected by following the protocol as stated. Isolation was confirmed via flow cytometry, staining with aCD3-PE/Cy7 (317334, BioLegend) and streptavidin–APC (405243, BioLegend).

Isolated TRBC1^+^ or TRBC2^+^ T cells were resuspended at 1 × 10^6^ cells per ml in R10 and stimulated with TransAct (Miltenyi Biotec, 130-111-160), 10 ng ml^−1^ IL-7 (Miltenyi Biotec, 130-095-367) and 10 ng ml^−1^ IL-15 (Miltenyi Biotec, 130-095-760). Forty-eight hours after, cells were collected, plated at a density of 0.6 × 10^6^ cells per well (0.5 ml) on retronectin-coated (Takara, T100B) 24-well plates in the presence of retroviral supernatant at a multiplicity of infection of 0.5. Total volume was adjusted to 2.5 ml using R10 supplemented with 10 ng ml^−1^ IL-7 (Miltenyi Biotec, 130-095-367) and 10 ng ml^−1^ IL-15 (Miltenyi Biotec, 130-095-760). The plates were centrifuged at 1,000*g* for 40 min. Seventy-two hours after spinoculation, the T cells were harvested and replated in complete R10 media supplemented with 10 ng ml^−1^ IL-7 (Miltenyi Biotec, 130-095-367) and 10 ng ml^−1^ IL-15 (Miltenyi Biotec, 130-095- 760). Transduction efficiency was determined on day 3 after transduction, and further experiments were commenced on days 7–10 after transduction. CAR expression was assessed by staining with aCD3-PE/Cy7 (317334, BioLegend) and QBend10 APC (FAB7227A, R&D System).

For the standard cytotoxicity assay, mock (non-transduced PBMCs) and CAR-transduced T cells were co-cultured with TRBC1^+^ or TRBC2^+^ non-transduced T cells, TRBC1^+^, TRBC2^+^ or TCR knockout Jurkat target cells. Target cells were labeled with CellTrace CFSE (C34554, Thermo Fisher Scientific) following the manufacturer’s instructions. Mock and CAR-transduced T cells were labeled with CellTrace Violet (C34557, Thermo Fisher Scientific) following the manufacturer’s instructions. Effector and target cells were mixed to reach an effector-to-target cell ratio of 1:1, 1:2, 1:4 and 1:8. Seventy-two hours after co-culture, live cell data were collected via flow cytometry using the MacsQuantX flow cytometer (Milteny). Data analysis was conducted using FlowJo v10 (Treestar, RRID: SCR_008520). The number of target cells was quantified using CountBright Counting Beads (Thermo Fisher Scientific, C36995). The percentage of live cells was calculated relative to the number of live target cells after co-culture with non-transduced T cells.

For the reverse killing assay, mock and CAR-transduced T cells were co-cultured with autologous TRBC1^+^ and TRBC2^+^ non-transduced T cells. Target cells were labeled with CellTrace CFSE (C34554, Thermo Fisher Scientific) following the manufacturer’s instructions. Effector mock and CAR-transduced T cells were labeled with CellTrace Violet (C34557, Thermo Fisher Scientific) following the manufacturer’s instructions. Effector and target cells were mixed to reach an effector-to-target cell ratio of 1:4, 1:1 and 4:1. Seventy-two hours after co-culture, live cell data were collected via flow cytometry using the MacsQuantX flow cytometer (Miltenyi). Data analysis was conducted using FlowJo v10 (Treestar, RRID: SCR_008520). The number of CAR T cells was quantified using CountBright Counting Beads (Thermo Fisher Scientific, C36995).

### Statistical analysis

Clinical data are captured in the clinical database via the Encapsia electronic data capture system v1.0. SAS 9.4 was used for clinical data analysis. All data are summarized descriptively due to the phase 1 exploratory nature of the study. Categorical variables are reported in terms of frequency and percentage, and continuous variables in terms of median and range unless otherwise specified. Time-to-event outcomes were summarized using the Kaplan–Meier method. Toxicity events are reported at the maximum grade experienced according to the CTCAE. In vitro data were analyzed with GraphPad Prism v10.1.2 (GraphPad software).

### Inclusion and ethics statement

The study was approved in the United Kingdom by the UK Medicines and Healthcare Products Regulatory Agency (clinical trial authorization no. CTA46113/004/001-0011), the London/West London GTAC Research Ethics Committee (REC ref. no. 17/LO/1730) and the research and development departments of all participating National Health Service trusts. The study was approved in Spain by the Spanish Agency of Medicines and Medical Products under EudraCT number 2017-001965-26. The study was managed by Autolus. Written informed consent was obtained from patients before study entry in accordance with the Declaration of Helsinki. This report incorporates data from all participants who received AUTO4 on study. Data were locked as of 28 April 2023.

### Reporting summary

Further information on research design is available in the Nature Portfolio Reporting Summary linked to this article.

## Online content

Any methods, additional references, Nature Portfolio reporting summaries, source data, extended data, supplementary information, acknowledgements, peer review information; details of author contributions and competing interests; and statements of data and code availability are available at 10.1038/s41591-024-03326-7.

## Supplementary information

Supplementary Information Supplementary Figs 1 and 2, Supplementary Table 1 and Supplementary Note 1

Reporting Summary

## Source data

Source Data Fig. 2 and Extended Data Figs. 2–7 Statistical source data.

## Figures and Tables

**Extended Data Table 1:**
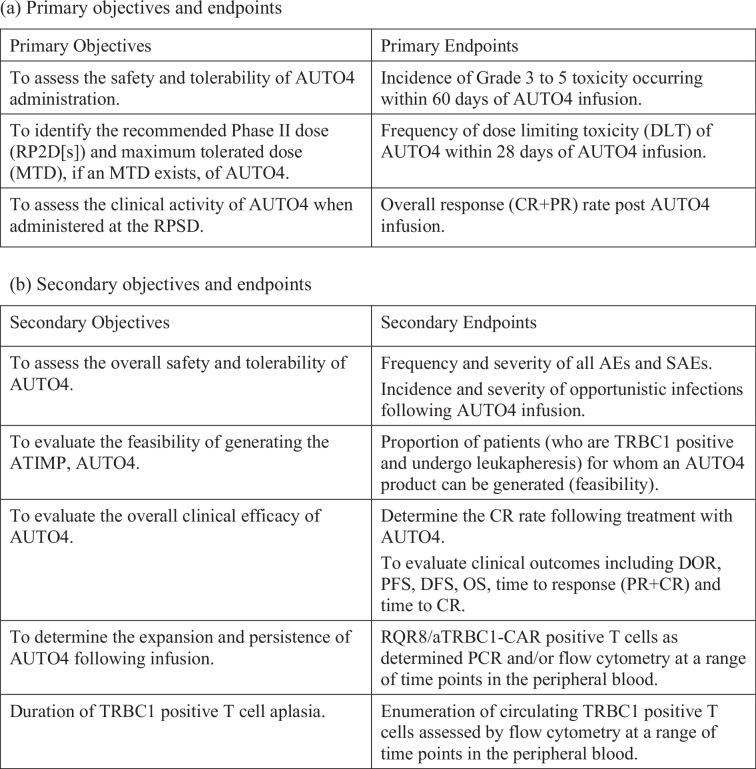
LibraT1 endpoints

(a) Primary objectives and endpoints

(b) Secondary objectives and endpoints

**Extended Data Table 2:**
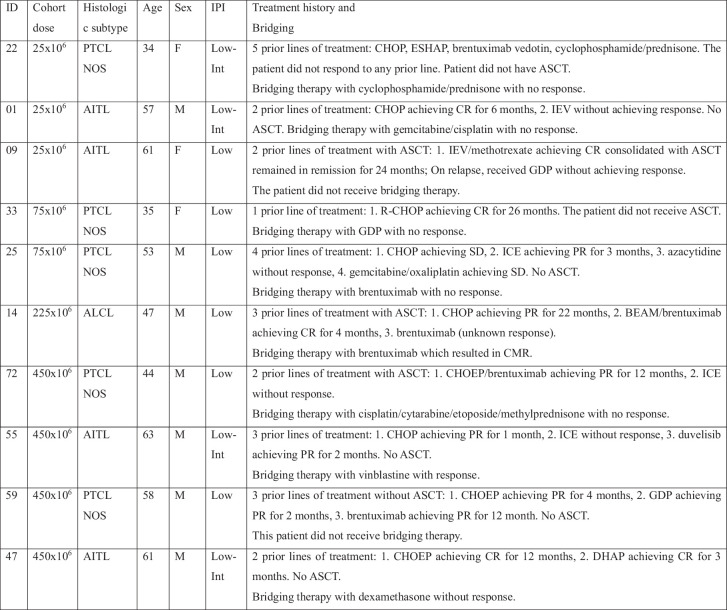
LibraT1 participants detailed treatment and bridging history

**Extended Data Table 3:**
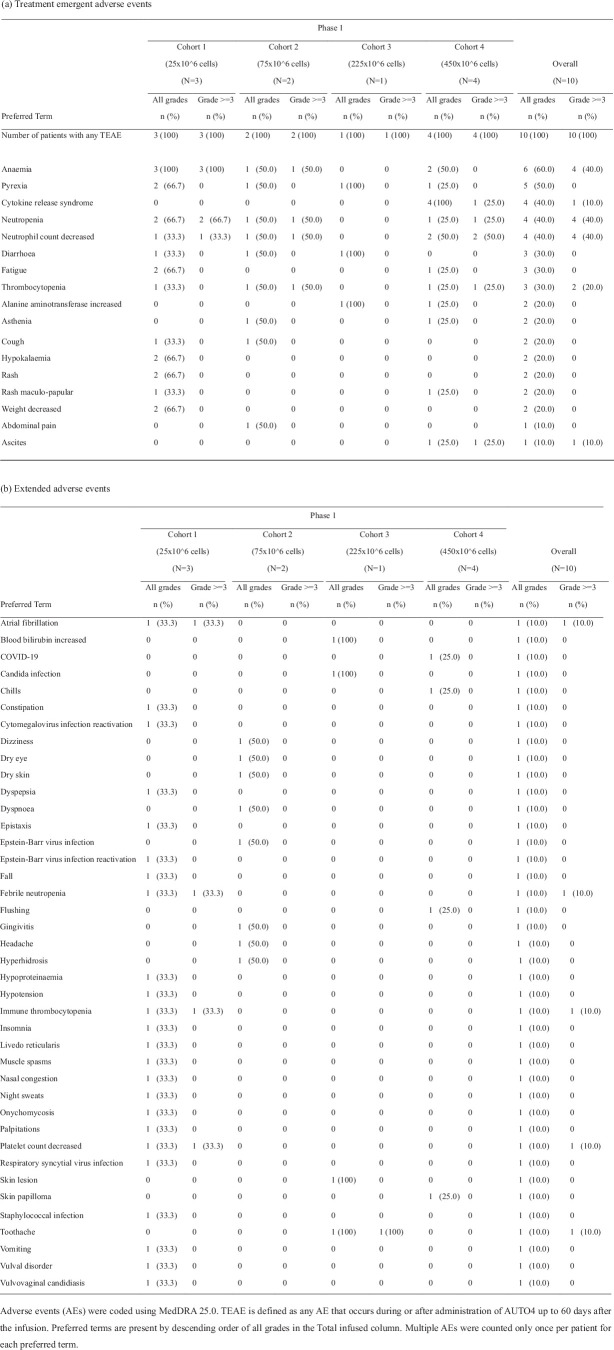
LibraT1 detailed adverse events

(a) Treatment emergent adverse events

(b) Extended adverse events

## Data Availability

Individual de-identified participant data are not disclosed. The study protocol is available in the Supplementary Information. The statistical analysis plan can be requested after clinical trial NCT03590574 completion. Researchers who provide an analysis proposal that complies with clinical study ethical and data integrity requirements can request the relevant information from M.P. Requests will be evaluated within 30 days. Source data are provided with this paper.
