# Supplementary material for: TRBC1-CAR T cell therapy in peripheral T cell lymphoma: a phase 1/2 trial
Source: Nat Med. 2024 Nov 11;31(1):137–43. doi: 10.1038/s41591-024-03326-7 (PMC11750712; doi:10.1038/s41591-024-03326-7)
Supplement: Supplementary file 1 — Supplementary Figs 1 and 2, Supplementary Table 1 and Supplementary Note 1 [file 41591_2024_3326_MOESM1_ESM.pdf]

nature medicine

Supplementary information https://doi.org/10.1038/s41591-024-03326-7

TRBC1-CAR T cell therapy in peripheral T cell lymphoma: a phase 1/2 trial

In the format provided by the authors and unedited

Supplementary Information

1) Whole blood flow cytometry gating strategy
2) Leukapheresis and drug product characterisation flow cytometry gating strategy
3) Antibody panel for flow cytometry leukapheresis and drug product characterisation
4) Study protocol

Supplementary Figure 1. Gating strategy for whole blood flow cytometry

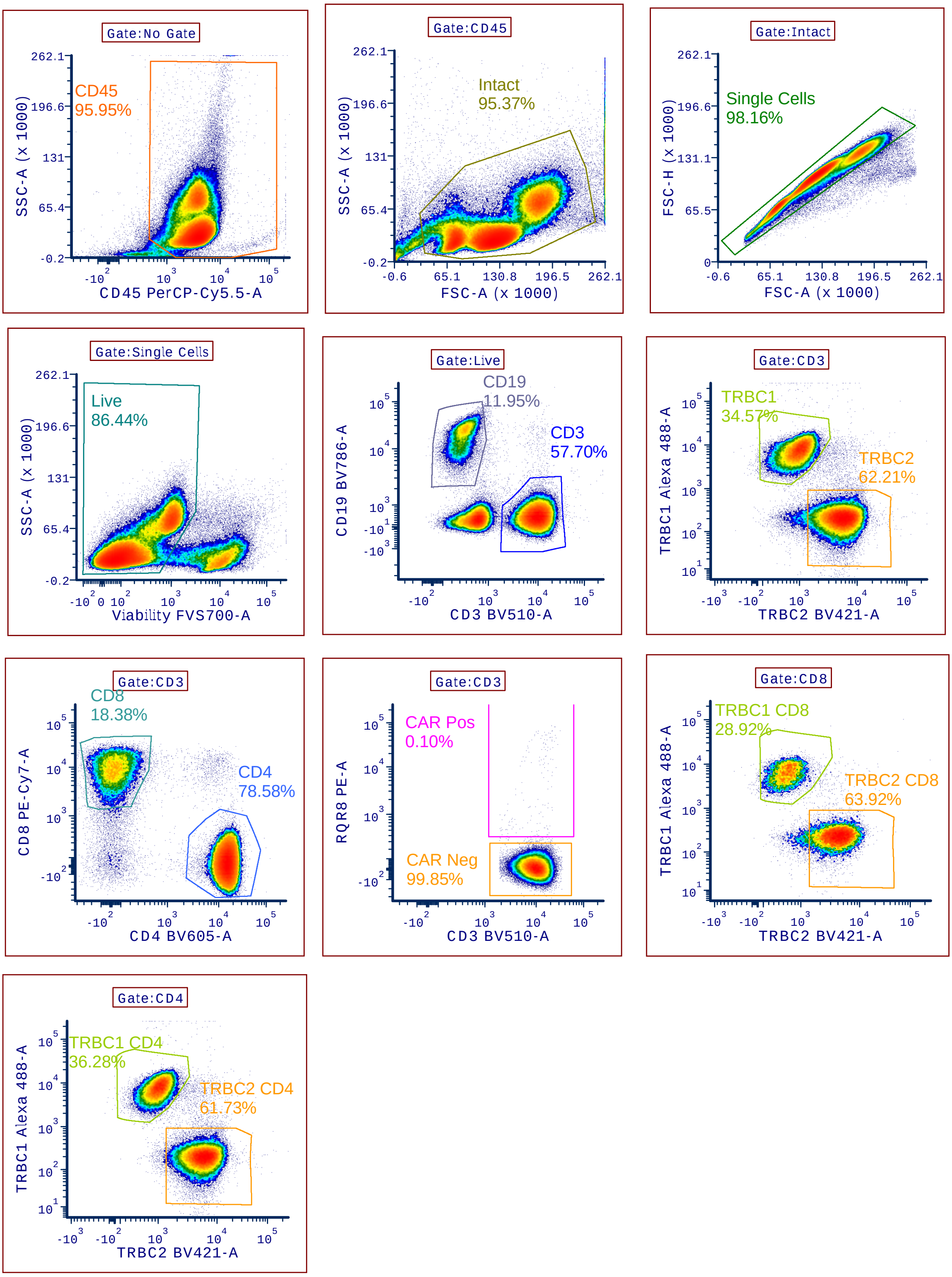

Gating Hierarchy

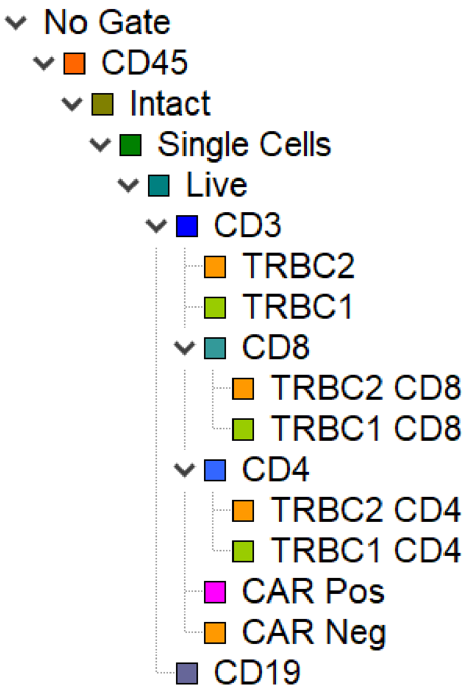

1) CD45 vs SSC-A to define leucocytes 2) Intact cells are gated on FSC-A vs SSC-A 3) FCS-A vs FCS-H is used to gate on single cells. 4) Live cells are gated using FVS-700 vs SSC-A. 5) CD3 vs CD19 is used to define lymphocytes 6) CD3 positive lymphocytes are dived by TRBC1 positive and TRBC2 positive populations 7) CD3 lymphocytes are subdivided into CD4 and CD8. 8) CD3 CAR positive population is defined by RQR8 positivity using CD3 vs RQR8 with the negative population labelled as CD3 CAR negative 9) CD4 positive cells are divided into TRBC1 positive and TRBC2 positive populations. 10) CD8 positive cells are divided into TRBC1 positive and TRBC2 positive populations.

Supplementary Figure 2. Gating strategy for AUTO4 drug product characterisation

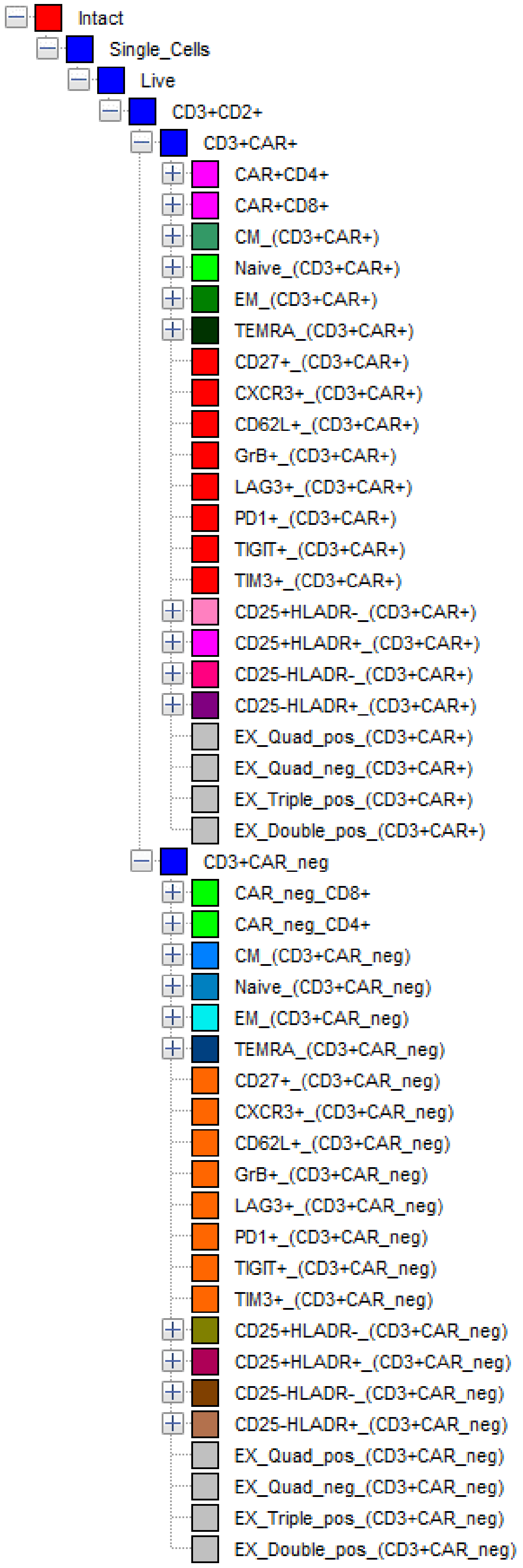

Flow gating strategy.

The sample was first gated on its scatter parameters to provide a population of intact and single cells. The live cells were selected using an amine reactive dye (fixable viability dye 780). The lymphocyte population was selected using anti-CD2 and anti-CD3. The lymphocytes were further subdivided using anti-CD4 and anti-CD8. The transformed CAR population was defined from these populations using a CAR anti-idiotype and secondary donkey anti-rabbit conjugated to PE. Further gating was performed in both the CAR positive and CAR negative cell populations (Figure 1).

The differentiation and memory status of the T-cells was assessed by staining for Anti-CD45RA and Anti-CCR7: dual positive (naïve), single anti-CCR7 positive (central memory), single anti-CD45RA positive (effector memory-TEMRA) and double negative (effector memory).

The exhaustion status of the T-cells is measured using anti-LAG3, anti-PD-1, anti-TIGIT and anti-TIM3.

Supplementary Table 1. Antibody panel for flow cytometry leukapheresis and drug product characterisation

Laser	Filter	Fluor	Antigen	Clone	Titre (µl) or dilution	Cat no	Supplier	Description
UV	UV 379/28	BUV395	HLA DR	G46-6	0.62	564040	BD	Late activation
	UV 515/30	BUV496	CD8	RPA-T8	0.16	612942	BD	Subset
	UV 740/35	BUV737	CD27	M-T271	0.32	741833	BD	Memory phenotype
	UV 805/60	BUV805	CD4	SK3	0.62	612887	BD	Subset
Violet	V 431/28	BV421	CXCR3	1C6/CXCR3	1.25	562558	BD	Trafficking / Upregulated on activation
	V 470/20	BV480	CD45RA	HI100 (BD	0.16	566114	BD	Memory phenotype
	V 610/20	BV605	CD2	RPA-2.10	0.63	300224	Biolegend	-
	V 670/30	BV650	CD62L	DREG-56	0.32	563808	BD	Memory phenotype
	V 710/50	BV711	TIM3	7D3	0.16	565566	BD	Checkpoint inhibitor
	V 780/60	BV786	CD25	M-A251	0.25	563701	BD	Intermediate stage activation
Blue	B 530/30	FITC	LAG3	11C3C65	5	369308	Biolegend	Checkpoint inhibitor
	B 710/50	PerCP eFluor710	Tigit	MBSA43	5	46-9500-42	Invitrogen	Checkpoint inhibitor
Yellow	YG 586/15	PE	Anti-CAR	29F2	Primary 5 Secondary 1:3000	Secondary Ab 406421	Biolegend	Secondary Ab for CAR detection
	YG 610/20	PE-Dazzle	CCR7	G043H7	2.5	353236	Biolegend	Memory phenotype
	YG 780/60	Pe-Cy7	PD-1	EH12.1	0.4	561272	BD	Checkpoint inhibitor
Red	Red 670/30	APC	CD3	SK7	1.25	345767	BD	-
	Red 730/45	AF700	GrB	GB11	2	560213	BD	Cytotoxic T cells
	Red 780/60	FVS780	L/D		1:1000	565388	BD	Live discriminator

Supplementary Note 1. Study Protocol

CLINICAL STUDY PROTOCOL

A SINGLE ARM, OPEN-LABEL, MULTI-CENTRE, PHASE I/II STUDY EVALUATING THE SAFETY AND CLINICAL ACTIVITY OF AUTO4, A CAR T CELL TREATMENT TARGETING TRBC1, IN PATIENTS WITH RELAPSED OR REFRACTORY TRBC1 POSITIVE SELECTED T CELL NON-HODGKIN LYMPHOMA

**Short study title:**	Phase I/II study evaluating AUTO4 in patients with TRBC1 positive T Cell Lymphoma
**Protocol Number:**	AUTO4-TL1
**Study Product:**	AUTO4 for i.v. infusion
**Development Phase:**	I/II
**Sponsor:**	Autolus Limited<br>Forest House<br>58 Wood Lane<br>White City<br>London, W12 7RZ<br>United Kingdom (UK)
**Protocol Version:**	Version 5.0
**EudraCT Number:**	2017-001965-26
**Protocol Date:**	07 July 2021
**Previous Version(s) and Dates**	Version 1.0 – 09 September 2017<br>Version 2.0 – 09 October 2017<br>Version 3.0 – 22 November 2017<br>Version 4.0 – 18 July 2019
**Compliance:**	This study will be conducted in accordance with standards of Good Clinical Practice (as defined by the International Conference for Harmonisation), ethical principles that have their origin in the Declaration of Helsinki and all applicable national and local regulations.

This protocol includes information and data that contain trade secrets and privileged or confidential information that is the property of the Sponsor (Autolus Limited). This information must not be made public without written permission from Autolus Limited. These restrictions on disclosure will apply equally to all future information supplied to you. This material may

be disclosed to and used by your staff and associates as may be necessary to conduct the clinical study.

ADMINISTRATIVE AND CONTACT INFORMATION

Sponsor: Autolus Limited
Forest House
58 Wood Lane
White City
London, W12 7RZ
United Kingdom (UK)
Tel: +44 (0)20 3829 6230

European Union Legal Representative:

Autolus GmbH
Luise-Ullrich-Straße 20
c/o Design Offices Arnulfpark
80636 Munich, Germany

Contact List

The List of Service Providers for the study will be maintained separately by Autolus Limited and kept in the Trial Master File.

Sponsor's Medical Monitor: [redacted]

Notification of SAEs: [redacted]

24-hour Safety Hotline: [redacted]

SPONSOR SIGNATURE PAGE

Study Title: A Single Arm, Open-Label, Multi-Centre, Phase I/II Study Evaluating the Safety and Clinical Activity of AUTO4, a CAR T Cell Treatment Targeting TRBC1, in Patients with Relapsed or Refractory TRBC1 Positive Selected T Cell Non-Hodgkin Lymphoma.

**Short Study Title:**	Phase I/II study evaluating AUTO4 in patients with TRBC1 positive T Cell Lymphoma
**Protocol Number:**	AUTO4-TL1
**Version Number:**	Version 5.0
**EudraCT No.:**	2017-001965-26
**Version Date:**	07 July 2021

I have read the protocol AUTO4-TL1 titled "A Single Arm, Open-Label, Multi-Centre, Phase I/II Study Evaluating the Safety and Clinical Activity of AUTO4, a CAR T Cell Treatment Targeting TRBC1, in Patients with Relapsed or Refractory TRBC1 Positive Selected T Cell Non-Hodgkin Lymphoma" and confirm that, to the best of my knowledge, the protocol accurately describes the design and conduct of the study.

This document has been electronically signed within the electronic data management system. Please refer to the last page of the document for the electronic signature.

Signature	Date
[redacted]	(DD MMM YYYY)

INVESTIGATOR SIGNATURE PAGE

Study Title: A Single Arm, Open-Label, Multi-Centre, Phase I/II Study Evaluating the Safety and Clinical Activity of AUTO4, a CAR T Cell Treatment Targeting TRBC1, in Patients with Relapsed or Refractory TRBC1 Positive Selected T Cell Non-Hodgkin Lymphoma.

**Short study title:**	Phase I/II study evaluating AUTO4 in patients with TRBC1 Positive T Cell Lymphoma
**Protocol Number:**	AUTO4-TL1
**EudraCT No.:**	2017-001965-26
**Version Number:**	Version 5.0
**Version Date:**	07 July 2021

I have read the protocol AUTO4-TL1 titled "A Single Arm, Open-Label, Multi-Centre, Phase I/II Study Evaluating the Safety and Clinical Activity of AUTO4, a CAR T Cell Treatment Targeting TRBC1, in Patients with Relapsed or Refractory TRBC1 Positive Selected T Cell Non-Hodgkin Lymphoma". By signing this protocol, I agree to conduct the clinical study, after approval by an Institutional Review Board or Independent Ethics Committee (as appropriate), in accordance with the protocol, the principles of the Declaration of Helsinki (2013), the standards of Good Clinical Practice (as defined by the International Conference for Harmonisation) and applicable regulatory requirements.

Changes to the protocol will only be implemented after written approval is received from Autolus Limited and the Institutional Review Board or Independent Ethics Committee (as appropriate), with the exception of medical emergencies. I will ensure that study staff fully understand and follow the protocol.

Signature Date

Name and address: (DD MMM YYYY)

PROTOCOL SYNOPSIS

**Title**	A Single Arm, Open-Label, Multi-Centre, Phase I/II Study Evaluating the Safety and Clinical Activity of AUTO4, a CAR T Cell Treatment Targeting TRBC1, in Patients with Relapsed or Refractory TRBC1 Positive Selected T Cell Non-Hodgkin Lymphoma.
**Protocol Number**	AUTO4-TL1
**EudraCT No.:**	2017-001965-26
**Sponsor**	Autolus Limited
**Phase**	I/II
**Study Product**	AUTO4 is an autologous, cell and gene therapy investigational medicinal product that is manufactured by genetic modification of the patient's own T cells by ex vivo transduction with a murine leukaemia virus-derived retroviral vector that has been engineered to stably deliver the RQR8 and anti- T cell receptor beta constant (TRBC1)-chimeric antigen receptor (CAR) (aTRBC1-CAR) genes. The active substance is the genetically modified (RQR8/aTRBC1-CAR positive) T cells.
**Study Population**	Patients with confirmed diagnosis of TRBC1 positive selected T cell non -Hodgkin lymphoma (T-NHL) who have relapsed or become refractory after exposure to ≥1 line of therapy.
**Study Duration**	The study will take approximately 7 years. The end of the trial will be 24 months after the last patient has received an AUTO4 infusion or the last patient last visit if this occurs earlier due to patient death or premature withdrawal before 24 months.
**Overview**	A single arm, open-label, multi-centre, Phase I/II dose escalation and expansion study to determine the safety and clinical activity of RQR8/aTRBC1-CAR positive T cells administered intravenously (i.v.) in patients with TRBC1 positive selected T-NHL.<br><br>During T cell development, each T cell generates a unique T cell receptor (TCR) by rearrangement of the alpha and beta chains. Each beta chain also includes a constant domain, which is not involved in antigen recognition. A facet of TCR gene-rearrangement is that the coding sequence for the T cell receptor beta Constant (TRBC) region is duplicated (TRBC1 and TRBC2). Hence, a mature T cell, and any subsequent clonal malignancy, is highly likely to express either TRBC1 or TRBC2. Normal T cells (including T cell subsets) comprise a mixture of T cells (approximately 40%, 60% mix) individually expressing either TRBC1 or TRBC2. Natural immunity including antiviral immunity is also distributed across the two populations. T cell lymphomas on the other hand are clonal tumours and are either entirely TRBC1 or TRBC2 positive.<br><br>In this study, Autolus plans to recruit patients with TRBC1 positive selected T cell lymphomas. Patients will receive autologous CAR T cells directed against TRBC1. These CAR T cells will target the lymphoma in its entirety as well as a proportion of normal T cells (i.e., those which are TRBC1 T cells) with the aim of maintaining immunity due to the preservation of TRBC2 positive cells.

	This first-in-human (FIH) study will assess the safety of AUTO4, determine the appropriate dose for Phase II and evaluate the efficacy of the AUTO4 in patients with selected relapsed or refractory T-NHL.
**Primary Objective(s) and Endpoints**	The primary objectives of the study are as follows:

Objectives	Endpoints
**Phase I**	
To assess the safety and tolerability of AUTO4 administration.	Incidence of Grade 3 to 5 toxicity occurring within 60 days of AUTO4 infusion.
To identify the recommended Phase II dose (RP2D) and maximum tolerated dose (MTD), if an MTD exists, of AUTO4.	Frequency of dose limiting toxicity (DLT) of AUTO4 within 28 days of AUTO4 infusion.
**Phase II**	
To assess the clinical activity of AUTO4 when administered at the RP2D.	Primary endpoint:<br>Overall response (CR+PR) rate post AUTO4 infusion.

Study Design

This is a Phase I/II, open-label, multi-centre study to characterise the safety and clinical activity of RQR8/aTRBC1-CAR T cells when administered to patients with relapsed or refractory TRBC1 positive selected T-NHL. The study will consist of two phases, a Phase I dose escalation, followed by a Phase II dose expansion. Both phases of the study will involve patients going through the following five sequential stages: screening (including TRBC assessment), leukapheresis, pre-conditioning, treatment with AUTO4 and follow-up.

Phase I (Dose Escalation): To identify the recommended Phase II dose (RP2D) based on safety, tolerability and anti-tumour activity of AUTO4 using a rolling six design. Up to five cohorts and a maximum of 25 patients with TRBC1 positive T-NHL will be dosed. The following dose levels are intended to be evaluated, but are subject to emerging safety data: 25 x 10^6, 75 x 10^6, 225 x 10^6 450 and 900 x 10^6 RQR8/aTRBC1-CAR positive T cells.

Phase II (Dose Expansion): To further characterise the safety and assess the efficacy of AUTO4 at the recommended dose identified in Phase I, up to 30 patients will be treated in Phase II, following a Simon's two-stage optimal design (Simon 1989).

Biomarkers relating to the CAR T cells and tumour will be evaluated in all patients. All patients treated in Phase I and II of the study will attend clinic visits until End of Study (defined as 24 months post AUTO4 infusion of the last patient treated, or less in the event of early withdrawal) for study-specific assessments including adverse event (AE) assessments, ECOG, physical examinations, disease assessment and laboratory and immunology tests.

All AUTO4 treated patients will be followed until death, study closure or study consent withdrawl (whichever occurs first). After the End of Study patients treated with AUTO4 will be invited to enrol on a long-term follow-up study protocol and will be followed for long-term safety and disease assessment (if applicable) until death or withdrawal of consent for up to 15 years from treatment administration.

**Number of Patients**	Approximately 200 patients in total are expected to be consented, registered and screened for eligibility. This assumes that approximately 35% of patients will be identified as TRBC1 positive (and assumes that a small number of patients will not provide sufficient evaluable tissue). Of those that are identified as TRBC1 positive, it is assumed that approximately 20% will fail the AUTO4 manufacture and/or will not continue to meet the inclusion and exclusion entry criteria. Up to 55 patients in total are anticipated to be treated with AUTO4 therapy.<br>• **Phase I (Escalation):** up to five dose cohorts and up to 25 patients in total (three to six patients per cohort) following a rolling six dose escalation design.<br>• **Phase II (Expansion):** up to 30 evaluable patients in total, using Simon's two-stage optimal design.<br>Simon's two-stage design trial will be used (Simon 1989). The null hypothesis that the true response rate is 10% will be tested against a onesided alternative. In the first stage, 10 evaluable patients will be accrued (12 weeks post treatment of tenth evaluable patient). If there are one or fewer responses in these 10 patients, the study will be stopped. Otherwise, 19 additional evaluable patients will be accrued for a total of 29. The null hypothesis will be rejected if six or more responses are observed in 29 patients. This design yields a type I error rate of 5% and 80% power when the true response rate is 30%.
**Criteria for Eligibility**	Only patients with confirmed TRBC1 positive selected T-NHL, and whose leukapheresis sample has been successful in generating AUTO4 in adequate quantity will be treated. The criteria for patient eligibility are summarised as follows:<br>**Inclusion Criteria:** Patients must meet all the following criteria for study entry:<br>1 Male or female, aged ≥18 years.<br>2 Willing and able to give written, informed consent to be screened for TRBC1 positive T-NHL and to enter the main study.<br>3 Confirmed diagnosis of selected T-NHL, including:<br>a Peripheral T cell lymphoma NOS, or<br>b Angioimmunoblastic T cell lymphoma, or<br>c Anaplastic large cell lymphoma<br>4 Confirmed TRBC1 positive tumour.<br>5 Relapsed or refractory disease and have had ≥1 prior lines of therapy.<br>6 Positron emission tomography (PET)-positive measurable disease per Lugano classification.<br>7 Eastern Cooperative Oncology Group (ECOG) Performance Status 0 or 1.<br>8 Adequate bone marrow function without the requirement for ongoing blood products and meets the following criteria:<br>a Absolute neutrophil count ≥1.0 x 10^9/L<br>b Absolute lymphocyte count ≥0.5 x 10^9/L (at entry and prior to leukapheresis).<br>c Haemoglobin ≥80 g/L<br>d Platelets ≥75 x 10^9/L<br>9 Adequate renal, hepatic, pulmonary, and cardiac function defined as:<br>a Creatinine clearance (as estimated by Cockcroft Gault) ≥60 cc/min.

b Serum alanine aminotransferase/aspartate aminotransferase ≤2.5 x upper limit of normal (ULN).

c Total bilirubin ≤25 µmol/L (1.5 mg/dL), except in patients with Gilbert's syndrome.

d Left ventricular ejection fraction (LVEF) ≥50% by echocardiogram (ECHO) or multiple gated acquisition (MUGA) cardiac scan, unless the institutional lower limit of normal is lower.

e Baseline oxygen saturation ≥92% on room air and ≤Grade 1 dyspnoea.

10 For females of childbearing potential (defined as <2 years after last menstruation or not surgically sterile), a negative serum or urine pregnancy test must be documented at screening, prior to pre-conditioning and confirmed before receiving the first dose of study treatment.

For females who are not postmenopausal (<24 months of amenorrhea) or, who are not surgically sterile (absence of ovaries and/or uterus), a highly effective method of contraception together with a barrier method must be used from the start of the pre-conditioning stage and for at least 12 months after the last dose of AUTO4 (the study treatment). They must agree not to donate eggs (ova, oocytes) for the purposes of assisted reproduction during the study and for 12 months after receiving the last dose of study drug (please refer to Appendix 3).

11 For males, it must be agreed that two acceptable methods of contraception are used from the start of the pre-conditioning stage and for at least 12 months after the last dose of AUTO4 (one by the patient – usually a barrier method, and one by the patient's partner - refer to Appendix 3). Also, that sperm will not be donated during the treatment period and for at least 12 months after the last dose of study treatment.

12 No contra-indications for leukapheresis, or the pre-conditioning regimen.

Exclusion Criteria: Patients meeting any of the following exclusion criteria must not be enrolled into the study:

1 Patients with T cell leukaemia.

2 Females who are pregnant or lactating.

3 Prior treatment with investigational gene therapy or approved gene therapy or genetically engineered cell therapy product or allogeneic stem cell transplant.

4 Known history or presence of clinically relevant central nervous system (CNS) pathology, such as epilepsy, paresis, aphasia, stroke within the prior 3 months, severe brain injuries, dementia, Parkinson's disease, cerebellar disease, organic brain syndrome, uncontrolled mental illness, or psychosis. Patients with a known history or prior diagnosis of optic neuritis or other immunologic or inflammatory disease affecting the CNS.

5 Current or history of CNS involvement by malignancy.

6 Clinically significant, uncontrolled heart disease (New York Heart Association Class III or IV heart failure, uncontrolled angina, severe uncontrolled ventricular arrhythmias, sick-sinus syndrome, or electrocardiographic evidence of acute ischaemia or Grade 3 conduction system abnormalities unless the patient has a pacemaker) or a recent (within 12 months) cardiac event.

a Uncontrolled cardiac arrhythmia (patients with rate-controlled atrial fibrillation are not excluded).

b Evidence of pericardial effusion.

7 Patients with evidence of uncontrolled hypertension or with a history of hypertension crisis or hypertensive encephalopathy.

8 Patients with a history (within 3 months) or evidence of deep vein thrombosis or pulmonary embolism requiring ongoing therapeutic anticoagulation at the time of pre-conditioning.

9 Patients with active gastrointestinal (GI) bleeding.

10 Patients with any major surgical intervention in the last 3 months.

11 Active infectious bacterial, viral disease or fungal disease (hepatitis B virus, hepatitis C virus, human immunodeficiency virus [HIV], human T cell lymphotropic virus [HTLV] or syphilis) requiring treatment.

12 Active autoimmune disease requiring immunosuppression.

13 History of other neoplasms unless disease free for at least 2 years (adequately treated carcinoma *in situ*, curatively treated non-melanoma skin cancer, breast or prostate cancer on hormonal therapy are allowed).

14 Prior treatment with programmed cell death protein 1 (PD1), programmed death-ligand 1 (PD-L1), or cytotoxic T lymphocyte-associated protein 4 (CTLA-4) targeted therapy, or tumour necrosis factor (TNF) receptor superfamily agonists including CD134 (OX40), CD27, CD137 (41BB), and CD357 (glucocorticoid-induced TNF receptor family-related protein) within 6 weeks prior to AUTO4 infusion.

15 The following medications are excluded:

a Steroids: Therapeutic doses of corticosteroids within 72 hours of leukapheresis or pre-conditioning chemotherapy administration. However, physiological replacement, topical, and inhaled steroids are permitted.

b Cytotoxic chemotherapies within 2 weeks prior to leukapheresis or AUTO4 infusion.

c Antibody therapy use within 2 weeks prior to AUTO4 infusion, or five half-lives of the respective antibody, whichever is longer.

d Live vaccine within 4 weeks prior to enrolment.

16 Research participants receiving any other investigational agents, or concurrent biological, chemotherapy, or radiation therapy.

17 Use of rituximab (or rituximab biosimilar) within the last 6 months prior to AUTO4 infusion.

18 Patients, who in the opinion of the Investigator, may not be able to understand or comply with the safety monitoring requirements of the study.

For pre-conditioning chemotherapy and AUTO4 Infusion: Patients meeting any of the following exclusion criteria must not be treated with pre-conditioning chemotherapy or AUTO4 - and should have treatment delayed until they no longer meet these criteria:

1 Severe intercurrent infection at the time of pre-conditioning chemotherapy or the scheduled AUTO4 infusion.

2 Requirement for supplementary oxygen or active pulmonary infiltrates or significant deterioration of organ function at the time of pre-conditioning chemotherapy or scheduled AUTO4 infusion.

	3	Significant clinical deterioration of organ functions from screening, as determined by the investigator.

**Study Product Dose, Dosing Regimen, and Administration**	The AUTO4 product consists of enriched autologous genetically modified T cells (RQR8/aTRBC1-CAR positive T cells), that express RQR8 and the TRBC1 CAR on the cell surface, and non-genetically modified T cells. The dose is expressed as the number of RQR8/aTRBC1-CAR positive T cells. AUTO4 also contains non-transduced autologous lymphocytes as a product-related impurity. Eligible patients will receive a single dose i.v. infusion of AUTO4 following pre-conditioning treatment.<br>Up to five dose levels are planned to be explored.<br>The starting dose will be 25 x 10^6 RQR8/aTRBC1-CAR positive T cells administered as a single dose. The other planned doses are 75 x 10^6, 225 x 10^6 450 and 900 x 10^6 RQR8/aTRBC1-CAR positive T cells.<br>All patients will be admitted to the hospital for at least 14 days for monitoring after receiving AUTO4.
**Pre-conditioning Treatment**	All patients will receive a pre-conditioning regimen using fludarabine (FLU) 30 mg/m^2 i.v. immediately followed by cyclophosphamide (CY) 500 mg/m^2 i.v. on Days -6 and -5, and FLU 30 mg/m^2 i.v. and on Days -4, -3 (-1 day) before AUTO4 infusion.
**Safety Evaluation**	Safety will be assessed by physical examinations, ECOG status, vital signs, laboratory tests, electrocardiograms, AE and serious AE monitoring, and concomitant medication usage. The severity of AEs will be assessed using the National Cancer Institute Common Terminology Criteria for Adverse Events (Version 5.0), with the exception of neurotoxicity, which will be assessed according to the ASBMT Immune Effector Cell-associated Neurotoxicity Syndrome (ICANS) grading criteria.
**Efficacy Evaluation**	Efficacy will be evaluated as per Lugano Classification.<br>Response evaluations will be assessed by PET scans, computed tomography (CT) scans, magnetic resonance imaging (MRI), physical examination, and bone marrow biopsy (if needed).
**Biomarker Evaluation**	Post AUTO4 infusion biomarker evaluation will include the following:<br>• Expansion and persistence of RQR8/aTRBC1-CAR positive T cells as determined by polymerase chain reaction (PCR) and/or flow cytometry.<br>• Depletion of TRBC1 positive T cell compartment as determined by flow cytometry on peripheral blood.
**Special Study Procedures**	A separate tumour tissue consent will be sought from all patients who are entering the selection process for this trial for the provision of an archival lymph node tumour tissue or a newly acquired tumour tissue for evaluation of TRBC1 expression. If a patient cannot provide sufficient archival tumour tissue for the evaluation of TRBC1 expression, newly acquired tumour tissue can be obtained by core needle biopsy or excisional biopsy. If a core needle is used, an absolute minimum of two cores are required for the determination of TRBC1 status. Timing of screening biopsy should be discussed with Sponsor to ensure the result (TRBC1 positive or not) is known prior to the planned leukapheresis.<br>Only patients identified as TRBC1 positive will be invited to consent to the main study. These patients will undergo an unstimulated leukapheresis for AUTO4 generation. Where feasible, a fraction of leukapheresate will be stored as backup

	(rescue treatment) for immune reconstitution. This can be collected at the same time or at a second unstimulated leukapheresis prior to pre-conditioning.
**Statistical Analysis**	The data will be summarised using descriptive statistics. Continuous variables will be summarised using the number of observations, mean, standard deviation, median, and range as appropriate. Categorical values will be summarised using the number of observations, percentages and confidence intervals as appropriate. Time-to-event endpoints will be estimated using Kaplan-Meier methodology.<br>The Phase II part of the study will treat up to 30 evaluable patients. Overall response rate with two-sided 95% confidence interval will be estimated based on evaluable patients. The definition of an evaluable patient (for efficacy) is one that has received an AUTO4 dose (either partial or complete), and has measurable disease before starting the pre-conditioning therapy (and after receiving any bridging therapies).<br>Data from all treated patients (safety analysis set) will be used for safety analyses and will be summarised by dose level.
**Interim Analyses**	In Phase II of the study, an interim analysis will be performed after 10 evaluable patients are treated and had the opportunity to be followed for at least 12 weeks. The study will be stopped at this first stage if no more than one response has been observed.

Table 1 SCHEDULE OF ASSESSMENTS 1 (Safety and Efficacy Follow-up)

	SCREEN TRBC	SCREEN 1[$]	LEUKA-PHERESIS[@]	SCREEN 2[#]	PRE-CONDITIONING CHEMO-THERAPY		TREATMENT STAGE†				FOLLOW-UP STAGE		END OF STUDY **
		Day -84 to Day -35	Before Day -35	Day -35 to -7	Day -7 (-1d)	Day -6, -5, -4, -3 (-1 d)	Day 0	Day 1, then q.a.d. Hospitalisation ≥14 d	Weekly after discharge (±2 d)	End of DLT period* Day 28 (±3 d)	Month 2, 3, 4, 5, 6, 9, 12, 15, 18 & 24 (±7 d)	Every 6 months until EoS** (±4 wk)	
Informed Consent	Part A	Part B											
Demographic Data[1]	X												
Eligibility Criteria[2]	X	X			X		X						
Medical/ Lymphoma History[3]	X				X								
**Examinations/Investigations**													
ECOG Performance Status	X	X			X					X	X		X
Physical Examination[4]		X			X		X	X[D7, D14]	X	X	X		X
Weight		X	X		X					X	X		X
Vital Signs[5]		X			X		X[5]	X[D7, D14]	X	X	X		X
12-lead ECG[6]		X			X[6]								
ECHO or MUGA[7]		X											
**Tumour Tissues Assessment**													
Tumour Tissue Sample (TRBC1 status) [8]	X												

	SCREEN TRBC	SCREEN 1[$]	LEUKAPHERESIS[@]	SCREEN 2 [#]	PRE-CONDITIONING CHEMO-THERAPY		TREATMENT STAGE†				FOLLOW-UP STAGE		END OF STUDY **
		**Day -84 to Day -35**	**Before Day -35**	**Day -35 to -7**	**Day -7 (-1d)**	**Day -6, -5, -4, -3 (-1 d)**	**Day 0**	**Day 1, then q.a.d. Hospitalisation ≥14 d**	**Weekly after discharge (±2 d)**	**End of DLT period* Day 28 (±3 d)**	**Month 2, 3, 4, 5, 6, 9, 12, 15, 18 & 24 (±7 d)**	**Every 6 months until EoS** (±4 wk)**	
Tumour Tissue Sample (CAR T-cell persistence/TRBC1 expression)[9]								X [between D7-D21]			X[prog]		
Bone Marrow Biopsy (if applicable) [10]				X						X[10]			
**Disease Evaluations**													
CT Imaging (neck, chest, abdomen and pelvis)[11]				X						X	X[M3, M6, M9, M12, M15, M18, M24]	X	
18-FDG-PET Scan (skull base to the proximal femur)[11]				X						X	X[M3, M6, M9, M12, M15, M18, M24]	X	
**Safety Labs**													
Haematology[12]		X	X		X		X	X[D1, D7, D14]	X	X	X		
Biochemistry [13]		X	X		X		X	X[D1, D7, D14]	X	X	X		
Ferritin, C reactive protein[14]					X		X	X	X	X			
Coagulation[15]		X	X		X		X	X[D7, D14]					

	**SCREEN TRBC**	**SCREEN 1[$]**	**LEUKA-PHERESIS[@]**	**SCREEN 2 [#]**	**PRE-CONDITIONING CHEMO-THERAPY**		**TREATMENT STAGE†**				**FOLLOW-UP STAGE**		**END OF STUDY ****
		**Day -84 to Day -35**	**Before Day -35**	**Day -35 to -7**	**Day -7 (-1d)**	**Day -6, -5, -4, -3 (-1 d)**	**Day 0**	**Day 1, then q.a.d. Hospitalisation ≥14 d**	**Weekly after discharge (±2 d)**	**End of DLT period* Day 28 (±3 d)**	**Month 2, 3, 4, 5, 6, 9, 12, 15, 18 & 24 (±7 d)**	**Every 6 months until EoS** (±4 wk)**	
Infectious Disease Screen[16]		X	X[16]										
Monitoring for infections[17]				X						X	X		
Pregnancy Test[18]		X			X		X			X	X[M3, M6, M12]		

	SCREEN TRBC	SCREEN 1$	LEUKA-PHERESIS@	SCREEN 2 #	PRE-CONDITIONING CHEMO-THERAPY		TREATMENT STAGE†				FOLLOW-UP STAGE		END OF STUDY **
		Day -84 to Day -35	Before Day -35	Day -35 to -7	Day -7 (-1d)	Day -6, -5, -4, -3 (-1 d)	Day 0	Day 1, then q.a.d. Hospitalisation ≥14 d	Weekly after discharge (±2 d)	End of DLT period* Day 28 (±3 d)	Month 2, 3, 4, 5, 6, 9, 12, 15, 18 & 24 (±7 d)	Every 6 months until EoS** (±4 wk)	
**Pharmacokinetics, Pharmacodynamic and Biomarker Assays**													
Serum for cytokines[19]				X	X	X[D-6]	X[P]	X	X	X	X[M2, M3]		
Central Assessment- Blood for Tcell Subset (TRBC1+, TRBC1-, CD3+CD4+, CD3+ CD8+) Analysis [20]		X			X		X	X[D14 only]		X	X[M2, M3, M4, M5, M6, M9, M12, M15, M18, M24]		
Local Assessment- Blood for Tcell Subset (CD3+CD4+, CD3+CD8+) Analysis [20]		X			X		X	X[D14 only]		X	X[M2, M3, M4, M5, M6, M9, M12, M15, M18, M24]	X	
Blood for CAR T Cells persistance, PCR [21]					X		X[P]	X	X	X	X[M2, M3, M4, M5, M6, M9, M12, M15, M18, M24]	X	
Blood for CAR T Cells, Flow [21]					X		X[P]	X	X	X	X[M2, M3, M4, M5, M6, M9, M12, M15, M18, M24]	X	

	SCREEN TRBC	SCREEN 1$	LEUKA-PHERESIS@	SCREEN 2 #	PRE-CONDITIONING CHEMO-THERAPY		TREATMENT STAGE†				FOLLOW-UP STAGE		END OF STUDY **
		Day -84 to Day -35	Before Day -35	Day -35 to -7	Day -7 (-1d)	Day -6, -5, -4, -3 (-1 d)	Day 0	Day 1, then q.a.d. Hospitalisation ≥14 d	Weekly after discharge (±2 d)	End of DLT period* Day 28 (±3 d)	Month 2, 3, 4, 5, 6, 9, 12, 15, 18 & 24 (±7 d)	Every 6 months until EoS** (±4 wk)	
Blood for RCR Testing & Insertional Mutagenesis					X					X	$X^{M3, M6, M12, M18, M24}$	X	
Immunological / genomic profiling[22]					X		X^{P}	$X^{D7, D14}$	X	X	$X^{M3, M6, M9, M12, M15, M18, M24}$		
**Treatments**													
CY and FLU						X							
AUTO4 Infusion							X						
**Adverse events**													
Adverse Events[23]	X	X	X	X	X	X	X	X	X	X	X	X	X
Concomitant Medication[24]					X	X	X			X	X^{M2}	X	

Abbreviations: AE = adverse event; ALT = alanine aminotransferase; AST = aspartate aminotransferase; CAR = chimeric antigen receptor; CMV= Cytomegalovirus; CPK = creatine phosphokinase; CR = complete response; CRS = cytokine release syndrome; CT = computed tomography; CY = cyclophosphamide; D,d = day; DLT = dose limiting toxicity; EBV = Epstein Barr Virus; ECG = electrocardiogram; ECOG = Eastern Cooperative Oncology Group; ECHO = echocardiogram; EDTA = ethylenediaminetetraacetic acid; FDG = fluorodeoxyglucose; FFPE = formalin fixed paraffin embedded; FLU = fludarabine; JCV=John Cunningham Virus; M = month (where each month is approximately 4.2 weeks, with 12 months per year); MRI = magnetic resonance imaging; MUGA = multigated acquisition (cardiac scan); PET = positron emission tomography; q.a.d. = quaque altera die (every other day); RCR = replication competent retrovirus; SAE = serious adverse event; TRBC = T cell receptor beta constant.

*: End of DLT period set as **28 days** after the dose of AUTO4.

: End of Study visit is to be performed upon completion of all other study visits or in case of premature withdrawal. **The end of the study (EoS) is defined as the LPLV expected to be 24 months after the last treated patient with AUTO4 or earlier in the event of patient death or consent withdrawal. Of Note: The patients who experienced disease progression post AUTO4 infusion will continue to be followed under this study protocol until death, study closure or consent withdrawal, whichever occurs earlier (See section 8.5.2, Schedule of Assessments Table 2)

Note: Additionally, blood samples for CAR T persistence and RCR may also be collected

$: All tests must be undertaken (and results known) before leukapheresis.

@: Leukapheresis occurs after a patient is confirmed as TRBC1 positive.

X^{Dx}: Test to be performed on a particular day or month of the schedule rather than systematically at every visit. Please refer to the number to determine the day or month of assessment.

X^{p}: Sample to be taken prior to infusion.

X^{prog}: Test to be performed at disease progression.

Enrolment confirmed once all inclusion and exclusion criteria have been fulfilled and leukapheresate has been accepted for manufacturing.

Schedule of Assessment Footnotes:

1. Demographic data: race and ethnicity, height, age (month and year) and gender.
2. Eligibility criteria: Performance, disease characteristics and organ and bone marrow function to be assessed before a new node biopsy (if patient will undergo a node biopsy). ECHO may be done after node biopsy. Eligibility criteria to be re-assessed on Day -7 (-1 day) prior to pre-conditioning when the patient should continue to meet renal, hepatic, pulmonary function and performance status requirements. On Day 0, before infusion, it will be assessed whether the patient meets the AUTO4 infusion criteria.
3. Medical/lymphoma history: to include all current and prior clinically significant diseases, surgeries, cancer history (including prior T-cell lymphoma therapies or any other cancer therapies and procedures) and prior relevant medications). Obtain histological confirmation of disease diagnosis (pathology report) and the presence of T-cell lymphoma in the archived node tissue (if archived tissue is used for the TRBC1 status assessment). Record disease status at Screen 2 after leukapheresis has been completed.
4. Physical examination: a complete physical examination and complete neurological examination to be performed at Screen 1, Day -7 (-1 day) and Day 0; then focused and/or symptom related examination as appropriate at following visits.
5. Vital signs: temperature, systolic and diastolic blood pressure, pulse/heart rate, oxygen saturation and respiratory rate will be performed while the patient is in a seated position or supine. On Day 0 of any treatment stage, record vital signs immediately prior to AUTO4 infusion and every 30 minutes (± 10 min) for the next 4 hours post AUTO4 infusion, and thereafter monitored as per hospital policy but no less than 3 times a day whilst the patient is in hospital. Record weight as per Schedule of Assessments above.
6. 12-lead ECG: Repeat as clinically necessary and when patient experiences CRS.
7. ECHO or MUGA cardiac scan: to be performed at Screen 1 and to be repeated if clinically indicated. Same method should be used throughout the study.
8. Newly acquired tumour tissue sample may be required to determine TRBC1 status unless sufficient archival tumour biopsy material can be obtained – either by core needle biopsy or excisional biopsy (archived tissue must not be >5 years old and subtype of T-NHL unchanged from time of archived tissue to current status). If a core needle is used, **an absolute minimum of two cores** are required for the evaluation of TRBC1 expression on T cells using the LymphoTrack Dx TRB Assay. However, additional two cores are requested (if medically feasible) for the further development of a TRBC companion diagnostic assay and/or biomarker assessment on FFPE tissue.

9. Lymph node Tumour Tissue Sample for CAR T-cell persistence/TRBC1 expression. If there is a suitable lesion in an accessible location which would not put the patient at any safety risk per treating physician judgment. Biopsy samples should be taken once between Day 7 and Day 21 and at the time of progression. The tumour samples will be analysed by flow cytometry or immunohistochemistry.
10. Bone Marrow Biopsy. If the Investigator suspects there is lymphoma infiltration in the bone marrow, a bone marrow biopsy should be performed at screening (if patient receives bridging therapy it should be done after any bridging therapy). If a bone marrow biopsy is performed and shows lymphoma infiltration, a bone marrow biopsy should be repeated at the time of first complete response.
11. Imaging and scans: For those patients receiving a bridging chemotherapy regimen, the baseline PET/CT scans must be done after completion of bridging therapy and before start of the preconditioning and AUTO4 infusion. 18-FDG-PET Scan: If at 6 months the patient has a CMR on PET scan, CT scans alone may be used for future assessment timepoints, if clinically appropriate. If relapse occurs after CMR or disease progression is suspected (e.g. new or enlarging lesion(s) detected on CT scan), a PET scan is to be repeated to confirm relapse/progression. MRI may be used to evaluate sites of disease that cannot be adequately imaged using CT (in cases where MRI is desirable, the MRI must be obtained at baseline and at all subsequent response evaluations). For all other sites of disease, MRI studies do not replace the required neck, chest, abdomen, and pelvic CT scans. Brain MRI is only required if clinically indicated. If progression is suspected from scan(s), but the patient is otherwise not showing clinical progression/deterioration, the disease progression must be confirmed not less than 28 days after initial finding to rule out a pseudo-progression.
12. Haematology: haemoglobin, red blood cell count, platelet count, white blood cell count with differential (neutrophils, eosinophils, lymphocytes, monocytes, and basophils). Test to be performed prior to chemotherapy on pre-conditioning days and prior to AUTO4 infusion on Day 0 of any treatment stage.
13. Biochemistry: Whole panel: sodium, phosphate, potassium, magnesium, chloride, bicarbonate, ALT, AST, urea or blood urea nitrogen, creatinine, serum CPK, lactate dehydrogenase, glucose, total bilirubin, calcium (albumin adjusted), total protein, albumin . Serum uric acid to be measured only on Day 0, 1, and 7 of any AUTO4 treatment stage. All tests must be performed prior to AUTO4 infusion on Day 0 of any AUTO4 treatment stage. Glomerular filtration rate should be calculated at screening as per institutional preferred method.
14. Ferritin, C reactive protein: May be done more frequently as clinically necessary and during CRS if necessary.
15. Coagulation: prothrombin time, international normalised ratio, activated partial thromboplastin time, fibrinogen. Day 7, after AUTO4 infusion.
16. Infectious disease screen: must be performed at screening for the eligibility criteria and within 30 days prior to leukapheresis and must be negative. It must be repeated on the day of leukapheresis (or within 7 days after). HIV-1 and 2, Hep B virus, Hep C virus, HTLV-1, HTLV-2, Syphilis .
17. Monitoring for infections: CMV, EBV & adenovirus monitoring as per schedule in table. Additonal monitoring for opportunistic infections, such as JCV, toxoplasmosis and fungal infections as per institutional guidelines (e.g institutional guidelines used for bone marrow transplant patients) or as clinically indicated.. Monitoring beyond 3 months should be done if there is low levels of CD4+ T-cells or if clinically indicated.
18. Pregnancy test: serum (β-human chorionic gonadotropin) or urine pregnancy testing for women of childbearing potential.
19. Serum for cytokines and biomarkers: During hospital stay, sample collection to be performed every other day (± 1 day). If patient experiences ≥Grade 2 CRS then additional samples should be collected daily until CRS resolves or clinically indicated
20. Blood for analysis of T cell Subsets will be done both locally and via a Central Lab. Samples are to be collected at the following timepoints: Screening, Day -7, Day 0 (predose), Day 14, Day 28, Month 2, 3, 4, 5, 6, 9, 12, 15, 18, 24, and as clinically indicated e.g. in case of opportunistic infections
21. Blood for CAR T cells persistance: one sample to be taken on Day-7, Day 0 prior to AUTO4 infusion. During hospital stay, sample collection to be performed every other day (± 1 day) and ideally on a Monday to Friday. Following hospital discharge (Day 14), blood samples for CAR- T cells (by flow and PCR) are to be collected at the following timepoints: Day 21, Day 28, Month 2, 3, 4, 5, 6, 9, 12, 15, 18 and 24. Additional samples should be collected if clinically indicated ; an additional sample should be collected at the time of disease progression.
22. Blood for Immunological / genomic profiling: During hospital stay, sample collection to be performed on Day 7 and Day 14 (± 1 day) and ideally on a Monday to Friday

23. Adverse Events: Only AEs/SAEs related to study procedures should be collected until admission for lymphodepletion chemotherapy (Day -6 [-1 day]). AEs related to intervening/bridging non-study related anti-cancer therapy administered prior to pre-conditioning or AEs associated with disease progression during the same period will not be reported as AEs. These events will be recorded as an update to the patient's medical history. After Day 60, only collect: All SAES and AUTO4 treatment-related non-serious AEs; All AEs of special interest and AEs related to a study procedure. Please refer to Section 12 for specifics about the AE reporting periods.
24. Concomitant medications: Collect as described in the Schedule of Assessments. Before Day -7 (-1 day) and after 2 months, collect only concomitant medications relevant to AUTO4 treatment-related Grade 3 to 4 AEs and treatment-related SAEs; AEs of special interest or AEs related to a study procedure.

Note: The total estimated volume of blood collected for safety, biomarkers and immunological assessments (with the exception of the leukapheresis procedure) across any one year will not normally exceed 850 mL (this is expected volume for females with serum pregnancy test). The maximum volume of blood collected on any day will unlikely exceed 80mL. No more than 470mL of blood will be collected in any 28-day period.

Table 2 SCHEDULE OF ASSESSMENTS 2 (Safety and Survival Follow-up)

Applies to all patients who fail to respond to treatment or who respond but subsequently experienced disease progression. Please refer to the relevant abbreviations and footnotes in **Error! Reference source not found.** and to Section **Error! Reference source not found.** for further details.

Visits / Assessments	SAFETY AND SURVIVAL FOLLOW-UP							END OF STUDY
	M2 ±7d	M3 ±7d	M6 ±7d	M9 ±7d	M12 ±7d	M24 ±7d	Every 6 months until EoS** # ±4wk	EoS**/Early Withdrawal
**PATIENT INFORMATION**								
Subsequent anti-T-cell lymphoma therapy and disease response	X							
Survival Status	X							
Selected Concomitant Medication [23]	X							
**EXAMINATIONS, INVESTIGATIONS AND SAFETY EVALUATIONS**								

Visits / Assessments	SAFETY AND SURVIVAL FOLLOW-UP							END OF STUDY
	M2 ±7d	M3 ±7d	M6 ±7d	M9 ±7d	M12 ±7d	M24 ±7d	Every 6 months until EoS** # ±4wk	EoS**/Early Withdrawal
Pregnancy test [17]		X	X			X		
Adverse events [22]	X							
Physical Exam	X yearly							
**BIOMARKERS**								
PERIPHERAL BLOOD								
Local Assessment- Blood for Tcell Subset (CD3+CD4+, CD3+CD8+) Analysis [20]	X every 6 months							
Blood for CAR T Cells persistance, PCR [21]	X as clinically indicated							
RCR		X	X		X	X	X yearly	X
Insertional Mutagenesis		X	X		X	X	X	X

d = days; EoS = end of study; M = month; RCR = replication competent retrovirus; wk = weeks.

TABLE OF CONTENTS

PROTOCOL SYNOPSIS **6**
SCHEDULE OF ASSESSMENTS **14**
TABLE OF CONTENTS **23**
LIST OF APPENDICES **28**
LIST OF TABLES **28**
LIST OF FIGURES **29**
1 INTRODUCTION **34**
1.1 T CELL LYMPHOMAS 34
1.2 T CELL RECEPTOR BETA CONSTANT 1 AS A TARGET FOR THERAPY 35
1.3 CLINICAL EXPERIENCE WITH CAR T THERAPIES IN T CELL LYMPHOMA 35
1.4 DESCRIPTION OF AUTO4 – RQR8/ANTI-TRBC1 CAR POSITIVE T CELLS 36
1.5 SUMMARY OF NONCLINICAL STUDIES 37
1.5.1 Estimating Safety Concerns of Targeting T Cells 38
1.6 SUMMARY OF CLINICAL STUDIES WITH AUTO4 39
1.7 OVERALL RATIONALE FOR THE STUDY 39
2 STUDY OBJECTIVES AND ENDPOINTS **40**
2.1 PRIMARY OBJECTIVES AND ENDPOINTS 40
2.2 SECONDARY OBJECTIVES AND ENDPOINTS 40
2.3 EXPLORATORY OBJECTIVES 40
3 STUDY DESIGN **42**
3.1 STUDY OVERVIEW 42
3.2 PHASE I DOSE ESCALATION RULES AND ROLLING SIX DESIGN 43
3.3 PLANNED DOSE LEVELS 45
3.4 PHASE II DOSE EXPANSION 46
3.5 DOSE LIMITING TOXICITY 46
3.6 SAFETY STOPPING CRITERIA FOR THE CLINICAL TRIAL 47
3.7 STUDY DURATION 48
3.8 NUMBER OF PATIENTS 48
4 SCIENTIFIC RATIONALE FOR STUDY **49**
4.1 DESIGN RATIONALE 49
4.2 POPULATION RATIONALE 49
4.2.1 Current Therapeutical Options for this Population and Rationale for Inclusion 50
4.3 STARTING DOSE RATIONALE 51
4.4 RATIONALE FOR MAXIMUM ADMINISTERED DOSE (MAD) 52
4.5 RATIONALE FOR DOSING SCHEDULE 52

4.6 FLUDARABINE AND CYCLOPHOSPHAMIDE PRE-CONDITIONING RATIONALE 53
4.7 BIOMARKER COLLECTION AND ASSESSMENT RATIONALE 53
5 RISKS AND MITIGATION STRATEGY 54
5.1 TUMOUR TISSUE BIOPSY 54
5.2 LEUKAPHERESIS 54
5.3 PRE-CONDITIONING CHEMOTHERAPY 54
5.4 AUTO4 INFUSION 55
5.5 UNKNOWN LONG-TERM RISKS OF GENE THERAPY 58
6 PATIENT POPULATION 59
6.1 INCLUSION CRITERIA 59
6.2 EXCLUSION CRITERIA 60
7 ADVANCED THERAPY INVESTIGATIONAL MEDICINAL PRODUCT: AUTO4 62
7.1 AUTO4 DESCRIPTION 62
7.2 AUTO4 MANUFACTURING 62
7.3 PACKAGING AND LABELLING 62
7.4 SUPPLY AND STORAGE 63
7.5 ACCOUNTABILITY AND DESTRUCTION 63
8 STUDY PROCEDURES AND TREATMENT 64
8.1 CONSENT, SCREENING, TISSUE BIOPSY AND REGISTRATION 64
8.1.1 Consent processes 64
8.1.2 Patient Registration 64
8.1.3 Patient Screening for TRBC1 Positive T-NHL 65
8.1.4 Tumour Tissue Provision 65
8.1.5 TRBC1 Immunohistochemistry Assay Used to Select TRBC1 Positive Patients for Entry 66
8.1.6 Patient Screening to the Main Study 66
8.1.7 Infectious Disease Screening 67
8.2 LEUKAPHERESIS FOR AUTO4 MANUFACTURING 67
8.2.1 Leukapheresis for Immune Reconstitution (Rescue Treatment Storage) 68
8.3 PRE-CONDITIONING CHEMOTHERAPY 68
8.3.1 Pre-conditioning Chemotherapy Dose and Regimen 69
8.3.2 Supply of Fludarabine and Cyclophosphamide 69
8.3.3 Accountability of Fludarabine and Cyclophosphamide 69
8.4 AUTO4 TREATMENT AND PATIENT MONITORING 69
8.4.1 Assessment Prior to AUTO4 Infusion 70
8.4.2 AUTO4 Administration 70
8.4.3 Monitoring During and After Drug Administration 71
8.4.4 Monitoring for Infections 71

8.4.5 Dose Delay of Pre-conditioning and AUTO4 Treatment 72
8.4.6 Interruption of Infusion 72
8.4.7 Duration of Treatment 72
8.4.8 Re-treatment of Patients 72
8.5 FOLLOW-UP PHASE 73
8.6 LONG-TERM FOLLOW-UP 75
9 STUDY ASSESSMENTS 76
9.1 DEMOGRAPHIC AND BASELINE ASSESSMENTS 76
9.1.1 Demographic Data and Baseline Variables (Screen 1) 76
9.1.2 Tumour Tissue Biopsy and TRBC1 Status at Baseline (During Screen 1) 76
9.1.3 Efficacy Scans at Baseline (Screen 2) 76
9.2 SAFETY EVALUATIONS 77
9.2.1 Adverse Events and Toxicity 77
9.2.2 Clinical Laboratory Tests 77
9.2.3 12-lead Electrocardiogram 78
9.2.4 Echocardiogram or Multiple Gated Acquisition Cardiac Scan 79
9.2.5 Vital Signs 79
9.2.6 Physical Examination 79
9.2.7 Concomitant Medications 79
9.2.8 ECOG Performance Status 79
9.3 PHARMACOKINETICS, PHARMACODYNAMICS AND BIOMARKER EVALUATION 79
9.3.1 Evaluation of AUTO4 Persistence in Peripheral Blood 80
9.3.2 Evaluation of RCR in Peripheral Blood 80
9.3.3 TRBC1 Positive T Cell Aplasia 80
9.3.4 Insertional Mutagenesis 80
9.3.5 Exploratory Biomarker Assessments 80
9.3.6 Immunogenicity Analysis 81
9.4 EFFICACY EVALUATION 81
9.4.1 Radiographic Image Assessments (CT/MRI) 82
9.4.2 Positron Emission Tomography Scan 82
9.4.3 Efficacy Criteria 82
9.5 BLOOD VOLUME COLLECTIONS 83
10 GUIDELINES FOR PREVENTION, MONITORING, AND MANAGEMENT OF ADVERSE EVENTS 85
10.1 GENERAL SUPPORTIVE CARE GUIDELINES FOR PATIENTS RECEIVING CAR T CELL THERAPY 85
10.2 PRE- AND POST-INFUSION SUPPORTIVE THERAPY 86
10.3 MANAGEMENT OF INFUSION RELATED REACTIONS 87

10.4 GRADING AND MANAGEMENT OF CYTOKINE RELEASE SYNDROME (CRS) 89
10.5 MANAGEMENT MACROPHAGE ACTIVATION SYNDROME (MAS) 96
10.6 GRADING AND MANAGEMENT OF NEUROTOXICITY (ICANS) 96
10.7 MANAGEMENT OF TUMOUR LYSIS SYNDROME 101
10.8 T CELL APLASIA 102
10.9 MANAGEMENT OF IMMUNE-RELATED ADVERSE EVENTS DUE TO ON-TARGET BUT OFF-TUMOUR TOXICITY 102
11 CONCOMITANT MEDICATIONS AND THERAPIES 103
11.1 BRIDGING THERAPY 103
11.2 ALLOWED CONCOMITTANT MEDICATIONS/THERAPIES 103
11.3 PROHIBITED AND CAUTIONARY THERAPIES 104
11.4 OVERDOSAGE 104
11.5 DIETARY AND LIFESTYLE RESTRICTIONS 105
12 SAFETY AND PHARMACOVIGILANCE 106
12.1 DEFINITIONS 106
12.1.1 Definition of an Adverse Event 106
12.1.2 Definition of an Adverse Reaction 106
12.1.3 Definition of a Serious Adverse Event 106
12.1.4 Adverse Events of Special Interest 107
12.1.5 Disease Progression 107
12.2 ASSESSMENT OF ADVERSE EVENTS 108
12.2.1 Severity of Adverse Events 108
12.2.2 Relationship of Adverse Events to Treatment 108
12.3 REPORTING PROCEDURES 109
12.3.1 All Adverse Events 109
12.3.2 Adverse Events Associated with Laboratory Values 111
12.3.3 Adverse Events Associated with Cytokine Release Syndrome (CRS) 111
12.3.4 Adverse Events Associated with CAR-T Neurotoxicity (ICANS) 111
12.3.5 Serious Adverse Events Reporting 112
12.3.6 Adverse Events of Special Interest Reporting 113
12.3.7 Pregnancy Reporting and Management 113
12.3.8 Overdose Reporting and Dosing Errors 114
13 OVERSIGHT COMMITTEES 116
13.1 SAFETY EVALUATION COMMITTEE 116
13.2 INDEPENDENT DATA MONITORING COMMITTEE (IDMC) 116
14 STATISTICS 118
14.1 SAMPLE SIZE ESTIMATION 118
14.2 DESCRIPTION OF ANALYSIS DATASETS 118
14.2.1 Full Analysis Set 118

14.2.2 Safety Analysis Set for the Study and for Study Treatment (Pre-conditioning and AUTO4) ... 118
14.2.3 Efficacy Analysis Set ... 118
14.3 STATISTICAL ANALYSES AND METHODS ... 119
14.3.1 Primary Endpoints ... 119
14.3.2 Secondary Endpoints ... 120
14.3.3 Exploratory Objectives ... 121
14.3.4 Immunogenicity Data ... 122
14.3.5 Other Analyses ... 122
14.3.6 Interim Analyses ... 122
14.3.7 Final Analysis ... 122
15 STUDY COMPLETION, PREMATURE DISCONTINUATION AND WITHDRAWAL FROM AUTO4-TL1 ... 123
15.1 COMPLETION OF STUDY ... 123
15.2 PREMATURE DISCONTINUATION FROM AUTO4-TL1 ... **ERROR! BOOKMARK NOT DEFINED.**
15.3 PATIENT WITHDRAWAL FROM AUTO4-TL1 ... 123
15.3.1 Procedures for Handling Premature Withdrawal from the Study ... 123
15.4 REPLACEMENT POLICY ... 124
16 END OF TRIAL/TERMINATION BY SPONSOR ... 125
17 ETHICAL AND REGULATORY CONSIDERATIONS ... 126
17.1 ETHICS COMMITTEE REVIEW AND APPROVAL ... 126
17.2 REGULATORY AUTHORITY REVIEW AND APPROVAL ... 126
17.3 INVESTIGATOR RESPONSIBILITIES ... 126
17.3.1 Overall Responsibilities ... 126
17.3.2 Site Review ... 126
17.3.3 Informed Consent ... 127
17.3.4 Delegation of Investigator Duties ... 127
17.3.5 Communication with Independent Ethics Committee/Institutional Review Board ... 127
17.3.6 Confidentiality of Trial Documents and Patient Records ... 128
17.4 LONG-TERM RETENTION OF SAMPLES FOR ADDITIONAL FUTURE RESEARCH ... 128
18 ADMINISTRATIVE REQUIREMENTS ... 130
18.1 DATA QUALITY CONTROL AND ASSURANCE ... 130
18.1.1 Compliance with the Protocol and Protocol Revisions ... 130
18.1.2 Protocol Violations and Deviations ... 130
18.1.3 Monitoring ... 130
18.1.4 Audits and Site Inspections ... 131
18.2 DATA HANDLING AND RECORD KEEPING ... 131
18.2.1 Data Management ... 131

18.2.2 Study Documentation and Retention of Records....132
18.3 CLINICAL TRIAL AGREEMENT, FINANCE, AND INSURANCE....132
19 PUBLICATION OF DATA AND PROTECTION OF TRADE SECRETS....133
20 REFERENCES....134
21 APPENDICES....142

LIST OF APPENDICES

Appendix 1: Patients with T-NHL will be Evaluated Using Response Criteria for Non-Hodgkin Lymphoma for Documenting Disease Response....142
Appendix 2: Eastern Cooperative Oncology Group Performance Status Score....145
Appendix 3: Highly Effective and Acceptable Methods of Birth Control....146

LIST OF TABLES

Table 1: Primary Objectives and Endpoints for Phase I....40
Table 2: Primary Objectives and Endpoints for Phase II....40
Table 3: Secondary Objectives and Endpoints for Phase I and II....40
Table 4: Dose Escalation Decision Rules....45
Table 5: Planned Dose Levels and Treatment Cohorts....45
Table 6: Leukapheresis – Risks and Mitigation Strategy....54
Table 7: Pre-conditioning Chemotherapy – Risks and Mitigation Strategy....55
Table 8: AUTO4 Infusion – Risks and Mitigation Strategy....56
Table 9: Distribution of TRBJ and TRBC in Tonsil TCR-β Transcripts....66
Table 10: Infectious Disease Screening....67
Table 11: Clinical Laboratory Tests....78
Table 12: General Supportive Care Guidelines for Patients Receiving CAR T Cell Therapy....85
Table 13: Pre- and Post-infusion Medications....87
Table 14: Guidelines for the Management of Infusion-related Reactions....88
Table 15: Severity Grading of Cytokine Release Syndrome (ASTCT/ASBMT CRS Consensus Grading and CTCAE Version 5.0) **(Lee et al. 2019)**....90
Table 16: Management of Cytokine Release Syndrome....91
Table 17: Pharmacologic Management of CRS and ICANS....93
Table 18: Assessment & Grading of ICANS....98
Table 19: Immune Effector Cell-associated Encephalopathy (ICE) Scale....98
Table 20: Management of ICANS....99
Table 21: Recommendations for the Management of Status Epilepticus After CAR T Cell Therapy....100
Table 22: Recommendation for Management of Raised Intracranial Pressure (ICP) After CAR T Cell Therapy....101

Table 23: Severity Grading of AEs Not Listed on the NCI CTCAE Grading System ... 108
Table 24: Reporting Period for All AEs ... 110

LIST OF FIGURES

Figure 1: Transgenic Proteins Expressed on Autologous T Cells (AUTO4) ... 37
Figure 2: Proposed Dose Escalation and Dose Expansion Phases ... 42
Figure 3: Overview of the Stages of the Study ... 43
Figure 4: Cytokine Release Syndrome Management Overview ... 92

LIST OF ABBREVIATIONS

Abbreviation	Definition
AE	Adverse event
AESI	Adverse event of special interest
ALK	Anaplastic lymphoma kinase
ALL	Acute lymphoblastic leukaemia
ALT	Alanine aminotransferase
ANC	Absolute Neutrophil Count
ASBMT	American Society for Blood and Marrow Transplantation
AST	Aspartate aminotransferase
ASTCT	American Society for Transplantation and Cellular Therapy
ATIMP	Advanced therapy investigational medicinal product
aTRBC	Anti-T cell receptor beta constant
aTRBC -CAR	Anti-T cell receptor beta constant 1 chimeric antigen receptor
BCMA	B cell maturation antigen
B-NHL	B cell non-Hodgkin lymphoma
CAR	Chimeric antigen receptor
CD3, 19, 20, 28, 68, 134	Cluster of differentiation 3, 19, 20, 28, 68 134
CHIM	Cell handling instruction manual
CHOP	Cyclophosphamide, Hydroxydoxorubicin, Oncovin, Prednisone
CMR	Complete Metabolic Response
CMV	Cytomegalovirus
CNS	Central nervous system
CPK	Creatine phosphokinase
CR	Complete response
Cru	Complete response unconfirmed
CRS	Cytokine release syndrome
CT	Computed tomography
CTCAE	Common Terminology Criteria for Adverse Events
CTLA	Cytotoxic T lymphocyte-associated protein
CY	Cyclophosphamide
DFS	Disease-free survival
DHAP	Dexamethasone, High-dose Ara-C, Platinum
DIC	Disseminated Intravascular Coagulation
DLBCL	Diffuse Large B Cell Lymphoma
DLT	Dose limiting toxicity

Abbreviation	Definition
DMSO	Dimethyl sulphoxide
DNA	Deoxyribonucleic acid
DOR	Duration of response
EBV	Epstein Barr Virus
ECG	Electrocardiogram
ECHO	Echocardiogram
ECOG	Eastern Cooperative Oncology Group
eCRF	Electronic Case Report Form
EDC	Electronic data capture
EDTA	Ethylenediaminetetraacetic acid
EFS	Event free survival
ESHAP	Etoposide, Methylprednisolone, Ara-C, Platinum
EU	European Union
EudraCT	European Clinical Trials Database
FDA	Food and Drug Administration
FDG	Fluorodeoxyglucose
FFPE	Formalin fixed paraffin embedded
FIH	First-in-human
FLU	Fludarabine
FLU-CY	Fludarabine and cyclophosphamide
GCP	Good Clinical Practice
G-CSF	Granulocyte-Colony Stimulating Factor
GI	Gastrointestinal
GM CSF	Granulocyte-Macrophage Colony-Stimulating Factors
GMP	Good Manufacturing Practice
HDAC	Histone deacetylase
HIV	Human immunodeficiency virus
HLH	Haemophagocytic lymphohistiocytosis
HSCT	Hematopoietic stem cell transplantation
HTLV	Human T cell lymphotropic virus
IB	Investigator’s Brochure
ICANS	Immune Effector Cell-associated Neurotoxicity Syndrome
ICE	Ifosfamide, Carboplatin, Etoposide
ICF	Informed Consent Form
ICH	International Conference on Harmonisation

Abbreviation	Definition
IDMC	Independent Data Monitoring Committee
IEC	Independent Ethics Committee
IFN	Interferon
IHC	Immunohistochemistry
IL	Interleukin
IMPD	Investigational Medicinal Product Dossier
irAE	Immune-related adverse event
IRB	Institutional Review Board
i.v.	Intravenous(ly)
JCV	John Cunningham Virus
JOVI-1	JOVI-1 assay
LDH	Lactate dehydrogenase
Ldi	Longest transverse diameter of a lesion
LVEF	Left ventricular ejection fraction
MAD	Maximum administered dose
MAS	Macrophage activation syndrome
MLV	Murine Leukaemia Virus
MRI	Magnetic resonance imaging
MTD	Maximum tolerated dose
MUGA	Multiple gated acquisition
NCI	National Cancer Institute
NHL	Non-Hodgkin lymphoma
NGS	Next-Generation Sequencing
NOS	Not otherwise specified
NSAID	Non-steroidal anti-inflammatory drugs
OR	Overall response
ORR	Overall response rate
OS	Overall survival
OX40	Also known as tumour necrosis factor receptor superfamily 4 [TNFRSF4] and cluster of differentiation 134 [CD134])
PBMCs	Peripheral blood mononuclear cells
PCR	Polymerase chain reaction
PD1	Programmed cell death protein 1
PD-L1	Programmed death-ligand 1
PET	Positron emission tomography
PFS	Progression-free survival

Abbreviation	Definition
PI	Prescribing information
PIS	Patient Information Sheet
PPD	Perpendicular diameter (cross product of the LDi and perpendicular diameter)
PR	Partial response
PTCL	Peripheral T cell lymphoma
PTT	Partial thromboplastin time
q.a.d	Quaque altera die (every other day)
QP	Qualified Person
RCR	Replication competent retrovirus
RP2D	Recommended Phase II dose
RQR8	Ritux-QBEND/10-Ritux-CD8 sort-suicide gene generated as a marker/suicide gene for T cells
RSI	Reference Safety Information
SAE	Serious adverse event
scFv	Single chain variable fragment
SCT	Stem cell transplant/transplantation
SEC	Safety Evaluation Committee
SmPC	Summary of Product Characteristics
SPD	Sum of the product of the perpendicular diameters for multiple lesions
SUSAR	Suspected unexpected serious adverse reaction
TBD	To be determined
TCR	T cell Receptor
TLS	Tumour lysis syndrome
TNF	Tumour necrosis factor
TRBC	T cell receptor beta constant
T-NHL	T cell non-Hodgkin Lymphoma
ULN	Upper limit of normal
US	United States

1 INTRODUCTION

AUTO4 is an autologous, cell and gene therapy investigational medicinal product that is manufactured by genetic modification of the patient's own T cells by *ex vivo* transduction with a murine leukaemia virus (MLV)-derived retroviral vector that has been engineered to stably deliver the RQR8 and aTRBC1-CAR genes. The active substance is the genetically modified (RQR8/aTRBC1-CAR positive) T cells. RQR8 is a Ritux-QBEnd/10-Ritux-cluster of differentiation 8 (CD8) sort-suicide gene generated as a marker/suicide gene for T cells combining target epitopes from both CD34 and CD20 antigens.

T cell receptor (TCR) beta constant region (TRBC) 1 and 2 are isoforms of the TCR and all T cells, normal and malignant, express either TRBC1 or TRBC2. Malignant T cells are clonal and the majority express exclusively either entirely TRBC1 or TRBC2. Normal T cells consist of a mixture of T cells individually expressing either TRBC1 or TRBC2. In this study, Autolus Limited (Autolus) plans to recruit patients with TRBC1 positive T cell lymphomas. Patients will receive autologous CAR T cells directed against TRBC1. These CAR T cells will target the lymphoma in its entirety as well as a proportion of normal T cells (i.e. those which are TRBC1 T cells) with the aim of maintaining immunity due to preservation of TRBC2 positive cells.

The objectives of this Phase I/II first-in-human (FIH) study (AUTO4-TL1) are to: (1) evaluate the safety profile, (2) establish a recommended Phase II dose (RP2D), and (3) in Phase II determine the anti-lymphoma activity of AUTO4 in patients with relapsed or refractory T cell non-Hodgkin Lymphoma (T-NHL). AUTO4 will be administered following lymphodepletion with fludarabine (FLU) and cyclophosphamide (CY).

A summary of the nonclinical characteristics of AUTO4 are described below in Section 1.5. For the most comprehensive nonclinical information, refer to the Investigator's Brochure (IB). Details regarding AUTO4 administration and laboratory procedures are provided in the cell handling manual (CHM) and laboratory manual, respectively.

1.1 T CELL LYMPHOMAS

T cell lymphoma is a rare and heterogeneous form of NHL with low incidence rates in Europe and the United States (Bellei et al. 2012). T cell lymphomas represent approximately 3% to 4% of all haematological malignancies and 10% to 20% of non-Hodgkin lymphomas (NHL) (Ascani et al. 1997) and (Anderson et al. 1998). While T cell lymphoma is the smaller subset of all lymphomas (e.g., compared to B cell lymphomas), this is an aggressive disease with a very poor prognosis for T cell lymphoma patients. In the United Kingdom, there are about 650 T cell lymphoma cases expected per annum and the incidence rates have shown modest increases in the past decades (NICE 2016). The first line treatment for the more common T cell lymphoma subtypes, i.e. peripheral T cell lymphoma, peripheral T cell lymphoma not otherwise specified (NOS) and angioimmunoblastic T cell lymphoma, consists of combination chemotherapy, such as cyclophosphamide, doxorubicin, vincristine and prednisolone, but is not without toxicity concerns. Little is understood in terms of treatment guidance for the lesser T cell subtypes and they remain without clear treatment guidelines. A large proportion of T cell lymphoma patients are refractory to or relapse following standard therapies and there remains a requirement to develop an effective therapy for this unmet need.

Unlike B cell lymphomas, patients with T cell lymphomas have not benefited from advances in immunotherapeutic approaches such as BiTE or CAR T cell therapies. This is mainly due to the lack of therapeutic development in T cell lymphomas to identify a suitable target antigen(s)

to discriminate malignant T cells from normal T cells. While a similar problem exists with B cell lymphomas, targeting a pan B cell antigen is an acceptable strategy, as the concomitant depletion of the normal B cell compartment is well tolerated and certain targeted approaches may be ameliorated via the i.v. administration of immunoglobulin. In contrast, targeting a pan T cell antigen would result in severe immunosuppression, where there is currently no available rescue medication. Autolus has developed an innovative ATIMP, AUTO4, which uses a novel targeting approach to attempt to address this issue.

1.2 T CELL RECEPTOR BETA CONSTANT 1 AS A TARGET FOR THERAPY

During T cell development, each T cell generates a unique T cell receptor by rearrangement of the alpha and beta chains. Each beta chain also includes a constant domain which is not involved in antigen recognition. A facet of TCR gene-rearrangement is that the coding sequence for the T cell receptor beta constant region is duplicated (TRBC1 and TRBC2) (Maciocia et al. 2016). Hence, in a manner analogous to antibody light chain selection, a mature T cell is highly likely to express either TRBC1 or TRBC2. Normal T cells (including T cell subsets) comprise a mixture of T cells (approximately 40%, 60% mix) individually expressing either TRBC1 or TRBC2. Natural immunity including antiviral immunity is also distributed across the 2 populations. T cell lymphomas on the other hand are clonal tumours and are either entirely TRBC1 or TRBC2 positive.

With this present study, Autolus plans to recruit patients with the most common T cell lymphomas that are also TRBC1 positive.

Patients will receive autologous CAR T cells directed to TRBC1. These CAR T cells will target the lymphoma in its entirety as well as a proportion of normal T cells (i.e. those which are TRBC1 T cells). It is hypothesised untargeted (TRBC2) normal T cells will constitute sufficient immunity to protect the patient from opportunistic infections.

1.3 CLINICAL EXPERIENCE WITH CAR T THERAPIES IN T CELL LYMPHOMA

To date, clinical experience with CAR-T therapy in NHL has mainly been limited to B cell lymphomas and there is little clinical experience with CAR T therapies in T cell lymphomas. One recent Phase 1 study with preliminary data demonstrated a tolerable safety profile and promising clinical efficacy with a first-in-class autologous CD7-CAR-T cell product in relapsed and refractory T-Lymphoblastic Leukemia/Lymphoma (Zhang et al 2020). In B cell lymphomas, several clinical trials have been initiated targeting CD19 demonstrating efficacy with overall response rate (ORR) ranging from 50% to 86% (Brentjens et al. 2003, Cooper et al. 2003, Hoyos et al. 2010, Jensen et al. 2010, Kochenderfer et al. 2010, Porter et al. 2011, Savoldo et al. 2011, Kochenderfer et al. 2012). While the CD19 CAR construct used by each institution differs in several respects, including CAR design, T cell activation, transduction methods, and cell doses, the net observed effects remain consistent with regards to the safety and efficacy of CD19-targeted CAR T therapies. The major toxicities observed after CD19 CAR positive T cell transfusion are B cell depletion, cytokine release syndrome (CRS) and neurotoxicity. Management of CRS has significantly improved with the use of guidelines and early use of tocilizumab. Neurotoxicity is the most challenging clinical problem. Close monitoring, use of steroids and other immunosuppressants have improved the management of neurotoxicity; however, further improvements are still needed (Brudno and Kochenderfer 2016).

CAR T immunotherapy for B cell malignancies typically targets pan B cell antigens, resulting in B cell aplasia. However, similar approaches of targeting pan T cell antigens resulting in elimination of malignant and non-malignant T cells is challenging as T cell aplasia will result in significant morbidity and mortality, necessitating an allogenic stem cell transplantation.

Considering the significant unmet need in these patients and in the absence of truly tumour-specific targets applicable to a high proportion of patients, the alternate approach of targeting an antigen expressed in normal T cells followed by allogenic stem cell transplantation is considered a viable option. Pre-clinical studies using an anti-CD5 CAR have shown select killing of CD5+ T cell targets both *in vitro* and *in vivo* (Mamonkin et al. 2015). A clinical trial using anti-CD5 CAR T cells is listed on clinicaltrials.gov, but to date has yet to start recruitment (NCT03081910). As expected, this study only recruits patients suitable for consideration for allogeneic hematopoietic stem cell transplantation (HSCT) with an identified donor, contingent on entering remission.

An alternate approach is using CAR T cells to target CD4, which is expressed on approximately 40% of cases of peripheral T cell lymphoma (PTCL) or T cell acute lymphoblastic leukemia (T-ALL) as well the majority of peripheral T cells. Whilst pre-clinical studies have demonstrated CD4 CAR T mediated killing of CD4+ tumour (Pinz et al. 2016), this approach will also lead to aplasia of the entire CD4+ compartment likely compromising normal T cell immunity necessitating an allogeneic HSCT. Another approach targets the pan-T cell antigen CD3 with chimeric antigen receptors (Chen et al. 2016), but utilises natural killer cells as the effector population. Efficacy was shown *in vitro* and *in vivo* against cell lines and primary tumour cells187 but again the disadvantage of this approach would be complete T cell aplasia, necessitating rescue transplant.

The proposed approach with AUTO4 would not necessitate a rescue transplant as it would only result in partial depletion of the normal T cell compartment and preservation of natural immunity (Brudno and Kochenderfer 2016)

1.4 DESCRIPTION OF AUTO4 – RQR8/ANTI-TRBC1 CAR POSITIVE T CELLS

The AUTO4 therapeutic comprises of enriched autologous T cells retrovirally transduced to express an anti-TRBC1 specific CAR, as well as the safety switch RQR8. The active substance, which consists of the genetically modified T cells, will be referred to as RQR8/aTRBC1-CAR positive T cells. AUTO4 also contains non-transduced autologous lymphocytes. This is a second-generation CAR which has a 41BB-CD3ζ endodomain. The retroviral cassette co-expresses the RQR8 safety switch with the CAR. RQR8 expression renders the T cells susceptible to rituximab-mediated depletion of AUTO4. A schematic representation of the transgenic expression of AUTO4 is provided in Figure 1.

Figure 1: Transgenic Proteins Expressed on Autologous T Cells (AUTO4)

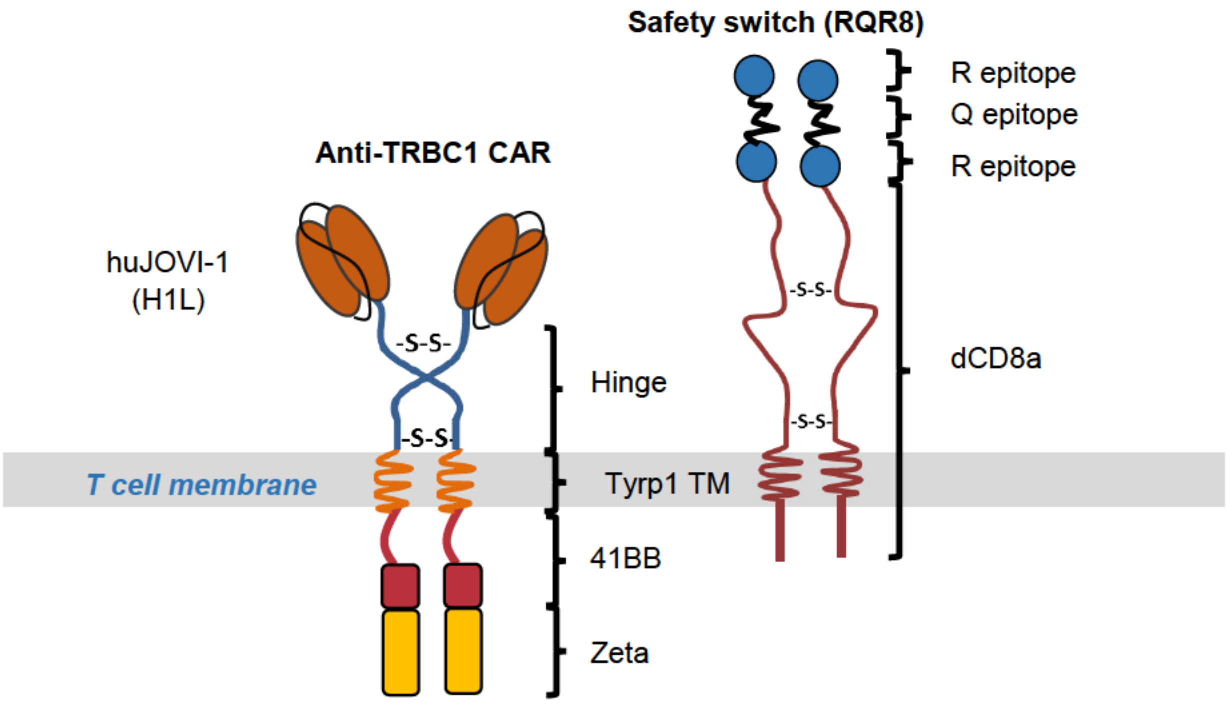

Structure of transgenes: The anti-TRBC1 CAR is a type I transmembrane protein. The extracellular amino terminus is composed of a single chain variable fragment (scFv) derived from a humanised version of the JOVI-1 antibody, called H1L, is fused to the hinge region of IgG1. This is in turn connected to a human Tyrosinase-related protein-1 (Tyrp-1) transmembrane domain fused to the costimulatory endodomains composed 41BB (CD137) and CD3-ζ (CD247). RQR8 is also a type I transmembrane protein. The amino terminus is a composite of two copies of a disulphide constrained loop closely resembling the main extracellular loop of CD20. These flank a portion of CD34 which binds the QBEnd/10 antibody. The amino-terminus is connected to the CD8α stalk, transmembrane and anchor.

1.5 SUMMARY OF NONCLINICAL STUDIES

Further details regarding AUTO4 nonclinical studies can be found in the IB. A package of nonclinical work has been completed that demonstrates the *in vitro* and *in vivo* specificity and potency of AUTO4 to target TRBC1-expressing cells.

Due to the restrictions of testing CAR T cells in conventional toxicological, safety and efficacy studies, the nonclinical programme has focused on verifying specificity and potency of anti-tumour activity. Murine anti-TRBC1 antibody (clone, JOVI-1) derived single chain variable fragment (scFv) was humanised by Complementary Determining Region grafting and derived variants were shown to have similar binding affinities to the parent scFv. The specificity and functionality of the selected humanised aTRBC1-CAR was established *in vitro* using human lymphoblastoid cell lines and primary human cells. The *in vivo* efficacy of humanised RQR8/aTRBC1-CAR positive T cells was established in a tumour xenograft mouse model using immune-compromised NSG™ (non-obese [NOD] severe combined immunodeficiency [Scid] Gamma) mice. The humanised aTRBC1 does not cross the species barrier from human to mouse which restricts the ability to assess off-tumour effects in this model. However, a human tissue cross-reactivity study was used to assess potential off-target activity and no unexpected cross-reactivity was observed beyond the expected tissue distribution of T lymphocyte.

The *in vivo* function of the RQR8 safety switch has previously been demonstrated using a mouse model (Bellei et al. 2012). Additionally, the functionality of RQR8 combined with the TRBC1-CAR has been demonstrated *in vitro*.

In summary, it has been demonstrated both *in vitro* and *in vivo* that AUTO4 aTRBC1-CAR positive T cells can effectively eliminate TRBC1-expressing tumour cells. Nonclinical studies suggest that no off-target toxicity is anticipated.

1.5.1 Estimating Safety Concerns of Targeting T Cells

Comparing the cellular distribution and frequency of B cells and T cells may help estimate the relative potential for on-target off-tumour toxicities due to cytolysis of the normal cell population. The distribution of T cells and B cells within human organs is generally similar. The major distribution of both T and B cells is located within most lymphoid organs (lymph nodes, spleen, tonsils, thymus, bone marrow and lymphoid-associated tissue). T and B cells are also abundant in the blood, as well as part of the cellular infiltrate of multiple organs such as heart, lung, liver, various glandular tissue, and reproductive tissues.

The tissue cross reactivity studies have noted T cell, but not B cell, distribution in the cerebral cortex of the brain, blood vessels, kidney, pituitary gland, placenta, skeletal muscle, skin, testes and thyroid. Neither B nor T cells were found in the cerebellum of the brain, spinal cord, eye and nerve. A comparison of the relative numbers of T and B cells from normal tissues has been made (Sathaliyawala et al. 2013). The frequency of B and T cells from blood and eight different healthy tissues, including multiple lymphoid tissues (spleen, inguinal, mesenteric and bronchial/lung-draining lymph nodes) and mucosal tissues including the lung, small intestine regions (jejunum, ileum) and colon tissues (blood, spleen, inguinal lymph node) was compared. The frequency of T cells outnumbered B cells in all sites except the spleen - by 2 to 4-fold in blood and LNs, 5 to 8-fold in ileum and colon and >15 to 20-fold in the lung and jejunum. A similar number of B and T cells were found in the spleen. In summary, whilst there is a slightly higher cellular distribution of T cells across major organs, the frequency of T cells distribution within some organs and blood are significantly higher compared to B cells. Whilst targeting TRBC1 cells will only ablate approximately one-third of normal T cells, there is likely to be more cytolysis of normal T cells within the lung and jejunum compared to targeting B cells. However, a significant number of these cells would have been depleted by the pre-conditioning chemotherapy.

An additional observations is the target antigen density on normal cells, the antigen density of TCR is about 100,000 copies per T cell, (Schodin BA et al., 1996) which is 5 times higher than the antigen density of CD19 on B cells. However, the CAR T cell response against target cell as well as the production of interleukin 2 (IL-2), interferon-g (IFN-γ) and granzyme-b follows a sigmoid curve, and higher antigen density is likely to generate a saturated response (Nguyen S et al., 2016).

Considering the current experience with CD19 CARs in acute lymphoblastic leukaemia (ALL), diffuse large B-cell lymphoma and chronic lymphocytic leukaemia involving patients with significant amounts of antigen-positive disease in various organs, including brain, the acute toxicity is unlikely to be significantly worse than that observed with CD19 CARs and, most probably manageable with current safety management guidelines. In addition, starting at a low CAR T cell dose is likely to add to the safety.

1.6 SUMMARY OF CLINICAL STUDIES WITH AUTO4

The current study will be a first-in-human (FIH) study of the ATIMP, AUTO4, in patients with T cell non-Hodgkin lymphoma (T-NHL). Therefore, no clinical experience is available at the time of the initial application.

1.7 OVERALL RATIONALE FOR THE STUDY

Outcomes for patients with T cell lymphoma are generally poor. The patients relapsing after one line of therapy or those with refractory disease have worse outcome and the disease remains incurable with the currently established therapy modalities. A considerable number of T cell lymphoma patients will ultimately experience a final tumour relapse without an additional, effective treatment option. Novel immunotherapies such as CAR T cell therapies hold promise for significant improvement in the overall outcome of patients with T cell lymphoma. CD19 CAR positive T cells in clinical development for the treatment of B cell lymphoma have demonstrated significant efficacy in clinical trials. Similar approaches of pan T cell depletion are not possible for T cell malignancies, as survival without T cells is likely to be poor. Selective depletion of only TRBC1 positive T cells offers a unique way to address this issue without making the patient T cell aplastic or compromising patients' T cell immunity. AUTO4 offers the potential to provide a therapeutic option for patients with different T cell lymphomas that are TRBC1 positive.

Further, AUTO4 has also been engineered to be effective and safe with the incorporation of both 41BB-ζ co-stimulatory signals and incorporation of a RQR8 safety switch, which would allow for selective depletion of AUTO4 if necessary. Additionally, the storage of unprocessed apheresate (where possible at the site) as backup, to be used for immune re-constitution if necessary also adds to the safety of the current trial. This FIH, Phase I/II study will assess the safety and activity of AUTO4, in patients with relapsed or refractory select T cell lymphoma.

2 STUDY OBJECTIVES AND ENDPOINTS

2.1 PRIMARY OBJECTIVES AND ENDPOINTS

Primary objectives and endpoints for Phase I and Phase II of the study are presented in Table 1 and Table 2, respectively.

Table 1: Primary Objectives and Endpoints for Phase I

Objectives	Endpoints
To assess the safety and tolerability of AUTO4 administration.	Incidence of Grade 3 to 5 toxicity occurring within 60 days of AUTO4 infusion.
To identify the RP2D and MTD, if an MTD exists, of AUTO4.	Frequency of DLT of AUTO4 within 28 days of AUTO4 infusion.

DLT = dose limiting toxicity; MTD = maximum tolerated dose; RP2D = recommended Phase II dose

Table 2: Primary Objectives and Endpoints for Phase II

Objectives	Endpoints
To assess the clinical activity of AUTO4 when administered at the RP2D	Overall response (CR+PR) rate post AUTO4 infusion

CR = complete response; PR = partial response

2.2 SECONDARY OBJECTIVES AND ENDPOINTS

Secondary objectives and endpoints are presented in Table 3.

Table 3: Secondary Objectives and Endpoints for Phase I and II

Objectives	Endpoints
To assess the overall safety and tolerability of AUTO4.	Frequency and severity of all AEs and SAEs
	Incidence and severity of opportunistic infections following AUTO4 infusion.
To evaluate the feasibility of generating the ATIMP, AUTO4.	Proportion of patients (who are TRBC1 positive and undergo leukapheresis), for whom an AUTO4 product can be generated (feasibility).
To evaluate the overall clinical efficacy of AUTO4.	Determine the CR rate following treatment with AUTO4.<br>Evaluate clinical outcomes including DOR, DFS, PFS, OS, time to response (PR+CR) and time to CR.
To determine the expansion and persistence of AUTO4 following infusion.	RQR8/aTRBC1-CAR positive T cells as determined by PCR and/or flow cytometry at a range of time points in the peripheral blood.
Duration of TRBC1 positive T cell aplasia.	Enumeration of circulating TRBC1 positive T cells assessed by flow cytometry at a range of time points in the peripheral blood.

AE = adverse event; ATIMP = advanced therapy investigational medicinal product; CR = complete response; DFS = disease free survival; DOR = duration of response; PCR = polymerase chain reaction: OS = overall survival; PFS = progression free survival; PR = partial response; SAE = serious adverse event; TRBC = T cell receptor beta constant.

2.3 EXPLORATORY OBJECTIVES

The exploratory objectives for this study are as follows:

- To determine the time course and magnitude of cytokine release evaluated using an appropriate assay.

- To assess the duration of depletion of circulating TRBC1 positive T cells as determined by flow cytometry on the peripheral blood and correlate this with disease response.
- To assess antibody and or T cell mediated immune responses against AUTO4.
- To characterise the relationship between the CAR T cell phenotype/genomics and persistence.
- To investigate if there is a relationship between parameters of activity, percent TRBC1 positive T cells, level of TRBC1 expression on tumour cells and CAR T cell phenotype.
- To investigate if there is a relationship between incidence and severity of CRS, neurotoxicity or other toxicity, and tumour burden, percent TRBC1 positive T cells, level of TRBC1 expression on tumour cells pre-treatment and CAR T cell phenotype.

3 STUDY DESIGN

3.1 STUDY OVERVIEW

This multi-centre, single-arm study will consist of two parts, a Phase I/dose escalation phase and a Phase II/dose expansion phase:

- Phase I: **Dose escalation**, using a rolling six design to identify the RP2D (optimal) dose of AUTO4. The following doses are intended to be evaluated, but are subject to emerging safety data: 25 x 10^6, 75 x 10^6, 225 x 10^6 , 450 x 10^6 and 900 x 10^6 RQR8/aTRBC1-CAR positive T cells, administered as a single dose.
- Phase II: **Dose expansion** to assess anti-lymphoma activity and safety at the RP2D.

An overview of the study design is presented in Figure 2 below.

Figure 2: Proposed Dose Escalation and Dose Expansion Phases

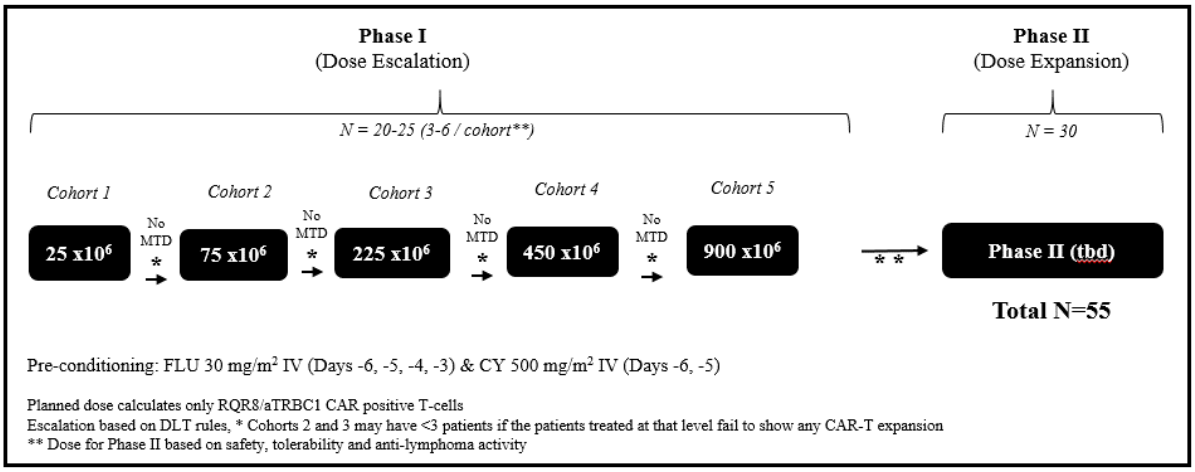

*Cohort 2 and 3 could have at least 1 patient for the dose escalation decision. In this case, any potential single patient cohort would be expanded to n=3-6 (per rolling six study design), if at least 1 patient experiences any level of CAR T-cell expansion (above the limit of detection) or Grade ≥ 1 CRS/Neurotoxicity or ≥ Grade 2 AUTO4-related adverse event in the first 28 days after AUTO4 infusion. The inter-patient interval between patients in 2 different dose level cohorts can not be less than 28 days from AUTO4 infusion of the previous patients to the start of the lymphodepletion of the next patient (the Safety Evaluation Committee (SEC) will meet after the first patient in every cohort completes 28 days).

**At least 3 patient must be treated per cohort from Cohort 4 onwards (per rolling six study design), Subsequent dose increase/dose level (Cohort 5) to be determined based on the available safety-, pharmacokinetic-, pharmacodynamic-data and manufacturing feasibility.

CAR = chimeric antigen receptor; CY = cyclophosphamide; DLT = dose-limiting toxicity; FLU = fludarabine; MTD = Maximum Tolerated Dose; RQR8/aTRBC1 = Ritux QBEnd/10-Ritux-cluster of differentiation 8 sort-suicide gene/anti-T cell receptor beta 1; tbd = to be determined.

The study consists of two phases – Phase I (dose escalation and RP2D), followed by Phase II (dose expansion, efficacy and safety). Each patient (irrespective of phase) will go through the following five stages:

- **Screening stage**: There are two separate Informed Consent Forms (ICFs) during the screening stage – ICF Part A and ICF Part B. After providing written informed consent (ICF Part A) to enter the screening process to determine the TRBC1 status, patients will

be screened for preliminary study eligibility, demographic details, and the provision of an archival tumour tissue sample or a newly acquired tumour tissue sample.

Once a patient is confirmed as TRBC1 positive, the patient will be asked to consent to the main study (ICF Part B). All the remaining screening activities, including the baseline disease assessment, will occur at Screen 1 and Screen 2 – see the Schedule of Assessments for details. Only patients who are identified as TRBC1 positive, and otherwise eligible, will proceed to leukapheresis.

- **Leukapheresis**: Eligible patients that are TRBC1 positive will undergo leukapheresis to facilitate manufacture of the ATIMP, AUTO4.
- **Pre-conditioning**: If sufficient AUTO4 is successfully manufactured and released, and the patient continues to meet eligibility requirements for the study, the patient will proceed to receive a lymphodepleting pre-conditioning treatment with FLU for 4 days and CY for 2 days, to end 3 days (-1 day) before AUTO4 infusion.
- **Treatment stage**: AUTO4 will be administered i.v. as a single infusion on Day 0.
- **Follow-up stage**: The follow-up stage will begin after AUTO4 administration and end 24 months (104 weeks) after the infusion of the last patient with AUTO4 or at their disease progression or withdrawal of consent, which ever happens first. For patients who experienced disease progression post AUTO4 infusion before the End of Study and those still in CR 24-months post infusion please see the Schedule of Assessments and Section 8.5.2 for their follow-up schedule. An End of Study visit will be conducted for all patients.

An overview of the five study stages is presented in Figure 3.

Figure 3: Overview of the Stages of the Study

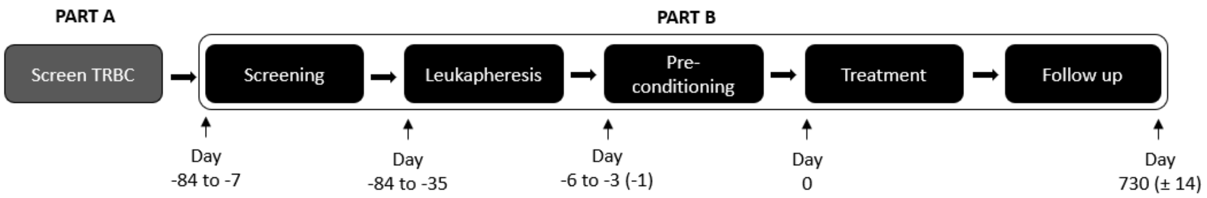

From signing of informed consent (ICF Part A) until the End of Study visit, information relating to adverse events (AEs), laboratory abnormalities, disease response and biomarker changes will be collected according to the Schedule of Assessments.

All AUTO4-treated patients will be eligible for enrolment into a long-term follow-up protocol (AUTO-LT1) at the end of the study (see Section 15). The patients will be followed for safety evaluation and survival for 15 years from the AUTO4 infusion or until death or withdrawal of consent, whichever happens first.

3.2 PHASE I DOSE ESCALATION RULES AND ROLLING SIX DESIGN

Phase I is designed to determine the RP2D of AUTO4 in patients with selected T-NHL. Each dose level may treat up to six patients and is based on total RQR8/aTRBC1-CAR positive T cells. Escalation to the next dose level requires the evaluation of a dose level with at least three patients treated at the planned dose level and completing the 28-day DLT evaluation period. The dose escalation decision rules are outlined in Table 4.

Patient entry at a given dose level will follow a rolling six design which will enable patients to continue to be enrolled at a given dose level up to a maximum of six patients.

The trial is intended to consist of five dose levels (Figure 2); however, the dose levels may be adjusted depending on emerging safety data. Eligible patients will be assigned to dose groups sequentially. The inter-patient dosing interval for AUTO4 in Phase I will be **4 weeks** between the first and second patient and **2 weeks** between subsequent patients (measured from infusion of AUTO4 in one patient to start of pre-conditioning in the next patient), to allow for assessment of possible toxicity, until a dose level is declared safe.

Patients will be admitted to hospital for at least 14 days (or longer) for monitoring after receiving AUTO4 or until all AUTO4-related non-haematological toxicities have returned to ≤Grade 1 or baseline, or longer as clinically necessary. The DLT evaluation period will be **28 days** after the dose of AUTO4.

The Safety Evaluation Committee (SEC) will meet after the first patient in every cohort completes 28 days, to confirm continuation of enrolment to that cohort and thereafter meet again after the third and or sixth patient in a cohort has completed the DLT assessment period.

For Cohorts 2 (75 x 10^6 cells) and 3 (225 x 10^6 cells) if there is no CAR-T expansion (eg no expansion is observed above the assay limit of detection threshold) in any of the patients treated (with at least one patient treated at that dose), together with no Grade ≥ 1 CRS/Neurotoxicity or ≥ Grade 2 AUTO4-related adverse events in the DLT period (first 28 days after AUTO4 infusion) SEC may then meet to determine if that dose level can be declared safe and to escalate to the next level. If any CAR-T expansion level is seen in the potential single patient cohorts (Cohort 2 and 3) the cohort must be expanded and have a minimum of 3 patients treated to be considered complete (per rolling six study design). From cohort 4 (450 x 10^6 cells) onwards, the standard rolling 6 design will apply with a minimum of 3 patients treated per cohort.

Only after a cohort is declared safe by the SEC can the next higher dose level be opened.

Enrolment to a cohort (maximum n=6 evaluable patients/cohort) that has already been declared safe may be undertaken in parallel to the ongoing enrolment of a higher dose level, to enable the treatment of a patient who would not otherwise be treated (for example, a patient with manufactured product who is unable to wait until a slot is available in the higher dose cohort due to rapid disease progression). In this situation, a minimum dosing interval of 7 days will be maintained between patients - even if they are enrolled to a cohort that is declared safe in the Phase I part of the study.

The starting dose level will be 25 x 10^6 RQR8/aTRBC1-CAR positive T cells (Table 5) and if this dose is declared safe then the second cohort will receive 75 x 10^6 RQR8/aTRBC1-CAR positive T cells. Dose escalation may continue until the planned maximum administered dose (MAD) of 900 x 10^6 RQR8/aTRBC1-CAR positive T cells is reached. A dose lower than planned MAD (or next higher dose) may be evaluated if emerging safety data suggest that it may be more appropriate.

Additionally, based on emerging data, more than one RP2D may be determined. For example, if the 75 x 10^6 RQR8/aTRBC1-CAR positive T cell dose is declared as RP2D then Phase II can be opened and in parallel further evaluation of Phase I dose escalation may be continued to determine a second (higher) RP2D dose and or schedule. Should there be a need to assess more than one RP2D in Phase II then this will be undertaken only after a protocol amendment.

If patients are treated below the planned dose due to AUTO4 manufacturing limitations (outside the ± 20% window) or other reasons, then those patients will not be considered evaluable for making dose escalation decisions (additional patients will be treated to meet the minimum number needed to make the dose escalation decision). However, dose escalation

decisions will consider all available data, including biomarker data and the safety profile of all patients treated. No patient will be treated below 15 x 10^6 RQR8/aTRBC1-CAR positive T cells. All patients will be evaluated for efficacy.

Table 4: Dose Escalation Decision Rules

Number of Patients with DLT at a Given Dose Level	Escalation Decision Rule
Zero out of three (where all three patients have been followed for the 28-day DLT period)<br>For Cohorts with less than 3 patients (specifically Cohort 2 and 3):<br>• zero treated have a DLT<br>• zero Grade ≥ 1 CRS/Neurotoxicity or ≥ Grade 2 AUTO4-related adverse events in the first 28 days after AUTO4 infusion	Escalate to the next dose level. Or per sponsor decision enrol three additional patients at the current dose level for a total of six patients to further explore safety and efficacy.<br>For Cohorts with less than 3 patients (specifically Cohort 2 and 3):<br>Escalate to the next dose level. Or per sponsor decision, enrol two additional patients (to have a total of 3) at the current dose level or a total of six patients to further explore safety and efficacy.
One out of three (irrespective of whether all three patients have completed the 28-day DLT period)	Continue to enrol three additional patients at the current dose level for a total of six patients.
One out of six (where all six patients have completed the 28-day DLT period)	Escalate to the next dose level or an intermediate dose level.
Two or more patients in a dosing cohort (up to six patients)	The MTD has been exceeded. Either:<br>• Evaluate a dose lower than the current dose (following a substantial protocol amendment).<br>• Expand a prior cohort up to six patients.

DLT = dose limiting toxicity; MTD = maximum tolerated dose.
Note: The Investigators may override these guidelines if there are particular safety issues, for which moving to a higher dose is not considered appropriate.

3.3 PLANNED DOSE LEVELS

The planned dose levels are presented in Table 5. These dose levels were selected as they provide an optimum range for assessing safety, CAR T cell persistence and anti-tumour activity. This range is within the dose levels assessed in other CAR T studies. Based on emerging data, intermediate or additional dose levels may also be explored as described above.

Table 5: Planned Dose Levels and Treatment Cohorts

Dose Levels	Treatment Cohorts	Pre-conditioning (Flu & CY; Days -6, -5, -4, -3 [-1 day])	Total RQR8/aTRBC1-CAR Positive T Cells (Day 0)	Number of Patients
Dose Level 1	Cohort 1	Yes	25 x 10^6	3 treated
Dose Level 2	Cohort 2	Yes	75 x 10^6	1-6*
Dose Level 3	Cohort 3	Yes	225 x 10^6	1-6*
Dose Level 4	Cohort 4	Yes	450 x 10^6	3-6 **
Dose Level 5	Cohort 5	Yes	900 x 10^6	3-6**

CAR = chimeric antigen receptor; FLU-CY = fludarabine and cyclophosphamide; RQR8/aTRBC1 = Ritux QBEnd/10-Ritux-cluster of differentiation 8 sort-suicide gene/anti-T cell receptor beta 1.
* For Cohorts 2 (75 x 10^6 cells) and 3 (225 x 10^6 cells) if there is no CAR-T expansion in any of the patients treated (with at least one patient treated at that dose) together with no Grade ≥ 1 CRS/Neurotoxicity or ≥ Grade 2 AUTO4-related adverse

events in the first 28 days after AUTO4 infusion, SEC may approve escalation to next level. If any CAR-T expansion (above the assay limit of detection) is seen the cohort must have a minimum of 3 patients treated to be considered complete (per rolling six study design)
** From Cohort 4 (450 x 10^6 cells) onwards, standard rolling 6 design will apply with a minimum of 3 patients treated per cohort. Cohort is expanded from 3 to 6 patients when 1 patient has a DLT.

Note: The dose determination is based solely upon the genetically modified cells (i.e. RQR8/aTRBC1-CAR positive T cells). A patient may be evaluable for a planned dose level if the dose is within ±20% of the prescribed RQR8/aTRBC1-CAR positive T cells dose.

- On occasion, AUTO4 production may fail to generate sufficient cells for the current dose level. In this case, the patient can be treated on study, but at a lower dose; however, if production fails to generate ≥15 x 10^6 (approximately 0.2 x 10^6/kg RQR8/aTRBC1-CAR positive T cells), then the patient will not be treated on the study. Only patients treated at the planned dose level will be evaluable for dose escalation decision making and primary efficacy analysis. Additional patients will be treated to meet the minimum number of patients needed to make the dose escalation decision.
- If emerging data suggest that escalation to an intermediate dose which is a lower than the planned dose is appropriate, then such an intermediate dose escalation can be undertaken. The total number of patients in Phase I may also be increased if necessary.
- If emerging safety and efficacy data suggest further dose escalation is warranted, any doses higher than 900 x 10^6 RQR8/aTRBC1-CAR T cells will not be undertaken without a protocol amendment.

3.4 PHASE II DOSE EXPANSION

Once the RP2D is determined, the Phase II dose expansion part of the study will open. This part will recruit and treat up to 30 patients with AUTO4 at the RP2D. There is no mandated inter-patient dosing interval in this phase.

3.5 DOSE LIMITING TOXICITY

Toxicities will be graded for severity according to the National Cancer Institute (NCI) Common Terminology Criteria for Adverse Events (CTCAE), version 5.0, with the exception of neurotoxicity, which will be assessed according to the American Society for Transplantation and Cellular Therapy (ASTCT)/American Society for Blood and Marrow Transplantation (ASBMT) Immune Effector Cell-associated Neurotoxicity Syndrome (ICANS) grading criteria. The DLT criteria take into consideration the single dose nature of AUTO4 treatment (unlike repeat dose treatments of usual anti-cancer agents), the potential for differential expansion of CARs post infusion in different patients and the inclusion of consolidation therapy. As well as features inherent to CAR therapy, transient fever due to low grade CRS, and in the setting of pre-conditioning, induced cytopenias/neutropenia are seen in most patients. These are not necessarily classical neutropenic fevers resulting from infection.

The DLT evaluation period will be **28 days** after the infusion of AUTO4.

Dose limiting toxicity will be defined as:

- Any new non-haematological AE of Grade 3 or higher toxicity using the NCI CTCAE (version 5.0), which is probably or definitely related to AUTO4 therapy, which occurs within the DLT evaluation period, and which fails to resolve to Grade 2 or better within 14 days, despite appropriate supportive measures.

- A Grade 4 CRS.
- Any other fatal adverse reaction (Grade 5) or life-threatening event (Grade 4) that cannot be managed with conventional supportive measures or which in the opinion of the SEC necessitates dose reduction or other modification to trial treatment to avoid a similar hazard in future patients. Efforts should be made to perform an autopsy in case of a fatal event where the aetiology is unclear.
- Any reason for activation of the safety switch after receiving AUTO4.
- Any event that in the opinion of treating investigators and/or Medical Monitor puts the patient at undue risk may also be considered a DLT

Reporting Requirements for DLT

All DLTs must be reported to the Sponsor as SAEs within 24 hours of site staff becoming aware of them (see Section 12.3.5). The Sponsor will notify all DLTs to the SEC and the Independent Data Monitoring Committee (IDMC).

Maximum Tolerated Dose: The MTD is defined as the highest dose level of AUTO4 at which ≤one patient out of six patients experiences a DLT during the DLT evaluation period. If two or more out of six patients at a dose level experience a DLT during the DLT evaluation period, the MTD has been exceeded. If the MTD is exceeded due to a specific toxicity that can be managed with supportive care, an additional three patients may be treated at the dose level that exceeded the MTD with establishment of supportive care measures. A summary of available safety data and a description of the plans for supportive care measures with further enrolment at that dose level will be provided to Independent Ethics Committees/Institutional Review Boards (IECs/IRBs) prior to dosing.

Maximum Administered Dose: The planned MAD for this study is 900×10^6 RQR8/aTRBC1-CAR positive T cells in the event the MTD is not defined. The MAD may be lower based on emerging data.

Recommended Phase II Dose: The RP2D is the optimal dose and will be either identical to the MTD or MAD (or a lower dose), selected on the basis of a cumulative review of safety, persistence of the CAR T cells and clinical activity. The RP2D dose level may be expanded to up to six patients to further characterise safety.

3.6 SAFETY STOPPING CRITERIA FOR THE CLINICAL TRIAL

The study could be stopped by the SEC or the IDMC upon occurrence of any of the following events:

- Unexpected and related SAEs that exposed patients participating in the study to unacceptable risk of harm *(class-related toxicities such as CRS and neurotoxicities will not automatically result in stopping unless patients are exposed to risk above what is generally seen with similar therapies).*
- Uncontrolled SAEs related to identified risks (see Section 5).
- The occurrence of Grade 4 toxicity in three patients, unless in the opinion of the IDMC (after review), it is likely manageable with the implementation of appropriate supportive care.
- Death of a patient at any time after therapy that is at least possibly related to CAR T cell therapy.

- The occurrence of a second malignancy at any point after therapy that is at least possibly related to the CAR T cell therapy.

The study may be restarted after appropriate preventive or management guidelines have been instituted and a substantial protocol amendment has been approved by the relevant regulatory authorities and ethics committees.

3.7 STUDY DURATION

The total study duration is estimated to be 7 years from first patient enrolled to the last patient last visit (24month visit). The end of the study (EoS) is defined as LPLV and is expected to be 24 months after the last patient has received AUTO4 infusion (or earlier in the event of patient death, study closure by the Sponsor or consent withdrawal).

In the event of disease progression prior to the EoS, patients will continue to be monitored for safety and survival under this study protocol in order to collect health authority requested data (e.g. delayed AEs) until the end of the study. The survival follow-up can be conducted via telephone contact if necessary. At Study Closure/End of the Study, all patients who have received AUTO4 will be eligible to enrol in a separate long-term follow-up study protocol (separate informed consent will be provided for this protocol; Section 8.5). All patients will undergo semi-annual and annual evaluations for up to 15 years following AUTO4 infusion as recommended by health authority guidance for patients treated with such gene therapies.

3.8 NUMBER OF PATIENTS

Approximately 200 patients in total are expected to be consented, registered and screened for eligibility for entry to this study. This assumes that approximately 35% of patients are identified as TRBC1 positive (and assumes that a small number of patients will not provide sufficient evaluable tissue). Of those that are identified as TRBC1 positive, it is assumed that a further approximate 20% will fail the AUTO4 manufacture and/or will not continue to meet the inclusion and exclusion entry criteria. Up to 55 patients in total are anticipated to be treated with AUTO4 therapy.

- **Phase I:** Dose escalation involving up to a total of 25 patients (up to six patients per dose cohort and five cohorts).
- **Phase II:** Dose expansion involving up to a total of 30 patients.

The sample size for Phase II of the study will follow Simon's two-stage optimal design as described in Section 14.1.

4 SCIENTIFIC RATIONALE FOR STUDY

4.1 DESIGN RATIONALE

This single-arm, multi-centre, open-label, dose-escalation Phase I/II study is designed to assess safety, tolerability, and RP2D and MTD, if a MTD exists, for AUTO4. A cautious dose escalation design encompassing three dose levels is considered appropriate. As these patients are expected to be relatively uncommon and may have rapid disease progression, a rolling six dose escalation design in Phase I of the study has been chosen. This will enable continued enrolment of patients even if not all the previously dosed patients (usually three) at the current dose level have been followed for the 28-day DLT period.

A dose escalation with ≤half log increase over the previous dose is appropriate considering the *in vivo* expansion of the CAR T cells. CAR T studies in B cell lymphoma have used half-log/300% (Brudno and Kochenderfer 2016) to a log/1000% (Turtle et al. 2016) increase in dose. The proposed dose levels between 25x 10^6 and 900 x 10^6RQR8/aTRBC1-CAR positive T cells are conservative and designed to identify a safe and efficacious dose for Phase II of the study. The rationales for the starting dose levels and dosing schedule are outlined in detail in Sections 4.2.1 and 4.4.

During Phase I, a minimum dosing interval of 4 weeks between the first and second patient, and thereafter 2 weeks between patients until the dose is declared safe, is incorporated to reduce the risk of inducing severe adverse effects in more than one patient. Additionally, hospitalisation for at least 14 days after AUTO4 infusion is designed to enable patient monitoring for safety. A DLT assessment window of 28 days is incorporated and is appropriate as the therapy involves a single dose of AUTO4 and the majority of toxicities with CAR T therapy occur within the first few weeks (Davila et al. 2014). Considering the extensive clinical experience with these agents, this window is considered adequate.

The rationale for Simon’s two-stage optimal design for the Phase II part of the study is to allow for the termination of the study at interim analysis after 10 patients if the true response rate is 10% or less. The rationale for dosing up to 30 patients is to detect early signs of efficacy in addition to generating additional safety data at the RP2D.

4.2 POPULATION RATIONALE

Most patients with PTCL will either not achieve remission or will relapse after first line therapy. Given the paucity of data and limited therapeutic options, participation in clinical trials is recommended (National Comprehensive Cancer Network PTCL Guidelines 2016). Most patients with relapsed or refractory PTCL have poor outcomes with short survival. In a study of 153 patients (Mak V et al, 2013) with PTCL-NOS, Angioimmunoblastic T cell lymphoma and Anaplastic large cell lymphoma (both ALK+ and ALK-); after relapse or progression in the absence of hematopoietic stem-cell transplantation, the median time from initial diagnosis to relapse or progression after first treatment was only 6.7 months. The median overall survival and median progression-free survival after relapse or progression were 5.5 and 3.1 months respectively. This was minimally improved in patients who received chemotherapy at relapse with median overall survival and median progression-free survival at 6.5 and 3.7 months respectively. For subsets like ALK-positive anaplastic large cell lymphoma (ALCL) with newer treatment options such as brentuximab, outcomes have shown incremental improvement (Pro et al. 2012), but overall the prognosis for most PTCL subtypes in the relapse or refractory setting remains poor.

The study patient population for this study is TRBC1 positive selected T- cell lymphoma and will include the three most common sub-types of PTCL which are typically nodal and present with late stage disease and have similar clinical course and poor prognosis in the relapsed/refracted setting. These subtypes are:

- Peripheral T cell lymphoma NOS
- Angioimmunoblastic T cell lymphoma
- Anaplastic large cell lymphoma

4.2.1 Current Therapeutical Options for this Population and Rationale for Inclusion

In the frontline setting most patients with PTCL are treated with multi-agent anthracycline based chemotherapy regimens such as cyclophosphamide-doxorubicin-oncovin (vincristine)-prednisone (CHOP). A systematic meta-analysis evaluated CHOP or CHOP-like regimens in 2815 patients with PTCL. The CR rates associated with anthracycline-based regimens ranged from 30% to 76% across studies and subtypes of PTCL. As expected, ALCL showed a higher complete response (CR) rate with anthracycline-based chemotherapy than other T cell lymphomas; in patients with Angioimmunoblastic T cell lymphoma, a CR rate of 36% to 70% was seen and in patients with PTCL-NOS, 44% to 64% was observed. The 5-year overall survival (OS) was of 38.5% (Abouyabis et al. 2011). Autologous hematopoietic cell transplantation is incorporated into the initial treatment of patients with PTCL as consolidation therapy after initial combination chemotherapy.

Most patients with PTCL will however either not achieve remission or will relapse after first line therapy (Dreyling et al. 2013). Given the paucity of data and limited therapeutic options, participation in clinical trials is recommended (National Comprehensive Cancer Network PTCL Guidelines 2016, ESMO consensus guidelines). Most patients with relapsed or refractory PTCL have poor outcomes with short survival. In a study of 153 patients (Mak et al. 2013) with PTCL-NOS, Angioimmunoblastic T cell lymphoma and Anaplastic large cell lymphoma (both ALK+ and ALK-) and in the absence of hematopoietic stem-cell transplantation, the median time from initial diagnosis to relapse or progression after first treatment was only 6.7 months. The median overall survival and median progression-free survival after relapse or progression were 5.5 and 3.1 months respectively. This was minimally improved in patients who received chemotherapy at relapse with median overall survival and median progression-free survival at 6.5 and 3.7 months respectively. For subsets such as ALCL, with newer treatment options such as brentuximab, outcomes have shown incremental improvement (Pro et al. 2012), but overall the prognosis for most PTCL subtypes in the relapse or refractory setting remains poor.

Conventional platinum-based regimens such as DHAP, ESHAP or ICE, as used in the larger Diffuse Large B Cell lymphoma population, are used in patients with relapsed PTCL especially those who are transplant candidates. The efficacy of these regimens in PTCL is not well known as no large published study is available in these lymphomas. The Canadian Cancer Trials Group LY.12 Phase 3 trial included 59 patients with PTCL. Among these, 81% had advanced stage disease, and 41% were refractory to primary therapy. The ORR after two cycles of salvage chemotherapy was 36%; no difference was observed between dexamethasone, cytarabine, cisplatin (33%), and gemcitabine, cisplatin, dexamethasone (38%) therapy. At one year, event-free survival (EFS) was 16% and OS was 28% (Skamene et al. 2017). Patients who received autologous stem cell transplant (SCT), two-year EFS and OS were 21% and 42%, respectively.

Patients with PTCL had inferior OS and outcomes when compared to patients with B cell lymphomas.

Newer agents are being evaluated and have shown some marginal efficacy in patients with relapsed PTCL. A single arm Phase 2 PROPEL study of pralatrexate evaluated 115 heavily pre-treated patients with the most common types of PTCL. In this study, the ORR was 29% with CR/CRu (complete response unconfirmed) of 11%; the median PFS and OS was 3.5 and 14.5 months respectively. Responses were observed across all histologic subtypes, although patients with Angioimmunoblastic T cell lymphoma were less likely to respond than patients with other common PTCL subtypes (O'Connor et al. 2011).

HDAC inhibitors such as belinostat and romidepsin have shown modest efficacy in patients with PTCL. In the single arm Phase 2 BELIEF study with belinostat, a total of 129 patients were enrolled, with a median of two prior systemic therapies. ORR and CR in the 120 evaluable patients was 26%, 11% respectively. Median duration of response was 13.6 months, whilst median PFS and OS were 1.6 and 7.9 months respectively (O'Connor et al. 2015).

Similarly, in the single arm Phase 2 study of romidepsin, 130 patients with histologically confirmed PTCL who had relapsed or were refractory to least one prior therapy were treated. The ORR was 25% including 15% CR/CRu. The median duration of response was 17 months (Coiffier et al. 2012).

For patients with CD30 positive Anaplastic large cell lymphoma, newer treatment options such as anti CD30 antibody drug conjugate brentuximab have shown incremental improvement in outcomes (Pro et al. 2012). In this Phase 2 study of 58 patients the ORR was 86% and 57% achieved a CR. The median durations of overall response and CR were 12.6 and 13.2 months, respectively.

With exception of brentuximab, these newer agents are only approved by the Food and Drug Administration (FDA) and are not readily available elsewhere.

Relapses in patients with PTCL are common; therefore, SCT as a consolidation and salvage strategy may improve therapeutic results. However, the role of SCT especially in relapsed PTCL remains to be determined and there is lack of prospective clinical trials. Some limited retrospective studies have evaluated the efficacy of autologous and allogeneic transplant in relapsed PTCL. For example, a longitudinal study of 241 patients with PTCL NOS, ALCL and angioimmunoblastic T cell lymphoma has shown that SCT is better in the earlier disease setting with chemo-sensitive disease when used as consolidation therapy at first CR (Smith 2013).

4.3 STARTING DOSE RATIONALE

A starting dose of 25 x 10^6 RQR8/aTRBC1-CAR positive T cells (single dose) is proposed, which corresponds to a dose of approximately 0.33 x 10^6 RQR8/aTRBC1-CAR positive T cells/kg based on an average person's weight of 75 kg. [redacted].

[redacted]

[redacted].

4.4 RATIONALE FOR MAXIMUM ADMINISTERED DOSE (MAD)

A MAD of 900x 10^6 RQR8/aTRBC1-CAR T cells in this study has been chosen based on the experience with CD19 CARs in B cell lymphoma. [redacted].

4.5 RATIONALE FOR DOSING SCHEDULE

Chimeric antigen receptor T cell therapies are generally administered once, undergo significant expansion *in vivo* upon contact with the target antigen expressed on tumour cells and, particularly where a 41BB-ζ co-stimulatory domain is incorporated into the CAR, persist long-term in a proportion of patients (Maude et al. 2014). It is anticipated that AUTO4 will have similar expansion and persistence *in vivo* to the CD19 CAR positive T cells with 41BB-ζ co-stimulatory domain, such as those utilised in the University of Pennsylvania studies, rendering the need for re-dosing unnecessary (Schuster et al. 2016). On rare occasion, a re-treatment dose may be given if the patient meets the criteria for re-treatment (Section 8.4.8).

Patients will receive a single dose of AUTO4. After treatment with AUTO4, safety will be monitored closely (both in hospital and as an outpatient), and efficacy will be assessed periodically as described in the Schedule of Assessments. The timing of assessments is designed to capture early signs of toxicity and efficacy.

4.6 FLUDARABINE AND CYCLOPHOSPHAMIDE PRE-CONDITIONING RATIONALE

Pre-conditioning strategies that deplete host lymphocytes prior to adoptive transfer of tumour specific T-lymphocytes are thought to enhance treatment efficacy by eliminating regulatory T cells and increasing access of the transfused CAR T cells to activating cytokines (Klebanoff et al. 2005, Wrzesinski and Restifo 2005). Cyclophosphamide has an established history in lymphodepleting regimens used prior to adoptive cell immunotherapy (Sporn et al. 1993, Curti et al. 1998, Brentjens et al. 2011, Chu et al. 2012). It is used alone or often used in combination with other agents (Dudley et al. 2008, Laurent et al. 2010, Geller et al. 2011) such as FLU (Louis et al. 2011). The FLU-CY combination is well tolerated and is commonly used in the treatment of chronic lymphocytic leukaemia (Hallek 2013).

Cyclophosphamide and FLU based pre-conditioning have become the preferred regimen for CAR T therapies and have been used in multiple studies (Lee et al. 2014, Kochenderfer et al. 2015, Ali et al. 2016). In terms of overall efficacy, the FLU-CY combination is also considered to be superior to CY alone (Lee et al. 2016, Turtle et al. 2016).

The exact dose regimens used for lymphodepletion prior to CAR T cell therapy are variable (IB Section 7.2). The regimen utilised in this study FLU-CY (FLU [30 mg/m^2 for 4 days] and CY [500 mg/m^2 for 2 days]) is similar to that evaluated in multiple clinical studies across institutions and appears both safe and active (Lee et al. 2014, Ali et al. 2016, Fry 2016, Kochenderfer et al. 2016, Neelapu et al. 2016, Turtle et al. 2016). See IB Section 7.2 for further background information relating to the pre-conditioning treatment.

In the current study, we propose pre-conditioning treatment with low dose FLU & CY (FLU [30 mg/m^2 for 4 days] and CY [500 mg/m^2 for 2 days]) followed, (after a 3-day wash out) by a low starting dose of 25 x 10^6 RQR8/aTRBC1-CAR positive T cells and up titrating the CAR T dose based on emerging safety data. The 3-day washout period prior to infusion of AUTO4 is incorporated in consideration of the 20-hour half-life of FLU.

In summary, given the nature of the patient population, the role of FLU-CY in enhancing the clinical efficacy of CAR T cells, the proposed dose and regimen are justified.

4.7 BIOMARKER COLLECTION AND ASSESSMENT RATIONALE

Current experience with CAR T cell therapies indicates that the safety depends on the disease burden (in ALL), CAR T proliferation, and cytokine release; and efficacy depends on CAR T proliferation and persistence. To this end, the current biomarker strategy is designed to assess blood cytokine levels, and CAR T levels and persistence at various time points after AUTO4 infusion. In addition, peripheral blood sampling will be performed to assess functionality (such as proliferation) of RQR8/aTRBC1-CAR T positive cells, which would help evaluate surrogate efficacy as well as resistance.

5 RISKS AND MITIGATION STRATEGY

This exploratory study is designed to assess the safety and biological activity of different doses of AUTO4 in a limited number of patients. Given the novelty of the treatment, this study will involve patients whose disease has progressed after treatment with the main approved classes of agents. Such patients are considered to have poor prognosis and are candidates for promising experimental therapies.

5.1 TUMOUR TISSUE BIOPSY

If it is necessary for the patient to provide newly acquired tumour tissue, this can be provided from either an excisional biopsy or core needle biopsy. Although an excisional biopsy is preferred, the Investigator may choose to provide a core needle biopsy if a lesion is suitable. The serious risks from a biopsy include pain, haemorrhage, biopsy site infection and rarely, death. The people performing these procedures are well experienced and routinely perform such procedures under required anaesthesia (local, regional) and under strict aseptic conditions which is likely to minimise the pain/discomfort, bleeding and infection risk.

5.2 LEUKAPHERESIS

Leukapheresis is a prerequisite for preparing AUTO4. Cell production efficiency of at least 80% of the anticipated dose is required and is dependent on the quality of the leukapheresate. AUTO4 infusion may or may not provide clinical benefit to these patients. Risks of leukapheresis are summarised in Table 6.

Table 6: Leukapheresis – Risks and Mitigation Strategy

Risks	Mitigation Strategy
**Pain and bruising** due to insertion of cannula/central venous access.	Experienced clinicians/nurses performing the procedure, analgesics to be used as needed for pain.
**Bacterial bloodstream infections** associated with the insertion of access and return venous access devices.	The procedure will be carried out by trained and experienced personnel and risks will be minimised by strict adherence to aseptic measure.
**Symptoms of hypocalcaemia e.g., muscle cramps** due to chelation by anticoagulants used to prevent clotting.	Patients will be monitored for symptoms of hypocalcaemia, the rate of citrate infusion to the patient and duration of the procedure will be controlled by experienced personnel. Calcium supplements will be given as needed.

5.3 PRE-CONDITIONING CHEMOTHERAPY

Pre-conditioning with CY and FLU will only be undertaken after confirmation of the successful production of AUTO4. The risks of pre-conditioning with chemotherapy are summarised in Table 7. Please refer to the Summaries of Product Characteristics (SmPCs) for more details on these products.

Table 7: Pre-conditioning Chemotherapy – Risks and Mitigation Strategy

Risks	Mitigation Strategy
**Myelosuppression** resulting in anaemia, thrombocytopenia, and lymphopenia, is the most common toxicity. Moderate to severe myelosuppression is possible. Nadir for granulocyte is 1 to 2 weeks and platelets 2 to 4 weeks after chemotherapy, with recovery usually within 4 to 6 weeks. Neutropenic fever, infections, sepsis, and septic shock may occur and may sometimes be fatal.	The FLU and CY chemotherapy given is milder than general chemotherapy received by patients and will be given only once (one cycle, given over 3 days). Anti-microbial prophylaxis (including pneumocystis prophylaxis and acyclovir) may be given to prevent infections and if infections arise, these will be treated as per institutional guidelines. Blood, platelet and fresh frozen plasma transfusions will be given as per standard institutional guidelines. All sites have extensive expertise in managing these complications.
**Cyclophosphamide** associated toxicities including, but not limited to haemorrhagic cystitis, pyelitis, myocarditis and myo-pericarditis, pneumonitis and pulmonary fibrosis; veno-occlusive liver disease (per approved label) may also occur.	Given the low dose and short duration of treatment, these toxicities are unlikely. Patients will be given anti-emetic prophylaxis and hydration during lymphodepletion as per institutional policy. If haemorrhagic cystitis occurs, i.v. fluids and mesna will be given. Other toxicities will be managed as per standard institutional policy and by trained personnel.
**Fludarabine** is generally well tolerated: the most common side effects are lymphopenia and infection. Serious, and sometimes fatal infections, including opportunistic infections and reactivations of latent viral infections such as Herpes zoster, Epstein-Barr virus, and progressive multifocal leukoencephalopathy, have been reported in patients treated with higher doses and for much longer durations. Neurotoxicity can occur but generally at higher doses. Other associated toxicities (as per the label) include but are not limited to autoimmune disorders, hepatic impairment, neuro-toxicity, and renal impairment.	Given the low dose and short duration of treatment, these toxicities are less likely to occur. Toxicities will be managed as per standard institutional policy and by trained personnel.

CY = cyclophosphamide; FLU = fludarabine; i.v. = intravenous

5.4 AUTO4 INFUSION

Risks associated with the infusion of AUTO4 are presented in Table 8.

Table 8: AUTO4 Infusion – Risks and Mitigation Strategy

Risks	Mitigation Strategy
**Infusion reactions** may occur with the infusion of AUTO4.	The product is autologous and the risk is likely to be low. Patients may be pre-medicated with diphenhydramine/chlorpheniramine and paracetamol/acetaminophen (See Section 10.3).
**Cytokine-release syndrome** is a recognised toxicity associated with CAR T cell therapies. Clinical symptoms indicative of CRS includes culture negative fever, but may also include, myalgia, nausea/vomiting, tachycardia, hypoxia, hypotension, headache, confusion, tremor, and delirium. Potentially life-threatening complications of CRS may include cardiac dysfunction, acute respiratory distress syndrome, renal and/or hepatic failure, and DIC. The clinical features may overlap with macrophage activation syndrome. The absolute level of risk in T-NHL is difficult to determine.	Patients will be monitored for CRS and appropriate treatment given in the event of the occurrence (see Section 10.4). These have been formulated based on prior CAR T cell-gene therapy experience at various institutions.
**Neurotoxicity** has been seen in patients with leukaemia after treatment with CAR T cell therapy and is now referred to as ICANS. The cause of neurotoxicity is not well-understood, although it is generally reported to be fully reversible. Although symptoms can vary, the early manifestations of ICANS are often tremor, dysgraphia, and mild difficulty with expressive speech especially naming objects, impaired attention, apraxia, and mild lethargy. Other symptoms can include confusion, depressed level of consciousness/encephalopathy, hallucinations, dysphasia, ataxia, apraxia, cranial nerve palsies, and seizures. Headache is a non-specific symptom, frequently occurring during fever or after chemotherapy, thus, headache alone is not a useful marker of ICANS. Expressive aphasia, on the other hand, appears to be a very specific symptom of ICANS.	The patient will be closely monitored for neurological signs and symptoms; neuroimaging will be performed as required. Appropriate treatment, including dexamethasone, will be given in the event of severe neurotoxicity, including cerebral oedema and seizures (See Section 10.6).
**Off-tumour toxicity** could be due to either on-target (due to expression of the antigen on non-tumour cells) or off-target (recognition of a molecular target other than RQR8/aTRBC1) interactions. Historically, there have been reports of on-target/off-tumour toxicity with CAR therapy as well as T cell receptor engineered T cells; the details are described in the IB.	Preclinical toxicology indicates the risk to be low as TRBC1 expression is limited to the T cell lineage and this risk is likely to be low.
**Cardiac toxicity**: One cardiac-related fatality has been reported with CD19 CAR positive T cells, although the causality was unclear.	The protocol excludes patients with underlying/prior cardiac history. Additionally, patients will be required to undergo ECHO or MUGA cardiac scans to demonstrate normal cardiac functioning prior to study enrolment and at the onset of CRS.

Risks	Mitigation Strategy
**Tumour lysis syndrome** may occur on treatment with AUTO4 due to rapid destruction of malignant cells in the context of a high tumour burden, although this is rarely seen after CD19 CAR positive T cell therapy in lymphoma and is also less likely to happen with AUTO4 in T-NHL.	If TLS occurs, all sites have extensive experience in managing this complication and supportive care will be initiated rapidly as per standard institutional protocols. Based on emerging data prophylactic therapy with allopurinol and i.v. fluids may be given (see Section 10.7).
**T cell aplasia** may occur because of depletion of normal T cells by AUTO4. Potentially this may increase the risk of infections. However, since only half of the T cell population is being targeted for depletion, and polyclonal nature of the immune response this risk is likely to be low. The duration of partial T cell aplasia is likely to be variable depending on the persistence of CAR T cells.	Patients will be monitored for infections as per the Schedule of Assessments for CMV, EBV and adenovirus. Other infections such as JCV, toxoplasmosis and fungal infections will also be monitored per institutional guidelines for bone marrow transplant patients, or as clinically indicated. In the event of active infection, patients will be managed as per the institutional standards or as clinically indicated.<br>Additionally, where possible, unprocessed leukapheresate will be stored. Should a patient develop complete T cell aplasia or, severe and/or recurrent infections, then the rituximab safety switch can be activated to deplete AUTO4. Since stem cells are not impacted, normal haematopoiesis should lead to recovery of T cell compartment. In addition, the unprocessed leukapheresate can be infused to facilitate rapid immune re-constitution. Should this be unsuccessful then the patient could be rescued with an allogenic stem cell transplant. The patients recruited in this study are naïve for allogenic stem cell transplant.
**Sepsis:** Sepsis leading to death has been noted in 3 patients treated with very high doses (5 x 10^8 CAR T cells) of CD19 CAR in a study done at University of Pennsylvania.	Gradual titration of dose, prophylactic antibiotics and antiviral medications as appropriate, close monitoring of patients and early intervention is likely to reduce the risk.
**Insertional mutagenesis**: Disruption of the cellular transcriptome by retroviral-mediated insertion has, in rare cases and in haematopoietic stem cell gene therapy trials only, resulted in insertional mutagenesis, and given rise to haematopoietic stem cells with elevated expression of growth-related genes, which subsequently resulted in T cell leukaemia and/or lymphoma.	T cells that have been transduced with a retroviral vector have, to date, been proven safe. Unlike haematopoietic stem cells, T cells are highly resistant to retroviral vector-induced transformation. The patients will be monitored for secondary malignancy and survival in a long-term follow-up protocol for a total of 15 years.

Risks	Mitigation Strategy
**Risk of transducing and re-infusing lymphoma cells**	Typically, these patients will have very low circulating malignant cells, and hence the absolute number of malignant T cells present within the leukapheresate is also likely to be low. Additionally, it has been noted that TRBC1 positive cells are refractory to transduction with RV29328 (this is postulated to be due to receptor interference, as the virions may bind the cells via the TCR rather than the RD114 receptor).<br>Moreover, any incidental TRBC1 positive lymphoma cells will be killed by RQR8/aTRBC1-CAR positive T cells during the expansion phase of the manufacturing process.<br>In addition, a negative sorting step has been incorporated during manufacturing to deplete any normal or malignant TRBC1 positive cells prior to transduction.
**Risks associated with a replication competent retrovirus (RCR):** There is a risk that a recombination event may occur during vector production that results in a RCR, which may be pathogenic in humans.	All vector lots are tested for RCR prior to release to sites. The risks of an RCR are unknown. To date, no patient has developed an RCR with a retroviral based CAR T cell therapy. Patients will be monitored for RCR by PCR during their scheduled follow-up visits. If a positive signal is confirmed, additional testing will be performed and medical and research experts will be consulted for the optimal treatment approach should any complication arise.
**Dimethyl sulfoxide,** which is part of cryopreservative buffer, may cause an abnormal taste at the time of infusion and body odour lasting 1 to 2 days afterwards. At high doses, DMSO may cause nausea, vomiting, abdominal pain, headache, and haemolysis. Rarely, patients may experience mild or severe cardiac, pulmonary, renal, or neurological symptoms.	Most patients are likely to be exposed to a small dose of DMSO; for every patient, the amount of DMSO exposure will be kept lower than the general institutional maximum limit of 70 g. Additionally, at these dose levels, the side effects are likely to be mild and short lasting.

ALL = acute lymphoblastic leukaemia; BCMA= B Cell Maturation Antigen; CAR = chimeric antigen receptor; CD19 = cluster of differentiation 19; CD22 = cluster of differentiation 22; DIC = disseminated intravascular coagulation; CMV = cytomegalovirus; CRS = cytokine release syndrome; DMSO = dimethyl sulfoxide; EBV = Epstein Barr Virus; ECHO = echocardiogram; IB = Investigator’s Brochure; ICANS = Immune Effector Cell-associated Neurotoxicity Syndrome; JCV = John Cunningham Virus; i.v. = intravenous; MUGA = multigated acquisition (cardiac scans); PCR = polymerase chain reaction; RCR = replication competent retrovirus; RQR8/aTRBC1 = Ritux QBEnd/10-Ritux-cluster of differentiation 8 sort-suicide gene/anti-T cell receptor beta 1; TLS = tumour lysis syndrome; T-NHL = T cell non-Hodgkin Lymphoma.

5.5 UNKNOWN LONG-TERM RISKS OF GENE THERAPY

Following treatment of AUTO4, all patients will be invited to enrol in a long-term follow-up study and monitored for up to 15 years for SAEs related to AUTO4, Adverse Events of Special Interest (AESIs), new malignancies and survival.

6 PATIENT POPULATION

Patients will be eligible for the trial if all the inclusion criteria are met and none of the exclusion criteria applies. There will be no exception to the eligibility requirements at the time of registration. Ensuring patient eligibility is the responsibility of the Principal Investigator or other delegated Investigator(s).

6.1 INCLUSION CRITERIA

Patients must meet all the following criteria for study entry:

1. Male or female, aged ≥18 years.
2. Willing and able to give written, informed consent to be screened for TRBC1 positive T-NHL and to enter the main study.
3. Confirmed diagnosis of selected T-NHL including:
    a. Peripheral T cell lymphoma NOS, or
    b. Angioimmunoblastic T cell lymphoma or
    c. Anaplastic large cell lymphoma.
4. Confirmed TRBC1 positive tumour.
5. Relapsed or refractory disease and have had ≥1 prior lines of therapy.
6. Positron emission tomography (PET)-positive measurable disease per Lugano classification.
7. Eastern Cooperative Oncology Group (ECOG) Performance Status 0 or 1.
8. Adequate Bone Marrow Function without the requirement for ongoing blood products and meets the following criteria:
    a. Absolute neutrophil count ≥1.0 x 10^9/L
    b. Absolute lymphocyte count ≥0.5 x 10^9/L (at entry and prior to leukapheresis)
    c. Haemoglobin ≥80 g/L
    d. Platelets ≥75 x 10^9/L
9. Adequate renal, hepatic, pulmonary, and cardiac function defined as:
    a. Creatinine clearance (as estimated by Cockcroft Gault) ≥60 cc/min.
    b. Serum alanine aminotransferase / aspartate aminotransferase ≤2.5 x upper limit of normal (ULN).
    c. Total bilirubin ≤25 µmol/L (1.5 mg/dL), except in patients with Gilbert's syndrome.
    d. Left ventricular ejection fraction (LVEF) ≥50% by echocardiogram (ECHO) or multiple gated acquisition (MUGA) cardiac scan, unless the institutional lower limit of normal is lower.
    e. Baseline oxygen saturation ≥92% on room air and ≤Grade 1 dyspnoea
10. For females of childbearing potential (defined as <2 years after last menstruation or not surgically sterile), a negative serum or urine pregnancy test must be documented at

screening, prior to pre-conditioning and confirmed before receiving the first dose of study treatment.

For females who are not postmenopausal (<24 months of amenorrhea) or who are not surgically sterile (absence of ovaries and/or uterus), a highly effective method of contraception together with a barrier method must be used from the start of the pre-conditioning stage and for at least 12 months after the last dose of AUTO4 (study treatment). They must agree not to donate eggs (ova, oocytes) for the purposes of assisted reproduction during the study and for 12 months after receiving the last dose of study drug (please refer to Appendix 3).

11 For males, it must be agreed that two acceptable methods of contraception are used from the start of the pre-conditioning stage and for at least 12 months after the last dose of AUTO4 (one by the patient – usually a barrier method, and one by the patient's partner - refer to Appendix 3). Also, that sperm will not be donated during the treatment period and for at least 12 months after the last dose of study treatment.

12 No contra-indications for leukapheresis or the pre-conditioning regimen.

6.2 EXCLUSION CRITERIA

Patients meeting any of the following exclusion criteria must not be enrolled into the study:

1 Patients with T cell leukaemia.

2 Females who are pregnant or lactating.

3 Prior treatment with investigational gene therapy or approved gene therapy or genetically engineered cell therapy product or allogeneic stem cell transplant.

4 Known history or presence of clinically relevant central nervous system (CNS) pathology such as epilepsy, paresis, aphasia, stroke within prior 3 months, severe brain injuries, dementia, Parkinson's disease, cerebellar disease, organic brain syndrome, uncontrolled mental illness, or psychosis. Patients with a known history or prior diagnosis of optic neuritis or other immunologic or inflammatory disease affecting the CNS.

5 Current or history of CNS involvement by malignancy.

6 Clinically significant, uncontrolled heart disease (New York Heart Association Class III or IV heart failure, uncontrolled angina, severe uncontrolled ventricular arrhythmias, sick-sinus syndrome, or electrocardiographic evidence of acute ischaemia or Grade 3 conduction system abnormalities unless the patient has a pacemaker) or a recent (within 12 months) cardiac event.

   a Uncontrolled cardiac arrhythmia (patients with rate-controlled atrial fibrillation are not excluded).

   b Evidence of pericardial effusion

7 Patients with evidence of uncontrolled hypertension or with a history of hypertension crisis or hypertensive encephalopathy.

8 Patients with a history (within 3 months) or evidence of deep vein thrombosis or pulmonary embolism requiring ongoing therapeutic anticoagulation at the time of pre-conditioning.

9 Patients with active gastrointestinal (GI) bleeding.

10 Patients with any major surgical intervention in the last 3 months.

11 Active infectious bacterial, viral or fungal disease (hepatitis B virus, hepatitis C virus, human immunodeficiency virus [HIV], human T cell lymphotropic virus [HTLV] or syphilis) requiring treatment.

12 Active autoimmune disease requiring immunosuppression.

13 History of other neoplasms unless disease free for at least 2 years (adequately treated carcinoma *in situ*, curatively treated non-melanoma skin cancer, breast or prostate cancer on hormonal therapy are allowed).

14 Prior treatment with programmed cell death protein 1 (PD1), programmed death-ligand 1 (PD-L1), or cytotoxic T lymphocyte-associated protein 4 targeted therapy (CTLA-4), or tumour necrosis factor (TNF) receptor superfamily agonists including CD134 (OX40), CD27, CD137 (41BB), and CD357 (glucocorticoid-induced TNF receptor family-related protein) within 6 weeks prior to AUTO4 infusion.

15 The following medications are excluded:

  a Steroids: Therapeutic doses of corticosteroids within 72 hours of leukapheresis or pre-conditioning chemotherapy administration. However, physiological replacement, topical, and inhaled steroids are permitted.

  b Cytotoxic chemotherapies within 2 weeks prior to leukapheresis or AUTO4 infusion.

  c Antibody therapy use within 2 weeks prior to AUTO4 infusion, or five half-lives of the respective antibody, whichever is longer

  d Live vaccine within 4 weeks prior to enrolment.

16 Research participants receiving any other investigational agents, or concurrent biological, chemotherapy, or radiation therapy.

17 Use of rituximab (or rituximab biosimilar) within the last 6 months prior to AUTO4 infusion.

18 Patients, who in the opinion of the Investigator, may not be able to understand or comply with the safety monitoring requirements of the study.

For pre-conditioning chemotherapy and AUTO4 Infusion: Patients meeting any of the following exclusion criteria must not be treated with pre-conditioning chemotherapy or AUTO4 - and have treatment delayed until they no longer meet these criteria:

1 Severe intercurrent infection at the time of pre-conditioning chemotherapy or the scheduled AUTO4 infusion.

2 Requirement for supplementary oxygen or active pulmonary infiltrates or significant deterioration of organ function at the time of pre-conditioning chemotherapy or scheduled AUTO4 infusion.

3 Significant clinical deterioration of organ functions from screening, as determined by the investigator.

7 ADVANCED THERAPY INVESTIGATIONAL MEDICINAL PRODUCT: AUTO4

7.1 AUTO4 DESCRIPTION

AUTO4 is an autologous, cell and gene therapy investigational medicinal product that is manufactured by genetic modification of the patient's own T cells by *ex vivo* transduction with a MLV-derived retroviral vector ([redacted] that has been engineered to stably deliver the RQR8 and aTRBC1-CAR genes. The active substance is the genetically modified (RQR8/aTRBC1-CAR positive) T cells. AUTO4 also contains non-transduced autologous lymphocytes.

AUTO4 is presented for i.v. infusion in one or more CryoMACS® bag(s) as a cell dispersion in phosphate-buffered saline/EDTA/human albumin solution and a final concentration of 7.5% DMSO.

7.2 AUTO4 MANUFACTURING

The Sponsor is responsible for the manufacturing of the drug product, AUTO4, according to Good Manufacturing Practice (GMP) principles and guidelines applicable to ATIMPs. The starting material for generation of AUTO4 is the unstimulated leukapheresate taken from the patient and which will be performed at the study site apheresis collection unit. This may require insertion of central venous access and is a day case procedure to collect peripheral blood mononuclear cells (PBMCs) only. Sites will be responsible for ensuring that the patient biological screening and leukapheresis procedures, including the labelling and issue of the leukapheresis product are carried out per the Human Tissue (Quality and Safety for Human Application) Regulations (SI 2007/1523) and in line with local procedures.

The leukapheresis procedure will be performed by the apheresis unit staff as described in Section 8.2. The collected PBMCs will be transported to the Sponsor Manufacturing Unit for generation of AUTO4, where under aseptic GMP conditions, the PBMCs will be transduced with a recombinant viral vector to co-express aTRBC1 and RQR8. Further details regarding this process can be found in the IB.

AUTO4 is manufactured by genetic modification of the patient's T cells by *ex vivo* transduction with a MLV-derived retroviral vector ([redacted]). Briefly, T cells will be obtained from the leukapheresate taken from each lymphoma patient who has been identified as TRBC1 positive during study screening. After a TRBC1 positive cell depletion of the leukapheresate, the T cells within the TRBC1 negative cell collected fraction are activated and then transduced with the [redacted] retroviral vector. Cells are then expanded (drug substance) and then cryopreserved (drug product).

AUTO4 manufacture and release will take approximately 4 weeks from receipt of leukapheresate at the manufacturing site. Full release testing will be performed to pre-defined specifications.

AUTO4 will be cryopreserved in one or more CryoMACS® bag(s) and stored in a vapour-phase liquid nitrogen environment prior to administration.

Please refer to the AUTO4 IB and CHIM for further details on the manufacture of AUTO4, shipment, storage, and handling.

7.3 PACKAGING AND LABELLING

AUTO4 is supplied as a cryopreserved cell dispersion in a sealed CryoMACS® bag containing the required number of RQR8/aTRBC1-CAR positive T cells for the planned dose level. Each

AUTO4 CryoMACS® freezing bag will be labelled and will include all the required information, including the exact cell content, as applicable per local regulations.

CryoMACS® freezing bags are single use, sterile containers intended for a single cycle of freezing, storage (down to -196°C), and subsequent thawing (at 37°C) of AUTO4 cells. The CryoMACS® freezing bags are comprised of a freezing bag (with access ports) as the primary containment for AUTO4 and an overwrap bag as secondary containment. Additionally, the CryoMACS® freezing bag has a built-in label pocket, which allows for the insertion of the patient label to include patient identification and product specifications for AUTO4. As part of the bag assembly, two spike ports are available which allow access to the bag contents for therapeutic use of the product (via attachment of a sterile transfusion assembly).

7.4 SUPPLY AND STORAGE

Cryopreserved AUTO4 will be supplied to the study sites (authorised to receive genetically modified organisms, in accordance with local requirements) by the Sponsor. Transport of AUTO4 to the study site will be performed by validated shippers under controlled temperature conditions below -150°C. In the case of a temperature excursion occurring during transit, the disposition of AUTO4 will be decided following the review of the temperature data. All temperature excursions outside the storage conditions specified in the Labels/Trial-Specific Procedures for receipt and storage of AUTO4 must be documented and reported to the Sponsor via the Clinical Research Associate as per the CHIM. The Sponsor must be informed immediately if a patient has been administered AUTO4 where a temperature excursion has occurred.

The Investigator/pharmacist/designated personnel will take an inventory and acknowledge receipt of all shipments of the study product to confirm the shipment condition and content.

AUTO4 must be stored at below -150°C, under controlled temperature and will be maintained in a validated liquid nitrogen shipper and temperature monitored, in a secure storage area with restricted access until ready for thawing and preparation as specified in the CHIM. The study site will be responsible for the receipt, storage, and issue of AUTO4, and will comply with trial-specific procedures and the CHIM.

AUTO4 supplied for the AUTO4-TL1 trial is specific for each patient.

7.5 ACCOUNTABILITY AND DESTRUCTION

Study drug accountability and traceability records will be maintained at the site at all times. The unique identification number of the patient, the administration date, batch number, expiry date and dose of AUTO4 dispensed will be recorded on the appropriate inventory forms.

The Sponsor may authorise unused AUTO4 and any product contact materials to be disposed of as standard clinical waste (typically autoclaving). The site must obtain authorisation from the Sponsor before any AUTO4 is destroyed, and AUTO4 destruction must be documented on the appropriate form. Further details can be found in the CHIM. Documented evidence of destruction will be made available to the Sponsor. Ancillary supplies are not required to be returned to the Sponsor. Accurate records of all AUTO4 received at, dispensed from, returned to, and disposed of by the study site should be recorded on the Product Inventory Log.

8 STUDY PROCEDURES AND TREATMENT

8.1 CONSENT, SCREENING, TISSUE BIOPSY AND REGISTRATION

8.1.1 Consent processes

All patients must sign the relevant informed consent form (ICF) prior to the conduct of any study-related procedure. Sites are responsible for assessing a patient's capacity to give informed consent. There are two informed consents for this study:

i. Consent to be screened for TRBC1 positive T-NHL. Patients will be provided with a PIS and ICF (PIS/ICF Part A) before a tumour tissue sample is obtained (i.e. archived tissue) or undertaken to determine TRBC1 status.

ii. Consent to enter the main study will be sought from patients who are identified as TRBC1 positive. A separate PIS and ICF (PIS/ICF Part B) will be provided.

Sites must ensure that all patients have been given the current approved version of the relevant PIS, are fully informed about the selection process and the trial, and have confirmed their willingness to take part in the selection process by signing the PIS/ICF Part A consent form. Separately, patients must confirm their willingness to take part in the main trial by signing the PIS/ICF Part B consent form. Sites must assess a patient's ability to understand verbal and written information in English or the local language, and whether an interpreter would be required to ensure fully informed consent. If a patient requires an interpreter and none is available, the patient should not be considered for the trial.

The Investigator, or, where delegated by the Investigator, other appropriately trained site staff, is required to provide a full explanation of the screening process and the trial and all relevant treatment options to each patient prior to any trial-specific procedures being conducted. During these discussions, the current and relevant approved PIS should be discussed with the patient.

Sufficient time must be allowed for the patient to consider and discuss participation in the selection process for the trial (PIS/ICF Part A). The patient can also receive and review the PIS/ICF Part B at the same time, but the patient cannot provide consent to the main study until their disease is confirmed as TRBC1 positive. Once the patient is confirmed as having TRBC1 positive T-NHL, adequate time should be given to the patient to review PIS/ICF Part B; however, no minimum time is specified.

Written informed consent on the current and relevant approved version of the ICF(s) for the trial must be obtained before any trial-specific procedures are conducted. The discussion and consent process(es) must be documented in the patient notes.

8.1.2 Patient Registration

Patient registration will be performed after patient consent (PIS/ICF Part A) to enter the screening process and prior to commencement of any trial procedure. Registration is completed using the Registration Form which must be sent to Autolus Limited after the following have been completed:

- Patient written consent to screening (PIS/ICF Part A) for TRBC1 positive T-NHL.
- Provisional confirmation of eligibility of a patient at a site.

The patient information will be reviewed by the Sponsor's Medical Monitor.

8.1.3 Patient Screening for TRBC1 Positive T-NHL

After providing written informed consent to participate in the screening process, the patient will be issued with a unique patient identification number. The patient identification number will be used to identify the patient for the duration of the study. Patient identification numbers will not be reassigned or reused.

The screening for TRBC1 positive T-NHL begins with the provisional assessment of eligibility and obtaining a tumour tissue sample. During this screening process, preliminary eligibility criteria will be reviewed, demographic data (age, gender, race, and ethnicity), lymphoma disease history and a tumour tissue sample (new or archived) will be obtained. The patient's tumour tissue will be analysed for TRBC1 status and TRBC1 positive disease confirmed before written consent is sought and obtained to enter the main study.

Within PIS/ICF Part A, the patient will be asked to consent to the provision of an archival tumour tissue sample or a newly acquired tumour biopsy tissue.

A screening log must be maintained by the site and filed in the Investigator Site File, and data collected during screening will be recorded on the electronic Case Report Form (eCRF). In accordance with Table 24, Section 12.3.1, AEs related to a study screening procedure will be collected.

The screening process to determine TRBC1 status is expected to take up to 28 days.

8.1.4 Tumour Tissue Provision

Following patient consent (PIS/ICF Part A) and registration into the TRBC1 screening process, all patients must provide an archival tumour tissue (FFPE block) or, if not available, patients must undergo a tumour tissue biopsy to provide a newly acquired tumour tissue sample.

The new tumour tissue sample could be either from an excisional biopsy or from a core needle biopsy. An excisional biopsy sample is preferred. If a core needle biopsy is undertaken, a minimum of 2 cores are required for the evaluation of TRBC1 expression on tumour cells using the LymphoTrack Dx TRB Assay on FFPE tissue (see Section 8.1.5). Additional two cores are requested (if medically feasible) for the future development of a TRBC companion diagnostic assay. Core needle biopsies should be preferably taken with a needle of gauge 18 or larger. See pathology manual for additional details and information regarding fixation, storage and shipping.

If available, additional archival tumour biopsy material should be provided for diagnostic assay development and biomarker evaluation (e.g. PD-L1 expression). Archival material can be provided as a FFPE block or slides (see the pathology manual). If the patient is identified as having TRBC1 positive T-NHL, and subsequently receives AUTO4, further tumour tissue biopsies (one between Day 7 and Day 21 after AUTO4 administration and at progression of disease) are requested if a suitable lesion is available. These are required for biomarker (such as PD-L1 expression) evaluation and for the presence of TRBC1-CAR T cells. If emerging safety and efficacy data suggest further investigation is warranted, bone marrow sampling may be requested.

Tumour lesions used for newly acquired biopsies should not be the same lesions used as Lugano Classification target lesions, unless there are no other lesions suitable for biopsy.

8.1.5 NGS Assay Used to Select TRBC1 Positive Patients for Entry

TRBC1 expression on FFPE tumour biopsy tissue will be evaluated using the LymphoTrack Dx TRB Assay – MiSeq to identify clonal T cell receptor beta (TRB) gene rearrangements using Next-Generation Sequencing (NGS) with the Illumina MiSeq. There is a cluster of TRBV genes that are shared by both types of TCR-β chain. Located downstream of the TRBV, there are two TRBD-TRBJ-TRBC clusters which are TRBD1-TRBJ1-TRBC1 followed by TRBD2-TRBJ2-TRBC2 with a 2.6 kb intergenic region in between. The VDJ recombination occurs at the genomic level, while at the transcription level, the rearranged VDJ is joined with TRBC1 or TRBC2 via splicing. Autolus has previously demonstrated through NGS analysis of healthy human T cells, that in most cases, TRBJ1 links to C1 (99.9%) and TRBJ2 links to C2 (99.99%) (Table 9). This forms the rationale for genotyping the TCR-β-related VDJ in tumour containing tissue samples via NGS and using the information of the clonal VDJ rearrangement to indicate if the tumour expresses TRBC1 or TRBC2.

In brief, a minimum of six and a maximum of fifteen 5µm sections will be cut from the archival or newly acquired FFPE biopsy tissue and placed into Eppendorf tubes. Genomic DNA will be extracted and purified from the tissue. The extracted and purified genomic DNA will then be placed into a single multiplex master mix for a PCR reaction. The PCR amplicons will then be purified to remove excess primers, nucleotides, salts and enzymes using the Agencourt AMPure XP system. The purified amplicons are quantified using the KAPA Library Quantification Kit for Illumina Platforms. Finally, 600 µL of the final prepared library will be loaded onto a MiSeq Reagent Cartridge and the MiSeq run will be started. The sequencing data can be analyzed using the LymphoTrack Dx Software-MiSeq package. The merged Read Summary Report should be used to identify the top merged read sequences and their frequencies.

A patient will be identified as having a TRBC1 positive T-NHL if the tumour sample is determined to be clonal by the LymphoTrack Dx TRB Assay – MiSeq; where the presence of a J1 sequence determines C1 positivity.

Table 9: Distribution of TRBJ and TRBC in Tonsil TCR-β Transcripts

Distribution	Number of Reads	Percentage Among All Reads
TRBJ1 – TRBC1	1,653,275	40.37
TRBJ2 – TRBC2	2,437,579	59.52
TRBJ1 – TRBC2	4,206	0.1
TRBJ2 – TRBC1	360	0.0088

8.1.6 Patient Screening to the Main Study

Once a patient is identified as having TRBC1 positive T-NHL, the remaining screening procedures can be conducted following written informed consent to participate in the main study (PIS/ICF Part B). The remaining screening (including the baseline disease assessment) and other procedures will be performed and are likely to take at least 8 weeks (56 days). The total screening window (from initial consent Part B to AUTO4 administration) is up to 12 weeks (84 days).

If an assessment is or has been performed as part of the patient's routine clinical evaluation and not specifically for this study, it does not need to be repeated after signed informed consent

has been obtained, provided that the assessments fulfil the study requirements and are performed within the specified timeframe prior to the AUTO4 administration.

With the exception of re-test for TRBC1 positive status, the re-testing of abnormal screening values that lead to exclusion is allowed during the screening phase (to reassess eligibility). On Day -7 (-1 day), the patient should continue to meet renal, hepatic, pulmonary function and performance status requirements prior to initiating pre-conditioning chemotherapy. On Day 0, before infusion, it will be assessed whether the patient meets the AUTO4 infusion criteria. The measurements collected at the time closest to, but prior to, the administration of AUTO4 will be defined as the baseline values for safety assessment and treatment decisions. Adverse events associated with screening procedures will be collected. Baseline data will be collected during screening as described in the Schedule of Assessments.

8.1.7 Infectious Disease Screening

Patients will be tested by the site (in accordance with the site's Human Tissue Authority license for human application) for infectious diseases as outlined below prior to (but within 30 days of) leukapheresis; and then, tested again on the day of leukapheresis or within 7 days after leukapheresis. Table 10 lists the tests that must be performed as a minimum requirement.

Table 10: Infectious Disease Screening

Pathogen	Test
HIV 1 and 2	Anti-HIV-1 and 2
Hepatitis B	Hepatitis B surface antigen, anti-hepatitis B core antibody
Hepatitis C	Anti-hepatitis C virus antibody
Syphilis	Syphilis serology (chemiluminescent microparticle immunoassay)*
HTLV 1 and 2	Anti-HTLV-1 and 2

HIV = human immunodeficiency virus; HTLV = human T cell lymphotropic virus.
* A validated testing algorithm must be applied to exclude the presence of active infection with Treponema pallidum. A non-reactive test, specific or non-specific, can allow tissues and cells to be released. When a non-specific test is performed, a reactive result will not prevent procurement or release if a specific Treponema confirmatory test is non-reactive. A patient whose specimen tests reactive on a Treponema-specific test will require a thorough risk assessment to determine eligibility for clinical use.

8.2 LEUKAPHERESIS FOR AUTO4 MANUFACTURING

Following completion of all the procedures and assessments required in 'Screen 1' (including the confirmation that the patient is TRBC1 positive), patients will undergo an unstimulated leukapheresis for the generation of AUTO4.

Leukapheresis must be performed within 30 days of infectious disease testing and will be done following the standard institutional processes. Sites may choose to take the blood sample for infectious disease screening at the time of the tumour tissue biopsy to ensure the infectious disease screen result is known within 30 days before leukapheresis. An additional sample is to be taken for a second infectious screen on the day of leukapheresis (or within 7 days after).

In general, leukapheresis should be performed at least 35 days before the planned AUTO4 dosing date, as AUTO4 manufacture and release takes approximately 1 month. Based on emerging data this window can be changed. If the product is ready early the patient may receive the product early.

The leukapheresate is the starting material for the manufacture of the ATIMP, AUTO4. The total cell number that is required for successful manufacture varies according to the dose level. Typically, a double volume leukapheresis will be performed. The target collection is between 1 x 10^9- 5 x 10^9 PBMCs. If the collection is insufficient, the Sponsor will advise as to the feasibility of successful manufacture. If collection is determined to be inadequate, then collected cells may be used for research purposes as per the patient informed consent. The leukapheresate will be transported to the Sponsor for generation of AUTO4 at a temperature of 2 to 8°C as soon as possible and within 48 hours, ideally within 24 hours. Further details regarding this process can be found in the CHIM.

Bridging chemotherapy may be prescribed to the patient during the AUTO4 manufacturing period at the discretion of the Investigator and in accordance with the exclusion criteria and washout periods outlined in the eligibility criteria (see Section 6.2). Each leukapheresate will be identified by a unique patient identification number plus any additional patient identifiers as allowed per local regulations (typically initials and date of birth).

Upon release of AUTO4 product, and if the patient continues to meet the eligibility criteria for entry to the study (as outlined in Section 6), the site will liaise with the manufacturer to arrange transfer of the AUTO4 product to the participating site. Further details are provided in the CHIM.

However, if a patient is withdrawn after the successful leukapheresis and manufacture of AUTO4, but is subsequently re-screened and eligible for treatment, it may not be necessary to repeat leukapheresis and manufacture of AUTO4. To be discussed with the Sponsor.

8.2.1 Leukapheresis for Immune Reconstitution (Rescue Treatment Storage)

Where feasible, a fraction of leukapheresate (minimum 1 x 10^6 lymphocytes/kg) should be stored as a backup (rescue treatment) for immune reconstitution. This apheresate can be collected at the same time as the primary leukapheresis described above (in a different bag) or at a second unstimulated leukapheresis prior to pre-conditioning. This apheresate should be processed, frozen and stored at the local institution using the local standard labelling, freezing and storage process. If, and when necessary, these cells should be released for immune re-constitution following depletion of AUTO4 using Rituximab. These cells will be thawed and infused as per the local institutional standards for autologous cells.

8.3 PRE-CONDITIONING CHEMOTHERAPY

Patients that still meet eligibility requirements for the study and for whom the AUTO4 product has been release to specification, will proceed to receive a lymphodepleting pre-conditioning treatment with Fludarabine (FLU) and Cyclophosphamide (CY) before AUTO4 infusion. The pre-conditioning phase will begin with administration of pre-conditioning chemotherapy and will end with the beginning of treatment with AUTO4 infusion. During this phase, AEs associated with pre-conditioning chemotherapy as well as use of concomitant medications will be collected.

Prior to administration of pre-conditioning chemotherapy, patients will undergo clinical and laboratory assessments as per the Schedule of Assessments and the site Investigator or designee will determine if the patient is fit to receive pre-conditioning chemotherapy. If considered to be fit, patients will proceed to receive a lymphodepleting pre-conditioning treatment with FLU and CY for 4 days (starting Day -6 [-1 day]) and timed to end 3 days (-1 day) before AUTO4 infusion.

8.3.1 Pre-conditioning Chemotherapy Dose and Regimen

Patients will receive FLU and CY according to the dosing described below; FLU will be given first.

- Day -6: FLU 30 mg/m^2 followed by CY 500 mg/m^2
- Day -5: FLU 30 mg/m^2 followed by CY 500 mg/m^2
- Day -4: FLU 30 mg/m^2
- Day -3: FLU 30 mg/m^2

The pre-conditioning chemotherapy should be completed a minimum of 3 days (-1 day) prior to AUTO4 infusion.

Fludarabine will be given by i.v. infusion over 30 minutes in sodium chloride 0.9%. For patients with renal impairment (glomerular filtration rate 30 to 60 mL/min/1.73 m^2 [corrected]), the dose of FLU should be reduced per routine clinical practice (generally by 25%).

Cyclophosphamide will be given by i.v. infusion over 30 minutes. Adequate pre- and post-hydration for up to 4 to 6 hours (or as per institutional practice) should be given post-infusion to induce diuresis. Use of mesna for the prescribed dose is generally considered unnecessary but may be considered based on institutional practice. Cyclophosphamide dose may be reduced if the leukocyte count is <2500 cells/μL with 6 hours post-hydration.

Anti-emetic prophylaxis will be given as per standard institutional policy. Prophylaxis for TLS with allopurinol and i.v. fluids may be given if clinically necessary

For additional information, please refer to the FLU and CY SmPCs.

8.3.2 Supply of Fludarabine and Cyclophosphamide

Fludarabine and CY are non-investigational medicinal products as they are not being tested or used as a comparator in this trial. Fludarabine and CY will be used to induce lymphodepletion in the current study as a pre-conditioning treatment, prior to AUTO4 treatment. Both drugs are authorised and commercially available in the UK and EU. Investigators will be responsible for their own supply of FLU and CY, sourced from their institution. Sufficient quantities of FLU and CY will be dispensed to cover the prescribed dose and will be prepared as per site Standard Operating Procedures and according to manufacturer recommendations. Fludarabine and CY are cytotoxic and must be handled with care in accordance with local policy. Good aseptic practice must be employed when preparing FLU and CY solutions for infusion. Further details may be found in the SmPCs for FLU and CY.

8.3.3 Accountability of Fludarabine and Cyclophosphamide

Pharmacy records should be kept of the FLU and CY dispensed to trial patients. The expiry date, batch number used, and manufacturer should be recorded as well as details of vials dispensed and returned.

8.4 AUTO4 TREATMENT AND PATIENT MONITORING

The treatment phase will involve infusion of AUTO4 on Day 0. The patient will be admitted to hospital for at least 14 days for monitoring and management of CRS and other toxicities.

The actual time in hospital will be clinically determined depending on the patient's toxicity profile.

8.4.1 Assessment Prior to AUTO4 Infusion

Prior to administration of AUTO4, patients will undergo clinical and laboratory assessments and the site Investigator or designee will determine if the patient is eligible to receive AUTO4 as per protocol Section 6 (See Schedule of Assessments for details). For patients who receive bridging therapy, baseline disease assessment with PET/CT should be done after completion of bridging therapy and before preconditioning and AUTO4 infusion.

If a patient is not eligible to receive infusion of AUTO4 (Section 6.2), the AUTO4 infusion will be delayed. Infusion may be performed at a later date, as per dose delay guidelines (Section 8.4.5) or cancelled.

8.4.2 AUTO4 Administration

AUTO4 will be administered as a single rapid infusion on Day 0 in an in-patient setting. Full details will be provided in the CHIM. Only the Investigator or Investigator's designee will dispense the study product.

Premedication with diphenhydramine/chlorpheniramine and paracetamol/acetaminophen may be given prior to infusion of AUTO4 as per standard institutional practice (see Section 10.2), but steroids should not be given as part of premedication.

The Investigator or Investigator's designee along with a second person (e.g., research nurse) will verify that the patient details (Unique Patient Number) on the AUTO4 label matches the recipient, check the dose of cells, volume, and number of cryobags to be infused and prescribe the AUTO4 on a blood product chart. Details of the exact dose, volume (from label), time of completion of thawing and time of completion of infusion will be documented in the applicable study records.

AUTO4 will be infused as described in the CHM. In brief:

- AUTO4 will be thawed rapidly in a 37°C water bath under sterile conditions.
- The entire contents of the bag(s) will be given as an i.v. infusion using a syringe or gravity aided infusion through a central or large bore peripheral venous access over **a few minutes** (maximum 30 minutes from AUTO4 being thawed to preserve cell viability).
- **A leukodepleting filter MUST NOT be used for the infusion of the T cell product.**
- A nurse or physician experienced in the administration of cellular blood products as per the trial-specific procedure.
- The infusion line and the bag(s) should be flushed as described in the CHM to ensure all cells have been administered.
- If there are multiple bags of cell product, one bag should be thawed and safely infused before the second one is thawed.
- If the AUTO4 cell product appears to have a damaged or leaking bag, or otherwise appears to be compromised, it should not be infused.

Although not routinely practiced, there may be circumstances (e.g. when AUTO4 is administered by a site for the first time), when Sponsor representatives may be present to

observe the thawing and/or dosing of AUTO4. These individuals will have no participation in the preparation of the product nor in any part in the patient's medical care. This will only occur if the practice is allowed by local IRB/Ethics Committee and institutional guidelines and the consent of the patient. This may also allow the sponsor to collect best practices and further refine dose administration.

The time between completion of thawing and completion of infusion should not exceed 30 minutes.

8.4.3 Monitoring During and After Drug Administration

Patients are intended to stay as in-patients for at least 14 days following the cell infusion (longer if clinically necessary). All patients will be monitored closely with temperature, pulse/heart rate, blood pressure, respiratory rate, and oxygen saturations immediately prior to and every 30 minutes (±10 minutes) for the next 4 hours after AUTO4 infusion. In the event of allergic adverse reactions, anti-histamines may be administered, as well as oxygen and salbutamol in the event of respiratory distress.

During hospitalisation care, patients will be monitored at a minimum 1 to 2 times daily. The patients will undergo blood tests (see Schedule of Assessments) for signs of toxicity, in particular for CRS, TLS and neurological disturbance. Transfusions of blood products, antibiotics, analgesics, and intensive care will also be provided as clinically indicated. Prophylaxis for TLS may be initiated if the risk is considered high or for all new patients if TLS occurs at a certain dose level. Urate levels will be monitored and treatment started if indicated.

Patients may be discharged from hospital when clinically appropriate or following successful management of CRS. The in-patient stay may be extended if deemed necessary by the treating physician.

Following discharge from the hospital, the patient will be closely monitored for AEs and laboratory abnormalities, at least on a weekly basis. The required study procedures and assessments to be conducted during the Treatment Stage are outlined in the Schedule of Assessments. Clinical evaluations and laboratory studies may be repeated more frequently, if clinically indicated. Provided that all criteria are satisfied (see Section 8.4.8), patients may receive re-treatment (second cycle) of AUTO4.

After completing the study (Month 24, post AUTO4 administration), or after disease progression, patients will roll over into a separate long-term follow-up protocol (AUTO-LT1) if informed consent is provided.

8.4.4 Monitoring for Infections

Patients should receive Pneumocystis prophylaxis with trimethoprim-sulfamethoxazole or suitable alternative agents, and either acyclovir or valacyclovir for herpes virus prophylaxis from the start of conditioning chemotherapy until at least 6 months (or longer if clinically indicated) post AUTO4 infusion.

It is likely that in the first few weeks following pre-conditioning chemotherapy patients will be neutropenic and pan lymphopaenic. The patients will be monitored for infections in accordance with the Schedule of Assessments; specifically, patients will be monitored for viral infections cytomegalovirus (CMV), Epstein Barr virus (EBV) and adenovirus. Other monitoring, such as

John Cunningham virus (JCV), toxoplasmosis and fungal infections should be per institutional guidelines used for bone marrow transplant patients or as clinically indicated.

All patients with fevers while neutropenic must have blood cultures drawn and receive broad-spectrum antibiotics as per institutional practice.

8.4.5 Dose Delay of Pre-conditioning and AUTO4 Treatment

If the pre-conditioning regimen is interrupted for intercurrent illness or other reasons, the patient may complete or recommence the preparative regimen after recovery, or proceed with partial pre-conditioning according to the Investigator's judgment after consultation with the Sponsor. Patients will be closely monitored during and after the pre-conditioning regimen.

If a patient is unable to be dosed with AUTO4 on the planned day, they may undergo delayed dosing after having received the pre-conditioning chemotherapies again (if appropriate – and if they continue to meet the study enrolment criteria). Imaging studies may not need to be repeated if the patient has not received any other anti-lymphoma therapy in the interim (excluding steroids and pre-conditioning chemotherapy). Patients undergoing delayed dosing may be evaluable for dose escalation decision making if the SEC so concludes.

If the patient is deemed unsuitable to receive AUTO4, they will be discontinued from the clinical trial (see Section **Error! Reference source not found.**) and replaced. Each case will be discussed with the Sponsor.

8.4.6 Interruption of Infusion

In the event of severe (≥Grade 3) infusion reaction, the FLU-CY or AUTO4 infusion should be stopped and the patient treated as clinically indicated. When the patient has recovered, the infusion may be restarted.

Interruption of AUTO4 should not be greater than 30 minutes after thawing of AUTO4. If an infusion is interrupted for mechanical, technical or any other reason, then this should be dealt with per local practice and the infusion restarted as soon as possible. In case of uncertainty, individual cases should be discussed with the Sponsor.

If the patient develops an allergic reaction to the drugs in the pre-conditioning regimen, alternative drugs as per the institutional practice may be considered after discussion with the Sponsor.

8.4.7 Duration of Treatment

In most patients, it is expected that AUTO4 will be given once. However, if a patient has sufficient AUTO4 remaining from the original manufacture and meets the re-treatment criteria, a second treatment may be given (see re-treatment of patient Section 8.4.8).

8.4.8 Re-treatment of Patients

It is expected that most patients will receive a single dose of AUTO4, as part of their treatment. However, some patients may qualify for a re-treatment upon treating physician request. This re-treatment may be for patients in whom there has been no CAR T- cell engraftment (e.g absence or low levels of CAR T-cell expansion), and could use either remaining CAR T-cells from the initial manufacturing process (if there is AUTO4 product leftover), or by a new AUTO4 manufacturing (e.g repeating the leukapheresis procedure and manufacturing process),

if the patient clinical status allows (per treating physician decision). Specific criteria for re-treatment are described below and individual risk-benefit considerations should be taken into account.upon Sponsor and treating physician discussion

Prior to re-treatment, the patient must meet the following criteria:

1 Circulating levels of AUTO4 are low (<0.2 x 10^9/dL AUTO4 cells) or undetectable and no significant anti-tumour effect (no CR) after the first dose, and the first treatment dose is considered to be sub therapeutic.

OR

2 There was objective clinical evidence of anti-tumour activity following the previous AUTO4 infusion (i.e. Stable Disease or better).

OR

3 The patient has evidence of progressive disease in the context of declining levels of AUTO4. Circulating levels AUTO4 cells must be low (<0.2 x 10^9/dL) or undetectable for at least 2 weeks prior to the second infusion.

AND all of the following:

4

5 The patient tolerated the first infusion without dose-limiting or other severe (≥Grade 4) or unmanageable toxicity for a follow-up period of at least 28 days.

6 The patient still fulfils the trial entry criteria required to tolerate another pre-conditioning treatment and AUTO4 infusion.

7

Patients undergoing a second AUTO4 infusion should receive the same pre-conditioning chemotherapy. The dose of AUTO4 can be at (or up to) the highest dose that has been declared safe with at least three patients completing the DLT period (or at least 1 patient in case there is any single patient dose cohort) and is considered to be more likely to be more active than the patient’s first dose of AUTO4. Depending on the number of cells available, an intermediate dose may also be administered if considered appropriate. The decision to re-treat a patient will be made by the treating Investigator and Sponsor in consultation with the SEC. Patients re-treated will be monitored in a similar way to patients being treated for the first time, in that they would start evaluation and management as defined in the protocol, starting from the pre-conditioning stage.

8.5 FOLLOW-UP PHASE

Upon completion of the first 28 days following AUTO4 infusion, patients will enter the Follow-up Phase where they will continue to be followed as per the schedule of assessments for the duration of the study (Table 1) or until early withdrawal or death whichever is the latest for the assessment of anti-tumour response and or safety evaluation.

8.5.1 Efficacy and Safety Follow-up

After AUTO4 infusion, all patients will be followed up for efficacy and safety. Patients will have periodic monitoring of response based as described in the Schedule of Assessments

(Table 1). Patients who proceed to hematopoietic stem cell transplantation or any other anti-T-cell lymphoma therapies while in remission will also be monitored for efficacy and safety until the EoS. Patients will be invited to participate in the long-term follow-up study at EoS.

Relapse/Disease Progression evaluation: If at any time during the Efficacy and Safety Follow-Up, a patient experiences disease progression a full disease evaluation will be completed.

Any patients in CR who have completed the 24 months follow-up period post first AUTO4 infusion, will be monitored thereafter every 6 months until the EoS as per the **Schedule of Assessments 1**, including:

- Survival, limited concomitant medication (e.g. therapeutic doses of steroids, inmunossuppressive, medications AE-related, any T-cell Lymphoma treatments), all SAEs and AEs considered related to the AUTO4, AEs of special interest and any new malignancy.
- Disease response until the end of the study or until the patient experiences disease progression (whichever occurs first).
- Once a year for AUTO4 vector persistence and RCR.
- T-cell Subset Analysis (local) – every 6 months until end of study

8.5.2 Safety and Survival Follow-up

Patients who fail to respond (within 2 months post AUTO4 treatment) to treatment or who respond but subsequently relapse or progress will continue to be monitored for safety and survival through to EoS as per the safety and survival Schedule of Assessments 2 (Table 2). At EoS, the patients will complete an EoS visit and will be invited to participate to the long-term follow-up study.

The first assessment for the safety and survival follow-up will happen at the next planned visit as per the safety and survival follow-up **Schedule of Assessments 2**.

During the safety and survival follow-up, patients will come to the site as per the Schedule of Assessments. If a patient cannot attend a scheduled visit, the information can be collected over the phone and the Investigator should attempt at a minimum to determine the survival status and whether the patient has started receiving additional antineoplastic therapies and associated response to new treatment.

In the event that a patient still in response is no longer willing to be followed up for efficacy and safety anymore but however agrees to remain on study to be monitored for safety and survival until completion of the study, the Investigator should attempt at a minimum to determine the relapse/disease progression status and whether the patient has started receiving additional antineoplastic therapies.

The efficacy data collected as per the local standard of care may recorded in the clinical database until they experience disease progression/relapse or rollover to the long-term follow-up study.

Selected AEs and concomitant medications will be recorded, please refer to Section 12.3.

Patients who are discontinued from the study will complete the EoS visit. The reason for discontinuation will be documented. For patients who are lost to follow-up, the Investigator should show “due diligence” by documenting in the source documents steps taken to contact the patient, e.g. dates of telephone calls, registered letters, etc. Any patients who progressed post AUTO4 infusion and have completed the 24 months follow-up period will be monitored every 6 months until the EoS as per the Schedule of Assessments (Table 2). They will be followed-up for:

- Survival, all SAEs and AEs considered related to AUTO4, AEs of special interest and any new malignancy
- Subsequent therapy and response if applicable
- Once a year for AUTO4 vector persistence and RCR

If a patient misses a scheduled visit, or if the visit time-point where survival status is required does not align with a scheduled visit, survival status can be obtained via phone contact with the patient or correspondence with local health care provider.

8.6 LONG-TERM FOLLOW-UP

If a patient signs a new ICF for entry to the long-term follow-up study, they will be enrolled into a long-term follow-up study after treatment of AUTO4-TL1 on completion of the study. They will be monitored for SAEs considered related to the study treatment, AESIs and new malignancy for a period of up to 15 years following last treatment with AUTO4. See Sections 15.1 and **Error! Reference source not found.**.

9 STUDY ASSESSMENTS

9.1 DEMOGRAPHIC AND BASELINE ASSESSMENTS

The following will be collected at Screen 1 and Screen 2 to determine eligibility and baseline status of the patient.

9.1.1 Demographic Data and Baseline Variables (Screen 1)

Demographic data

- Demographic data will include self-reported race/ethnicity, age, gender, and height at screening visit.

Medical/lymphoma history

- Medical history includes all current and past clinically significant diseases, surgeries, cancer history (including prior cancer therapies and procedures) and medications (e.g., prescription drugs, over-the-counter drugs, herbal/homeopathic remedies, nutritional supplements). Histological confirmation of disease diagnosis will be obtained (pathology report).

Physical examinations and investigations:

- Physical examination (see Section 9.2.6)
- Weight, vital signs (see Section 9.2.5)
- Electrocardiogram (ECG – see Section 9.2.3), ECHO or MUGA (Section 9.2.4)
- ECOG (see Section 9.2.8)
- Laboratory (haematology, coagulation and biochemistry – Section 9.2.2)

Pregnancy test

- Serum (β-human chorionic gonadotropin) or urine pregnancy testing will be performed for females of childbearing potential at the screening visit and will be repeated when the patient is admitted prior to starting pre-conditioning treatment, and prior to AUTO4 infusion. Following AUTO4 infusion, pregnancy testing will be performed at Day 28, Month 3, Month 6 and Month 12 as described in the Schedule of Assessments.

9.1.2 Tumour Tissue Biopsy and TRBC1 Status at Baseline (During Screen 1)

The patient must provide tumour tissue from an archival biopsy or a new biopsy for the evaluation of TRBC1 status (see Section 8.1.4). The TRBC1 status of the lymphoma tumour tissue will be determined by a central laboratory, and the TRBC1 status must be known prior to leukapheresis.

9.1.3 T-cells subsets analysis

Local assessment of peripheral blood T-cell subsets levels is required according to the SoA (Table 1). Documentation of the absolute number of the total lymphoid events in the sample analysed, as well as absolute numbers of CD3+, CD3+CD4+, CD3+CD8+ cells are the minimum requirements.

9.1.4 Efficacy Scans

Patients will undergo baseline scan(s) – CT imaging of the neck, chest, abdomen and pelvis. MRI may be used to evaluate sites of disease that cannot be adequately imaged using CT (in cases where MRI is desirable, the MRI must be obtained at baseline and at all subsequent response evaluations). For all other sites of disease, MRI studies do not replace the required neck, chest, abdomen, and pelvic CT scans. Brain MRI is only required if clinically indicated. A positron emission tomography (PET) scan will additionally be conducted.

A PET scan will be considered a baseline PET scan if the patient did not receive any treatment for T-cell lymphoma between the date on which the PET scan was performed and the start of preconditioning therapy. If patients received any treatment for T-cell lymphoma between the screening period and the start of preconditioning the PET scan needs to be repeated after all treatment is completed and the result should be entered in the CRF as the baseline disease assessment.

Specifically for those patients receiving a bridging chemotherapy regimen, the baseline PET/CT scans must be done after completion of bridging therapy and before preconditioning and AUTO4 infusion.

9.2 SAFETY EVALUATIONS

All patients who receive AUTO4 will be considered evaluable for toxicity assessment. Any clinically relevant changes occurring during the study must be recorded on the AE section of the eCRF. Safety assessments will be based on medical review of AE reports and the results of vital sign measurements, ECGs, physical examinations, clinical laboratory tests, and performance status assessments at specified time points as described in the Schedule of Assessments. Any clinically significant abnormalities persisting at the end of the study/early withdrawal will be followed by the Investigator until resolution, or until a clinically stable endpoint is reached. The study will be monitored by the SEC and IDMC (details regarding the SEC and IDMC are provided in Section 13). The study will include the following evaluations of safety and tolerability according to the time points provided in the Schedule of Assessments.

9.2.1 Adverse Events and Toxicity

Adverse events will be reported by the patient (or, when appropriate, by a caregiver, surrogate, or the patient's legally acceptable representative) for the duration of the study. Adverse event recording and reporting is described in detail in Section 12.3.1.

Toxicity will be graded using the NCI CTCAE version 5.0 criteria and collected according to Section 12.3.1.

The incidence of AEs will be tabulated and reviewed for potential significance and clinical importance.

9.2.2 Clinical Laboratory Tests

Blood samples for haematology, coagulation and biochemistry will be collected at each visit as specified in the Schedule of Assessments. Where appropriate, tests must be performed prior to receiving pre-conditioning chemotherapy or AUTO4 infusion. More frequent clinical laboratory tests may be performed if indicated by the overall clinical condition of the patient or by abnormalities that warrant more frequent monitoring. Screening laboratory results must be available to the Investigator for evaluation before AUTO4 infusion and subsequent

laboratory results should be available at the time of the patient's evaluation by the treating physician. The Investigator must review the laboratory reports, document this review, and ensure that any clinically relevant changes occurring during the study are recorded in the AE section of the eCRF. The laboratory reports must be filed with the source documents. A summary of the tests that will be performed by the local laboratory is presented in Table 11.

Table 11: Clinical Laboratory Tests

Assessment	Description
Haematology	Haemoglobin, red blood cell count, platelet count, white blood cell count with differential (neutrophils, eosinophils, lymphocytes, monocytes, and basophils). All tests must be performed prior to pre-conditioning chemotherapy, and prior to AUTO4 infusion on Day 0 of any treatment stage.
Coagulation	Prothrombin time, international normalised ratio, activated partial thromboplastin time, fibrinogen. Tests must be performed as per the Schedule of Assessments, including Day 7 after AUTO4 infusion.
Biochemistry (full panel)	Sodium, phosphate, potassium, magnesium, chloride, bicarbonate, alanine aminotransferase, aspartate aminotransferase, serum uric acid (on Days 0, 1 and 7 of AUTO4 treatment stage), urea or blood urea nitrogen, creatinine, serum creatinine phosphokinase, lactate dehydrogenase, glucose, total bilirubin, calcium (albumin adjusted), total protein, albumin. On Day 0, all tests must be performed prior to AUTO4 infusion.
Ferritin, C-reactive protein	Ferritin, C-reactive protein as per the Schedule of Assessments and as clinically necessary and during CRS if necessary.
Pregnancy test	Serum (β-human chorionic gonadotropin) or urine pregnancy testing for females of childbearing potential.
Infectious Disease Screen - Serology (at screening and then repeated at leukapheresis or within 7 days after leukapheresis)	• HIV antibody.<br>• Hepatitis B core antibody: if positive, further testing (DNA by PCR) to rule out active disease or chronic carrier. Must be confirmed negative prior to screening.<br>• Hepatitis C virus antibody: if positive for hepatitis C virus, further testing (by ribonucleic acid PCR) should be performed to rule out active infection.<br>• Anti-HTLV-1<br>• Anti-HTLV-2<br>• Syphilis serology.
Monitoring for infection.	• CMV, EBV & adenovirus. Other, such as JCV, toxoplasmosis and fungal pathogens will be monitored as per institutional guidelines for bone marrow transplant patients, or as clinically necessary. Monitoring beyond 3 months should be done if there is complete T cell aplasia or if clinically indicated.

DNA = deoxyribonucleic acid; HIV = human immunodeficiency virus; HTLV = human T cell lymphotropic virus; PCR = polymerase chain reaction.

9.2.3 12-lead Electrocardiogram

A 12-lead ECG will be obtained using an ECG machine that automatically calculates the heart rate and measures PR, RR, QRS, QT, and corrected QT intervals. Refer to the Schedule of Assessments for details regarding the frequency of ECG assessments. At each time point, a single 12-lead ECG will be performed by qualified site personnel. The clinical Investigator or designee will review the printout, including ECG morphology. The ECG should be repeated in triplicate if motion artefacts or clinically relevant abnormalities are noted. Additional cardiovascular assessments should be performed as clinically appropriate and when patient

experiences CRS to ensure patient safety. If blood sampling or vital sign measurement is scheduled for the same time point as ECG recording, the procedures should be performed in the following order: ECG(s), vital signs, then blood draw.

9.2.4 Echocardiogram or Multiple Gated Acquisition Cardiac Scan

Echocardiogram is the preferred method to assess cardiac ejection fraction and cardiac valve abnormalities; MUGA cardiac scan is an acceptable alternative. Assessments should be performed at screening and additional assessments may be performed at the onset of CRS and/or when clinically indicated. Generally, for a patient, the same procedure should be performed at screening and at any follow-up assessment to allow direct comparison.

9.2.5 Vital Signs

Vital signs will include temperature, pulse/heart rate, oxygen saturation, respiratory rate, and blood pressure (systolic and diastolic), and weight. Blood pressure and pulse/heart rate measurements should be recorded with the patient in a seated position or supine. Multiple time points (a minimum of three) will be collected prior to treatment to establish a good baseline blood pressure for the patient. Please refer to the Schedule of Assessments for timings and frequency of measurement. Blood pressure and pulse/heart rate measurements will be assessed with an automated device. Manual techniques will be used only if an automated device is not available.

9.2.6 Physical Examination

A complete physical examination and complete neurological examination will be conducted at screening as per the institutional standard practice. Thereafter, a symptom-directed physical examination will be conducted at subsequent visits. The schedule for physical and neurological examinations is provided in the Schedule of Assessments.

9.2.7 Concomitant Medications

Concomitant medications are to be collected as described in the Schedule of Assessments.

9.2.8 ECOG Performance Status

The ECOG scale provided in Appendix 2 will be used to grade changes in the patient’s daily living activities. The frequency of ECOG assessments is provided in the Schedule of Assessments.

9.3 PHARMACOKINETICS, PHARMACODYNAMICS AND BIOMARKER EVALUATION

Blood-based pharmacodynamics biomarkers will be evaluated in all patients as described in the Schedule of Assessments. Peripheral blood biomarkers may be assessed pre- and post-AUTO4 treatment. Assessment at additional or fewer time points may be performed based on emerging data. Details regarding sample collection and processing are provided in the laboratory manual.

9.3.1 Evaluation of AUTO4 Persistence in Peripheral Blood

Two validated assays will be used to measure the expansion/persistence of RQR8/aTRBC1-CAR positive T cells at the time points indicated in the Schedule of Assessments. Flow cytometry will be used to measure the frequency of RQR8/aTRBC1-CAR positive T cells per microliter of whole blood and/or a PCR assay will be used to quantify the number of copies of the RQR8/aTRBC1-CAR transgene per microgram of genomic DNA and/or per cell in peripheral blood. Please refer to the AUTO4-TL1 laboratory manual for the handling and storage of samples.

9.3.2 Evaluation of AUTO4 Persistence and TRBC1 Expression in Lymph Node Tumour

When possible, a lymph node tumour biopsy sample should be sent to the Sponsor for the assessment of presence of CAR T-cells and TRBC1 expression in the tumour. These assessments will be performed using flow cytometry or immunohistochemistry. Biopsy samples should be taken at the timepoints outlined in the Schedule of Assessments (Table 1, Footnote 9).

9.3.3 Evaluation of RCR in Peripheral Blood

As per health authorities' guidelines, tests will be performed to evaluate and monitor the presence of RCR by PCR in whole blood or PBMCs. Please refer to the AUTO4-TL1 laboratory manual for the handling and storage of samples.

9.3.4 TRBC1 Positive T Cell Aplasia

AUTO4 will target polyclonal TRBC1+ T-cells. If AUTO4 expansion is observed, TRBC1 positive T-cells aplasia may occur. Blood samples will be collected for the analysis of the levels of TRBC1 positive and TRBC1 negative T cell subsets (including those that are CD4+ and CD8+) in accordance with the Schedule of Assessments (Table 1). Samples will be analyzed centrally with a validated Flow Cytometry assay.

9.3.5 Insertional Mutagenesis

Blood samples will be stored but not analysed (unless clinical evidence dictates) as per the Schedule of Assessments for insertional mutagenesis unless there is evidence that AUTO4 is no longer present. The result will allow identification of any potential relationship between AUTO4 treatment and the development of any new malignancy.

9.3.6 Exploratory Biomarker Assessments

Serum cytokine profile: The serum cytokine profile (using a minimum dataset of TNF-α, interferon-γ, and IL-6) will be measured using a highly sensitive, reproducible, and validated cytokine assay at time points indicated in the Schedule of Assessments. Additional samples may be taken where clinically indicated, for example during CRS. Blood samples for cytokine measurements may be frozen and batched for analysis or assayed using fresh serum. Serum may also be used for measurement of other biomarkers as appropriate.

Immunological/genomic phenotyping: PBMCs may be isolated from whole blood following standard procedures and cryopreserved in liquid nitrogen for later immunological assessment

or assessed immediately. Samples will not be stored for more than 15 years. PBMCs may be used for various immunological assessments such as phenotyping by flow cytometry, genomic analysis and other assays as developed. Immunophenotyping of PBMCs will be evaluated at selected time points (per the Schedule of Assessments) and dependent upon a minimum frequency of RQR8/aTRBC1-CAR positive cells

TRBC1 expression on lymphoma tissue: If sufficient FFPE tumour tissue is provided at baseline, the expression of TRBC1 on lymphoma cells may be evaluated by IHC.

PD-L1 expression on lymphoma cells: If sufficient FFPE tumour tissue is provided at baseline, the expression of PD-L1 on lymphoma cells may be evaluated by IHC, and where possible from tumour tissue samples collected between Day 7 and 21 and/or at progression of disease.

If a patient is declared to have disease progression outside of scheduled time points, blood and tumour tissue/bone marrow samples will be collected to help elucidate the reasons for primary or acquired resistance using appropriate methods.

9.3.7 Immunogenicity Analysis

Detection of human anti-CAR T cell responses and antibodies, or related antibodies, may be measured in cryopreserved PBMCs and serum. Serum or plasma samples at selected time points, for example at Day 0, end of DLT evaluation period and Month 3 to 6, may be analysed if clinically indicated. Additional samples may be analysed if clinically indicated e.g., if AUTO4 cells become undetectable or at relapse. The assays have not yet been developed but will be based on assays used to measure human anti-human antibodies in patients treated with monoclonal antibodies or on assays to measure T cell responses in cell therapy trials. The development of the assays may utilise plasma, serum or PBMCs collected during the trial, and data will not be reported if a suitable assay cannot be developed.

9.4 EFFICACY EVALUATION

Response evaluations will be conducted as specified in the Schedule of Assessments and will include the following: CT and PET using [^{18}F]-fluorodeoxyglucose (FDG), physical examination, and other procedures as necessary. MRI may be used to evaluate sites of disease that cannot be adequately imaged using CT (in cases where MRI is desirable, the MRI must be obtained at baseline and at all subsequent response evaluations). For all other sites of disease, MRI studies do not replace the required neck, chest, abdomen, and pelvic CT scans. Brain MRI is only required if clinically indicated. These assessments should be performed throughout the study at each time point in the Schedule of Assessments using the same method of assessment used to assess disease at baseline. Patients receiving bridging therapy should have the baseline disease assessment after the bridging therapy is completed and prior to starting pre-conditioning. Response to treatment will be assessed by the Investigator at the site and the results will be recorded in the eCRF.

It is important that instances and evidence of disease progression be reported to the Sponsor as soon as possible. If progression is suspected from scan(s), but the patient is otherwise not showing clinical progression/deterioration, the disease progression must be confirmed not less than 28 days after initial finding (to rule out a pseudo-progression). The Medical Monitor will review the data to confirm that the criteria for disease progression have been met..

9.4.1 Radiographic Image Assessments (CT/MRI)

During the study, disease response will be assessed using CT scans with i.v. (and oral as necessary) contrast of the neck, chest, abdomen, pelvis and any other location where disease was present at Screening, and whole body [^{18}F]-FDG-PET scans. Patients who are intolerant of i.v. CT contrast agents may have CT scans performed without i.v. contrast.

A separate CT scan and PET scan are preferred, but if the only available modality is combined/dual PET/CT scanner, then the CT portion of a PET/CT may be used in lieu of a dedicated CT; CT scanning must be done according to certain imaging requirements that ensure that an optimised CT examination is done.

Magnetic resonance imaging may be used to evaluate sites of disease that cannot be adequately imaged using CT (in cases where MRI is desirable, the MRI must be obtained at baseline and at all subsequent response evaluations). For all other sites of disease, MRI studies do not replace the required neck, chest, abdomen, and pelvic CT scans. Brain MRI is only required if clinically indicated.

Radiological assessments will be performed as outlined in the Schedule of Assessments.

9.4.2 Positron Emission Tomography Scan

Positron emission tomography using [^{18}F]-FDG is important for the complete assessment of response and progression. Whole body [^{18}F]-FDG-PET scan (skull base to the proximal femur) is required at Screening and then performed as outlined in the Schedule of Assessments.

For patients who achieve a complete metabolic response (CMR), disease assessments after Month 6 can be based on CT scans alone, if clinically appropriate. If relapse occurs after CMR or disease progression is suspected (e.g. new or enlarging lesion(s) detected on CT scan), a PET scan is to be repeated to confirm relapse/progression.

Assessment of PET results is based on published criteria. Visual assessment is considered adequate for determining whether a PET scan is positive, and use of the standardised uptake value is not necessary. A positive scan is defined as focal or diffuse [^{18}F]-FDG uptake above background in a location incompatible with normal anatomy or physiology, without a specific standardised uptake value cut-off. Other causes of false-positive scans should be ruled out. Exceptions include mild and diffusely increased [^{18}F]-FDG uptake at the site of moderate- or large-sized masses with an intensity that is lower than or equal to the mediastinal blood pool, hepatic or splenic nodules 1.5 cm with [^{18}F]-FDG uptake lower than the surrounding liver/spleen uptake, and diffusely increased bone marrow uptake within weeks after treatment.

9.4.3 Efficacy Criteria

9.4.3.1 Assessment of Disease Response and Progressive Disease

Efficacy assessments for the purpose of the study result analyses will be performed by the Investigators according to the Lugano Classification (Cheson et al. 2014) (Appendix 1).

9.4.3.2 Definition of Measurable and Assessable Disease

Eligible patients must have PET-positive disease at baseline (FDG-avid disease corresponding with a 5-point scale score of 4 or 5). Patients who receive bridging therapy after study enrolment must have a PET/CT scan performed after completion of bridging therapy. Patients

who do not have PET-positive disease (5-point scale score of 4 or 5) after bridging treatment will be excluded from the primary efficacy analysis. Patients with PET-positive disease at baseline but without measurable disease per CT scan will be included in the primary efficacy analysis.

For radiological assessments based on CT scan (or MRI), measurable sites of disease are defined as lymph nodes, lymph node masses, or extranodal sites of lymphoma. Each measurable site of disease must be greater than 1.5 cm in the long axis regardless of short axis measurement, or greater than 1.0 cm in the short axis regardless of long axis measurement, and clearly measurable in two perpendicular dimensions. Measurement must be determined by imaging evaluation. All other sites of disease are considered assessable, but not measurable. Up to six measurable sites of disease, clearly measurable in two perpendicular dimensions, will be followed for each patient. Measurable sites of disease should be chosen such that they are representative of the patient’s disease (this includes splenic and extranodal disease). If there are lymph nodes or lymph node masses in the mediastinum or retroperitoneum larger than 1.5 cm in two perpendicular dimensions, at least one lymph node mass from each region should always be included. In addition, selection of measurable lesions should be from as disparate regions of the body as possible.

All other sites of disease will be considered assessable. Assessable disease includes objective evidence of disease that is identified by radiological imaging, physical examination, or other procedures as necessary, but is not measurable as defined above. Examples of assessable disease include bone lesions; mucosal lesions in the GI tract; effusions; pleural, peritoneal, or bowel wall thickening; disease limited to bone marrow; and groups of lymph nodes that are not measurable but are thought to represent lymphoma. In addition, if more than six sites of disease are measurable, these other sites of measurable disease may be included as assessable disease.

9.4.3.3 Efficacy Endpoints

The efficacy endpoints are ORR, DOR, DFS, PFS, and OS:

Overall response rate: CR or PR by the Criteria for Response Assessment of NHL (i.e. Lugano Classification). The proportion of patients achieving PR and CR at 1 and 3, and 6 months post-AUTO4 infusion will also be determined.

The **time to response** (PR+CR) and the **time to CR** will be calculated. These are defined as the time from the first treatment of AUTO4 to the response (either PR or CR as appropriate).

Duration of response: DOR is defined as the time from the first observed CR or PR to documented disease progression or death due to any cause, for patients who are considered as responders.

Progression-free survival: PFS is defined as the time from the first treatment of AUTO4 to documented disease progression/relapse or death due to any cause.

Overall survival: OS is defined as the time from the first treatment of AUTO4 to death due to any cause. Date of death will be recorded.

9.5 BLOOD VOLUME COLLECTIONS

In general blood, will be taken from a Hickman line or other central venous access for the administration of pre-conditioning treatment and for AUTO4 administration, and then for the first year after AUTO4 infusion so that venepuncture will not be needed. The total estimated volume of blood collected for safety, biomarkers and immunological assessments (with the

exception of the leukapheresis procedure) across the entire study will not normally exceed 900 mL (this is expected volume for females with serum pregnancy test). The maximum volume of blood collected for study related assessments on any one day will generally not exceed 80 mL. Additional samples may be collected as required to ensure the safety of the patient. Refer to the laboratory manual for the handling and storages of samples.

10 GUIDELINES FOR PREVENTION, MONITORING, AND MANAGEMENT OF ADVERSE EVENTS

The following guidance are recommendations and should be modified according to local practice and the patient's clinical need.

10.1 GENERAL SUPPORTIVE CARE GUIDELINES FOR PATIENTS RECEIVING CAR T CELL THERAPY

The guidelines detailed in Table 12 are suggested ways of supporting patients receiving CAR T cell therapy.

Table 12: General Supportive Care Guidelines for Patients Receiving CAR T Cell Therapy

Toxicity	Preventive and Supportive Care Interventions
General	Administer paracetamol for symptomatic management of fevers in patients;
	Appropriate personnel and appropriate resuscitation equipment should be available in or near the infusion room and a physician should be readily available during the infusion of study drug.
	Tocilizumab must be available at sites managing patients receiving CAR T cell products.
	Avoid corticosteroids.
	If thrombocytopenia, avoid non-steroidal anti-inflammatory drugs.
Infection Prophylaxis	Patients should receive prophylaxis with antimicrobials due to the use of fludarabine and potentially extended duration of lymphopenia and neutropenia.<br>Patients should receive Pneumocystis prophylaxis with trimethoprim-sulfamethoxazole or suitable alternative agents, and either acyclovir or valacyclovir for herpes virus prophylaxis from the start of conditioning chemotherapy until 6 months post AUTO4 infusion or longer as per institutional guidelines. Consider starting prophylaxis from the time of leukapheresis.<br>Additional anti-microbial (e.g. ciprofloxacin) and anti-fungal prophylaxis to be given as per institutional practice.<br>All patients should be monitored for CMV by PCR weekly during admission or as necessary.<br>All patients with fevers while neutropenic must have blood cultures drawn and receive broad-spectrum antibiotics as per institutional practice.
Respiratory	Monitor for oxygen saturation, a significant decrease in oxygen saturation at room air should be investigated and managed with supportive care including supplemental oxygen, anti-microbials and ventilator support as appropriate.<br>Patients with an oxygen requirement of >40% in the setting of CRS should receive treatment with Tocilizumab (see Section 10.4).
Cardiovascular	Stop or taper antihypertensive medications prior to cell infusion.
	Monitor vital signs at least every 4-8 hours on an inpatient unit during hospital stay.
	Monitor vital signs at least every 2 hours in patients with fevers and tachycardia.

Toxicity	Preventive and Supportive Care Interventions
	Initiate replacement i.v. fluids for patients with poor oral intake or high insensible losses to maintain net even fluid balance.<br>Administer i.v. fluid boluses for patients with systolic blood pressure less than 80% of their pre-infusion baseline and poor peripheral perfusion.<br>In patients receiving >2 i.v. fluid bolus for hypotension, consider transfer to the intensive care unit, an ECG and an ECHO should be performed to evaluate for cardiac toxicity.<br>For support and vasopressor use in the context of CRS See Section 10.4.
Hematologic	Initiate allopurinol and i.v. fluid prophylaxis for TLS prophylaxis at start of lymphodepletion in patients with high disease burden (See Section 10.7).
	Monitor complete blood count with differential at least daily.
	Maintain haemoglobin ≥80 g/L;
	Maintain platelets ≥20,000/µL;
	If absolute neutrophil count<500/µL at 30 days post CAR-T infusion (or earlier if clinically indicated), initiate granulocyte colony stimulating factor support. Continue until absolute neutrophil count increases to ≥1000 µL;
	Transfuse fresh frozen plasma with a goal of normalisation of partial thromboplastin time (PTT) in patients with a PTT >1.5-fold above the ULN; and transfuse cryoprecipitate to maintain fibrinogen of ≥1g/L. If patient is bleeding, a higher level of fibrinogen should be maintained.
Neurologic	Conduct focused neurologic examinations every 4-6 hours in patients experiencing neurologic toxicity (See Section 10.6)
	Perform brain magnetic resonance imaging (MRI) in any patient experiencing neurologic toxicity.
	Consider a neurology consultation for any patient experiencing neurologic toxicity.
	Standard antiepileptic medications for patients with active seizures.<br>Prophylactic antiepileptic medications are not required.

CMV = cytomegalovirus; ECG = electrocardiogram; ECHO = echocardiogram; i.v. = intravenous; MRI = magnetic resonance imaging; PTT = partial thromboplastin time; TLS = tumour lysis syndrome; ULN = upper limit of normal.

10.2 PRE- AND POST-INFUSION SUPPORTIVE THERAPY

AUTO4 is an autologous product and is unlikely to be immunogenic and induce an infusion or hypersensitivity reaction. The following medications should be given 30 minutes before the study drug infusion: paracetamol/acetaminophen orally and an intravenous antihistamine (chlorpheniramine or equivalent). Additional pre-infusion medications listed in Table 13 may also be administered if necessary.

Post-infusion medication listed in Table 13 may be considered following AUTO4 infusion if necessary. Post-infusion medication may be continued for up to 48 hours after the infusion. Use of additional supportive care measures may be instituted as clinically necessary at the discretion of the Investigator.

Table 13: Pre- and Post-infusion Medications

Medication	Dose	Administration	Pre-infusion	Post-infusion
Antihistamine	Chlorpheniramine (10 mg, 6 hourly). Refer to local prescribing information	I.V. – administer at least 30 minutes prior to study drug	Recommended	Optional as clinically indicated
Antipyretic	Paracetamol /acetaminophen (1000 mg; 6 hourly). Refer to local prescribing information	Oral - administer at least 30 minutes prior to study drug	Recomended-	Optional as clinically indicated
Antiemetic	Ondansetron 8 mg or equivalent Refer to local prescribing information	I.V. - start infusion 30 minutes prior to study drug	Optional	Optional as clinically indicated
	Ondansetron (8 mg, twice daily) or equivalent. Refer to local prescribing information	Oral - as clinically indicated	-	Optional as clinically indicated

I.V. = intravenous;
Note: Steroids should in general be avoided but may be used in case of severe reactions not controlled by other measures.

10.3 Management of Infusion Related Reactions

Patients who experience infusion-related reactions to AUTO4 that manifest as wheezing, flushing, hypoxemia, fever, chills, rigors, bronchospasm, headache, rash, pruritus, arthralgia, hypo- or hypertension or other symptoms, should have the symptoms managed according to the recommendations provided in Table 14 or as per institutional practice. All NCI CTCAE Grade 3 or 4 infusion-related reactions should be reported within 24 hours to the Sponsor as an AESI. Such events may also meet the criteria as an SAE and should be reported accordingly.

Table 14: Guidelines for the Management of Infusion-related Reactions

NCI CTCAE Grade	Treatment/Intervention<br>Refer to local prescribing information for all described medications.
**Grade 1**<br>Mild reaction; infusion interruption not indicated; intervention not indicated	**No intervention indicated**<br>Monitor patient as medically indicated until recovery from symptoms.
**Grade 2**<br>Moderate reaction;<br>Therapy or infusion interruption indicated but responds promptly to symptomatic treatment e.g., antihistamines, NSAIDS (if appropriate), narcotics, i.v. fluids; prophylactic medications indicated for <=24 hrs	**Interrupt infusion**<br>Start i.v. fluids; give chlorpheniramine (10 mg 6 hourly) (or equivalent) i.v. and/or paracetamol; consider bronchodilator therapy; may also consider corticosteroids if necessary; monitor patient closely until recovery from symptoms.<br>Restart infusion if AUTO4 dose has not been fully administered.<br>**Symptoms recur:** Stop and discontinue further infusion.<br>The amount of AUTO4 infused must be recorded on the eCRF.
**Grade 3 or 4**<br>Severe reaction;<br>**Grade 3**<br>Prolonged (e.g., not rapidly responsive to symptomatic medication and/or brief interruption of infusion); recurrence of symptoms following initial improvement; hospitalization indicated for clinical sequelae<br>**Grade 4:** life-threatening; pressor or ventilator support indicated	**Stop infusion**<br>Start i.v. saline infusion; recommend bronchodilators and chlorpheniramine (10 mg 6 hourly) i.v. ± adrenaline 1 µg/kg for patients <50 kg and 50 µg for patients over 50 kg, injected slowly for i.v. administration or administer intramuscularly. If unresponsive, give with methylprednisolone 1 mg/kg (max 80 mg) i.v. (or equivalent), as needed, and other drugs as appropriate.<br>Patients should be monitored until the Investigator is comfortable that the symptoms will not recur. Investigators should follow their institutional guidelines for the treatment of anaphylaxis. In the case of late-occurring hypersensitivity symptoms (e.g. appearance of a localised or generalised pruritus within 1 week after treatment), symptomatic treatment may be given (e.g. oral antihistamine or corticosteroids), as appropriate.

CTCAE = Common Terminology Criteria for Adverse Events (Grade 5.0); eCRF = Electronic Case Report Form; i.v. = intravenous; NCI = National Cancer Institute; NSAID = non-steroidal anti-inflammatory drug.
Refer to local prescribing information for all described medications.

10.4 Grading and Management of Cytokine Release Syndrome (CRS)

Cytokine Release Syndrome (CRS) is a recognised toxicity with CAR T cell therapies and for some CAR-T therapies, can be severe (Grade 3 or 4) in 40% of patients, with Acute Lymphoblastic Leukaemia (ALL) and in 20% of patients, with Diffuse Large B Cell Lymphoma (DLBCL) (Kymriah (tisagenlecleucel) EU SmPC 2018, Kymriah US Prescribing Information 2018). Clinical symptoms indicative of CRS includes culture negative fever, but may also include, myalgia, nausea/vomiting, tachycardia, hypoxia, hypotension, headache, confusion, tremor, and delirium. Potentially life-threatening complications of CRS may include cardiac dysfunction, acute respiratory distress syndrome, renal and/or hepatic failure, and disseminated intravascular coagulation (DIC) (Brudno and Kochenderfer 2016). Symptoms usually present within the first 1 to 2 weeks following infusion (Neelapu et al. 2018).

Some studies have suggested several possible risk factors for severe CRS, such as higher peak expansion level of CAR-T, tumour burden, baseline high lactate dehydrogenase (LDH) level, early onset CRS (within 3 days of infusion) and elevation of some cytokine levels after infusion (Hay et al. 2017, Siddiqi et al. 2017, Santomasso et al. 2018).

Macrophage activation syndrome (MAS) and haemophagocytic lymphohistiocytosis (HLH) may occur in some for whom CAR-mediated inflammatory responses continue to evolve. The clinical syndrome of MAS is characterised by high grade non-remitting fever, cytopenias affecting at least two of three lineages, and hepatosplenomegaly (See Section 1.1). It is associated with biochemical abnormalities, such as high circulating levels of serum ferritin, soluble interleukin-2 receptor (sCD25), and triglycerides, together with a decrease of circulating NK activity. Other findings include variable levels of transaminases up to signs of acute liver failure and coagulopathy with findings consistent with DIC.

Following the first AUTO4 infusion patients should be closely monitored for early signs and symptoms indicative of CRS with clinical review and blood tests including C-reactive protein, serum ferritin levels and clotting. Serum cytokines should be obtained periodically as indicated in the schedule of assessments. Clinical personnel should be trained in the diagnosis and management of CRS.

Grading for CRS is provided in Table 15 with recommendations regarding treatment provided in Table 16.

Table 15: Severity Grading of Cytokine Release Syndrome (ASTCT/ASBMT CRS Consensus Grading and CTCAE Version 5.0) (Lee et al. 2019)

**CRS Parameter**	**Grade 1**	**Grade 2**	**Grade 3**	**Grade 4**
Fever†	Temperature ≥38°C	Temperature ≥38°C	Temperature ≥38°C	Temperature ≥38°C
With either:				
Hypotension	None	Not requiring vasopressors	Requiring one vasopressor with or without vasopressin	Requiring multiple vasopressors (excluding vasopressin)
And/or‡				
Hypoxia	None	Requiring low-flow<br>nasal cannula^ or blow-by	Requiring high-flow nasal cannula^, facemask, non-rebreather mask, or Venturi mask	Requiring positive pressure (eg: CPAP, BiPAP, intubation and mechanical ventilation)

Organ toxicities associated with CRS may be graded according to CTCAE v5.0 but they do not influence CRS grading.
ASBMT = American Society for Blood and Marrow Transplantation; ASTCT = American Society for Transplantation and Cellular Therapy; BiPAP = bilevel positive airway pressure CPAP = continuous positive airway pressure; CRS = cytokine release syndrome; CTCAE = Common Terminology Criteria for Adverse Events;

† Fever is defined as temperature ≥38°C not attributable to any other cause. In patients who have CRS then receive antipyretics or anti-cytokine therapy such as tocilizumab or steroids, fever is no longer required to grade subsequent CRS severity. In this case, CRS grading is driven by hypotension and/or hypoxia.

‡ CRS grade is determined by the more severe event: hypotension or hypoxia not attributable to any other cause. For example, a patient with temperature of 39.5°C, hypotension requiring one vasopressor and hypoxia requiring low-flow nasal cannula is classified as having Grade 3 CRS.

^ Low-flow nasal cannula is defined as oxygen delivered at ≤ 6 L/minute. Low flow also includes blow-by oxygen delivery, sometimes used in paediatrics. High-flow nasal cannula is defined as oxygen delivered at > 6 L/minute.

Table 16: Management of Cytokine Release Syndrome

CRS Grade (CTCAE Version 5.0)	Treatment
**Grade 1**<br>Symptoms are not life-threatening and require symptomatic treatment only, e.g. fever, nausea, fatigue, headache, myalgia, and malaise or organ toxicity.	Supportive care per institutional standards including analgesics and antipyretics, assess and treat for neutropenic infections.<br>Consider anti-IL-6 therapy for persisting (> 3 days) and refractory fever
**Grade 2**<br>Symptoms require and respond to moderate intervention	Supportive care including fluid substitution is recommended.<br>Low-flow-oxygen (<40% fraction of inspired oxygen).<br>Consider anti-IL-6 therapy early if persistent fever of ≥39°C despite antipyretics for 10 hours (Lee et al. 2014, Neelapu et al. 2018).
**Grade 3**<br>Symptoms require and respond to aggressive intervention	Intensive care should be considered.<br>Oxygen (flow ≥40% fraction of inspired oxygen)<br>Vasopressors as needed (See Table 17)<br>Treat with Immunotherapy anti-IL-6 therapy (tocilizumab or siltuximab, See Table 17)<br>Add siltuximab as necessary if not previously administered.<br>Add steroids if unresponsive within 24 hours.<br>CRS associated with MAS or haemophagocytosis may also be treated with anakinra, an IL-1 receptor antagonist (Shah et al. 2017)<br>Also, consider anti-TNF antibodies as clinically appropriate.
**Grade 4**<br>Life-threatening symptoms,	Intensive care.<br>Treat with Anti-IL-6 therapy with tocilizumab, ± siltuximab, (See Table 17)<br>Treat with Corticosteroids (See Table 17).<br>CRS associated with MAS or haemophagocytosis may also be treated with anakinra, an IL-1 receptor antagonist (Shah et al. 2017).<br>Consider alternative agents such as anti-TNF, and other agents as appropriate
**Grade 5**<br>Death	

CRS = cytokine release syndrome; CTCAE = Common Terminology Criteria for Adverse Events; IL = interleukin; MAS = macrophage activation syndrome; NCI = National Cancer Institute; TNF = tumour necrosis factor.

Details of supportive medication and steroid doses are presented in Table 17.

An overview of the CRS management is presented in Figure 4.

Figure 4: Cytokine Release Syndrome Management Overview

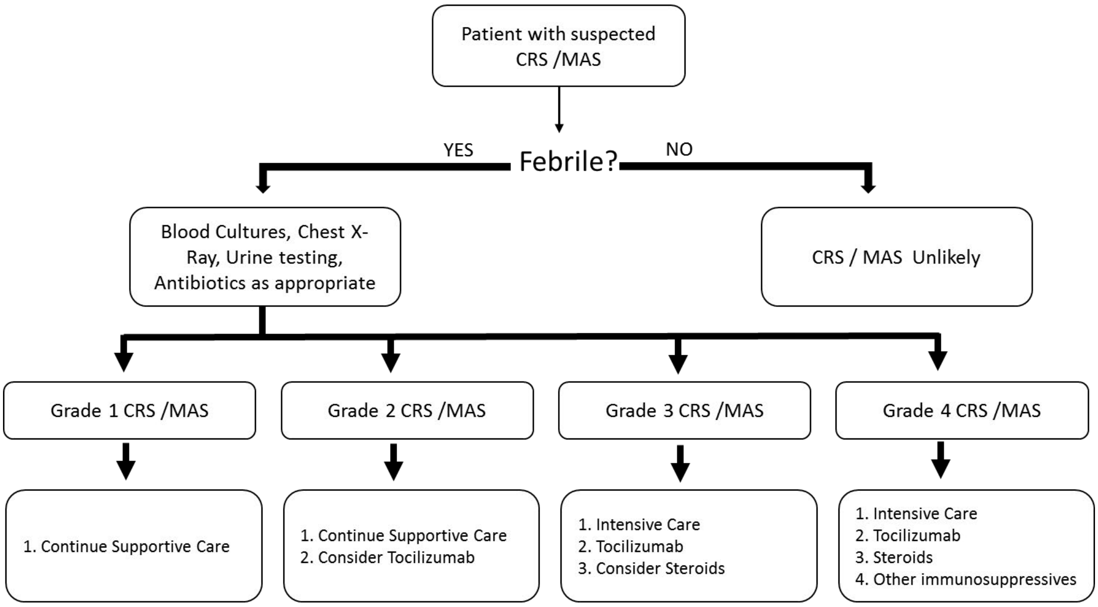

Table 17: Pharmacologic Management of CRS and ICANS

**Drug**	**Indication**	**Dose**
Vasopressor	Hypotension not responsive to fluid resuscitation alone<br>Vasopressor use according to local institutional practice. Noradrenaline/norepinephrine is the preferred first-line vasopressor (Brudno and Kochenderfer 2016). Recommended doses provided (Lee et al. 2014). Vasopressor should be used for at least 3 hours.<br><br>Note: Vasopressin not available in all countries.	Noradrenaline/norepinephrine monotherapy ≥0.2 mcg/kg/minute<br>Dopamine monotherapy ≥10 µg/kg/minute<br>Adrenaline monotherapy ≥0.1 µg/kg/minute<br><br>If on vasopressin:<br>Vasopressin + Noradrenaline/norepinephrine equivalent of ≥10 µg/min*<br>If on a combination vasopressors (not vasopressin):<br>Noradrenaline/Norepinephrine equivalent of ≥20 µg/min*
Anti-IL-6 therapy	Anti-IL-6 Therapy may be given according to local institutional practice.<br>Use of Anti-IL-6 Treatments is not currently believed to impair the efficacy of CAR-T cell therapy (Davila et al. 2014, Lee et al. 2015).<br>Brundno and Kochenderfer (Brudno and Kochenderfer 2016) recommended the use of Anti-IL6 therapy (specifically Tocilizumab) in the event of the following associated with CRS (CRS Grade ≥2 with associated conditions):<br>• Left ventricular ejection fraction ,40% by echocardiogram;<br>• Creatinine >2.5-fold higher than the most recent level prior to CAR T cell infusion;<br>• Norepinephrine requirement at a dose >2 mg/min for 48 h since the first administration of norepinephrine, even if administration is not continuous;<br>• SBP of 90 mm Hg that cannot be maintained with norepinephrine;<br>• Oxygen requirement of FiO2 50% or more for more than 2 hours continuously;<br>• Dyspnoea that is severe enough to potentially require mechanical ventilation;<br>• Activated PTT >2 x the upper limit of normal;<br>• Clinically-significant bleeding;<br>• Creatine kinase >5 x the upper limit of normal for longer than 2 days	Tocilizumab<br>Adults: The recommended dosing of Tocilizumab for the treatment of CRS given as a 60-minute intravenous infusion is 8 mg/kg in patients weighing greater than or equal to 30 kg or 12 mg/kg in patients weighing less than 30 kg (Maude et al. 2015).<br>Tocilizumab can be given alone or in combination with corticosteroids. If no clinical improvement in the signs and symptoms of CRS occurs after the first dose, up to 3 additional doses of Tocilizumab may be administered. The interval between consecutive doses should be at least 8 hours. Doses exceeding 800 mg per infusion are not recommended in CRS patients

Drug	Indication	Dose
	Use of tocilizumab may also be considered for persistent fever of ≥39°C despite antipyretics for 10 hours, persistent/ recurrent hypotension after initial fluid bolus, and initiation of oxygen supplementation (Lee et al. 2014, Neelapu et al. 2018).<br>**Tocilizumab**<br>Tocilizumab is approved in the US and Europe for the management of CRS patients (Actemra Prescribing Information 2018, RoActemra SmPC 2018).<br>Refer to local prescribing information.<br>Note: organ dysfunction secondary to CRS and cytopaenias due to disease/chemotherapy will not constitute a contraindication to tocilizumab.<br><br>**Siltuximab**<br>Siltuximab is an alternative to tocilizumab (Grupp et al. 2013, Calabrese and Rose-John 2014, Maude et al. 2015, Teachey et al. 2018) and may be used according to local institutional guidance. Siltuximab has been approved to treat Castleman's disease (EU SmPC and US PI).<br><br>Siltuximab may be added if there is no response to tocilizumab ± corticosteroids (Teachey et al. 2018, Neelapu et al. 2018).	(Actemra Prescribing Information 2018, RoActemra SmPC 2018).<br>Refer to local prescribing information and institutional guidance.<br><br>Siltuximab<br>11mg/kg over 1 hour as an i.v. infusion as a single dose (Neelapu et al. 2018, Siltuximab US Prescribing Information 2019).

Drug	Indication	Dose
Corticosteroids	Corticosteroids should generally be avoided in patients who have received CAR-T cell therapy, due to concerns of the effect on the T cells (Neelapu et al. 2018), though, some studies have shown a clinical response to CAR-T therapy after high doses of methylprednisolone (Maude et al. 2015, Mueller et al. 2018).<br>Corticosteroids are indicated in Grade 4 CRS and Grade 3 CRS refractory to Anti-IL-6 therapy. Corticosteroids are also indicated in patients with neurotoxicity (ICANS) Grade 2 and above (See Section 10.6). Patients with ICANS in the absence of CRS should receive corticosteroids prior to Anti-IL6 therapy.<br>Although dosing and choice of corticosteroid should be tailored to the individual patient, and according to local institutional guidance (if specified). Commonly used initial doses include methylprednisolone.<br>For patients with suspected ICANS, dexamethasone may be more suitable, due more efficient penetration of the blood brain barrier (Mitchell et al. 2005) (see section 10.6).<br>Patients on IV steroids may be switched to an equivalent dose of oral corticosteroids (e.g. prednisone) at start of tapering or earlier, once sustained clinical improvement is observed.<br>Prophylactic antibiotics or other antimicrobials as clinically appropriate.<br>Rigorous control of blood pressure and electrolytes (particularly calcium and magnesium).	Methylprednisolone<br>2 mg/kg i.v. every 12 hours weaned over 5 days.<br><br>Dexamethasone<br>10mg i.v. every 6 hours (if refractory can increase to 20mg every 6 hours) (Neelapu et al. 2018)
Anti-TNF therapy	**Infliximab** (anti-TNFα antibody),<br>**Etanercept** (soluble TNFα receptor)<br>These agents have also demonstrated efficacy in the setting of CRS, macrophage activation syndrome and other syndromes (Prahalad et al. 2001, Gabay et al. 2010, Flammiger et al. 2012, Ruella et al. 2017)	
Anti-IL-1 Therapy	**Anakinra**<br>Uncontrolled MAS or haemophagocytosis may also be treated with anakinra, an IL-1 receptor antagonist (Brudno and Kochenderfer 2016, Shah et al. 2017, Liu and Zhao 2018).	100 mg by subcutaneous injection, to be repeated daily as clinically indicated.

* VASST (Russell et al. 2008) vasopressor equivalent equation: norepinephrine equivalent dose = [norepinephrine (µg/min)] + [dopamine (µg/kg/min) ÷ 2] + [epinephrine (µg/min)] + [phenylephrine (µg/min) ÷10].
CAR = chimeric antigen receptor; CRS = cytokine release syndrome; CTCAE = Common Terminology Criteria for Adverse Events; ICANS = Immune Effector Cell-associated Neurotoxicity Syndrome; IL = interleukin; MAS = macrophage activation syndrome; NCI = National Cancer Institute; PI = product insert; PPT = partial thromboplastin time; SBP = systolic blood pressure; SmPC = summary of product characterstics; TNF = tumour necrosis factor; US = United States;
VASST = Vasopressin and Septic Shock Trial.

10.5 Management Macrophage Activation Syndrome (MAS)

Macrophage activation syndrome (MAS) and HLH encompasses a group of severe immunological disorders characterised by hyperactivation of macrophages and lymphocytes, proinflammatory cytokine production, lymphohistiocytic tissue infiltration, and immune-mediated multiorgan failure.

Haemophagocytic lymphohistiocytosis and/or MAS may occur in some for whom CAR-mediated inflammatory responses continue to evolve. The clinical syndrome of MAS is characterised by high grade non-remitting fever, cytopenias affecting at least two of three lineages, and hepatosplenomegaly.

A patient might have HLH/MAS if they have a peak serum ferritin level of >10,000 ng/mL during the CRS phase of CAR-T-cell therapy and subsequently developed any two of the following (Neelapu et al. 2018):

- CTCAE Grade ≥3 increase in serum bilirubin, aspartate aminotransferase, or alanine aminotransferase levels
- CTCAE Grade ≥3 oliguria or increase in serum creatinine levels
- CTCAE Grade ≥3 pulmonary oedema
- Presence of haemophagocytosis in bone marrow or organs based on histopathological assessment of cell morphology and/or CD68 immunohistochemistry

HLH/MAS may be refractory to Anti-IL6 therapy and can be associated with a high mortality if not treated promptly. Patients with suspected HL/MAS should be treated with Anti-IL6 therapy and corticosteroids (Neelapu et al. 2018) (See Table 17). If the patient has no improvement, clinically or serologically, within 48 hours, consider etoposide 75-100mg/m^2 as appropriate (Jordan et al. 2011, Schram and Berliner 2015, Tamamyan et al. 2016). Intrathecal cytarabine can be considered in patients with HLH associated neurotoxicity (Neelapu et al. 2018).

Uncontrolled HLH/MAS may also be treated with anakinra, an IL-1 receptor antagonist (See Table 17) (Brudno and Kochenderfer 2016, Shah et al. 2017, Liu and Zhao 2018).

10.6 Grading and Management of Neurotoxicity (ICANS)

Neurotoxicity has been seen in patients with leukaemia and lymphoma after treatment with CAR T cell therapy (Neelapu et al. 2018) and is now referred to as ICANS. The cause of neurotoxicity is not well understood, although it is generally reported to be fully reversible (Kymriah (tisagenlecleucel) EU SmPC 2018, Kymriah US Prescribing Information 2018). Enrichment of pro-inflammatory cytokines in the central nervous system, endothelial activation, and macrophage activation syndrome have all been proposed as potential mechanisms (Karschnia et al. 2019). Transient neurological complications have also been reported with CD19 bispecific T cell engagers, suggesting that the target may have some relevance (Goebeler and Bargou 2016).

Although symptoms can vary, the early manifestations of ICANS are often tremor, dysgraphia, mild difficulty with expressive speech especially with naming objects, impaired attention, apraxia, and mild lethargy. Other symptoms can include confusion, depressed level of consciousness/encephalopathy, hallucinations, dysphasia, ataxia, apraxia, cranial nerve palsies, and seizures. Headache is a non-specific symptom, frequently occurring during fever or after

chemotherapy, thus, headache alone is not a useful marker of ICANS. Expressive aphasia, on the other hand, appears to be a very specific symptom of ICANS (Santomasso et al. 2018).

In addition to more common neurotoxicity symptoms such as encephalopathy, aphasia, delirium, tremor, and seizures, rare cases of rapid-onset and lethal diffuse cerebral oedema have occurred with some CAR-T cell therapies (Gilbert 2017, Gust et al. 2017, Locke et al. 2017).

There appears to be no correlation between ICANS and CRS/MAS and both can occur together or separately. Although ICANS occurs more frequently in the presence of severe CRS. There is also some suggestion that patients with a high disease burden prior to treatment, higher peak CAR-T expansion and early and higher elevations of serum cytokines may have a higher risk of neurotoxicity. Of note, patients can develop ICANS even after treatment with an nti-IL6 therapy, after the resolution of CRS.

Patients will be monitored closely after the AUTO4 infusion and closely monitored for neurological signs and symptoms of neurotoxicity. If neurotoxicity is observed, a neurology opinion should be sought, MRI imaging performed, and the patient will receive supportive care (e.g. anti-convulsant therapy), as appropriate (See Table 20).

In general, neurotoxicity is transient and resolves spontaneously with no long-term sequelae so that supportive care alone is sufficient in most patients. Steroids may be given for Grade 3 or 4 neurotoxicity (See Table 20) or in the case of cerebral oedema or generalised seizures but otherwise treatment with steroids should be avoided as this may be deleterious to the persistence of AUTO4.

Neurotoxicity may also be caused by fludarabine but usually at higher doses than those being administered in this protocol (Helton et al. 2013). Symptoms of fludarabine neurotoxicity including objective weakness, agitation, confusion, seizures, visual disturbances, optic neuritis, optic neuropathy, blindness, and coma have been reported in CLL patients treated with multiple cycles of fludarabine.

Neurotoxicity may also be associated with prior methotrexate treatment (either high dose or intrathecal) (Inaba et al. 2008, Bhojwani et al. 2014). Although neurological toxicities associated with methotrexate are usually acute, radiological abnormalities may persist (Bhojwani et al 2014).

Please see Table 18 for grading of neurotoxicity as per the ASTCT/ASBMT guidelines for ICANS (Lee et al. 2019).

Table 18: Assessment & Grading of ICANS

	Grade 1	Grade 2	Grade 3	Grade 4
**ICE Score for Adults^ (See Table 19)**	7-9	3-6	0-2	0 (patient is unarousable and unable to perform ICE)
**Depressed level of consciousness***	Awakens spontaneously	Awakens to voice	Awakens only to tactile stimulus	Patient is unarousable or requires vigorous or repetitive tactile stimuli to arouse; stupor or coma
**Seizure (any age)**	N/A	N/A	Any clinical seizure focal or generalized that resolves rapidly; or Non-convulsive seizures on EEG that resolve with intervention	Life-threatening prolonged seizure (>5 min); or Repetitive clinical or electrical seizures without return to baseline in between.
**Motor weakness (any age)§**	N/A	N/A	N/A	Deep focal motor weakness such as hemiparesis or paraparesis
**Raised ICP / Cerebral Oedema (any age)**			Focal/local oedema on neuroimaging#	Decerebrate or decorticate posturing; or Cranial nerve VI palsy; or Papilledema; or Cushing's triad; or Signs of diffuse cerebral oedema on neuroimaging

Adapted from (Lee et al. 2019), ASBMT ICANS Consensus
ICANS grade is determined by the most severe event (ICE or CAPD score, level of consciousness, seizure, motor findings, raised ICP/cerebral oedema) not attributable to any other cause.
ASBMT = American Society for Blood and Marrow Transplantation; CAPD = Cornell Assessment of Paediatric Delirium; CTCAE = Common Terminology Criteria for Adverse Events; EEG = electroencephalogram; ICANS = Immune Effector Cell-associated Neurotoxicity Syndrome; ICE = Immune effector Cell-associated Encephalopathy; ICP = Intracranial pressure.
^ A patient with an ICE score of 0 may be classified as having Grade 3 ICANS if the patient is awake with global aphasia. But a patient with an ICE score of 0 may be classified as having Grade 4 ICANS if the patient is unarousable.
* Depressed level of consciousness should be attributable to no other cause (e.g. no sedating medication)
§ Tremors and myoclonus associated with immune effector cell therapies may be graded according to CTCAE v5.0 but they do not influence ICANS grading.
# Intracranial haemorrhage with or without associated oedema is not considered a neurotoxicity feature and is excluded from ICANS grading. It may be graded according to CTCAE v5.0.

Table 19: Immune Effector Cell-associated Encephalopathy (ICE) Scale

Orientation: orientation to year, month, city, hospital	4 points
Following commands: ability to follow simple commands (e.g. “Show me 2 fingers” or “Close your eyes and stick out your tongue”)	1 point
Naming: ability to name 3 objects (e.g. point to clock, pen, button)	3 points
Writing: ability to write a standard sentence (e.g. “Our national bird is the bald eagle”)	1 point
Attention: ability to count backwards from 100 by 10	1 point
**Scoring**<br>10, no impairment;<br>7-9, grade 1 ICANS;<br>3-6, grade 2 ICANS;<br>0-2, grade 3 ICANS;<br>0 due to patient unarousable and unable to perform ICE assessment	

ICANS = Immune Effector Cell-associated Neurotoxicity Syndrome; ICE = Immune effector Cell-associated Encephalopathy.

Patient may be managed as per the suggested guidelines below (Table 20) but may also be managed as per institutional management guidelines.

Table 20: Management of ICANS

Grade	Recommended Management
**Grade 1**	Vigilant supportive care; aspiration precautions; i.v. hydration<br>Withhold oral intake of food, medicines, and fluids, and assess swallowing<br>Convert all oral medications and/or nutrition to i.v. if swallowing is impaired<br>Avoid medications that cause central nervous system depression<br>Low doses of lorazepam (0.25 to 0.5 mg i.v. every 8) or haloperidol (0.5 mg i.v. every 6 hours) can be used, with careful monitoring, for agitated patients<br>Neurology consultation<br>Fundoscopic exam to assess for papilloedema<br>MRI of the brain with and without contrast; diagnostic lumbar puncture with measurement of opening pressure; MRI spine if the patient has focal peripheral neurological deficits; CT scan of the brain can be performed if MRI of the brain is not feasible<br>Daily 30 min EEG until toxicity symptoms resolve; if no seizures are detected on EEG.<br>Consider levetiracetam 750 mg every 12 hours (oral or i.v.) for a month for seizure prophylaxis.<br>If EEG shows non-convulsive status epilepticus, treat as per algorithm (Table 21)<br>Consider Anti-IL-6 therapy if neurotoxicity is associated with concurrent CRS (See Section 10.4).<br>**Worsening**: treat as ≥Grade 2
**Grade 2**	Supportive care and neurological work-up as indicated for Grade 1.<br>Anti-IL-6 therapy if associated with concurrent CRS (See Section 10.4).<br>Dexamethasone 10 mg i.v. every 6 hours or methylprednisolone 1 mg/kg i.v. every 12 hours if refractory to anti-IL-6 therapy, or for neurotoxicity without concurrent CRS.<br>Consider transferring patient to ICU if neurotoxicity is associated with Grade ≥2 CRS.<br>**Worsening:** treat as Grade 3 to 4
**Grade 3 neurologic toxicities,** (with the exception of headaches, that last continuously for 24 hours or longer)	Supportive care and neurological work-up as indicated for Grade 1.<br>ICU transfer is recommended<br>Anti-IL-6 therapy if associated with concurrent CRS, as described for Grade 2 neurotoxicity and if not administered previously.<br>Dexamethasone 10 mg i.v. every 6 hours or methylprednisolone 1 mg/kg IV every 12 hours if refractory to anti-IL-6 therapy, or for neurotoxicity without concurrent CRS; continue corticosteroids until improvement to Grade 1 neurotoxicity and then taper (See Table 17).<br>Stage 1 or 2 papilloedema with CSF opening pressure <20 mmHg should be treated as per algorithm (Table 22).<br>Consider repeat neuroimaging (CT or MRI) every 2 to 3 days if patient has persistent Grade ≥3 neurotoxicity.

Grade	Recommended Management
**Grade 4 neurologic toxicity of any duration**<br><br>**Any generalised seizures**	Supportive care and neurological work-up as outlined for Grade 1 neurotoxicity.<br>ICU monitoring; consider mechanical ventilation for airway protection.<br>Anti-IL-6 therapy and repeat neuroimaging as described for Grade 3 neurotoxicity.<br>High-dose corticosteroids continued until improvement to Grade 1 neurotoxicity and then taper; for example, methylprednisolone i.v. 1 g/day for 3 days, followed by rapid taper at 250 mg every 12 hours for 2 days, 125 mg every 12 hours for 2 days, and 60 mg every 12 hours for 2 days (See Table 17).<br>For convulsive status epilepticus, treat as per algorithm (Table 21).<br>Stage ≥3 papilloedema, with a CSF opening pressure ≥20 mmHg or cerebral oedema, should be treated as per algorithm (Table 22).<br>**Worsening:** May consider use of lymphodepleting drugs such as cyclophosphamide (Garfall et al. 2015) or other drugs (Klinger et al. 2016) if unresponsive to standard immunosuppressive therapies.

Adapted from (Neelapu et al. 2018). Grading of neurotoxicity as per the ASTCT/ASBMT guidelines for Immune effector Cell-Associated Neurotoxicity Syndrome (ICANS) (Lee et al. 2019).
AE = adverse event; ASBMT = American Society for Blood and Marrow Transplantation; ASTCT = American Society for Transplantation and Cellular Therapy; CRS = cytokine release syndrome; CSF = cerebrospinal fluid; CT = computerised tomography; EEG = electroencephalogram; ICU = intensive-care unit; i.v. = intravenous; MRI = magnetic resonance imaging.

Table 21: Recommendations for the Management of Status Epilepticus After CAR T Cell Therapy

Event	Management
General	Patients should be managed according to local institutional practice and with the consultation, or management, of a neurologist
Non-convulsive status epilepticus	Assess airway, breathing, and circulation; check blood glucose.<br>Lorazepam 0.5 mg i.v., with additional 0.5 mg i.v. every 5 min, as needed, up to a total of 2 mg to control electrographical seizures.<br>Levetiracetam 500 mg IV bolus, as well as maintenance doses.<br>If seizures persist, transfer to ICU and treat with phenobarbital loading dose of 60 mg i.v.<br>Maintenance doses after resolution of non-convulsive status epilepticus are as follows: lorazepam 0.5 mg i.v. every 8 hours for three doses; levetiracetam 1000 mg i.v. every 12 hours; phenobarbital 30 mg i.v. every 12 hours.
Convulsive status epilepticus	Assess airway, breathing, and circulation; check blood glucose.<br>Transfer to ICU.<br>Lorazepam 2 mg i.v., with additional 2 mg i.v. to a total of 4 mg to control seizures.<br>Levetiracetam 500 mg i.v. bolus, as well as maintenance doses.<br>If seizures persist, add phenobarbital treatment at a loading dose of 15 mg/kg i.v.<br>Maintenance doses after resolution of convulsive status epilepticus are: lorazepam 0.5 mg i.v. every 8 hours for three doses; levetiracetam 1000 mg i.v. every 12 hours; phenobarbital 1–3 mg/kg i.v. every 12 hours.<br>Continuous electroencephalogram monitoring should be performed, if seizures are refractory to treatment.

Adapted from (Neelapu et al. 2018)
ICU = intensive-care unit; i.v. = intravenous.
All indicated doses of medication are for adult patients. Please check local prescribing information

Table 22: Recommendation for Management of Raised Intracranial Pressure (ICP) After CAR T Cell Therapy

Condition	Management
Stage 1 or 2 papilloedema* with CSF opening pressure of <20 mmHg without cerebral oedema	Acetazolamide 1000 mg i.v., followed by 250 to 1000 mg i.v. every 12 hours (adjust dose based on renal function and acid–base balance, monitored 1 to 2 times daily).
Stage 3, 4, or 5 papilloedema*, with any sign of cerebral oedema on imaging studies, or a CSF opening pressure of ≥20 mmHg	Use high-dose corticosteroids with methylprednisolone i.v. 1 g/day. See Table 17<br>Elevate head end of the patient's bed to an angle of 30 degrees.<br>Hyperventilation to achieve target partial pressure of arterial carbon dioxide ($PaCO_2$) of 28 to 30 mmHg but maintained for no longer than 24 hours.<br>Hyperosmolar therapy with either mannitol (20 g/dL solution) or hypertonic saline (3% or 23.4%, as detailed below)<br>• Mannitol: initial dose 0.5 to 1 g/kg; maintenance at 0.25 to 1 g/kg every 6 hours while monitoring metabolic profile and serum osmolality every 6 hours and withhold mannitol if serum osmolality is ≥320 mOsm/kg, or the osmolality gap is ≥40.<br>• Hypertonic saline: initial 250 mL of 3% hypertonic saline; maintenance at 50 to 75 mL/h while monitoring electrolytes every 4 hours, and withhold infusion if serum Na levels reach ≥155 mEq/l.<br>• For patients with imminent herniation: initial 30 mL of 23.4% hypertonic saline; repeat after 15 minutes, if needed.<br>If patient has Ommaya reservoir, drain CSF to target opening pressure of <20 mmHg.<br>Consider neurosurgery consultation and IV anaesthetics for burst-suppression pattern on electroencephalography.<br>Metabolic profiling every 6 hours and daily CT scan of head, with adjustments in usage of the medications to prevent rebound cerebral oedema, renal failure, electrolyte abnormalities, hypovolemia, and hypotension.

Adapted from (Neelapu et al. 2018)
CSF = cerebrospinal fluid; CT = computerised tomography; i.v. = intravenous.
*Papilloedema grading should be performed according to the modified Frisén scale (Frisen 1982)

10.7 Management of Tumour Lysis Syndrome

Treatment with CAR T cells can lead to rapid killing of malignant cells, which can occasionally result in Tumour Lysis Syndrome (TLS) associated with a release of intracellular ions and metabolic by-products into the systemic circulation in patients with a high leukaemic/tumour burden. Clinically, TLS can be characterised by rapid development of hyperuricemia, hyperkalaemia, hyperphosphatemia, hypocalcaemia, and potentially acute renal failure.

Patients with a high leukaemic/ tumour burden (such as >25% bone marrow blasts in leukaemia) should be given allopurinol and i.v. fluids from the start of lymphodepletion to prevent TLS. Patients should be monitored vigilantly for early signs and symptoms of TLS with daily clinical review and blood biochemistry.

Should TLS occur, supportive care will be given as per standard institutional practice Management of TLS will usually include i.v. fluids, aggressive correction of abnormal laboratory test results such as hyperkalaemia, hyperuricemia, and hypocalcaemia, together with rasburicase (refer to local prescribing information) to increase urate excretion if needed.

10.8 T cell Aplasia

Hypogammaglobulinaemia has been seen as a consequence of depletion of normal B cells by CAR-T therapy. T cell aplasia may occur because of depletion of normal T cells by AUTO4. Potentially this may increase the risk of infections. However, since only a proportion of the T cell population is being targeted for depletion, and polyclonal nature of the immune response this risk is likely to be low. The duration of partial T cell aplasia is likely to be variable depending on the persistence of CAR T cells.

Patients will be monitored for CD4 and CD8 T-cell levels as well as for infections as per the Schedule of Assessments for CMV, EBV and adenovirus. Other infections such as JCV, toxoplasmosis and fungal infections will also be monitored per institutional guidelines used for bone marrow transplant patients, or as clinically indicated. In the event of active infection, patients will be managed as per the institutional standards or as clinically indicated.

Additionally, where possible, unprocessed leukapheresate will be stored. Should a patient develop complete T cell aplasia or, severe and/or recurrent infections, then the rituximab safety switch can be activated to deplete AUTO4. Since stem cells are not expected to be impacted, normal haematopoiesis should lead to recovery of the T cell compartment. In addition, the unprocessed leukapheresate can be infused to facilitate rapid immune re-constitution. Should this be unsuccessful then the patient could be rescued with an allogenic stem cell transplant. The patients recruited in this study are naïve for allogenic stem cell transplant.

In the event of any impact on B-cells, management of hypogammaglobulinaemia associated with CAR-T therapy will be according to local institutional practice.

10.9 Management of Immune-Related Adverse Events due to On-target but Off-tumour Toxicity

Though it is unlikely that AUTO4 will cause immune-related AEs (irAEs) due to on-target but off-tumour toxicity, the patients will be closely monitored for signs and symptoms indicative of immune related AEs, which may allow for an early recognition of these events. Special attention should be paid to vital organs and immune related AEs of any grade involving vital organs (e.g. lung, brain, and eyes), more aggressive monitoring and rapid institution of appropriate supportive care including systemic steroids should be administered. In case of severe immune related AE, not successfully managed by general supportive care, treatment with steroids and other agents may be considered.

11 CONCOMITANT MEDICATIONS AND THERAPIES

All concomitant medication the patient is receiving at Day -7 (-1 day) or receives during pre-conditioning chemotherapy, or in the 28-day DLT period and up to Month 2 (Day 60) must be recorded. Outside these timeframes (that is, before Day -7 [-1 day] and after 2 months), only concomitant medication given for AUTO4 treatment-related SAEs and treatment-related Grade 3 to 4 AEs, AESIs or study related procedures will be collected.

The following must be recorded:

- Reason for use.
- Dates of administration, including start and end dates.
- Dosage information including dose, route and frequency.

Concomitant medication may be given as medically indicated for AEs, and these details (including doses, frequency, route and start and stop dates) of the concomitant medication/treatment given must be recorded in the patient's medical records and details entered to the eCRF. The period for collection of AE related concomitant medication is the whole study.

Standard drugs required by the patient may be administered alongside the trial protocol.

All safety management guidelines are only recommendations and deviations from this scheme are allowed according to the Investigator's judgement and local institutional practice.

11.1 BRIDGING THERAPY

Patients may receive bridging therapy, between leukapheresis and admission for pre-conditioning therapy, prior to AUTO4 infusion. The dates of bridging therapy, the chemotherapy agents and the doses given must be recorded in the eCRF. Additionally, the intent of bridging therapy, such as prevention of disease progression or induction of response in a rapidly progressing disease, should be documented in the eCRF.

Cytotoxic chemotherapies should be stopped 2 weeks prior to AUTO4 infusion. Other therapies should also have an adequate washout as indicated in exclusion criteria. Patients should have a baseline PET/CT after the bridging chemotherapy is given and prior to starting pre-conditioning.

11.2 ALLOWED CONCOMITTANT MEDICATIONS/THERAPIES

Palliative radiotherapy: Palliative radiotherapy may be given concomitantly as clinically appropriate.

Other permitted therapies: The following medications and supportive therapies are examples of support therapies that may be used during the study:

- Anti-microbials including antivirals and supportive therapy as required to prevent infections.
- Colony stimulating factors (use granulocyte-macrophage colony-stimulating factors [GM CSF] with caution up to 3 weeks after AUTO4 infusion, due to the potential to worsen CRS symptoms. Granulocyte-colony stimulating factor (G-CSF) would be the preferred myeloid growth factor over GM CSF, if medically indicated. The effects of G CSF are unknown and can be used at the physician's discretion).

- Erythropoietin, and transfusion of platelets and red cells.

Pre-and post AUTO4 infusion supportive therapies:

Please refer to Section 10.2.

11.3 PROHIBITED AND CAUTIONARY THERAPIES

Herbal, homeopathic agents or very high dose vitamins and mineral supplements:

No herbal, homeopathic agents or very high dose vitamin and mineral supplements will be allowed between Day -10 and Day 60 following AUTO4 infusion, unless recommended by the Principal Investigator.

Corticosteroids and other immunosuppressants (except for managing treatment-related toxicity):

Patients should not be receiving corticosteroids at doses of >5 mg prednisolone or equivalent within 72 hours of leukapheresis and pre-conditioning chemotherapy administration and, at the time of AUTO4 infusion. The use of immunosuppressants such as high dose corticosteroids, should be avoided where possible, as these are likely to influence the efficacy and possibly safety of AUTO4. Corticosteroids may be used in the context of severe CRS, neurotoxicity, other inflammatory pathologies or infusion reactions (See Section 10). Physicians may use any medication as clinically appropriate and necessary to manage emerging AEs. The use of other immunosuppressants should be discussed with the Sponsor's Medical Monitor.

Anti-cancer therapies:

In general, patients should not receive other anti-cancer therapy or any other investigational drugs after administration of AUTO4. Administration of other systemic anti-cancer therapy at any time will be considered an indicator of treatment failure (progressive disease). However, palliative radiotherapy for symptom control can be administered without necessarily indicating progressive disease. Patients who have been administered AUTO4 and subsequently require alternative anti-cancer therapy will complete the End of Study visit and roll on to a follow-up protocol.

Live vaccinations:

Administration of live vaccinations is generally not recommended. On a case by case basis where specific live vaccines may be appropriate without other options, the Investigator or delegate is to follow institution guidelines and discuss with the patient. Generally, live vaccinations should not be administered unless the T cell lymphocyte count has recovered and for at least 24 months post AUTO4 infusion.

Investigators can use any medication based on their clinical judgement and local institutional practice to optimise patient's safety. All medications should be recorded in the eCRF.

11.4 OVERDOSAGE

There is currently no experience of overdose of AUTO4 as no clinical studies have been performed to date. There is no specific treatment for an overdose of AUTO4. In the event of overdose, any adverse reactions should be treated symptomatically. In the event of unmanageable toxicity, steroids and/or rituximab may be used to deplete AUTO4.

AUTO4 cells will be provided in patient specific dose aliquots and will be administered by trained staff in a hospital setting; therefore, the chance of overdose is unlikely.

11.5 DIETARY AND LIFESTYLE RESTRICTIONS

No dietary restrictions are recommended. A normal balanced diet is recommended; the patient may continue his/her normal diet as appropriate.

12 SAFETY AND PHARMACOVIGILANCE

12.1 DEFINITIONS

12.1.1 Definition of an Adverse Event

An AE is defined as any untoward medical occurrence in a patient or clinical investigation subject administered a medicinal product which does not necessarily have a causal relationship with the treatment.

An AE can therefore be any unfavorable and unintended sign (including an abnormal laboratory finding, for example), symptom or disease temporally associated with the use of a medicinal product, whether or not considered related to the medicinal product.

12.1.2 Definition of an Adverse Reaction

An adverse reaction is any untoward and unintended responses to a medicinal product related to any dose administered. A causal relationship between a medicinal product and an AE is at least a reasonable possibility, e.g. the relationship cannot be ruled out.

An unexpected adverse reaction is an adverse reaction in which the nature or severity of which is not consistent with the Reference Safety Information section outlined in the Investigator Brochure (IB).

12.1.3 Definition of a Serious Adverse Event

A SAE is defined as an AE that meets any of the following criteria:

- **Results in death** (death due to disease progression will not be considered as an SAE).
- **Life-threatening** (the term 'life-threatening' refers to an event in which the patient was at risk of death at the time of the event. It does not include any AE that, had it occurred in a more severe form, might have caused death).
- **Requires in-patient hospitalisation or prolonged existing hospitalisation.**
- **Results in persistent or significant disability/incapacity.**
- **Congenital anomaly/birth defect.**
- **Medically significant** (e.g. important medical events that may not be immediately life-threatening or result in death or hospitalisation but may jeopardise the patient or may require intervention to prevent one of the other outcomes listed above).

If the event was initially non-serious, the onset of the SAE should be considered the time when the AE met the above seriousness criteria. Resolution of the SAE should also be considered when the event no longer is serious and is either a non-serious event or has resolved.

Serious adverse events must be reported by the Investigator to the Sponsor within 24 hours of being made aware of their existence (see Section 12.3.5 for reporting instructions).

Events NOT considered as SAEs:

- Hospitalisations not intended to treat an acute illness or AE (e.g. social reasons such as pending placement in a long-term care facility).

- The administration of blood or platelet transfusion as routine treatment of studied indication*
- A procedure that is planned (i.e. planned prior to starting of treatment on study)*
- An elective treatment of a pre-existing condition unrelated to the studied indication.
- A procedure requiring hospitalisation for protocol/disease-related investigations (e.g. surgery, scans, endoscopy, sampling for laboratory tests, and BM sampling)*
- Emergency outpatient treatment or observation that does not result in admission, unless fulfilling other seriousness criteria (i.e. Medically Significant).
- Routine treatment or monitoring of the indication not associated with any deterioration in condition.
- Hospitalisation or prolongation of hospitalisation for technical, practical, or social reasons, in absence of an AE.
- Extension of planned in-patient hospital stay beyond 30 days for general management purposes / management of underlying disease.
- An event which is part of the natural course of the disease under study (i.e. disease progression or hospitalisation due to disease progression, or pain control, stabilisation of fractures) does not need to be reported as an SAE.
- Death due to the disease under study is to be recorded on the Death Case Report Form and is not considered an Adverse Event, as this is an expected outcome of the disease (See Section 12.1.5).

Note *Hospitalisation or prolonged hospitalisation for a complication of those indicated above remains a reportable SAE.

Suspected Unexpected Serious Adverse Reactions

A suspected unexpected serious adverse reaction (SUSAR) is any suspected adverse reaction related to the study treatment that is both unexpected and serious (see Section 12.1.3). Assessment of expectedness will be made by the Sponsor, or delegated through a vendor, with reference to the Reference Safety Information (RSI) in the Investigator Brochure.

12.1.4 Adverse Events of Special Interest

The following are AESIs:

- Grade 2-5 CRS.
- Grade 2-5 neurotoxicity (including depressed level of consciousness, dysphagia, ataxia, seizures and cerebral oedema).
- Grade 3-4 Infusion Related Reaction to AUTO4

Adverse Events of Special Interest will be reported on an AESI/SAE form to the Sponsor within 24 hours of becoming aware of the event. Events can be both an AESI and a SAE (Section 12.3.6).

12.1.5 Disease Progression

Due to the nature of the disease under investigation, disease progression is an anticipated occurrence in many patients, due to the nature and prognosis of the disease. Disease progression is therefore not considered an adverse event, unless the disease progression is

greater than that which would normally be expected for that patient, or if the Investigator considers that there was a causal relationship between treatment with study medication(s) or protocol design/procedures and the disease progression, then this must be reported as an SAE, due to the medical significance of the event.

12.2 Assessment of Adverse Events

Adverse events will be elicited at each study visit as indicated in the Schedule of Assessments and as clinically necessary. Patients will be instructed to report any AEs occurring between study visits to the study site. Adverse events will be assessed by the Investigator, or appropriately qualified designee, for severity, relationship to study treatment, action taken to AUTO4 treatment (as applicable), outcome and whether the event meets criteria as an SAE according to the guidelines presented in Section 12.1.3.

12.2.1 Severity of Adverse Events

The severity of AEs will be graded according to the NCI CTCAE (version 5.0).

Adverse events that are not defined by the NCI CTCAE should be evaluated for severity according to the following scale:

Table 23: Severity Grading of AEs Not Listed on the NCI CTCAE Grading System

Grade	Severity	
1	Mild	Transient or mild discomfort; no limitation in activity; no medical intervention/therapy required.
2	Moderate	Mild to moderate limitation in activity, some assistance may be needed; no or minimal medical intervention/therapy required.
3	Severe	Marked limitation in activity, some assistance usually required; medical intervention/therapy required, hospitalisation is possible.
4	Life-threatening	Extreme limitation in activity, significant assistance required; significant medical intervention/therapy required, hospitalisation or hospice care probable.
5	Fatal	Death because of this AE.

AE = adverse event.

It is important to distinguish between serious and severity of an adverse event. Severity is a measure of intensity whereas seriousness is defined by the criteria in Section 12.1.3.

Grade 3 or 4 severity does not necessarily imply seriousness. Due to the nature of the patient's disease and treatment Grade 3 and 4 cytopenia (neutropenia, thrombocytopenia and anaemia) are common and are not usually considered medically significant in this context. For other events, there should be a low threshold in considering a grade 3, and particularly a Grade 4 event as serious.

12.2.2 Relationship of Adverse Events to Treatment

The Investigator must determine the relationship between the administration of the study drug and the occurrence of an AE/SAE as defined below:

Relationship assessments classified as "Not Related" to study treatment:

Not Related: The AE is not related to the investigational product. The patient either did not receive the investigational product or the event is related to an

aetiology other than the investigational product (the alternative aetiology must be documented in the study patient's medical record).

Unlikely Related: The AE is doubtfully related to the investigational product.

The event is not clearly related to an identified aetiology other than the investigational product; but there is no plausible mechanism for the event to be related to the investigational product and/or there is no clear association between the event and the administration of the investigational product.

Relationship assessments classified as "Related" to study treatment:

Possibly Related: The AE may reasonably be related to the investigational product.

Probably Related: The AE is likely to be related to the investigational product.

There is an association between the event and the administration of the investigational product, there is a plausible mechanism for the event to be related to the investigational product, the event is less likely to be explained by known characteristics of the patient's clinical status, and an alternative aetiology is not apparent.

Definitely Related: The AE is clearly related to the investigational product.

If an event is assessed as related to a drug other than the investigational product which has not been provided by the Sponsor, the name of the manufacturer must be provided when reporting the event.

12.3 Reporting Procedures

12.3.1 All Adverse Events

The AE Event term recorded in the eCRF, when possible and appropriate, should be the 'medical diagnosis' using the most appropriate medical term. If a diagnosis is not known, then the signs and symptoms should be included. This can be updated later, when a diagnosis has been made.

Signs and symptoms, or additional known associated events (e.g. febrile neutropenia with fever and neutropenia) do not need to be reported as additional event terms and should be described in the narrative. Signs, symptoms or other events, not normally associated with the reported event, should be added as additional events (e.g. febrile neutropenia and liver function abnormal).

All measures required to manage an AE must be recorded in the patient's medical notes and reported accordingly in the eCRF.

Death is an outcome and should not be considered an adverse event, unless no other appropriate diagnosis can be made. This should be updated when a cause of death has been established.

In principle all Adverse Events, including Serious Adverse Events should be collected from the time that that patient consents to the study. However, due to the long period between consent and treatment with AUTO4, any AEs/SAEs related to holding chemotherapy that are not associated with study procedures do not require reporting as study AEs/SAEs. Any significant events should be added to the patient's medical history. All AEs/SAEs related to study procedures (leukapheresis, bone marrow assessments etc.) should be reported.

All AEs/SAEs are to be recorded from admission for conditioning chemotherapy (Day -6 [-1 day]). Due to the patient's disease state and expected effect of chemotherapy on laboratory values, not all laboratory value abnormalities will be considered as adverse events (See Section 12.3.2). Changes to laboratory values are better assessed through individual laboratory measurements.

The reporting period for all AEs is described in Table 24.

Table 24: Reporting Period for All AEs

**From**: ICF signature<br>**Until**: Month 6	**From**: Month 6<br>**Until**: End of Study/patient withdrawal<br>OR, when patient initiates a new treatment for their disease
• All AEs<br>• All SAEs<br>Note: Events associated with disease progression do not need to be reported as AEs.	• All AEs (regardless of grade) considered related to AUTO4<br>• All AEs of special interest (regardless of relationship to AUTO4) (Section 12.3.6)<br>• All AEs related to study procedures regardless of relationship to AUTO4<br>• All SAEs (regardless of relationship to AUTO4)<br><br>If a patient starts a new treatment for disease progression or undergoes a SCT:<br>• Only AEs (serious and non-serious) that are considered related to AUTO4 to be reported.

AE = adverse event; ICF = informed consent form; SAE = serious adverse event; SCT = stem cell transplant.

The Investigator should follow each AE until either:

- The AE has resolved to baseline
- The AE is assessed as stable by the Investigator
- Patient is lost to follow-up
- Patient withdraws consent
- Death

If a drug related AE and/or any SAE is ongoing at the time of study completion (2 years after dosing or disease progression, whichever happens first) the event will be followed until resolution, is assessed as stable by the Investigator, or one of the following apply:

- Death
- Withdrawal of consent
- Start of new treatment or
- Patient lost to follow-up

Patients who enter a long-term safety study will have their events considered continuing at the completion of this study, and the event will be followed up, as applicable, in the long-term safety study.

12.3.2 Adverse Events Associated with Laboratory Values

Anaemia, neutropenia, thrombocytopenia requiring blood product support are anticipated (due to disease and preconditioning) and are therefore not considered adverse events, unless they persist beyond Day 30 after CAR-T infusion or are considered clinically significant and/or require advanced intervention above blood product and G-CSF support.

Grade 1-2 laboratory abnormalities, at any time point are extremely frequent in this patient population and are too not considered adverse events unless felt to be clinically significant by the Investigator.

Whenever possible, AEs associated with laboratory values, should have these values included in the eCRF, e.g. reporting of AEs such as febrile neutropenia should be associated with temperature reading entry in vitals and Absolute Neutrophil Counts (ANC) in laboratory sections of the eCRF.

It should be noted that clinically, a single laboratory measurement may not have any clinical significance and may need to be taken in the context of other observations.

Abnormal laboratory values may be deemed clinically significant, by the investigator, if either of the following conditions is met:

- The abnormality suggests a disease and/or organ toxicity that is new or has worsened from baseline, in the context of the patient's disease state and recent chemotherapy and/or CAR-T therapy.
- The abnormality is of a degree that requires additional active management, e.g., closer observation, more frequent follow-up assessments, or further diagnostic investigation.
- Prolonged Grade 4 cytopenia lasting more than 60 days or those considered medically significant or requiring significant management in addition to transfusions and growth factor support (e.g. stem cell top up or transplant) should be reported as SAEs.

If the abnormal laboratory value, or values, indicate a specific medical condition, or diagnosis, then this medical condition, rather than the laboratory result should be used as the AE reported term (See Section 12.3.1).

12.3.3 Adverse Events Associated with Cytokine Release Syndrome (CRS)

As CRS can present with multiple signs and symptoms, it is important to assess the character and nature of these occurrences. Therefore, as well as entering the event of 'Cytokine Release Syndrome', with the appropriate grading (See Section 10.4) in the eCRF, please also add the associated adverse events (or signs and symptoms), as separate AEs, with the appropriate CTCAE grading as applicable (e.g. fever, hypotension etc.).

12.3.4 Adverse Events Associated with CAR-T Neurotoxicity (ICANS)

As Immune Effector Cell-associated Neurotoxicity Syndrome (ICANS) can present with multiple signs and symptoms, it is important to assess the character and nature of these occurrences. Therefore, as well as entering the event of 'ICANS', with the appropriate grading (See Section 10.6) in the eCRF, please also add the associated adverse events (or signs and symptoms), as separate AEs, with the appropriate CTCAE grading as applicable (e.g. confusion, aphasia, encephalopathy, seizure).

12.3.5 Serious Adverse Events Reporting

All SAEs (as described in Section 12.3.1) occurring during the study must be reported to the Sponsor within 24 hours of becoming aware of the event. Additional or follow-up SAE reports should be submitted with relevant information promptly. Should a regulatory authority require the Sponsor to submit additional data on the event, the Investigator will be requested to provide additional data to the Sponsor promptly.

Information to be Provided for an SAE:

Although much information will have been provided in the eCRF, this information may not always be readily available at the time of evaluating each Serious Adverse Event, or when reporting to the regulatory authorities, so it is essential that as much relevant information is included on the AESI/SAE form. At a minimum, the following is required.

- An identifiable patient (patient number)
- Reporter details
- The AUTO product (if not clear on the AESI/SAE form and if administered)
- The Protocol (if not clear on the AESI/SAE form)
- The AE, with a description of the event and a causality assessment

Please also ensure that this information is accurate, at the time of reporting. Missing information, or information that is inconsistent with the eCRF will automatically generate queries. The initial SAE report will be provided to the Sponsor (or designee) using the AEI/SAE form. The Sponsor or designee will require the following information related to the event:

The SAE form completion and reporting must not be delayed, even if all of the information is not available at the time of the initial report.

The AESI/SAE report should be submitted to the following by fax or email:

SAE Fax number: [redacted]

Email: [redacted]

The 24-Hour Safety Hotline: [redacted]

[redacted]

[redacted]

[redacted]

[redacted]

Fax numbers and email address are listed on the SAE form and in the SAE form completion guidelines.

SAE Follow-up Reporting:

After the initial SAE report, the Investigator is required to provide additional follow-up information on the SAE by submitting an updated SAE report form to the Sponsor (or designee). New significant information includes, but is not limited to, the following:

- New signs or symptoms or a change in the diagnosis
- Significant new diagnostic test results

- Change in causality based on new information
- Change in the event's outcome, including recovery
- Additional narrative information on the clinical course of the event

Serious adverse events must be followed through to resolution by the Investigator. Resolution is defined as a return to baseline status, Grade 1, or stabilisation of the condition with the expectation that it will remain chronic. For all SAEs, the Investigator may be requested to obtain additional information in an expedited manner. This additional information will allow for a complete medical assessment of the case and independent determination of possible causality. Information on other possible causes such as concomitant medication and illnesses must be provided.

The Medical Monitor may specify a longer period of time, if required, to assure the safety of the patient.

Suspected Unexpected Serious Adverse Reaction Reporting

If the event is evaluated as a SUSAR, i.e. unexpected events that are related (reasonable possibility) to AUTO4, the Sponsor will submit the SUSAR to the regulatory authorities and the Research Ethics Committee within 7 calendar days for initial reports of fatal/life-threatening events (with a follow-up report within a further 8 calendar days) and 15 calendar days for all other events, or in accordance with local regulatory requirements, if different. Where there are conflicting evaluations of causal relationship, between the Investigator and the company, the more conservative will be used for reporting purposes.

All reporting to regulatory authorities will be by Autolus, or through an Autolus designated vendor.

The Sponsor (or designee) will notify Investigators of all SUSARs, in accordance with local regulations. The Investigator must immediately review with the Investigator's site team and retain the documentation in the Investigator Site File.

12.3.6 Adverse Events of Special Interest Reporting

Adverse events of special interest occurring during the study must be reported to the Sponsor within 24 hours of becoming aware of the event using the AESI/SAE form. Additional or follow-up information should be submitted with relevant information promptly.

The AESI/SAE form should be completed and faxed or emailed to the sponsor or designee (Section 12.3.5) within 24 hours.

12.3.7 Pregnancy Reporting and Management

Pregnancies

Female patients of childbearing potential will be instructed to immediately inform the Investigator if they become pregnant during the study or within 12 months after the last dose of study treatment. Any pregnancies during this period must be reported to the Sponsor (or designee) within 24 hours of knowledge of the event using the pregnancy report form (fax numbers and email address are listed on the bottom of the form).

While pregnancy itself is not considered to be an AE or SAE, any pregnancy complication or elective termination of a pregnancy for medical reasons will be recorded as an AE or SAE. Abnormal pregnancy outcomes (e.g. spontaneous abortion, foetal death, stillbirth, congenital

anomalies, and ectopic pregnancy) are considered SAEs and must be reported to the Sponsor immediately using the SAE form (i.e. no more than 24 hours after learning of the event).

The Investigator must counsel the patient, discussing the risks of the pregnancy and the possible effects on the foetus. Monitoring of the patient should continue until conclusion of the pregnancy.

Pregnancies in Female Partners of Male Patients

Male patients will be instructed to immediately inform the Investigator if their partner becomes pregnant during the study or within 12 months after the last dose of study treatment. Any pregnancies during this period must be reported to the Sponsor (or designee) within 24 hours of knowledge of the event using the pregnancy report form (fax numbers and email address are listed on the bottom of the form).

Attempts should be made to collect and report details of the course and outcome of any pregnancy in the partner of a male patient exposed to study treatment. The pregnant partner will be asked to sign an authorisation for use and disclosure of pregnancy health information. The Investigator may provide information on the risks of the pregnancy and the possible effects on the foetus, to support an informed decision in cooperation with the treating physician and/or obstetrician.

Outcome

Additional information on the course and outcome of the pregnancy should be provided to the Sponsor (or designee) as soon as becoming available (i.e. no more than 24 hours after obtaining the information) using the paper pregnancy report form. The following pregnancy outcomes are SAEs and should be reported according to the procedure in Section 12.3.5.

- Spontaneous abortion (as the Sponsor considers spontaneous abortions to be medically significant events).
- Any congenital anomaly/birth defect in a child born to a female patient or female partner of a male patient.
- All neonatal deaths occurring within 30 days of birth should be reported as SAEs, without regard to causality.

Follow-up information regarding the outcome of the pregnancy and any postnatal sequelae in the infant will be required.

12.3.8 Overdose Reporting and Dosing Errors

Safety information on the study drug may require expedited reporting and/or safety evaluation. This will include:

- Overdose of a study drug
- Suspected abuse/misuse of a study drug
- Inadvertent or accidental exposure to a study drug
- Medication error involving a study drug

A study drug overdose is the accidental or intentional use of AUTO4 in an amount higher than the dose being studied. An overdose or incorrect administration of study drug is not an AE unless it results in untoward medical effects. Any AUTO4 overdose or incorrect administration of AUTO4 should be noted on the corresponding eCRF page. All AEs associated with an

overdose or incorrect administration of AUTO4 should be recorded on the AE eCRF page. If the associated AE fulfils the criteria for a SAE, the event should be reported immediately (i.e. no more than 24 hours after learning of the event; see Section 12.3.5).

13 OVERSIGHT COMMITTEES

13.1 SAFETY EVALUATION COMMITTEE

Patient safety will be monitored throughout all parts of the study by a SEC established by the Sponsor. This committee will monitor treatment-emergent data on an ongoing basis throughout study conduct, for the purpose of ensuring the continued safety of patients enrolled in this study.

The SEC will be chaired by the Sponsor's Medical Monitor and membership will include Study Investigators, a statistician and biomarker representative, along with additional Sponsor staff as appropriate. The team will meet at regular frequencies throughout study conduct:

During Phase I (dose escalation), the SEC will meet:

1 After the first patient in each cohort completes 28 days.

2 After the third and/or sixth patient in each cohort completes the DLT evaluation period of 28 days to make the dose escalation decision.

3 Additional ad hoc meetings if safety stopping criteria are met.

4 When clinically necessary based on emerging data.

During Phase II (dose expansion), the SEC will meet:

1 On a periodic basis (not exceeding 6 months) to assess cumulative safety data.

2 After 10, 20, and 30 patients have completed 28 days following AUTO4 infusion.

The Sponsor will maintain documentation of meeting outcomes. Decisions with the potential to impact patient safety (e.g., an unfavourable change in the risk/benefit assessment) will be promptly communicated to Investigators, ethics committees, regulatory authorities and patients, as appropriate. Throughout the trial, information regarding all SAEs and potential DLTs will be sent to the SEC members. Dose limiting toxicities will be monitored and the decision to assign the RP2D (the optimal dose) will be taken by the SEC.

Dose escalation decision in Phase I of the study will be made by the SEC and may be subject to a substantial protocol amendment. The schedule of dose escalation meetings will depend on the time to completion of a cohort, frequency of DLT and when an RP2D is determined. The SEC may stop further enrolment into one or more of the cohorts if treatment-emergent toxicity is determined to result in an unfavourable change in patient risk/benefit. Decisions and/or recommendations made by the SEC will be communicated to the Principal Investigators at all active study centres and to the Sponsor.

Mandatory SEC processes will be included in a SEC Charter.

13.2 INDEPENDENT DATA MONITORING COMMITTEE (IDMC)

An IDMC consisting of two independent physicians and one statistician will be established by the Sponsor and they will review serious safety events. The decision of the IDMC will supersede that of SEC. The IDMC will meet upon occurrence of the following:

- When any safety stopping, criteria (Section 3.6) are met.
- During Phase I – once during Cohort 1. This may be via telephone or correspondence.

- Prior to opening Phase II.
  - At the interim analysis of Phase II (after 10 patients have been treated and had an opportunity for a minimum of 12 weeks post treatment follow-up)
- Annually (6 monthly during active enrolment) during Phase II to review cumulative safety data.

Throughout the trial, information regarding all treatment emergent SAEs and DLTs will be sent to the IDMC members to keep them informed on emerging safety findings. The IDMC can ask for and will be provided with any additional information relevant to the SAEs and DLTs.

The IDMC will receive all dose evaluation/escalation meeting minutes (as soon as possible following the meeting) and will have the opportunity to review and can over-rule the SEC decision if clinically warranted and necessary.

When a RP2D decision is made by the SEC, the decision will need to be reviewed and endorsed by the IDMC prior to opening Phase II.

Prior to any meeting or upon occurrence of a stopping criteria, detailed event summaries and cumulative safety data will be sent to the IDMC. A recomendation to continue or to hold or modify the study will be made by the IDMC to thesponsor. If, and when a study is halted, it will be re-started after a substantial protocol amendment has been approved by the regulatory authorities and ethics committees.

Recommendations made by the IDMC will be communicated to the Principal Investigators at all active study centres and to the Regulatory Authorites, as applicable, by the Sponsor.

Mandatory IDMC processes will be included in the IDMC Charter.

14 STATISTICS

Further details of the statistical analysis of all the endpoints will be included in a separate Statistical Analysis Plan. Any analysis that deviates from the Statistical Analysis Plan will be documented and justified in the Clinical Study Report.

14.1 SAMPLE SIZE ESTIMATION

It is anticipated that approximately 200 patients are expected to be screened for eligibility for entry to this study (this includes both Phase I and II). This assumes that approximately 35% of patients will be identified as TRBC1 positive (a small number of patients will not provide sufficient evaluable tissue) Of those that are identified as TRBC1 positive, it is assumed that approximately 20% will fail the AUTO4 manufacture and/or will not continue to meet the inclusion and exclusion criteria. Up to 55 patients in total are anticipated to be treated with AUTO4 therapy.

- **Phase I (Escalation):** Up to 25 patients in total (three to six patients per dose cohort), following a rolling six design (Skolnik et al. 2008).
- **Phase II (Expansion):** Dose expansion: up to 30 evaluable patients in total, using a Simon's two-stage optimal design.

Using Simon's 2-stage design (Simon 1989) the null hypothesis that the true response rate is 10% will be tested against a one-sided alternative. In the first stage, 10 evaluable patients will be accrued (12 weeks post treatment of tenth evaluable patient). If there is only one or no responses in these 10 evaluable patients, the study will be stopped. Otherwise, if two responses or more are confirmed before the end of the first stage, the recruitment will continue, and 19 additional evaluable patients will be accrued for a total of 29. The null hypothesis will be rejected if six or more responses are observed in 29 evaluable patients. This design yields a type I error rate of 5% and power of 80% when the true response rate is 30%.

14.2 DESCRIPTION OF ANALYSIS DATASETS

14.2.1 Enrolled Set

The Enrolled set will consist of all patients registered and enrolled into the study. The number of patients who fail screening, together with the reason for screen failure will be summarised.

14.2.2 Infused Set

The Infused Set comprises all patients who have received at least one infusion of AUTO4 treatment.

14.2.3 Safety Set

The Safety Set comprises all patients who receive at least one dose (complete or partial dose) of AUTO4 treatment.

14.2.4 Efficacy Analysis Set

All patients in the Infused Set with PET-positive disease prior to start of pre-conditioning therapy will be included in the Efficacy Analysis Set (EAS). Patients with PET-negative

disease after bridging chemotherapy will not be included in the primary efficacy analysis set. Patients who did not receive bridging therapy and did not repeat disease assessment prior to pre-conditioning will be included if there is PET positive disease prior to enrollment.

14.3 STATISTICAL ANALYSES AND METHODS

Continuous data will be summarised using the mean, median, standard deviation, minimum and maximum, while frequency counts and percentages will be presented for discrete variables. Time to event endpoints will be summarised using Kaplan-Meier method. Summary statistics will be presented for baseline characteristics.

In all analyses, the Study day refers to the study day from AUTO4 infusion defined per CDISC (Clinical Data Interchange Standards) conventions as:

- (date of event/assessment - date of first AUTO4 infusion + 1), if event is on or after the date of first AUTO4 infusion
- (date of the event/assessment - date of first AUTO4 infusion), if event precedes the date of first AUTO4 infusion. In this case, the study day will be negative.

Note: In terms of statistical analysis only, the date of AUTO4 infusion will be considered Day 1 under the CDISC analysis convention.

14.3.1 Primary Endpoints

The primary endpoints of the study are as follows:

Phase I: Safety and RP2D

- Incidence of Grade 3 to 5 toxicity occurring within 60 days of AUTO4 infusion.
- Frequency of DLT occurring within 28 days of AUTO4 infusion.

Phase II: Anti-tumour effects

- Overall response rate (CR+PR), where the denominator will be the number of patients in the EAS.

14.3.1.1 Phase I: Safety

Safety associated with AUTO4 administration (only those who received AUTO4).

Summary statistics and analyses will be provided by dose level and overall. The safety analysis set for the study and study treatment will be used for the analysis of safety data.

Safety evaluations will be based on the incidence, severity and type of AEs, and changes in the patient’s vital signs and clinical laboratory results.

Adverse events will be coded using the Medical Dictionary for Regulatory Activities (MedDRA) AE coding system for the purpose of summarisation. All AEs occurring in the study will be listed in by--patient data listings. The following AEs will be tabulated:

- Treatment-emergent AEs, where “treatment-emergent” is defined as any AE that occurs during or after administration of AUTO4 up to 60 days after the infusion.

- Adverse events that are considered drug-related regardless of the start date of the event, or any event that is present at baseline and continues after the first dose of study treatment but worsens in intensity.
- Adverse events that are considered related to treatment (possibly, probably, or definitely related).
- Adverse events of special interest, together with grade.
- Adverse events by the NCI CTCAE toxicity grade will also be summarised.
- Deaths and SAEs will be tabulated in data listings including additional relevant information on the patient.

Laboratory toxicity grades will be calculated for the appropriate laboratory parameters according to NCI CTCAE version 5.0.

Adverse events of special interest will be analysed in greater depth, including the time to onset and time to resolution where appropriate.

14.3.1.2 Phase I: RP2D and MTD

At the end of the Phase I dose escalation phase, the RP2D will be identified based on the safety data and evaluation of the activity data collected in the dose escalation phase. The RP2D may also be determined prior to completion of all cohorts in Phase I.

14.3.1.3 Phase II: Anti-tumour effects

The primary objective in the Phase II part of the study is to evaluate the clinical efficacy of AUTO4 treatment. The primary endpoint is the overall response rate (ORR), defined as proportion of patients who achieve a best overall response (BOR) of CR or PR as assessed per Lugano criteria. The primary analysis will be performed on the EAS. The associated two-sided 95% confidence interval will be presented.

Patients in the study who are of unknown clinical response will be treated as non-responders. Patients who have a non-evaluable scan or with PET-negative disease prior to pre-conditioning therapy will be excluded in the EAS and hence excluded in the primary analysis of efficacy.

14.3.2 Secondary Endpoints

Objective: Safety and tolerability of AUTO4

- Frequency and severity of all AEs and SAEs.
- Incidence and severity of opportunistic infections following AUTO4 infusion.

Objective: Feasibility of AUTO4 manufacture in this patient population (all patients)

Feasibility of product generation will be examined by assessing the number of patients for whom AUTO4 was successfully manufactured as a fraction of the number of patients undergoing leukapheresis (all patients who are TRBC1 positive and undergone leukapheresis).

Objective: Clinical efficacy of AUTO4

- Determine the CR rate following treatment with AUTO4.

- The number of patients achieving objective response within 1, 3, & 6 months post AUTO4 infusion. The median time to response (PR+CR) and to CR will be calculated.
- Response rate per histology will be summarised.
- Evaluate clinical outcomes including DOR, PFS, and OS.
  - Duration of response will be calculated from the date of first observation of CR or PR, to the date of progression, relapse or death, for patients who are considered as responders. Patients who have not progressed, relapsed or died will be censored at the last adequate disease assessment.
  - Progression-free survival will be calculated from the date of AUTO4 treatment to the date of progression, relapse, or death. Patients who have not progressed or relapsed will be censored at the last adequate disease assessment.
  - Overall survival will be calculated from the date of AUTO4 treatment to the date of death. Patients who have not died will be censored at the date of last contact (clinic visit or telephone contact).
- Time to response will be calculated from the start date of treatment to the date of first observation of CR or PR for patients who are considered as responders.
- Time to CR will be calculated from the start date of treatment to the date of first observation of CR for patients who are considered as complete responders

Of Note: In the Phase 1 portion of the study, the same analysis conventions specified for the primary efficacy of Phase 2 will apply.

Objective: Biomarker, pharmacodynamics and pharmacokinetic effects of AUTO4

The following analyses on the pharmacodynamic and pharmacokinetic effects of AUTO4 will be performed for patients who received AUTO4:

- Duration of TRBC1 positive cell aplasia as determined by flow cytometry in the peripheral blood.
- Expansion and persistence of RQR8/aTRBC1-CAR positive T cells as determined by PCR and flow cytometry will be summarised using the appropriate statistical methods:
  - Expansion is defined as the maximum level of RQR8/aTRBC1-CAR expression in both PCR and flow cytometry assays during the treatment stage and follow-up.
  - Engraftment is defined as detection of any level of RQR8/aTRBC1-CAR expression in circulating T cells (i.e. PBMCs) by PCR and/or flow cytometry following infusion above baseline controls.
  - Persistence is defined as the duration of detectability, from infusion to the first negative result.
- The cellular kinetics of RQR8/aTRBC1-CAR positive T cells will be documented over time for each patient and the area under the curve calculated, then summarised for all patients.

14.3.3 Exploratory Objectives

Timing and magnitude of cytokine release, evaluated in serum using a cytokine assay:

- Data on timing (kinetic of change) will be summarised as the mean (or median) number of days post-infusion.
- Magnitude – kinetic and peak of cytokine levels, e.g., TNFα, IL-6 and IFN-γ (pg/mL), will be plotted for each patient and summarised.

14.3.4 Immunogenicity Data

Analyses of immunogenicity data will be documented in the SAP (Statistical Analysis Plan).

14.3.5 Other Analyses

Not applicable.

14.3.6 Interim Analyses

At the end of Phase I, the safety data will be summarised and reviewed by the SEC. The Phase II dose recommendation will be reviewed and endorsed by the IDMC prior to commencement of Phase II.

In Phase II of the study, an interim analysis will be performed after 10 evaluable patients are treated and had an opportunity for a minimum of 12 weeks post treatment follow-up. The study will be stopped in this first stage if no more than one response has been observed. If the response rate exceeds the interim analysis stopping criteria, the study will continue to full enrolment. Even if ≥ two patients are known to have responded prior to 10 evaluable patients having been treated, the formal interim analysis will still be performed after the tenth evaluable patient.

Recruitment will continue during the period of follow-up and during the interim analysis.

14.3.7 Final Analysis

If the study continues beyond the interim analysis, the final Phase II statistical analysis will be performed after up to 30 evaluable patients have had the opportunity to be followed for a minimum of 24 weeks (6 months) or earlier if the last patient has died or prematurely withdrawn.

Subsequent statistical analyses will be considered, as appropriate, up to the point of AUTO4-TL1 Last Patient Last Visit (24-month follow-up of the last patient entered).

15 STUDY COMPLETION, PREMATURE DISCONTINUATION AND WITHDRAWAL FROM AUTO4-TL1

15.1 COMPLETION OF STUDY

Patients enrolled will be considered to have completed the study when they have completed all assessments until the EoS visit (LPLV – 24 months after last patient dosed).

15.2 PATIENT WITHDRAWAL FROM AUTO4-TL1

A patient should be withdrawn before the end of the study if the following happens:

- Withdrawal of consent
- Lost to follow-up
- Non-compliance with study procedures
- The Sponsor terminates the study
- Death

The date of withdrawal will be recorded as the EoS visit or the last recorded visit. The reason for discontinuation/withdrawal is to be documented in the eCRF and in the source document. If a patient is lost to follow-up, every reasonable effort must be made by the study site personnel to contact the patient and to contact the patient's family doctor/general practitioner to obtain information on the patient's status to determine the reason for withdrawal. The measures taken to follow-up must be documented in the source documents and include steps taken to contact the patient, e.g. dates of telephone calls, registered letters, etc.

Future data collection and withdrawal of consent: Informed consent is a continuous and dynamic process. Patients are free to withdraw their consent at any point in the clinical study and without detriment to their future medical care. However, patients who wish to withdraw their consent from further intervention/study visits may be willing to be followed in the clinical trial so as to enable the collection of key safety information such as (but not limited to) follow-up information on SAEs. In this case, the Investigator will discuss this possibility with the patient to continue in the study and be contacted by a suitable means of communication agreeable to the patient to enable collection of information (survival status and evaluation of safety). In this event, the details should be recorded in the patient's hospital records and only key information such as (but not limited to) date of death will be entered into the eCRF thereafter. The above information will be presented in the PIS/ICF Part B to ensure the patient understands the available options.

Withdrawal from the use of samples in future research: The patient may withdraw consent for use of samples for future research. In such a case, samples will be destroyed after they are no longer needed for the clinical study. Details of the sample retention for research are presented in both PIS/ICF Part A and Part B.

15.2.1 Procedures for Handling Premature Withdrawal from the Study

A patient may, of their own volition, withdraw their consent at any time during the course of the study without any resulting detriment. All data collected up to the point of withdrawal will be maintained in the study database and included in subsequent analyses, as appropriate. Where a patient is withdrawn from the trial at their own request, or based on a decision of the

Investigator, the follow-up should be maintained for safety review, subject to the continuing consent of the patient. The Investigator will discuss the arrangements for withdrawing from any further study interventions and continuing to be followed for safety purposes.

If a patient is lost to follow-up at a site, every effort should be made to contact the patient's family doctor/general practitioner to obtain information on the patient's status.

In the event that AUTO4 does not meet the specifications of the release criteria, the case will be discussed with the Investigator. It may be necessary to generate AUTO4 again if time permits (and biological screening repeated if necessary), otherwise the patient will be withdrawn from the study.

15.3 REPLACEMENT POLICY

In Phase I, patients that have disease progression prior to completion of the DLT evaluation period or who are withdrawn from the study for reasons other than toxicity may be replaced, unless the SEC concludes that the patient is evaluable for dose escalation decision making.

16 END OF TRIAL/TERMINATION BY SPONSOR

For clinical trials located in the EU, a declaration of the end of the clinical trial will be made according to the procedures outlined in Directive 2001/20/EC, Article 10(c); for those countries outside the EU, local regulations will be followed.

The study will be considered complete when the last patient completes their final follow-up visit. A final Clinical Study Report will be provided to the relevant authorities within 12 months after the end of the study.

The Sponsor reserves the right to terminate the study at any time for reasonable medical or administrative reasons. Any premature discontinuation of the study will be appropriately documented per local requirements (e.g., IEC/IRB, regulatory authorities). The study will be stopped in Phase II at the interim analysis if ≤1 patient (out of 10) has an objective response.

In addition, the Investigator or the Sponsor has the right to discontinue a single site at any time during the study for medical or administrative reasons such as:

- Significant non-compliance with contractual enrolment timelines and targets.
- Serious or continued Good Clinical Practice (GCP) non-compliance.
- Inaccurate, incomplete, or delayed data collection.
- Failure to adhere to the study protocol.
- Failure to provide requested follow-up information for data queries.

17 ETHICAL AND REGULATORY CONSIDERATIONS

17.1 ETHICS COMMITTEE REVIEW AND APPROVAL

The final study protocol, including the final version of the written PIS/ICF Part A and Part B, and any other relevant patient facing material, must be approved, or given a favourable opinion in writing by an IEC/IRB as appropriate.

If it is necessary to amend the protocol or the PIS/ICF Part A or Part B during the study, an IEC/IRB approval of the amended protocol and/or relevant PIS/ICF must be obtained prior to implementation of the amended procedures and before new patients are consented to participate in the study using the amended version of the relevant PIS/ICF.

17.2 REGULATORY AUTHORITY REVIEW AND APPROVAL

The study will not commence before approval from the regulatory authority has been granted according to local requirements. The Sponsor (or designee) will be responsible for the preparation, submission, and confirmation of receipt of any regulatory authority approvals required prior to release of study treatment for shipment to the study site.

During the study, the Sponsor (or designee) is also responsible for submitting subsequent amendments and notifications to the regulatory authority according to local requirements.

17.3 INVESTIGATOR RESPONSIBILITIES

17.3.1 Overall Responsibilities

The Investigator is responsible for conducting the study in full accordance with the clinical study protocol, the latest revision of the Declaration of Helsinki, the GCP: Consolidated Guideline, and all applicable national and local laws and regulations for clinical research. Information regarding any investigational sites participating in this study that cannot comply with these standards will be documented and appropriate actions taken. For studies conducted in the EU/European Economic Area countries, the Investigator will ensure compliance with the EU Clinical Trial Directive [2001/20/EC]. For studies conducted in the United States or under a United States Investigational New Drug, the Investigator will additionally ensure adherence to the basic principles of "Good Clinical Practice" as outlined in the current version of 21 Code of Federal Regulations, subchapter D, part 312, "Responsibilities of Sponsor and Investigators", part 50, "Protection of Human Subjects", and part 56, "Institutional Review Boards".

17.3.2 Site Review

Prior to the study start, the Investigator is required to sign a Protocol signature page confirming his/her agreement to conduct the study in accordance with these documents and all the instructions and procedures found in this Protocol, and to give access to all relevant data and records to Clinical Research Associates, auditors and regulatory authorities as required. Investigators ascertain that they will apply due diligence to avoid protocol deviations.

The Investigator will make appropriate reports on the progress of this study to the Sponsor or its designee in accordance with applicable government regulations and their agreement with the Sponsor/Contract Research Organisation.

17.3.3 Informed Consent

In this study, there are two separate informed consents. Firstly, the patient is asked to consent to provide an archival tumour tissue sample or have a new tumour tissue biopsy to determine their T-NHL TRBC1 status (PIS/ICF Part A); secondly, having been confirmed as having TRBC1 positive disease, the patient is asked to consent to the main study (PIS/ICF Part B). It is the responsibility of the Investigator to obtain written informed consent from patients prior to conducting any study-related procedure. All consent documentation must be in accordance with applicable regulations and International Council on Harmonisation (ICH) GCP. Each patient is requested to sign and date the relevant and appropriate ICF after she/he has received and read the relevant and appropriate PIS and received an explanation of what the screening process involves and what the main study involves, including but not limited to: the objectives, potential benefits and risk, inconveniences and the patient's rights and responsibilities. Patients will be given sufficient time to consider PIS/ICF Part A before signing and dating the ICF. Patients may be given both PIS/ICF Part A and Part B at the same time; however, the patient cannot provide consent to the main study until their disease is confirmed as TRBC1 positive. Once a patient is confirmed as TRBC1 positive, adequate time to evaluate the information and to have any questions answered should be given before signing and dating the PIS/ICF Part B.

It will also be explained to the patients that they are free to refuse entry into the study at any time and without prejudice to future treatment. Each ICF must also be signed and dated by the person obtaining consent. The original signed ICF(s) for each patient will be retained on file by the Investigator and the second signed original(s) will be given to the patient.

Informed consent forms must be retained for all screened patients and must be available for verification by the Study Monitor at any time.

In the event of changes to the ICF(s) during the study, the Investigator must always use the most current IEC/IRB approved form for documenting written informed consent for ongoing and new patients.

17.3.4 Delegation of Investigator Duties

The Investigator should ensure that all persons involved in the clinical study are adequately qualified, informed about the protocol, any amendment to the protocol, the study treatments, and their study related duties and functions before any involvement takes place. Delegation of any study related duties and documentation of training performed will be recorded in the signature and delegation log.

17.3.5 Communication with Independent Ethics Committee/Institutional Review Board

The Sponsor or its designee is responsible for assisting the Investigator with applicable documentation for communication with IECs/IRBs. Before initiating a trial, the Investigator/institution or Sponsor should have written and dated approval of the study protocol, the patient ICF(s), any written information to the patients, patient recruitment procedures (e.g., advertisement if applicable), the IB, Investigational Medicinal Product labelling (if applicable) and the coordinating Investigator's curriculum vitae submitted to the relevant IEC/IRB for evaluation before the study start. The IEC/IRB's unconditional approval

statement will be acknowledged by the Sponsor or be transmitted by the Investigator to the Sponsor or a designee prior to shipment of AUTO4 to the site. This approval must refer to the study by exact protocol title and number, and should identify the documents reviewed and the date of review.

During the trial, the Investigator or designee is responsible for forwarding the applicable documents (including protocol deviations, protocol amendments, ICF(s) changes or revisions of other documents originally submitted for review; serious and/or unexpected adverse experiences occurring during the study; new information that may affect adversely the safety of the patients or the conduct of the study; annual updates and/or request for re-approval) for approval and/or review and when the study has been completed. The Investigator or designee will follow national and local requirements.

The Investigator must supply the Sponsor with a copy of the list of members of the IEC/IRB and the letter(s) of approval(s) defining the version of each document approved.

17.3.6 Confidentiality of Trial Documents and Patient Records

The Investigator must ensure that patients' anonymity will be maintained and that their identities are protected from unauthorised parties. The Sponsor will maintain confidentiality standards by assigning a unique coded identification number to each patient included in the study. Patient names will never be included in data sets that are transmitted to the Sponsor or their representatives, or to third parties as permitted by the ICF(s).

On eCRFs or other documents submitted to the Sponsor, patients will not be identified by their names, but by an identification code. The Investigator will keep a patient enrolment log relating codes to the names of patients. The Investigator will maintain documents not for submission to the Sponsor, e.g., patients' written consent forms, in strict confidence. Records will not be destroyed without giving the Sponsor prior written notice and the opportunity to further store such records, at the Sponsor's cost and expense.

After obtaining a patient's consent, the Investigator will permit the study monitor, independent auditor, or regulatory agency personnel to review the portion of the patient's medical record directly related to the study. This shall include all study relevant documentation (e.g., patient medical history to verify eligibility, laboratory test results, admission/discharge summaries for hospital admissions occurring while the patient is enrolled in the study, and autopsy reports for deaths occurring during the study).

Patient medical information obtained by this study is confidential and may only be disclosed to third parties as permitted by the ICF(s) signed by the patient, unless permitted or required by law.

17.4 LONG-TERM RETENTION OF SAMPLES FOR ADDITIONAL FUTURE RESEARCH

Samples collected in this study may be stored by Autolus Ltd. for up to 15 years (or according to local regulations) for additional research. Samples will only be used to further understand AUTO4, to understand advanced cancers, to understand differential drug responders, and to develop tests/assays related to AUTO4 and advanced cancer. The research may begin at any time during the study or the post-study storage period.

Stored clinical samples will be coded throughout the sample storage and analysis process and will not be labelled with personal identifiers. Good Manufacturing Practice AUTO4-derived

samples will be stored according to local GMP rules and procedures. Patients may withdraw their consent for their samples to be stored for research.

18 ADMINISTRATIVE REQUIREMENTS

18.1 DATA QUALITY CONTROL AND ASSURANCE

18.1.1 Compliance with the Protocol and Protocol Revisions

The study shall be conducted as described in the approved protocol. All revisions to the protocol must be discussed with and be prepared by the Sponsor. The Investigator should not implement any deviation or change to the protocol without prior review and documented approval/favourable opinion from the IEC/IRB of an amendment, except where necessary to eliminate an immediate hazard(s) to the study patients.

If a deviation or change to a protocol is implemented to eliminate an immediate hazard(s) prior to obtaining IEC/IRB approval/favourable opinion. The deviation or change will be submitted as soon as possible to:

- The IEC/IRB for review and approval/favourable opinion.
- The Sponsor.
- Regulatory authority(ies) if required by local regulations.

Documentation of approval signed by the chairperson or designee of the IEC(s)/IRB(s) must be sent to the Sponsor.

If an amendment substantially alters the study design or increases the potential risk to the patient (1) the ICF(s) must be revised and submitted to the IEC(s)/IRB(s) for review and approval/favourable opinion; (2) the revised ICF(s) must be used to obtain consent from patients currently enrolled in the study if they are affected by the amendment; and (3) the new ICF(s) must be used to obtain contact from new patients prior to enrolment.

If the revision is an administrative letter, Investigators must inform their IEC(s)/IRB(s).

18.1.2 Protocol Violations and Deviations

All patients who are enrolled into the study, irrespective of whether or not they receive any treatment, will be followed according to the protocol regardless of the number of treatments received, unless consent for follow-up is withdrawn. Protocol deviations that do not result in harm to the study patients or significantly affect the scientific value of the reported results of the study will be recorded. In case of an important protocol deviation occurring (i.e. a deviation that could have a significant effect on the patient's safety, rights, welfare and/or on the integrity of the study data), the Investigator must notify the Sponsor and the appropriate IEC/IRB as soon as possible or as per local requirements. Major protocol deviations that meet the criteria for a serious breach of GCP (e.g., a protocol violation, or nonreporting of critical safety information potentially jeopardising patient safety) should be reported within 24 hours to the Sponsor. The Sponsor is required to report a serious GCP breach within 7 days to the applicable Health Authorities. Protocol deviations will be recorded on the source documents with an explanation for the deviation. No deviation from the inclusion/exclusion criteria will be permitted.

18.1.3 Monitoring

Before the trial can be initiated at a site, the prerequisites for conducting the trial must be clarified and approved by the Sponsor.

Representatives of the Sponsor (or designee) must be allowed to visit all study site locations periodically to assess the data quality and study integrity according to EU directives, ICH GCP, and the Food and Drug Administration (FDA). On-site they will review study records and directly compare them with source documents, discuss the conduct of the study with the Investigator and verify that the facilities remain acceptable.

Electronic Case Report Form completion and accuracy will be checked by performing source data verification that is a direct comparison of the entries made against the appropriate source documentation. Any resulting discrepancies will be reviewed with the Investigator(s) and /or his/her site staff and resolved. Monitoring procedures require that patients' informed consents, adherence to the inclusion/exclusion criteria and SAE documentation be verified. Additional monitoring activities maybe outlined in the study specific monitoring plan.

The Sponsor must be informed immediately of any change in the personnel involved in the conduct of the trial.

18.1.4 Audits and Site Inspections

Authorised personnel from domestic and foreign regulatory authorities and the Sponsor Quality Assurance (or designee) may carry out inspections and audits respectively. The purpose of an audit/inspection is to ensure that ethical, regulatory and quality requirements are fulfilled in the Sponsor studies.

The Investigator will permit international, national, and local health authorities, the Sponsor monitors, representatives, and collaborators, and the IECs/IRBs to inspect facilities and records relevant to this study. The Investigator should promptly notify the Sponsor or their authorised representative of any inspections scheduled by any regulatory authorities and promptly forward copies of any audit reports received to the Sponsor or their authorised representative.

18.2 DATA HANDLING AND RECORD KEEPING

18.2.1 Data Management

Data for each patient will be entered into the (Sponsor-approved) clinical database via electronic data capture [EDC]) in a timely manner. The EDC application uses system controls to ensure that unauthorised users cannot access or modify data. All users must have successfully undergone EDC application training prior to entering data into the EDC system. Electronic Case Report Forms should be reviewed and electronically signed and dated by the Investigator or a designee. The eCRF system will be compliant with FDA Code of Federal Regulations 21 Part 11 and EU Clinical Trial Directive (EC) No. 2001/20/EC.

It is the responsibility of the Investigator to ensure that the data included in the eCRF is accurate, complete, and electronically signed where appropriate.

The data will be electronically verified through use of on-line checks during data entry and through programmed edit checks specified by the clinical team. Discrepancies in the data will be brought to the attention of the investigational site personnel. Data entered into the eCRF will be validated as defined in the data validation specifications. All updates to queried data will be made by authorised study site personnel only, and all modifications to the database will be recorded in an audit trail. Once all the queries have been resolved, eCRFs will be locked by password protection. Any changes to locked eCRFs will be approved by the Investigator. Once the full set of eCRFs have been completed and locked, the Sponsor will authorise a database

lock and all electronic data will be sent to the designated statistician for analysis. Subsequent changes to the database will then only be made with written agreement of the Sponsor.

Adverse events and medical/cancer history terms will be coded from the verbatim description (Investigator term) using the Medical Dictionary for Regulatory Activities. Prior and concomitant medications and therapies will be coded according to the World Health Organisation drug dictionary. Coding review will be performed by the Sponsor (or designee) prior to database lock.

At the end of the study, the clinical data will be transferred to the Sponsor and the investigative site will receive patient data for their site in a readable format that must be kept with the study records.

18.2.2 Study Documentation and Retention of Records

The Investigator must maintain records of all study documents and supporting information relating to the conduct of the study. This documentation should include but is not limited to, protocols, eCRF data, SAE reports, patient source data, correspondence with health authorities and IEC/IRBs, ICFs, Investigator(s) and study team members' curricula vitae, monitor visit logs, laboratory reference ranges, laboratory certification or quality control procedures and laboratory directors' curricula vitae (essential documentation listed in ICH GCP [CPMP/ICH/135/95]). Patient files and other source data must be kept for the maximum period of time required by applicable regulations and guidelines or institution procedures or for the period specified by the Sponsor, whichever is longer. The Sponsor must be consulted if the Investigator(s) wishes to assign the study files to someone else, remove them to another location or is unable to retain them for the specified period.

The patient medical records must contain the product name/code, the trial reference code, trial patient code and administration dates and dose in order to ensure that a link can be made back to the identity of the product and further traceability records of the Investigator and Sponsor.

18.3 ClINICAL TRIAL AGREEMENT, FINANCE, AND INSURANCE

This study will be conducted under a Clinical Trial Agreement between Autolus Limited ("Sponsor") and the institution(s) representing the investigational study site(s) ("Investigator"). Financial support to the investigational site(s) will be detailed in the Clinical Trial Agreement. The Clinical Trial Agreement must be signed before the commencement of the study and will clearly delineate the responsibilities and obligations of the Investigator and the Sponsor, and will form the contractual basis under which the clinical study will be conducted.

A Certificate of Clinical Trials Insurance will be provided to the study centres by the Sponsor, where required. Details of the Sponsor's arrangement for clinical study insurance to provide for compensation to patients for any claim for bodily injury or death arising from participation in the clinical study are provided in the PIS Part A and Part B.

19 PUBLICATION OF DATA AND PROTECTION OF TRADE SECRETS

Both the EU Data Protection Regulations and the FDA Amendments Act mandates the registration with ClinicalTrials.gov of certain clinical trials of drugs (including biological products) and medical devices subject to FDA regulations for any disease or condition. The International Committee of Medical Journal Editors requires study registration as a condition for publication of research results generated by a clinical study (http://www.icmje.org).

All information supplied by the Sponsor in connection with this study and not previously published to the public, is considered confidential information ("Confidential Information"). This confidential information includes, but is not limited to, the IB, clinical protocol, eCRFs and other scientific data. Any data collected during the study are also considered confidential information. This confidential information shall remain the sole and exclusive property of the Sponsor, shall not be disclosed to others without prior written consent of the Sponsor, and shall not be used except in the performance of this study.

The information developed during the conduct of this study is also considered confidential information, and will be used by the Sponsor in connection with the development of the ATIMP. The confidential information may be disclosed as deemed necessary by the Sponsor. To allow the use of the confidential information derived from this study, the Investigator is obliged to provide the Sponsor with complete test results and all data developed in this study.

The Sponsor has full ownership of the original eCRFs completed as part of the study.

By signing the Clinical Study Protocol, the Investigator agrees that the results of the study may be used for the purposes of national and international registration, publication, and information for medical and pharmaceutical professionals. The authorities will be notified of the Investigator's name, address, qualifications, and extent of involvement.

It is agreed that, consistent with scientific standards, publication of the results of the study shall be made only as part of a publication of the results obtained by all sites performing the protocol.

Should the Investigator wish to publish the results of this study, the Investigator agrees to provide the Sponsor with a manuscript for review 60 days prior to submission for publication.

The Sponsor retains the right to delete from the manuscript, information that is confidential or proprietary and to object suggested publication and/or its timing (at the Sponsor's discretion).

In addition, if requested by the Sponsor, the Investigator shall withhold publication an additional 6 months to allow for filing a patent application or taking such other measures as the Sponsor deems appropriate to establish and preserve its proprietary rights.

In the event that the Sponsor chooses to publish the data from this study, a copy will be provided to the Investigator at least 30 days prior to the expected date of submission to the intended publisher (Fitzgerald et al. 2016).

20 REFERENCES

Abouyabis AN, Shenoy PJ, Sinha R, Flowers CR and Lechowicz MJ (2011). A Systematic Review and Meta-Analysis of Front-line Anthracycline-Based Chemotherapy Regimens for Peripheral T-Cell Lymphoma. ISRN Hematol **2011**:623924.

Actemra Prescribing Information. (2018). from https://www.accessdata.fda.gov/drugsatfda_docs/label/2018/125276s117,125472s032lbl.pdf.

Ali SA, Shi V, Maric I, Wang M, Stroncek DF, Rose JJ, et al. (2016). T cells expressing an anti-B-cell-maturation-antigen chimeric antigen receptor cause remissions of multiple myeloma. Blood **128**(13):1688-1700.

Ali SA, Shi V, Maric I, Wang M, Stroncek DF, Rose JJ, et al. (2016). T cells expressing an anti-B-cell maturation antigen chimeric antigen receptor cause remissions of multiple myeloma. Blood **128**(13):1688-1700.

Anderson JR, Armitage JO and Weisenburger DD (1998). Epidemiology of the non-Hodgkin's lymphomas: distributions of the major subtypes differ by geographic locations. Non-Hodgkin's Lymphoma Classification Project. Ann Oncol **9**(7):717-720.

Ascani S, Zinzani PL, Gherlinzoni F, Sabattini E, Briskomatis A, de Vivo A, et al. (1997). Peripheral T-cell lymphomas. Clinico-pathologic study of 168 cases diagnosed according to the R.E.A.L. Classification. Ann Oncol **8**(6):583-592.

Bellei M, Chiattone CS, Luminari S, Pesce EA, Cabrera ME, de Souza CA, et al. (2012). T-Cell Lymphomas in South America and Europe. Revista Brasileira de Hematologia e Hemoterapia **34**(1):42-47.

Bhojwani D, Sabin ND, Pei D, Yang JJ, Khan RB, Panetta JC, et al. (2014). Methotrexate-induced neurotoxicity and leukoencephalopathy in childhood acute lymphoblastic leukemia. J Clin Oncol **32**(9):949-959.

Brentjens RJ, Latouche JB, Santos E, Marti F, Gong MC, Lyddane C, et al. (2003). Eradication of systemic B-cell tumors by genetically targeted human T lymphocytes co-stimulated by CD80 and interleukin-15. Nat Med **9**(3):279-286.

Brentjens RJ, Rivière I, Park JH, Davila ML, Wang X, Stefanski J, et al. (2011). Safety and persistence of adoptively transferred autologous CD19-targeted T cells in patients with relapsed or chemotherapy refractory B-cell leukemias. Blood **118**(18):4817-4828.

Brudno JN and Kochenderfer JN (2016). Toxicities of chimeric antigen receptor T cells: recognition and management. Blood **127**(26):3321-3330.

Brudno JN, Somerville RP, Shi V, Rose JJ, Halverson DC, Fowler DH, et al. (2016). Allogeneic T Cells That Express an Anti-CD19 Chimeric Antigen Receptor Induce Remissions of B-Cell Malignancies That Progress After Allogeneic Hematopoietic Stem-Cell Transplantation Without Causing Graft-Versus-Host Disease. J Clin Oncol **34**(10):1112-1121.

Calabrese LH and Rose-John S (2014). IL-6 biology: implications for clinical targeting in rheumatic disease. Nat Rev Rheumatol **10**(12):720-727.

Chen KH, Wada M, Firor AE, Pinz KG, Jares A, Liu H, et al. (2016). Novel anti-CD3 chimeric antigen receptor targeting of aggressive T cell malignancies. Oncotarget **5**(0).

Cheson BD, Fisher RI, Barrington SF, Cavalli F, Schwartz LH, Zucca E, et al. (2014). Recommendations for initial evaluation, staging, and response assessment of Hodgkin and non-Hodgkin lymphoma: the Lugano classification. J Clin Oncol **32**(27):3059-3068.

Chu CS, Boyer J, Schullery DS, Gimotty PA, Gamerman V, Bender J, et al. (2012). Phase I/II randomized trial of dendritic cell vaccination with or without cyclophosphamide for consolidation therapy of advanced ovarian cancer in first or second remission. Cancer Immunol. Immunother. **61**(5):629-641.

Coiffier B, Pro B, Prince HM, Foss F, Sokol L, Greenwood M, et al. (2012). Results from a pivotal, open-label, phase II study of romidepsin in relapsed or refractory peripheral T-cell lymphoma after prior systemic therapy. J Clin Oncol **30**(6):631-636.

Cooper LJN, Topp MS, Serrano LM, Gonzalez S, Chang W-C, Naranjo A, et al. (2003). T-cell clones can be rendered specific for CD19: toward the selective augmentation of the graft-versus-B-lineage leukemia effect. Blood **101**(4):1637-1644.

Curti BD, Ochoa AC, Powers GC, Kopp WC, Alvord WG, Janik JE, et al. (1998). Phase I trial of anti-CD3-stimulated CD4+ T cells, infusional interleukin-2, and cyclophosphamide in patients with advanced cancer. J. Clin. Oncol **16**(8):2752-2760.

Davila ML, Riviere I, Wang X, Bartido S, Park J, Curran K, et al. (2014). Efficacy and toxicity management of 19-28z CAR T cell therapy in B cell acute lymphoblastic leukemia. Sci Transl Med **6**(224):224ra225.

Dreyling M, Thieblemont C, Gallamini A, Arcaini L, Campo E, Hermine O, et al. (2013). ESMO Consensus conferences: guidelines on malignant lymphoma. part 2: marginal zone lymphoma, mantle cell lymphoma, peripheral T-cell lymphoma. Ann Oncol **24**(4):857-877.

Dudley ME, Yang JC, Sherry R, Hughes MS, Royal R, Kammula U, et al. (2008). Adoptive cell therapy for patients with metastatic melanoma: evaluation of intensive myeloablative chemoradiation preparative regimens. J. Clin. Oncol **26**(32):5233-5239.

Fitzgerald JC, Weiss SL, Maude SL, Barrett DM, Lacey SF, Melenhorst JJ, et al. (2016). Cytokine Release Syndrome After Chimeric Antigen Receptor T Cell Therapy for Acute Lymphoblastic Leukemia. Crit. Care Med.

Flammiger A, Fiedler W, Bacher U, Bokemeyer C, Schneider M and Binder M (2012). Critical imbalance of TNF-alpha and soluble TNF receptor 1 in a patient with macrophage activation syndrome: potential implications for diagnostics and treatment. Acta Haematol **128**(2):69-72.

Frisen L (1982). Swelling of the optic nerve head: a staging scheme. J Neurol Neurosurg Psychiatry **45**(1):13-18.

Fry EE (2016). CD22 CAR Update and Novel Mechanisms of Leukemic Resistance. Clin Cancer Res, New Orleans, Louisiana.

Gabay C, Lamacchia C and Palmer G (2010). IL-1 pathways in inflammation and human diseases. Nat Rev Rheumatol **6**(4):232-241.

Garfall AL, Maus MV, Hwang WT, Lacey SF, Mahnke YD, Melenhorst JJ, et al. (2015). Chimeric Antigen Receptor T Cells against CD19 for Multiple Myeloma. N. Engl. J. Med **373**(11):1040-1047.

Geller MA, Cooley S, Judson PL, Ghebre R, Carson LF, Argenta PA, et al. (2011). A phase II study of allogeneic natural killer cell therapy to treat patients with recurrent ovarian and breast cancer. Cytotherapy **13**(1):98-107.

Gilbert MJ (2017). Severe neurotoxicity in the Phase 2 trial of JCAR015 in adult B-ALL (ROCKET study): Analyses of patient, protocol and product attributes. Proceedings from the Society for Immunology of Cancer 8:12.

Goebeler ME and Bargou R (2016). Blinatumomab: a CD19/CD3 bispecific T cell engager (BiTE) with unique anti-tumor efficacy. Leukemia & lymphoma **57**(5):1021-1032.

Grupp SA, Kalos M, Barrett D, Aplenc R, Porter DL, Rheingold SR, et al. (2013). Chimeric antigen receptor-modified T cells for acute lymphoid leukemia. N Engl J Med **368**(16):1509-1518.

Gust J, Hay KA, Hanafi LA, Li D, Myerson D, Gonzalez-Cuyar LF, et al. (2017). Endothelial Activation and Blood-Brain Barrier Disruption in Neurotoxicity after Adoptive Immunotherapy with CD19 CAR-T Cells. Cancer Discov **7**(12):1404-1419.

Hallek M (2013). Chronic lymphocytic leukemia: 2013 update on diagnosis, risk stratification and treatment. Am. J. Hematol. **88**(9):803-816.

Hay KA, Hanafi LA, Li D, Gust J, Liles WC, Wurfel MM, et al. (2017). Kinetics and biomarkers of severe cytokine release syndrome after CD19 chimeric antigen receptor-modified T-cell therapy. Blood **130**(21):2295-2306.

Hay KA and Turtle CJ (2017). Chimeric Antigen Receptor (CAR) T cells: Lessons Learned from Targeting of CD19 in B cell malignancies. Drugs. 2017 Mar; 77(3): 237–245.

Helton KJ, Patay Z and Triplett BM (2013). Fludarabine-induced severe necrotizing leukoencephalopathy in pediatric hematopoietic cell transplantation. Bone Marrow Transplant **48**(5):729-731.

Hoyos V, Savoldo B, Quintarelli C, Mahendravada A, Zhang M, Vera J, et al. (2010). Engineering CD19-specific T lymphocytes with interleukin-15 and a suicide gene to enhance their anti-lymphoma/leukemia effects and safety. Leukemia **24**(6):1160-1170.

Inaba H, Khan RB, Laningham FH, Crews KR, Pui CH and Daw NC (2008). Clinical and radiological characteristics of methotrexate-induced acute encephalopathy in pediatric patients with cancer. Ann Oncol **19**(1):178-184.

Jensen MC, Popplewell L, Cooper LJ, DiGiusto D, Kalos M, Ostberg JR, et al. (2010). Antitransgene rejection responses contribute to attenuated persistence of adoptively transferred CD20/CD19-specific chimeric antigen receptor redirected T cells in humans. Biol Blood Marrow Transplant **16**(9):1245-1256.

Jordan MB, Allen CE, Weitzman S, Filipovich AH and McClain KL (2011). How I treat hemophagocytic lymphohistiocytosis. Blood **118**(15):4041-4052.

Karschnia P, Jordan JT, Forst DA, Arrillaga-Romany IC, Batchelor TT, Baehring JM, et al. (2019). Clinical presentation, management, and biomarkers of neurotoxicity after adoptive immunotherapy with CAR T-cells. Blood:blood-2018-2012-893396.

Klebanoff CA, Khong HT, Antony PA, Palmer DC and Restifo NP (2005). Sinks, suppressors and antigen presenters: how lymphodepletion enhances T cell-mediated tumor immunotherapy. Trends Immunol. **26**(2):111-117.

Klinger M, Benjamin J, Kischel R, Stienen S and Zugmaier G (2016). Harnessing T cells to fight cancer with BiTE® antibody constructs – past developments and future directions. Immunological Reviews **270**(1):193-208.

Kochenderfer J, Somerville R, Lu T, Shi V, Yang JC, Sherry R, et al. (2016). Anti-CD19 chimeric antigen receptor T cells preceded by low-dose chemotherapy to induce remissions of advanced lymphoma. J. Clin. Oncol. **34**(suppl; abstr LBA3010).

Kochenderfer JN, Dudley ME, Feldman SA, Wilson WH, Spaner DE, Maric I, et al. (2012). B-cell depletion and remissions of malignancy along with cytokine-associated toxicity in a clinical trial of anti-CD19 chimeric-antigen-receptor-transduced T cells. Blood **119**(12):2709-2720.

Kochenderfer JN, Dudley ME, Kassim SH, Somerville RP, Carpenter RO, Stetler-Stevenson M, et al. (2015). Chemotherapy-refractory diffuse large B-cell lymphoma and indolent B-cell malignancies can be effectively treated with autologous T cells expressing an anti-CD19 chimeric antigen receptor. J Clin Oncol **33**(6):540-549.

Kochenderfer JN, Yu Z, Frasheri D, Restifo NP and Rosenberg SA (2010). Adoptive transfer of syngeneic T cells transduced with a chimeric antigen receptor that recognizes murine CD19 can eradicate lymphoma and normal B cells. Blood **116**(19):3875-3886.

Kymriah (tisagenlecleucel) EU SmPC. (2018). Retrieved 17 May 2019, from https://www.ema.europa.eu/en/documents/product-information/kymriah-epar-product-information_en.pdf.

Kymriah US Prescribing Information. (2018). from https://www.fda.gov/media/107296/download.

Laurent J, Speiser DE, Appay V, Touvrey C, Vicari M, Papaioannou A, et al. (2010). Impact of 3 different short-term chemotherapy regimens on lymphocyte-depletion and reconstitution in melanoma patients. J. Immunother. **33**(7):723-734.

Lee DW, Gardner R, Porter DL, Louis CU, Ahmed N, Jensen M, et al. (2014). Current concepts in the diagnosis and management of cytokine release syndrome. Blood **124**(2):188-195.

Lee DW, Kochenderfer JN, Stetler-Stevenson M, Cui YK, Delbrook C, Feldman SA, et al. (2015). T cells expressing CD19 chimeric antigen receptors for acute lymphoblastic leukaemia in children and young adults: a phase 1 dose-escalation trial. Lancet **385**(9967):517-528.

Lee DW, Santomasso BD, Locke FL, Ghobadi A, Turtle CJ, Brudno JN, et al. (2019). ASTCT Consensus Grading for Cytokine Release Syndrome and Neurologic Toxicity Associated with Immune Effector Cells. Biol Blood Marrow Transplant **25**(4):625-638.

Lee DW, Stetler-Stevenson M, Yuan CM, Shah NN, Delbrook C, Yates B, et al. (2016). Long-Term Outcomes Following CD19 CAR T Cell Therapy for B-ALL Are Superior in Patients Receiving a Fludarabine/Cyclophosphamide Preparative Regimen and Post-CAR Hematopoietic Stem Cell Transplantation. Blood **128**(22):218-218.

Liu D and Zhao J (2018). Cytokine release syndrome: grading, modeling, and new therapy. J Hematol Oncol **11**(1):121.

Locke FL, Neelapu SS, Bartlett NL, Lekakis LJ, Jacobson CA, Braunschweig I, et al. (2017). Preliminary Results of Prophylactic Tocilizumab after Axicabtageneciloleucel (axi-cel; KTE-

C19) Treatment for Patients with Refractory,Aggressive Non-Hodgkin Lymphoma (NHL). Blood **130**(Suppl 1):1547-1547.

Louis CU, Savoldo B, Dotti G, Pule M, Yvon E, Myers GD, et al. (2011). Antitumor activity and long-term fate of chimeric antigen receptor-positive T cells in patients with neuroblastoma. Blood **118**(23):6050-6056.

Maciocia PM, Wawrzyniecka P, Philip B, Ricciardelli I, Akarca AU, Onuoha S, et al. (2016). Targeting T-Cell Receptor β-Constant Domain for Immunotherapy of T-Cell Malignancies. ASH 58th Annual Meeting & Exposition. San Diego, American Society of Hematology.

Mak V, Hamm J, Chhanabhai M, Shenkier T, Klasa R, Sehn LH, et al. (2013). Survival of patients with peripheral T-cell lymphoma after first relapse or progression: spectrum of disease and rare long-term survivors. J Clin Oncol **31**(16):1970-1976.

Mamonkin M, Rouce RH, Tashiro H and Brenner MK (2015). A T-cell–directed chimeric antigen receptor for the selective treatment of T-cell malignancies. Blood **126**(8):983-992.

Maude SL, Frey N, Shaw PA, Aplenc R, Barrett DM, Bunin NJ, et al. (2014). Chimeric antigen receptor T cells for sustained remissions in leukemia. N Engl J Med **371**(16):1507-1517.

Maude SL, Teachey DT, Porter DL and Grupp SA (2015). CD19-targeted chimeric antigen receptor T-cell therapy for acute lymphoblastic leukemia. Blood **125**(26):4017-4023.

Mitchell CD, Richards SM, Kinsey SE, Lilleyman J, Vora A and Eden TO (2005). Benefit of dexamethasone compared with prednisolone for childhood acute lymphoblastic leukaemia: results of the UK Medical Research Council ALL97 randomized trial. Br J Haematol **129**(6):734-745.

Morbach H, Eichhorn EM, Liese JG and Girschick HJ (2010). Reference values for B cell subpopulations from infancy to adulthood. Clin Exp Immunol **162**(2):271-279.

Mueller KT, Waldron E, Grupp SA, Levine JE, Laetsch TW, Pulsipher MA, et al. (2018). Clinical Pharmacology of Tisagenlecleucel in B-cell Acute Lymphoblastic Leukemia. Clin Cancer Res **24**(24):6175-6184.

Neelapu SS, Locke FL, Bartlett NL, Siddiqi T, Chavez JC, Hosing C, et al. (2016). Ongoing complete remissions (CR) in the phase 1 of ZUMA-1: a phase 1-2 multicenter study evaluating the safety and efficacy of KTE-C19 (anti-CD19 CAR T cells) in subjects with refractory aggressive B-cell Non-Hodgkin Lymphoma (NHL). J. Clin. Oncol. **34**(suppl; abstr 7559).

Neelapu SS, Locke FL, Bartlett NL, Siddiqi T, Chavez JC, Hosing C, et al. (2016a). Ongoing complete remissions (CR) in the phase 1 of ZUMA-1: a phase 1-2 multicenter study evaluating the safety and efficacy of KTE-C19 (anti-CD19 CAR T cells) in subjects with refractory aggressive B-cell Non-Hodgkin Lymphoma (NHL). J. Clin. Oncol. **34**(suppl; abstr 7559).

Neelapu SS, Tummala S, Kebriaei P, Wierda W, Gutierrez C, Locke FL, et al. (2018). Chimeric antigen receptor T-cell therapy - assessment and management of toxicities. Nat Rev Clin Oncol **15**(1):47-62.

NICE (2016). NG52: Non-Hodgkin’s lymphoma: diagnosis and management**:** 25.

O'Connor OA, Horwitz S, Masszi T, Van Hoof A, Brown P, Doorduijn J, et al. (2015). Belinostat in Patients With Relapsed or Refractory Peripheral T-Cell Lymphoma: Results of the Pivotal Phase II BELIEF (CLN-19) Study. J. Clin. Oncol. **33**(23):2492-2499.

O'Connor OA, Pro B, Pinter-Brown L, Bartlett N, Popplewell L, Coiffier B, et al. (2011). Pralatrexate in patients with relapsed or refractory peripheral T-cell lymphoma: results from the pivotal PROPEL study. J Clin Oncol **29**(9):1182-1189.

Oken MM, Creech RH, Tormey DC, Horton J, Davis TE, McFadden ET, et al. (1982). Toxicity and response criteria of the Eastern Cooperative Oncology Group. Am J Clin Oncol **5**(6):649-655.

Pinz K, Liu H, Golightly M, Jares A, Lan F, Zieve GW, et al. (2016). Preclinical targeting of human T-cell malignancies using CD4-specific chimeric antigen receptor (CAR)-engineered T cells. Leukemia **30**(3):701-707.

Porter DL, Levine BL, Kalos M, Bagg A and June CH (2011). Chimeric antigen receptor-modified T cells in chronic lymphoid leukemia. N. Engl. J. Med **365**(8):725-733.

Prahalad S, Bove KE, Dickens D, Lovell DJ and Grom AA (2001). Etanercept in the treatment of macrophage activation syndrome. J Rheumatol **28**(9):2120-2124.

Pro B, Advani R, Brice P, Bartlett NL, Rosenblatt JD, Illidge T, et al. (2012). Brentuximab vedotin (SGN-35) in patients with relapsed or refractory systemic anaplastic large-cell lymphoma: results of a phase II study. J Clin Oncol **30**(18):2190-2196.

Ramakrishnan A et al. Phase 1 Alexander Study of AUTO3, the First CD19/22 Dual Targeting CAR T Cell Therapy, with Pembrolizumab in Patients with Relapsed/Refractory (r/r) DLBCL. Abstract 600, ASH 2020.

RoActemra SmPC. (2018). from https://www.ema.europa.eu/en/documents/product-information/roactemra-epar-product-information_en.pdf.

Ruella M, Kenderian SS, Shestova O, Klichinsky M, Melenhorst JJ, Wasik MA, et al. (2017). Kinase inhibitor ibrutinib to prevent cytokine-release syndrome after anti-CD19 chimeric antigen receptor T cells for B-cell neoplasms. Leukemia **31**(1):246-248.

Russell JA, Walley KR, Singer J, Gordon AC, Hebert PC, Cooper DJ, et al. (2008). Vasopressin versus norepinephrine infusion in patients with septic shock. N Engl J Med **358**(9):877-887.

Santomasso BD, Park JH, Salloum D, Riviere I, Flynn J, Mead E, et al. (2018). Clinical and Biological Correlates of Neurotoxicity Associated with CAR T-cell Therapy in Patients with B-cell Acute Lymphoblastic Leukemia. Cancer Discov **8**(8):958-971.

Sathaliyawala T, Kubota M, Yudanin N, Turner D, Camp P, Thome JJ, et al. (2013). Distribution and compartmentalization of human circulating and tissue-resident memory T cell subsets. Immunity **38**(1):187-197.

Savoldo B, Ramos CA, Liu E, Mims MP, Keating MJ, Carrum G, et al. (2011). CD28 costimulation improves expansion and persistence of chimeric antigen receptor-modified T cells in lymphoma patients. J Clin Invest **121**(5):1822-1826.

Schram AM and Berliner N (2015). How I treat hemophagocytic lymphohistiocytosis in the adult patient. Blood **125**(19):2908-2914.

Schuster SJ, Svoboda J, Nasta SD, Chong EA, Winchell N, Landsburg DJ, et al. (2016). Treatment with Chimeric Antigen Receptor Modified T Cells Directed Against CD19

(CTL019) Results in Durable Remissions in Patients with Relapsed or Refractory Diffuse Large B Cell Lymphomas of Germinal Center and Non-Germinal Center Origin, "Double Hit" Diffuse Large B Cell Lymphomas, and Transformed Follicular to Diffuse Large B Cell Lymphomas. 58th Annual Meeting and Exposition. San Diego, CA, USA, American Society of Hematology.

Shah NN, Shalabi H, Yates B, Kane E, Dulau-Florea A, Cullinane A, et al. (2017). Beyond Cytokine Storm: Optimizing Treatment Strategies to Target the Complex Interplay between CAR Mediated Inflammatory Response, Disseminated Intravascular Coagulation and Macrophage Activation Syndrome. Blood **130**(Suppl 1):1277.

Siddiqi T, Abramson JS, Li D, Brown W, Devries T, Dave K, et al. (2017). Patient Characteristics and Pre-Infusion Biomarkers of Inflammation Correlate with Clinical Outcomes after Treatment with the Defined Composition, CD19-Targeted CAR T Cell Product, JCAR017. Blood **130(**Suppl 1):193-193.

Siltuximab US Prescribing Information. (2019). from https://www.accessdata.fda.gov/drugsatfda_docs/label/2014/125496s000lbl.pdf.

Simon R (1989). Optimal two-stage designs for phase II clinical trials. Control Clin Trials **10**(1):1-10.

Skamene T, Crump M, Savage KJ, Reiman T, Kuruvilla J, Good D, et al. (2017). Salvage chemotherapy and autologous stem cell transplantation for peripheral T-cell lymphoma: a subset analysis of the Canadian Cancer Trials Group LY.12 randomized phase 3 study. Leuk Lymphoma **58**(10):2319-2327.

Skolnik JM, Barrett JS, Jayaraman B, Patel D and Adamson PC (2008). Shortening the timeline of pediatric phase I trials: the rolling six design. J Clin Oncol **26**(2):190-195.

Smith SW (2013). The Role of Chelation in the Treatment of Other Metal Poisonings. J. Med. Toxicol. **9**(4):355-369.

Sporn JR, Ergin MT, Robbins GR, Cable RG, Silver H and Mukherji B (1993). Adoptive immunotherapy with peripheral blood lymphocytes cocultured in vitro with autologous tumor cells and interleukin-2. Cancer Immunol. Immunother. **37**(3):175-180.

Tamamyan GN, Kantarjian HM, Ning J, Jain P, Sasaki K, McClain KL, et al. (2016). Malignancy-associated hemophagocytic lymphohistiocytosis in adults: Relation to hemophagocytosis, characteristics, and outcomes. Cancer **122**(18):2857-2866.

Teachey DT, Bishop MR, Maloney DG and Grupp SA (2018). Toxicity management after chimeric antigen receptor T cell therapy: one size does not fit 'ALL'. Nat Rev Clin Oncol **15**(4):218.

Turtle CJ, Hanafi L-A, Berger C, Gooley TA, Cherian S, Hudecek M, et al. (2016). CD19 CAR–T cells of defined CD4+:CD8+ composition in adult B cell ALL patients. J Clin Invest **126**(6):2123-2138.

Turtle CJ, Hanafi LA, Berger C, Gooley TA, Cherian S, Hudecek M, et al. (2016). CD19 CAR-T cells of defined CD4+:CD8+ composition in adult B cell ALL patients. J Clin Invest **126**(6):2123-2138.

Wrzesinski C and Restifo NP (2005). Less is more: lymphodepletion followed by hematopoietic stem cell transplant augments adoptive T-cell-based anti-tumor immunotherapy. Curr. Opin. Immunol. **17**(2):195-201.

Zhang et al (2020). First-in-Class Autologous CD7-CAR-T Cells Exhibited Promising Clinical Efficacy for Relapsed and Refractory T-Lymphoblastic Leukemia/Lymphoma. Abstract 973 American Society of Hematology (ASH) 2020

21 APPENDICES

Appendix 1: Patients with T-NHL will be Evaluated Using Response Criteria for Non-Hodgkin Lymphoma for Documenting Disease Response

Lugano Classification (Cheson et al. 2014)

Response	Site	PET-CT-Based Response	CT-Based Response
**Complete**		**Complete metabolic response**	**Complete radiologic response (all of the following)**
	Lymph nodes and extralymphatic sites	Score 1, 2, or 3[a] with or without a residual mass on 5PS[b]<br>It is recognised that in Waldeyer's ring or extranodal sites with high physiologic uptake or with activation within spleen or marrow (e.g., with chemotherapy or myeloid colony-stimulating factors), uptake may be greater than normal mediastinum and/or liver. In this circumstance, complete metabolic response may be inferred if uptake at sites of initial involvement is no greater than surrounding normal tissue even if the tissue has high physiologic uptake	Target nodes/nodal masses must regress to ≤1.5 cm in the longest transverse diameter of the lesion (LDi).<br>No extralymphatic sites of disease
	Non-measured lesion	Not applicable	Absent
	Organ enlargement	Not applicable	Regress to normal
	New lesions	None	None
	Bone marrow	No evidence of FDG-avid disease in marrow	Normal by morphology; if indeterminate, IHC negative
**Partial**		**Partial metabolic response**	**Partial remission (all of the following)**
	Lymph nodes and extra-lymphatic sites	Score 4 or 5[b] with reduced uptake compared with baseline and residual mass(es) of any size<br>At interim, these findings suggest responding disease<br>At end of treatment, these findings indicate residual disease	≥50% decrease in sum of the product of the perpendicular diameters for multiple lesions (SPD) of up to 6 target measurable nodes and extranodal sites<br>When a lesion is too small to measure on CT, assign 5 mm × 5 mm as the default value<br>When no longer visible, 0 × 0 mm<br>For a node >5 mm × 5 mm, but smaller than normal, use actual measurement for calculation
	Non-measured lesion	Not applicable	Absent/normal, regressed, but no increase

Response	Site	PET-CT-Based Response	CT-Based Response
	Organ enlargement	Not applicable	Spleen must have regressed by >50% in length beyond normal
	New lesions	None	None
	Bone Marrow	Residual uptake higher than uptake in normal marrow but reduced compared with baseline (diffuse uptake compatible with reactive changes from chemotherapy allowed). If there are persistent focal changes in the marrow in the context of a nodal response, consideration should be given to further evaluation with MRI or biopsy or an interval scan	Not applicable
**No response or stable disease**		**No metabolic response**	**Stable disease**
	Lymph nodes and extralymphatic sites	Score 4 or 5 with no significant change in FDG uptake from baseline at interim or end of treatment	<50% decrease from baseline in SPD of up to 6 dominant, measurable nodes and extranodal sites; no criteria for progressive disease are met.
	Non-measured lesion	Not applicable	No increase consistent with progression
	Organ enlargement	Not applicable	No increase consistent with progression
	New lesions	None	None
	Bone marrow	No change from baseline	Not applicable
**Progressive disease**		**Progressive metabolic disease**	**Progressive disease requires at least 1 of the following:**
	Individual target nodes/nodal masses	Score 4 or 5 with an increase in intensity of uptake from baseline and/or	Cross product of the LDi and perpendicular diameter (PPD) progression:

Response	Site	PET-CT-Based Response	CT-Based Response
	Extranodal lesions	New FDG-avid foci consistent with lymphoma at interim or end-of-treatment assessment	An individual node/lesion must be abnormal with:<br>LDi >1.5 cm, and<br>increase by ≥50% from PPD nadir, and<br>an increase in LDi or SDi from nadir:<br>0.5 cm for lesions ≤2 cm<br>1.0 cm for lesions >2 cm<br>In the setting of splenomegaly, the splenic length must increase by >50% of the extent of its prior increase beyond baseline (e.g., a 15-cm spleen must increase to >16 cm). If no prior splenomegaly, must increase by at least 2 cm from baseline.<br>New or recurrent splenomegaly
	Non-measured lesions	None	New or clear progression of pre-existing non-measured lesions
	New lesions	New FDG-avid foci consistent with lymphoma rather than another aetiology (e.g., infection, inflammation). If uncertain regarding aetiology of new lesions, biopsy or interval scan may be considered	Regrowth of previously resolved lesions<br>A new node >1.5 cm in any axis<br>A new extranodal site >1.0 cm in any axis; if <1.0 cm in any axis, its presence must be unequivocal and must be attributable to lymphoma<br>Assessable disease of any size unequivocally attributable to lymphoma
	Bone marrow	New or recurrent FDG-avid foci	New or recurrent involvement

5PS = 5-point scale; CT = computed tomography; FDG = fluorodeoxyglucose; IHC = immunohistochemistry; LDi = longest transverse diameter of a lesion; MRI = magnetic resonance imaging; PET = positron emission tomography; PPD = cross product of the LDi and perpendicular diameter; SDi = shortest axis perpendicular to the LDi; SPD = sum of the product of the perpendicular diameters for multiple lesions.

a A score of 3 in many patients indicates a good prognosis with standard treatment, especially if at the time of an interim scan. However, in trials involving PET where de-escalation is investigated, it may be preferable to consider a score of 3 as inadequate response (to avoid undertreatment). Measured dominant lesions: Up to 6 of the largest dominant nodes, nodal masses, and extranodal lesions selected to be clearly measurable in 2 diameters. Nodes should preferably be from disparate regions of the body and should include, where applicable, mediastinal and retroperitoneal areas. Non-nodal lesions include those in solid organs (e.g., liver, spleen, kidneys, lungs), GI involvement, cutaneous lesions, or those noted on palpation. Non-measured lesions: Any disease not selected as measured, dominant disease and truly assessable disease should be considered not measured. These sites include any nodes, nodal masses, and extranodal sites not selected as dominant or measurable or that do not meet the requirements for measurability, but are still considered abnormal, as well as truly assessable disease, which is any site of suspected disease that would be difficult to follow quantitatively with measurement, including pleural effusions, ascites, bone lesions, leptomeningeal disease, abdominal masses, and other lesions that cannot be confirmed and followed by imaging. In Waldeyer's ring or in extranodal sites (e.g., GI tract, liver, bone marrow), fluorodeoxyglucose uptake may be greater than in the mediastinum with complete metabolic response, but should be no higher than surrounding normal physiologic uptake (e.g., with marrow activation as a result of chemotherapy or myeloid growth factors).

b PET 5PS: 1, no uptake above background; 2, uptake ≤mediastinum; 3, uptake >mediastinum but ≤liver; 4, uptake moderately >liver; 5, uptake markedly higher than liver and/or new lesions; X, new areas of uptake unlikely to be related to lymphoma

Appendix 2: Eastern Cooperative Oncology Group Performance Status Score

Grade	Eastern Cooperative Oncology Group Performance Status
0	Fully active, able to carry on all pre-disease performance without restriction
1	Restricted in physically strenuous activity but ambulatory and able to carry out work of a light or sedentary nature, e.g., light house work, office work
2	Ambulatory and capable of all self-care but unable to carry out any work activities. Up and about more than 50% of waking hours
3	Capable of only limited self-care, confined to bed or chair more than 50% of waking hours
4	Completely disabled. Cannot carry on any self-care. Totally confined to bed or chair
5	Dead

Source: Eastern Cooperative Oncology Group, Robert Comis M.D., Group Chair (Oken et al. 1982).

Appendix 3: Highly Effective and Acceptable Methods of Birth Control

For females of childbearing potential (defined as <2 years after last menstruation or not surgically sterile), a negative serum or urine pregnancy test must be documented at screening, prior to conditioning and confirmed before receiving the first dose of study treatment.

For females who are not postmenopausal (<24 months of amenorrhea) or surgically sterile (absence of ovaries and/or uterus), highly effective methods of contraception must be used from the start of the pre-conditioning stage and for at least 12 months after the last dose of study treatment. They must agree not to donate eggs (ova, oocytes) for the purposes of assisted reproduction during the study and for 12 months after receiving the last dose of study drug

For males, it must be agreed that two acceptable methods of contraception, including a barrier method of contraception are used from the start of the pre-conditioning stage and for at least 12 months after the last dose of AUTO4 (one by the patient – usually a barrier method, and one by the patient's partner) and that sperm will not be donated during the treatment period and for at least 12 months after the last dose of study treatment.

Highly effective methods of birth control:

For the purpose of this guidance, methods that can achieve a failure rate of less than 1% per year when used consistently and correctly are considered as highly effective birth control methods. Such methods include:

- Combined (oestrogen and progestogen containing) hormonal contraception associated with inhibition of ovulation[1]:
  - Oral
  - Intravaginal
  - Transdermal
- Progestogen-only hormonal contraception associated with inhibition of ovulation[1]:
  - Oral
  - Injectable
  - Implantable[2]
- Intrauterine device[2]
- Intrauterine hormone-releasing system[2]
- Bilateral tubal occlusion[2]
- Vasectomised partner[2, 3]
- Sexual abstinence[4]

1 Hormonal contraception may be susceptible to interaction with the AUTO4, which may reduce the efficacy of the contraception method.

2 Contraception methods that in the context of this guidance are considered to have low user dependency.

3 Vasectomised partner is a highly effective birth control method provided that the vasectomised partner is the sole sexual partner of the women of childbearing potential trial participant and that the vasectomised partner has received medical assessment of the surgical success.

4 In the context of this guidance, sexual abstinence is considered a highly effective method only if defined as refraining from heterosexual intercourse during the entire period of risk associated with

the study treatments. The reliability of sexual abstinence needs to be evaluated in relation to the duration of the clinical trial and the preferred and usual lifestyle of the patient.

Birth control methods that may NOT be considered as highly effective

Acceptable birth control methods that result in a failure rate of more than 1% per year include:

- Progestogen-only oral hormonal contraception, where inhibition of ovulation is not the primary mode of action
- Male or female condom with or without spermicide [5]
- Cap, diaphragm or sponge with spermicide [5]

[5] A combination of male condom with either cap, diaphragm or sponge with spermicide (double barrier methods) are also considered acceptable, but NOT highly effective, birth control methods.

Birth control methods that are considered UNACCEPTABLE in clinical trials

- Periodic abstinence (calendar, symptothermal, post-ovulation methods)
- Withdrawal (coitus interruptus)
- Spermicides only
- Lactational amenorrhoea method are not acceptable methods of contraception.
- Female condom and male condom should not be used together.

Acceptable methods of birth control in clinical trials:

- Total abstinence (when this is in line with the preferred and usual lifestyle of the patient. Periodic abstinence (e.g., calendar, ovulation, symptothermal, post-ovulation methods) and withdrawal are NOT acceptable methods of contraception.
- Female sterilisation (have had surgical bilateral oophorectomy with or without hysterectomy) or tubal ligation at least six weeks before taking study treatment. In case of oophorectomy alone, only when the reproductive status of the woman has been confirmed by follow-up hormone level assessment.
- Male sterilisation (at least 6 months prior to screening). For female patients on the study the vasectomised male partner should be the sole partner for that patient.
- BOTH of the following forms of contraception must be utilised:
  - a Use of oral, injected or implanted hormonal methods of contraception or other forms of hormonal contraception that have comparable efficacy (failure rate <1%), for example hormone vaginal ring or transdermal hormone contraception.
  - b Barrier methods of contraception: Condom or Occlusive cap (diaphragm or cervical/vault caps) with spermicidal foam/gel/film/cream/vaginal suppository
- Use of IUDs are excluded due to increased risks of infection and bleeding in this population.
- In case of use of oral contraception, women must be stable on the same pill for a minimum of 3 months before taking study treatment.

Women who are not of reproductive potential (defined as either <11 years of age, Tanner Stage 1, post-menopausal for at least 24 consecutive months or have undergone hysterectomy, salpingectomy, and/or bilateral oophorectomy) are eligible without requiring the use of

contraception. Acceptable documentation includes written or oral documentation communicated by clinician or clinician's staff of one of the following:

- Demographics show age <11 years.
- Physical examination indicates Tanner Stage 1.
- Physician report/letter.
- Operative report or other source documentation in the patient record.
- Discharge summary.
- Follicle stimulating hormone measurement elevated into the menopausal range.

Signature Page for VV-CLIN-000867 v1.0

Approval	Wolfram Brugger<br>Medical<br>12-Jul-2021 10:59:34 GMT+0000

Signature Page for VV-CLIN-000867 v1.0
