# Supplementary material for: TRBC1-CAR T cell therapy in peripheral T cell lymphoma: a phase 1/2 trial
Source: Nat Med. 2024 Nov 11;31(1):137–43. doi: 10.1038/s41591-024-03326-7 (PMC11750712; doi:10.1038/s41591-024-03326-7)
Supplement: Supplementary file 2 — Reporting Summary [file 41591_2024_3326_MOESM2_ESM.pdf]

nature portfolio

Corresponding author(s): Martin Pule

Last updated by author(s): Sep 18, 2024

Reporting Summary

Nature Portfolio wishes to improve the reproducibility of the work that we publish. This form provides structure for consistency and transparency in reporting. For further information on Nature Portfolio policies, see our Editorial Policies and the Editorial Policy Checklist.

Statistics

For all statistical analyses, confirm that the following items are present in the figure legend, table legend, main text, or Methods section.

n/a	Confirmed	
☐	☒	The exact sample size (*n*) for each experimental group/condition, given as a discrete number and unit of measurement
☐	☒	A statement on whether measurements were taken from distinct samples or whether the same sample was measured repeatedly
☐	☒	The statistical test(s) used AND whether they are one- or two-sided<br>*Only common tests should be described solely by name; describe more complex techniques in the Methods section.*
☒	☐	A description of all covariates tested
☒	☐	A description of any assumptions or corrections, such as tests of normality and adjustment for multiple comparisons
☐	☒	A full description of the statistical parameters including central tendency (e.g. means) or other basic estimates (e.g. regression coefficient) AND variation (e.g. standard deviation) or associated estimates of uncertainty (e.g. confidence intervals)
☐	☒	For null hypothesis testing, the test statistic (e.g. *F*, *t*, *r*) with confidence intervals, effect sizes, degrees of freedom and *P* value noted<br>*Give P values as exact values whenever suitable.*
☒	☐	For Bayesian analysis, information on the choice of priors and Markov chain Monte Carlo settings
☒	☐	For hierarchical and complex designs, identification of the appropriate level for tests and full reporting of outcomes
☒	☐	Estimates of effect sizes (e.g. Cohen's *d*, Pearson's *r*), indicating how they were calculated

Our web collection on statistics for biologists contains articles on many of the points above.

Software and code

Policy information about availability of computer code

Data collection	Clinical data capture system was set up in Encapsia and managed by a third party vendor, Aixial Group. The Investigator or his/her designee entered Electronic Case Report Form (eCRF) data directly into the data capture system. Flow cytometry data acquired using BD FACSLyric (BD Biosciences) and .MacsQuantX (Miltenyi) Immuno histochemistry data acquired on the Leica Bond RX platform (Leica Biosystems). Multispectral imaging aquired on the Phenoimager HT platform (Akoya biosciences).
Data analysis	Encapsia electronic data capture (EDC) system v1.0. SAS® Life Science Analytics Framework v9.4. GraphPad Prism V10.1.2. QuPath v0.4.3. InForm software v3.0, FlowJo v10 (Treestar, RRID:SCR_008520), FCS Express (De novo software) v7.22.0031

For manuscripts utilizing custom algorithms or software that are central to the research but not yet described in published literature, software must be made available to editors and reviewers. We strongly encourage code deposition in a community repository (e.g. GitHub). See the Nature Portfolio guidelines for submitting code & software for further information.

Data

Policy information about availability of data

All manuscripts must include a data availability statement. This statement should provide the following information, where applicable:

- Accession codes, unique identifiers, or web links for publicly available datasets
- A description of any restrictions on data availability
- For clinical datasets or third party data, please ensure that the statement adheres to our policy

Individual de-identified participant data not disclosed. Study protocol available in Supplementary Information. Statistical analysis plan can be requested after clinical trial NCT03590574 completion. Researchers who provide an analysis proposal which complies with clinical study ethical and data integrity requirements can request the relevant information to the corresponding author (m.pule@autolus.com). Requests will be evaluated within 30 days.

Research involving human participants, their data, or biological material

Policy information about studies with human participants or human data. See also policy information about sex, gender (identity/presentation), and sexual orientation and race, ethnicity and racism.

Reporting on sex and gender	All patients signed an informed consent form before the start of screening procedures. Both men and women aged ≥ 18 years could participate in the study. Sex or gender information was collected based on self-reporting and recorded within the Electronic Case Report Form (eCRF). Study objectives were designed regardless of sex or gender. Hence, this information was not used for data analysis.
Reporting on race, ethnicity, or other socially relevant groupings	None of these categorization variables was used.
Population characteristics	Relevant population characteristics of the human research participants are the following: PTCL lymphoma subtype including PTCL-NOS, AIT, ALCL, medical history and prior lymphoma treatments. Median Age 55 (range 34-63). Male and female patients included in the study. Patients characteristics on Table 1.
Recruitment	Participants were identified by the direct healthcare team as part of routine clinical practice and by review of medical records/ clinic database held within the institutions. Patients with confirmed diagnosis of TRBC1 positive selected T cell non -Hodgkin lymphoma (T-NHL) who have relapsed or become refractory after exposure to ≥1 line of therapy were included in the study. Complete list of inclusion and exclusion criteria are listed in Extended Data Table 1 and 2, and in the study protocol in Supplementary Information.<br>Patients were recruited from 5 active sites: 4 based in the UK and 1 in Spain.
Ethics oversight	The study was approved in the UK by the UK Medicines and Healthcare Products Regulatory Agency (clinical trial authorization no. CTA46113/004/001-0011), the London/West London GTAC Research Ethics Committee (REC ref no. 17/LO/1730) and the research and development departments of all participating National Health Service trusts. The study was approved in Spain by the Spanish Agency of Medicines and Medical Products under EudraCT number 2017-001965-26. The study was managed by Autolus. Written informed consent was obtained from patients prior to study entry in accordance with the Declaration of Helsinki. Use of human donor derived products was performed under approval of the Human Tissue Authority (HTA license 12642).

Note that full information on the approval of the study protocol must also be provided in the manuscript.

Field-specific reporting

Please select the one below that is the best fit for your research. If you are not sure, read the appropriate sections before making your selection.

☒ Life sciences ☐ Behavioural & social sciences ☐ Ecological, evolutionary & environmental sciences

For a reference copy of the document with all sections, see nature.com/documents/nr-reporting-summary-flat.pdf

Life sciences study design

All studies must disclose on these points even when the disclosure is negative.

Sample size	This paper described a rolling six (Skolnik et al. 2008) dose escalation design in Phase I of the study with test at 4 dosing levels: 25 x 10^6, 75 x 10^6, 225 x 10^6, and 450 x 10^6 RQR8/aTRBC1-CAR positive T cells. A minimum of 3 patients was planned to be treated per cohort, and could be expanded from 3 to 6 patients when 1 patient has a dose limiting toxicity (DLT).
Data exclusions	Patients who got enrolled but discontinued before the study treatment, or pending AUTO4 infusion were not included in the efficacy/safety analyses.
Replication	Patients with Relapsed or Refractory TRBC1 Positive Selected T Cell Non-Hodgkin Lymphoma who met all the inclusion/exclusion criteria were enrolled into the study. The study can be repeated later for patients using same inclusion/exclusion criteria. In vitro CAR T cell cytotoxicity and

	reverse killing data was performed across 6 independent healthy T cell donors.
Randomization	This is a single-arm, open-label study. No randomization was applied.
Blinding	Blinding was not applied since this is a single-arm, open-label study.

Reporting for specific materials, systems and methods

We require information from authors about some types of materials, experimental systems and methods used in many studies. Here, indicate whether each material, system or method listed is relevant to your study. If you are not sure if a list item applies to your research, read the appropriate section before selecting a response.

Materials & experimental systems

n/a	Involved in the study	
☐	☒	Antibodies
☐	☒	Eukaryotic cell lines
☒	☐	Palaeontology and archaeology
☒	☐	Animals and other organisms
☐	☒	Clinical data
☒	☐	Dual use research of concern
☒	☐	Plants

Methods

n/a	Involved in the study	
☒	☐	ChIP-seq
☐	☒	Flow cytometry
☒	☐	MRI-based neuroimaging

Antibodies

Antibodies used	Anti-HLA DR (BD 564040); Anti-CD8 (BD 612942);Anti-CD27 (BD 741833); Anti-CD4 (BD 612887); Anti-CXCR3 (BD 562558); Anti-CD45RA (BD 566114); Anti-CD2 (Biolegend 300224); Anti-CD62L (BD 563808); Anti-TIM3 (BD 565566); Anti-CD25 (BD 563701); Anti-LAG3 (Biolegend 369308); Anti-Tigit (Invitrogen 46-9500-42); Anti-CCR7 (Biolegend 353236); Anti-PD-1 (BD 561272); Anti-CD3 (BD 345767); Anti-GrB (BD 560213); Anti-CD45 (BD 332784); Anti-CD3 (Biolegend 300448); Anti-CD4 (Biolegend 344646); Anti-CD8 (BD 335822); Anti-CD19 (BD 563325); Anti-TCR (Santa Cruz Biotechnology); Anti-CD34 (R&D systems FAB7227P); Anti-CD34 (QBend10, Leica biosystems); Anti_CD3 (NCL-L-CD3565, Leica Biosystems); Anti-KI67 (MIB-1, Leica Biosystems); Anti-CD4 (CD4-368-L-CE, Leica Biosystems, UK); Anti-CD8 (CD8-4B11-L-CE, Leica Biosystems, UK); Anti-FOXP3 (236A/E7, Abcam, UK); Anti-PD1 (NAT105, Abcam, UK); Anti-PDL1 (SP263, Roche Diagnostics Limited, UK)
Validation	Anti-HLA DR (BD 564040) validated on human peripheral blood lymphocytes and monocytes. Anti-CD8 (BD 612942) routinely tested on flow cytometry. Anti-CD27 (BD 741833) flow cytometry qualified. Anti-CD4 (BD 612887) validated on human peripheral blood lymphocytes. Anti-CXCR3 (BD 562558) validated on human peripheral blood lymphocytes. Anti- CD45RA (BD 566114) validated on human peripheral blood lymphocytes; Anti-CD2 (Biolegend 300224) validated on human peripheral blood lymphocytes; Anti-CD62L (BD 563808) validated on human peripheral blood lymphocytes; Anti-TIM3 (BD 565566) validated on human peripheral blood lymphocytes; Anti-CD25 (BD 563701) validated on stimulated and unstimulated human peripheral blood lymphocytes; Anti-LAG3 (BioLegend 369308) flow cytometry quality testing; Anti-Tigit (Invitrogen 46-9500-42) validated on normal human peripheral blood cells; Anti-CCR7 (Biolegend 353236) validated on human peripheral blood lymphocytes; Anti-PD-1 (BD 561272) validated on human peripheral blood lymphocytes; Anti-CD3 (BD 345767) routinely tested in flow cytometry; Anti-GrB (BD 560213) valideted on peripheral blood CD8+ lymphocytes; Anti-CD45 (BD 332784) routinely tested in flow cytometry; Anti-CD3 (Biolegend 300448) validated on human peripheral blood lymphocytes; Anti-CD4 (Biolegend 344646) validated on human peripheral blood lymphocytes; Anti-CD8 (BD 335822) routinely tested in flow cytometry; Anti-CD19 (BD 563325) validated on human peripheral blood lymphocytes; Anti-TCR (Santa Cruz Biotechnology) validated in western blot analysis of TCR C β 1 expression in human PBL whole cell lysate and Direct FCM analysis of human peripheral blood leukocytes; Anti-CD34 (R&D systems FAB7227P) validated on Human peripheral blood mononuclear cells; Anti-CD34 (QBend10, Leica biosystems) Validated in IHC; Anti_CD3 (NCL-L-CD3565, Leica Biosystems) Validated in IHC; Anti-KI67 (MIB-1, Leica Biosystems) Validated in IHC; Anti-CD4 (CD4-368-L-CE, Leica Biosystems, UK) validated in IHC; Anti-CD8 (CD8-4B11-L-CE, Leica Biosystems, UK) validated in IHC; Anti-FOXP3 (236A/E7, Abcam, UK) Validated in IHC; Anti-PD1 (NAT105, Abcam, UK) Validated in IHC;; Anti-PDL1 (SP263, Roche Diagnostics Limited, UK) Validated in IHC.<br><br>All antibodies were routinely tested in our laboratory

Eukaryotic cell lines

Policy information about cell lines and Sex and Gender in Research

Cell line source(s)	Jurkat TRBC1+ were obtained from the American Type Culture Collection. Jurkat TCR KO were engineered from Jurkat TRBC1 +. HEK293T obtained from the American Type Culture Collection
Authentication	Cell lines were obtained from cell bank repositories. Engineered cell lines were validated by flow cytometric stainings
Mycoplasma contamination	Cell lines were tested negative for mycoplasma contamination
Commonly misidentified lines (See ICLAC register)	No commonly misidentified cell lines were used in this study

Clinical data

Policy information about clinical studies

All manuscripts should comply with the ICMJE guidelines for publication of clinical research and a completed CONSORT checklist must be included with all submissions.

Clinical trial registration	NCT03590574
Study protocol	Study protocol available as supplementary material
Data collection	Clinical data capture system was set up in Encapsia and managed by a third party vendor, Aixial Group. The Investigator or his/her designee entered Electronic Case Report Form (eCRF) data directly into the data capture system.
Outcomes	Primary and Secondary objectives and endpoints are described in Extended Data Table 3.<br>Disease response assessments were performed at protocol defined time points (pre-LD, months 1, 3,6,9,12,15,18,24) by 18FDG PET-CT according to the Response Criteria for Non-Hodgkin Lymphoma -Lugano Classification. All subjects had disease status evaluation within 4 weeks of initiation of lymphodepleting chemotherapy (LD).

Plants

Seed stocks	N/A
Novel plant genotypes	N/A
Authentication	N/A

Flow Cytometry

Plots

Confirm that:

☒ The axis labels state the marker and fluorochrome used (e.g. CD4-FITC).

☒ The axis scales are clearly visible. Include numbers along axes only for bottom left plot of group (a 'group' is an analysis of identical markers).

☒ All plots are contour plots with outliers or pseudocolor plots.

☒ A numerical value for number of cells or percentage (with statistics) is provided.

Methodology

Sample preparation

Frozen drug product and Leukapheresis characterization experiments included fluorescent minus multiple controls (FMX), PBMC and single stained UltraComp eBeads™ Compensation Beads (ThermoFisher Scientific, UK) to determine gating thresholds and calculate compensation. Samples were, rested overnight in TexMACS10 medium (Miltenyi Biotec) post thaw. Samples were stained with a Fixable viability dye (BD Horizon™) and then blocked (Miltenyi Biotec). Phenotypic Characterization was performed using antibodies for memory and exhaustion markers diluted in Brilliant Stain Buffer Plus (BD Horizon™). Staining for CCR7 was carried out prior to surface staining at 37°C for 15mins (Biolegend®). Intracellular staining was done using the Transcription Factor Buffer Set (eBioscience™) according to manufacturer's instructions. Transformed CAR cells were identified using CAR Anti-idiotype antibody and secondary donkey anti-rabbit conjugated to PE (Biolegend®).

For whole blood flow cytometry the surface assay follows a lyse wash protocol, for each sample an FMO control was generated to determine CAR positivity. The compensation was set up using the Lyric software reference settings maintained daily by using the performance QC wizard. When creating reference settings single stained compensation beads (BD Horizon™, BD Biosciences, USA) were used. A volume of 100µL of whole blood was used for staining. The sample was blocked (Miltenyi Biosciences, Germany) followed by staining with a cocktail of antibodies to identify the immune cell subsets, and the presence of transformed CARs.
To assess viability, a fixable viability dye (BD Horizon™) was added to Lysis solution (BD Biosciences). The Live/Lysis solution was added to the blood and incubated. Cells were washed and resuspended in BD Stain buffer (BD Biosciences).
The sample was then transferred to a TruCount™ Tube (BD Biosciences) for acquisition and analysis on the BD FACSLyric (BD Biosciences).
TRBC1/TRBC2 ratio was determined using a surface assay following a two-step staining protocol using Fluorescence minus one controls and secondary control for TRBC2. Frozen PBMCs were thawed, blocked using Human FcR Block (Miltenyi Biotec) and stained for viability using Fixable Viability Stain 700 (BD Biosciences). Cells were washed and resuspended in a surface stain master mix containing anti-CD45 PerCP-Cy 5.5 (332784Biol, BD), anti-CD3 BV510 (300448, Biolegend), anti-CD4 BV605 (344646, Biolegend), anti-CD8 PE-Cy7 (335822, BD), anti-CD19 BV786 (563325, BD), anti-TCR beta 1 AF488 (Santa Cruz

Biotechnology, US), CD34 PE (FAB7227P, R&D systems) and anti-TRBC2 biotin (Autolus). Cells were washed and resuspended in a secondary only master mix containing Streptavidin antibody BV421 (405225, Biolegend). Cells were washed, resuspended, and results acquired BD FACSLyric (BD Biosciences).

For standard cytotoxicity assay, mock (Non-Transduced PBMCs) and CAR-transduced T cells were co-cultured with TRBC1+ or TRBC2+ non transduced T cells, TRBC1+, TRBC2+ or TCR KO Jurkat target cells. Target cells were labeled with CellTrace™ CFSE (C34554, ThermoFisher Scientific) following manufacturer instructions. Mock and CAR-transduced T cells were labeled with CellTrace™ Violet (C34557, ThermoFisher Scientific) following manufacturer instructions. Effector and target cells were mixed to reach an E:T ratio of 1:1, 1:2, 1:4 and 1:8. 72 h after co-culture live cell data were collected via Flow cytometry using the MacsQuantX flow cytometer (Milteny).

For reverse killing assay, mock and CAR-transduced T cells were co-cultured with autologous TRBC1+ and TRBC2+ non transduced T cells. Target cells were labeled with CellTrace™ CFSE (C34554, ThermoFisher Scientific) following manufacturer's instructions. Effector mock and CAR-transduced T cells were labeled with CellTrace™ Violet (C34557, ThermoFisher Scientific) following manufacturer's instructions. Effector and target cells were mixed to reach an E:T ratio of 1:4, 1:1 and 4:1. 72 h after co-culture live cell data were collected via Flow cytometry using the MacsQuantX flow cytometer (Miltenyi).

Instrument

LBS FACSLyric (BD Bioscience), MacsQuantX flow cytometer (Miltenyi)

Software

Lyric software (BDFACS Suite) for data collection, FCS Express software v7.22.0031 (De Novo software, US) and FlowJo v10 (Treestar, RRID:SCR_008520) for data analysis

Cell population abundance

No sorting was performed

Gating strategy

1) CD45 vs SSC-A to define leucocytes 2) Intact cells are gated on FSC-A vs SSC-A 3) FCS-A vs FCS-H is used to gate on single cells. 4) Live cells are gated using FVS-700 vs SSC-A. 5) CD3 vs CD19 is used to define lymphocytes 6) CD3 positive lymphocytes are dived by TRBC1 positive and TRBC2 positive populations 7) CD3 lymphocytes are subdivided into CD4 and CD8. 8) CD3 CAR positive population is defined by RQR8 positivity using CD3 vs RQR8 with the negative population labelled as CD3 CAR negative 9) CD4 positive cells are divided into TRBC1 positive and TRBC2 positive populations. 10) CD8 positive cells are divided into TRBC1 positive and TRBC2 positive populations.

☒ Tick this box to confirm that a figure exemplifying the gating strategy is provided in the Supplementary Information.
